# The winding roads to adulthood: A twin study

**DOI:** 10.1002/jcv2.12053

**Published:** 2021-12-11

**Authors:** Kaili Rimfeld, Margherita Malanchini, Amy E. Packer, Agnieszka Gidziela, Andrea G. Allegrini, Ziada Ayorech, Emily Smith‐Woolley, Andrew McMillan, Rachel Ogden, Philip S. Dale, Thalia C. Eley, Robert Plomin

**Affiliations:** ^1^ Social, Genetic and Developmental Psychiatry Centre Institute of Psychiatry, Psychology and Neuroscience King's College London London UK; ^2^ Department of Psychology Queen Mary University of London London UK; ^3^ Department of Psychology PROMENTA Research Center University of Oslo Oslo Norway; ^4^ Nic Waals Institute Oslo Norway; ^5^ Population Health Sciences Bristol Medical School University of Bristol Bristol UK; ^6^ Medical Research Council Integrative Epidemiology Unit University of Bristol Bristol UK; ^7^ Department of Speech and Hearing Sciences The University of New Mexico Albuquerque New Mexico USA

**Keywords:** behavioural genetics, emerging adulthood, mental health, personality, physical health, wellbeing

## Abstract

**Aims:**

Here we report the results of the first systematic investigation of genetic and environmental influences on 57 psychological traits covering major issues in emerging adulthood such as aspirations, thoughts and attitudes, relationships and personality. We also investigate how these traits relate to physical and mental health, educational attainment and wellbeing.

**Materials & Methods:**

We use a sample of nearly 5000 pairs of UK twins aged 21–25 from the Twins Early Development Study. We included 57 measures of traits selected to represent issues in emerging adulthood (EA) such as aspirations, thoughts and attitudes, life events, relationships, sexual and health behaviour and personality. We also included measures related to what are often considered to be the core functional outcomes even though here we refer to the data collected at the same time: adverse physical health, adverse mental health, wellbeing, and education.

**Results:**

All 57 traits showed significant genetic influence, with an average heritability of 34% (SNP heritability ~10%). Most of the variance (59% on average) was explained by non‐shared environmental influences. These diverse traits were associated with mental health (average correlation 0.20), wellbeing (0.16), physical health (0.12) and educational attainment (0.06). Shared genetic factors explained the majority of these correlations (~50%). Together, these emerging adulthood traits explained on average 30% of variance in the outcomes (range = 8% to 69%), suggesting that these traits relate to the outcomes additively.

**Discussion & Conclusions:**

We conclude that even as the majority of individual differences in EA traits is explained by non‐shared environmental factors, genetic influence on these traits is still substantial; the environmental uncertainties of emerging adulthood in the 21st century do not diminish the importance of genetics. As adolescents travel down long and winding roads to adulthood, their trip is substantially influenced by genetic proclivities that nudge them down different paths leading to different destinations.

## Graphical abstract

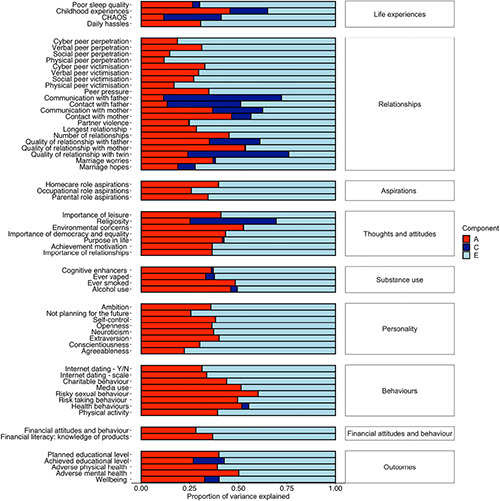

Univariate twin analyses of additive genetic (A), shared environmental (C), and non‐shared environmental (E) components of variance for emerging adulthood traits.

**Key points**

- In the 21st century, emerging adulthood has stretched from the late teens through the twenties
- The first systematic investigation of genetic and environmental influences on 57 psychological traits covering major issues in emerging adulthood showed an average heritability of 34%. Most of the variance (59% on average) was explained by non‐shared environmental influences
- These diverse traits were associated with mental health (average correlation 0.20), wellbeing (0.16), physical health (0.12), and educational attainment (0.06). Shared genetic factors explained the majority of these correlations (∼50%)
- Together, these emerging adulthood traits explained on average 30% of variance in the outcomes (range = 8 to 69%), suggesting that these traits relate to the outcomes additively

## INTRODUCTION

The winding road to adulthood is the subtitle of the field‐defining book on emerging adulthood (EA) by Jeffrey Arnett (Arnett, 2004, 2015). The book documents the recent societal changes—for example, insecure employment, unstable relationships, difficulties in owning a home—that have prolonged the transition from adolescence to adulthood, a process which he called *emerging adulthood*. The transition from adolescence to adulthood is marked by instability, self‐focus, feeling in‐between, exploring identity in work and love, and an optimistic sense of possibilities.

More than 6000 papers, primarily in psychology and psychiatry, have investigated emerging adulthood. Most of these are normative, describing average changes that take place during the twenties (Arnett, 2016). An enormous menu of experiences is on offer during emerging adulthood for exploring self, relationships, aspirations, attitudes, autonomy and occupations. The bespoke nature of these experiences is amplified by the Internet and social media. The current generation of 20+‐year‐olds is the first generation of digital natives. However, the paths individuals take can vary widely. Much less is known about individual differences, which are a key feature of emerging adulthood as the transition to adulthood is less lock‐stepped than in the past.

Genetic and environmental analyses of individual differences in emerging adulthood have been reported for domains that are assessed throughout the life course (Bergen et al., 2007). These include psychopathology (Polderman et al., 2015), personality (Turkheimer et al., 2014) and cognitive abilities (Briley & Tucker‐Drob, 2013). However, much less is known about the genetic and environmental aetiology of individual differences in traits especially salient in emerging adulthood, such as identity, aspirations, and relationships. Two exceptions are political orientation and participation (Hufer et al., 2020; Kornadt et al., 2018) and religiousness (Koenig et al., 2008). Interestingly, these studies generally suggest greater genetic influence and less shared environmental influence in emerging adulthood than in adolescence, especially for political ideology (Hatemi & McDermott, 2012).

The great heterogeneity of experience during emerging adulthood suggests the hypothesis that environmental differences are especially important. These environmental factors could be societal or historical or could be explained by family environment or socioeconomic factors. In addition, environmental factors specific to the individual could play an important role, such as health‐related factors, relationships and employment (Wood et al., 2018). A refinement of this hypothesis of heightened environmental influence is that any residual effect of family environment is overwhelmed as emerging adults make their own way in the world. The instability and uncertainty of emerging adulthood produces idiosyncratic experiences that nudge individuals down different paths. Such experiences are not likely to be shared by siblings, which in behavioural genetics is called non‐shared environment. Therefore, non‐shared environment, defined as all environmental factors that do not contribute to similarities between siblings growing up in the same family, could play an important role in emerging adulthood (Knopik et al., 2017).

A less obvious possibility is that, despite the heterogeneity of environmental experiences in emerging adulthood, genetic influence on individual differences remains substantial. When a larger proportion of individual differences is explained by environmental factors, as is expected in emerging adulthood traits, then a smaller proportion of variance is explained by inherited DNA differences between people (Knopik et al., 2017). However, genetic factors could still be important, which is the trend found in the few relevant twin studies of emerging adulthood (Hufer et al., 2020; Koenig et al., 2008; Kornadt et al., 2018). One major mechanism by which genotypes become phenotypes is by affecting how individuals select, modify and create experiences in part on the basis of their genetic propensities. This is called gene–environment correlation, that is, the environmental factors individuals choose are correlated with individuals' genetic propensities (Knopik et al., 2017). For this reason, it is possible that the instability and uncertainties of emerging adulthood create more opportunities for individuals to make choices and select increasingly diverse life experiences that are correlated with their genotypes.

The specific choices, aspirations, thoughts and feelings of emerging adults are likely to be related to outcomes such as physical and mental health, wellbeing and educational attainment (Bonnie et al., 2015; Johnson et al., 2011). The extended transition from adolescence to adulthood is associated with a prolonged period of risk‐taking, decision‐making and self‐regulation with markedly less parental control and parental influence on lifestyle, exercise and diet (Bonnie et al., 2015). These factors are all prone to be associated with physical and mental health and wellbeing. Emerging adults, for the first time, can make decisions about their daily life, such as sleeping, eating, exercise and entertainment. These decisions are likely to be linked to their personality and life goals, relationships and aspirations for life (Harris et al., 2006).

On average, the physical health of emerging adults tends to be better than that of older adults. For example, 96% of the US emerging adults report being in excellent, very good or good health (Park et al., 2006). Nonetheless, emerging adults differ, and poor health during this period predicts early onset of chronic conditions such as cardiovascular problems that increase in general later in life (Tanner, 2016; Yaffe et al., 2014). However, little is known about how psychological traits, especially salient in emerging adulthood, are associated with individual differences in physical health.

Unlike physical illness, mental illness is highly prevalent during emerging adulthood, as most mental illness begins to emerge by adolescence (Kessler et al., 2005; Tanner, 2016). The US National Survey of Comorbidity reports that up to 44% of young adults meet the criteria for a mental health disorder in a given year. Also important is wellbeing which is more than the absence of mental illness and is broadly defined as a spectrum of positive feelings and positive life assessments (Bartels, 2015). It is likely to be related to many traits especially relevant to emerging adulthood (Ong & Bergeman, 2004; Ong et al., 2006; van de Weijer et al., 2020; Veenhoven, 2008). For example, personality traits, especially neuroticism and extraversion, play a role in individual differences in wellbeing and mental health (Anglim et al., 2020; Baselmans et al., 2019). However, little is known about the causes and correlates of wellbeing or mental health and illness during emerging adulthood.

Another key outcome in emerging adulthood is educational attainment, often defined as years spent in education, for those who continue their education beyond compulsory schooling. This is especially important since educational outcomes enable young adults to pursue different lifelong trajectories. Importantly, educational attainment is related to physical and mental health across the lifespan (Cutler & Lleras‐Muney, 2012). Educational achievement, actual school performance, can be explained by a package of heritable cognitive and non‐cognitive traits, such as personality, behavioural problems and self‐esteem (Krapohl et al., 2014). However, less is known about the correlates of education‐related traits during emerging adulthood, such as the relationship between career goals, aspirations and family relationships.

The current study investigates the genetic and environmental aetiology of individual differences in traits especially relevant to emerging adulthood, including life goals, purpose in life, relationships, and attitudes towards marriage, money and occupation. These traits were selected based on the themes introduced by Arnett's field‐defining book on emerging adulthood (Arnett, 2015). We refer to these traits as EA traits. This is the first report of an emerging adulthood assessment of twins in the Twins Early Development Study (TEDS) when the twins were 21–25 years old (Rimfeld et al., 2019).

Our second objective is to examine the genetic and environmental aetiology of the extent to which these emerging adulthood traits are associated with physical health, mental health and illness, wellbeing and educational outcomes and aspirations.

Although this study was an exploratory study due to the limited research on emerging adulthood, we made some general predictions based on the results of behavioural genetic research throughout the life course. These predictions, together with the analysis plan, were preregistered in Open Science Framework (osf.io/m6vwj) prior to accessing the data.

1. All psychological traits and key functional outcomes will show substantial heritability (∼40%) and the proportion of individual differences explained by shared environmental factors will be modest (less than 10%); the rest of the variance will be explained by non‐shared environmental influences.
2. Traits with higher twin heritability will also have higher heritability estimates calculated from DNA of unrelated individuals (i.e., SNP heritability). SNP heritability will be significant and substantial for psychological traits (average SNP h2 around 10%). The gap between twin heritability and SNP heritability will be substantial; twin heritability will generally be roughly twice the SNP heritability.
3. Phenotypic correlations between psychological traits and outcome measures (physical health, mental health, wellbeing and educational attainment) will be significant and moderate (average correlation ∼0.30).
4. Genetic correlations between psychological traits and outcome measures (physical health, mental health, wellbeing and educational attainment) will be substantial (0.5–0.7). More than half of the phenotypic correlations between psychological traits and outcome measures (physical health, mental health, wellbeing and educational attainment) will be explained by genetic factors.
5. There will be significant mean differences between males and females in some psychological traits with males showing more risk‐taking, anti‐social behaviour, alcohol and substance use compared to females; and females reporting more anxiety, depression and victimization, but also more volunteering. We do not expect large differences between males and females in key functional outcomes. While many of the mean differences will be significant given our large sample size, we predict that the effect sizes will be small with variance explained by gender less than 5%.
6. There will be no differences between males and females in the aetiology of psychological traits in emerging adulthood, that is, we predict that there will be no significant qualitative or quantitative genetic differences between males and females.

## METHODS

### Sample

Our sample was drawn from TEDS, a longitudinal study that recruited through national birth records over 16,000 twin pairs born between 1994 and 1996 in England and Wales. Although there has been some attrition, more than 8000 twin pairs are still engaged in the study, and TEDS remains reasonably representative of the population in England and Wales in terms of ethnicity and family socio‐economic factors, including data from the most recent data collection used in the present study (Rimfeld et al., 2019). Zygosity was assessed using a parent‐reported questionnaire of physical similarity, which has been shown to be over 95% accurate when compared to DNA testing (Price et al., 2000). When zygosity was not clear, DNA testing was used to resolve zygosity.

Here we used the subsample of twins who had contributed to the TEDS‐21 data collection. At the start of TEDS‐21 data collection, twin ages ranged from 20.56 to 25.59 years (mean = 22.29, SD = 0.92). All data were collected using questionnaires. Due to the length and number of measures included, data collection was completed in two phases (phase 1 started in June 2017, phase 2 started in February 2018, and both continued until January or February 2019). In both phases of TEDS‐21 questionnaire data collection, participants could complete the assessment via a smartphone app, via the web, or on paper. Incentives included a £10 voucher on completion of each phase. Twins were also offered entries in prize draws at intervals during the data collections of phases 1 and 2. Ethical approval was provided by the King's College London Research Ethics Committee (reference number: PNM/09/10‐104) and informed written consent was received from all participants. No exclusions were applied. All available data were used (*N* = 10,614, twins who had completed at least one phase of TEDS‐21 data collection), therefore, the sample size varied between measures. Sample size by zygosity for each measure is presented in Tables S1 and S2 (one twin randomly selected from each pair so that the data points are independent).

### Measures

The TEDS measures have been summarised previously (Rimfeld et al., 2019). Figure 1A lists the measures included in this study, which were administered during TEDS‐21 data collection; a more detailed description of measures can be found in Table S3. We included 57 measures of traits selected to represent issues in EA such as aspirations, thoughts and attitudes, life events, relationships, sexual and health behaviour and personality. We also included measures related to what are often considered to be the core functional outcomes even though here we refer to the data collected at the same time: adverse physical health (e.g., over the counter painkillers taken, hospitalisations, self‐reported health), adverse mental health (e.g., variables covering diverse forms of psychopathology), wellbeing (e.g., financial wellbeing, satisfaction with relationships and community), and education. These variables were used to create factors representing wellbeing, adverse mental health, and adversephysical health using principal component analyses (PCA). These composite measures, including their factor loadings, are presented in Figure 1B. Two items were used that assessed the highest educational level achieved at the time of data collection and the highest planned educational level for the future. These two measures were used separately and served as an index of educational attainment.

FIGURE 1(A) Summary of emerging adulthood measures. (B) Summary of functional outcomes; physical and mental health, wellbeing, achievement and planned educational level. Outer ring includes factor loadings to First Principal Component (PC1) for each outcome; third ring shows the % of variance explained by PC1 across measures

**Figure:**
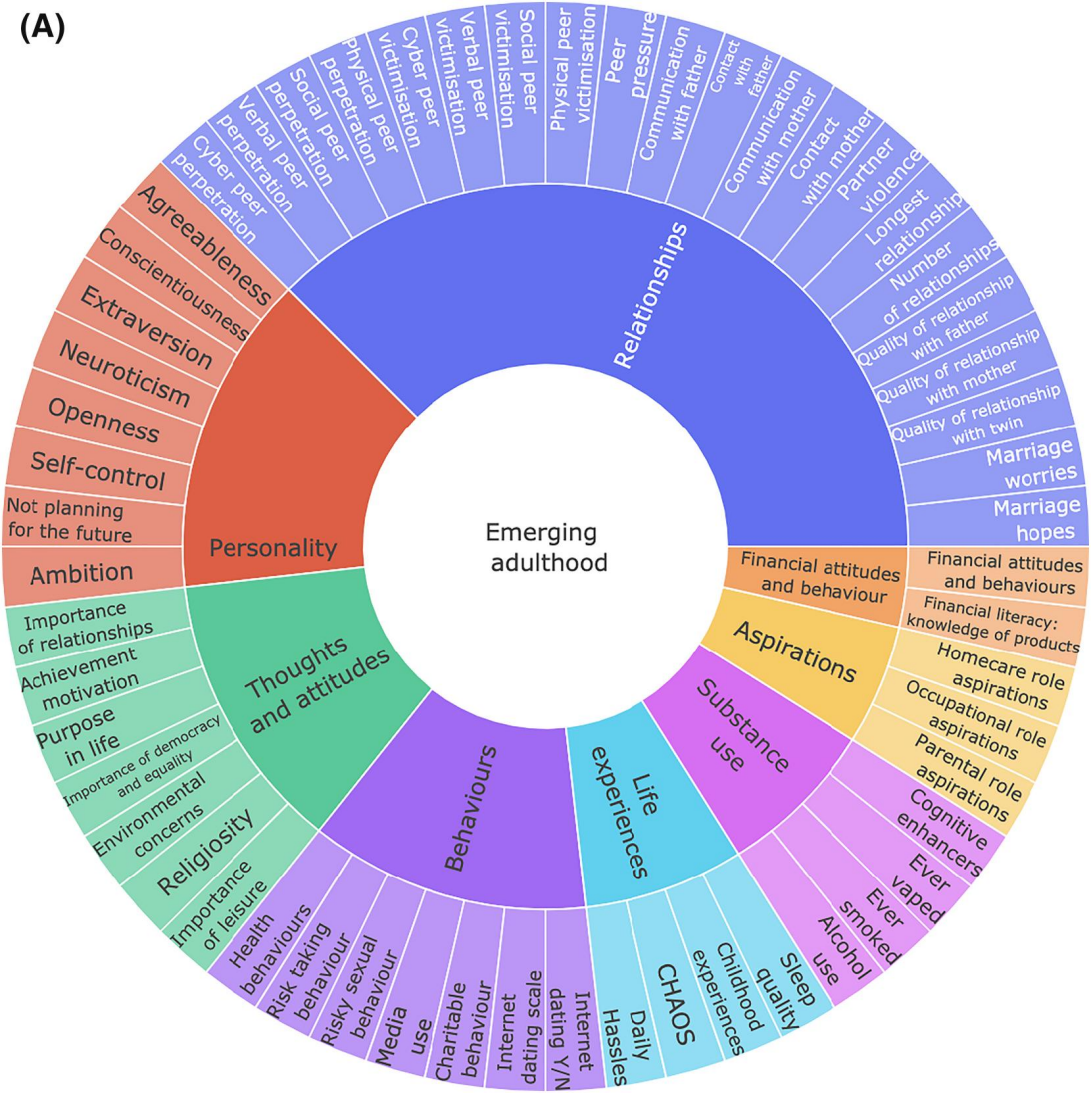

**Figure:**
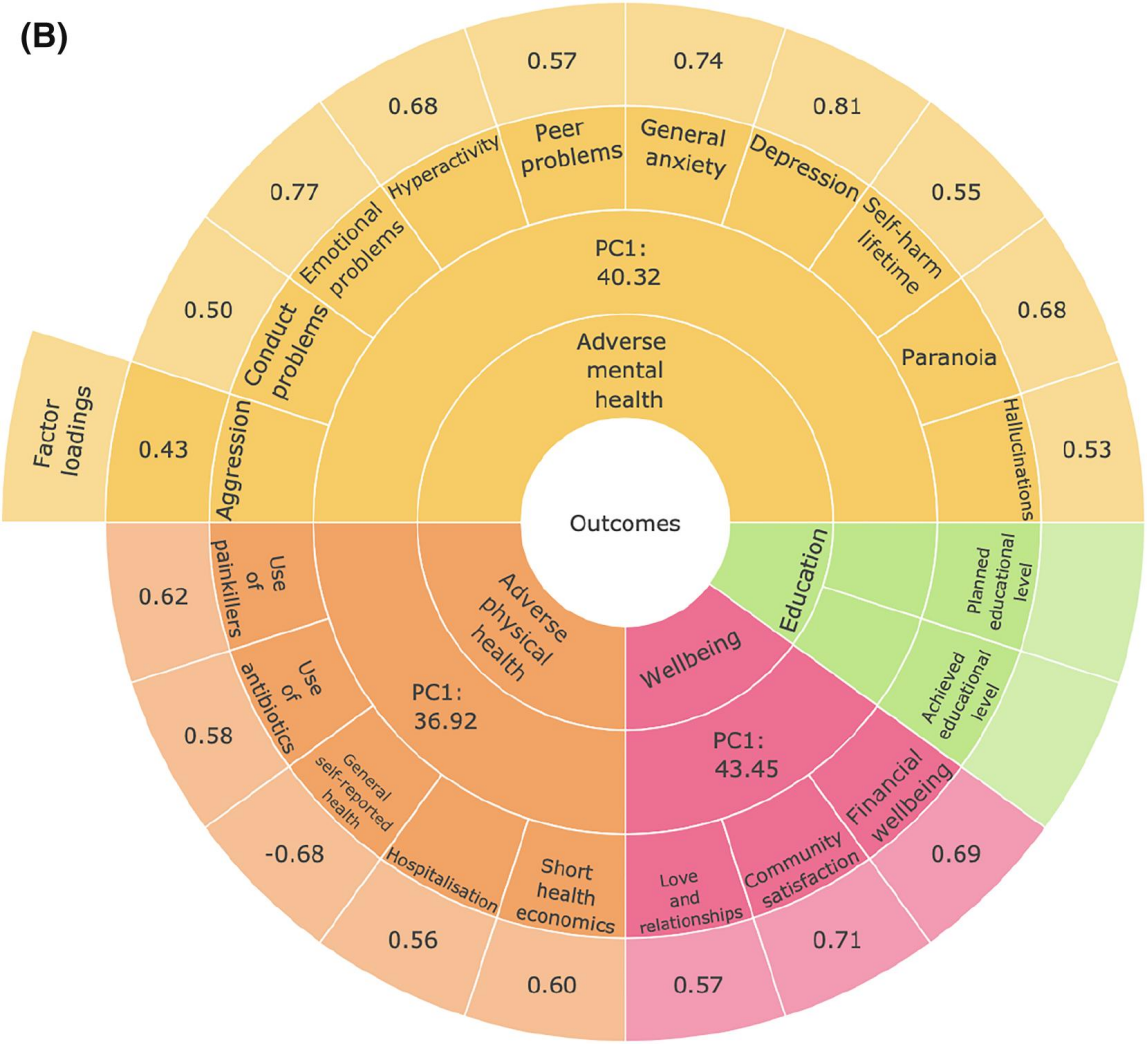

DNA samples have been obtained from 12,500 individuals and genotyped on one of two DNA microarrays (Affymetrix GeneChip 6.0 or Illumina HumanOmniExpressExome chips). After stringent quality control, the total sample size available for genomic analyses is 10,346 (including 7026 unrelated individuals and 3320 additional DZ co‐twins). Of these, 7289 individuals were genotyped on Illumina arrays, and 3057 individuals were genotyped on Affymetrix arrays (Rimfeld et al., 2019; see Selzam et al. [2018] for a detailed description of genotyping and quality control). DNA data were used for genome‐based restricted maximum likelihood methods using genome‐wide complex trait analyses (see genetic analyses). The sample size for DNA‐based analyses varied between measures.

### Quality control

Data cleaning (see https://www.teds.ac.uk/datadictionary/studies/rawdata/web_data_cleaning.htm#prandomteds21) included the identification of random responders, or “clickers”. Clickers were identified from a combination of the following: incorrect responses on a quality control item (e.g., we asked participants to select option ‘b’), too rapid responding (based on the mean item response time; web and smartphone app respondents only), or too uniform responding (i.e., selecting the same response over a series of consecutive items). Typically, around 1% of twins were excluded per questionnaire section but this varied from 0.3% to 5.3% (and of course 0% in themes without any measure containing a QC item). Twin data were excluded for an entire questionnaire section if the twin was identified as a clicker for any measure within that theme. Furthermore, twin data were excluded for an entire questionnaire (phase 1 or phase 2) if the twin was identified as a clicker for two or more themes within that questionnaire. For further details, including percentages of quality control exclusions, see Appendix S1.

### Statistical analyses

Our statistical analysis plan was registered in the OSF, prior to creation of the dataset and prior to analysis (osf.io/m6vwj). Scripts have been made available on the OSF site. All analyses were completed using SPSS Statistics software (v26, IBM Corp.) and R version 4.0 (R Core Team, 2020).

### Data transformations

Several variables were highly skewed. We therefore used the rank‐based van der Waerden's transformation to achieve normality (Lehmann, 1975; Van Der Waerden, 1975). Sensitivity analyses were performed to examine the influence of the van der Waerden transformation on results. The results remained highly similar to untransformed data analyses. Results are reported both for untransformed data and transformed data (see Supporting Information S1).

### Phenotypic analyses

Because the present sample is a twin sample, we maintained independence of data by randomly selecting one twin per pair for all phenotypic analyses. We repeated analyses of descriptive statistics for the other half of the sample. These results are presented in Supporting Information S1 (Tables S4 and S5) and are highly similar.

The 57 psychological trait measures were described in terms of means and variance and compared between males and females and between identical and non‐identical twins. Univariate analysis of variance (ANOVA) was used to test for mean differences for sex and zygosity and their interaction. Because significant, though small, sex differences emerged (Table S1), we corrected all scores for mean sex differences using the regression method. Correcting for sex and age is important in the analysis of twin data because members of a twin pair are identical in age and identical twins are identical for sex, which, if uncorrected, would inflate twin estimates of shared environment (McGue & Bouchard, 1984). These age‐ and sex‐adjusted standardised residuals were used in all subsequent analyses. For analyses using transformed data, we conducted the van der Waerden transformation prior to residualizing for age and sex as has been recommended by Pain et al. (Pain et al., 2018).

We created standardised composite scores representing adverse mental health, adverse physical health and wellbeing using PCA. We obtained the first principal component (first PC) of behaviour problem/mental health phenotypes (see Figure 1B and Table S3 for information about scales used). We also obtained the first principal component of physical health and wellbeing measures (see Table S3 for scales used). The first PCs of wellbeing, adverse mental health and adverse physical health were saved for subsequent analyses. Two separate items were used to assess educational outcomes: level of current education and level of planned education. We did not compute an educational attainment composite because the sample had not reached an age when educational attainment could be assessed.

We calculated phenotypic correlations between the 57 traits and the five functional outcome factors (adverse mental health factor, adverse physical health factor, wellbeing factor and two educational attainment variables) for the whole sample. We then repeated the analyses separately for males and females.

We also conducted multiple regression to assess how much variance in the functional outcomes could be explained in total by the 57 traits, reporting the adjusted *R* ^2^ values.

### Genetic analyses

#### Univariate twin analyses

We applied the twin method, specifically the univariate ACE model, to investigate the aetiology of individual differences in psychological traits and functional outcomes (adverse physical health, adverse mental health, wellbeing, and educational attainment). This method capitalises on the fact that identical (monozygotic, MZ) twin pairs are genetically identical and share 100% of their genes, while non‐identical (dizygotic, DZ) twin pairs share on average 50% of their segregating genes. Shared environment is defined as aspects of the environment that make members of twin pairs similar to one another. Non‐shared environmental influences are unique to individuals and do not contribute to similarities between twins. Using these family relatedness coefficients, it is possible to estimate the relative influence of additive genetic (*A*), shared environmental (*C*), and non‐shared environmental (*E*) effects on the variance and covariance of phenotypes, by comparing intraclass correlations for MZ and DZ twins (Knopik et al., 2017). In the model, non‐shared environmental variance also includes any measurement error. Heritability can be roughly calculated by doubling the difference between MZ and DZ correlations, C can be calculated by subtracting heritability from the MZ correlation and *E* can be estimated by deducting the MZ correlation from unity (Rijsdijk & Sham, 2002). The ACE model assumes that genetic effects are largely additive; however, the twin method can also estimate the effects of non‐additive genetic variance (*D*; dominance effects). Non‐additive genetic variants can be detected if the DZ correlation is less than half the correlation between MZ twins (Knopik et al., 2017). These parameters can be estimated more accurately using structural equation modelling (SEM), which also provides 95% confidence intervals and estimates of model fit. The SEM program OpenMx was used for all model‐fitting analyses (Boker et al., 2011). We report MZ and DZ same‐sex twin intraclass correlations, model fit statistics and ACE estimates.

#### Sex‐limitation analyses

The univariate model can be extended to the full sex‐limitation model to explore both qualitative and quantitative sex differences in the aetiology of individual differences in psychological traits, wellbeing, adverse physical health, adverse mental health and educational attainment. Qualitative sex differences indicate that different genetic or environmental factors influence a phenotype in males and females. Quantitative sex differences are observed when the same genetic and environmental factors influence variance in a given phenotype for males and females, but the magnitude of their effects differs across sexes. (For a detailed explanation of the full sex limitation model, see Medland, 2004). ACE estimates are also reported separately for males and females.

#### Bivariate correlated factors solution

The univariate model can be extended to a bivariate model to investigate the aetiology of the covariance between two traits. We used the bivariate twin design to calculate genetic correlations and estimate the *A*, *C* and *E* components of covariance between psychological traits versus wellbeing, adverse physical health, adverse mental health and the two educational outcomes. The bivariate genetic method decomposes the covariance between phenotypes into *A*, *C* and *E* components by comparing the cross‐trait cross‐twin correlations between MZ and DZ twin pairs (Neale et al., 2005). This method also enables estimation of the genetic correlation (*rG*), indicating the extent to which the same genetic variants influence two phenotypes independent of their heritabilities. The shared environmental correlation (*rC*) and non‐shared environmental correlation (*rE*) can also be estimated (Knopik et al., 2017; Rijsdijk & Sham, 2002). The bivariate genetic model also allows estimation of the proportion of phenotypic correlation explained by genetic and environmental factors between variables (see Figure S1 for Cholesky composition). The proportion of phenotypic correlation explained by genetic factors is calculated by the genetic correlation between two variables weighted by their heritabilities. The proportion of phenotypic correlation explained by shared environmental and non‐shared environmental factors can also be estimated in a similar manner.

#### Genome‐based restricted maximum likelihood methods using genome‐wide complex trait analyses (GREML‐GCTA)

We used univariate GREML GCTA software to calculate SNP heritability for the 57 traits and adverse physical health, adverse mental health, wellbeing and educational attainment. GREML uses individual‐level genotypic data to estimate the narrow‐sense SNP h^2^, that is, the proportion of phenotypic variation explained by the additive effects of SNPs assessed on a genotyping array and imputed SNPs (Yang et al., 2013). Population stratification was controlled by using the 10 first principal components of the population structure as covariates in the model (based on the PCA), along with a categorical variable indexing genotyping batch. Univariate GREML can also be extended to bivariate GREML to decompose covariance between traits. Bivariate GREML‐GCTA was used to calculate genetic correlations between psychological traits and adverse physical health, adverse mental health, wellbeing and educational attainment (Lee et al., 2012).

In our analyses we used all data, that is, we used all available data points. Missingness was minimal per data collection phase but was larger when both phase 1 and phase 2 data were used. Table S1 presents the exact sample sizes for each EA trait.

## RESULTS

### Descriptive statistics and comparisons between males and females

**FIGURE 2:**
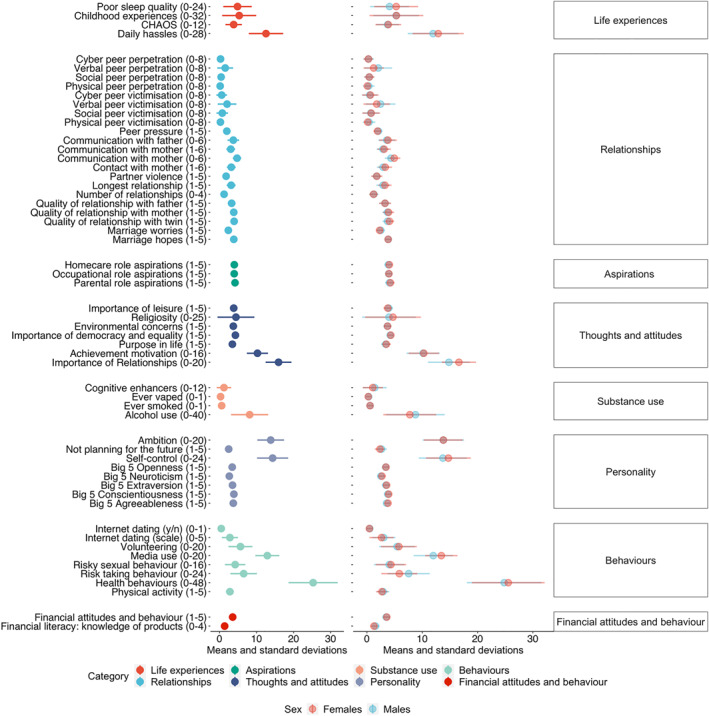
Descriptive statistics (means and standard deviations) for the whole sample (first column of results) and separately for males and females (second column, with minimum and maximum score for each scale in parentheses). For subsequent analyses, the data were corrected for mean age and sex differences, as described in Methods

Means and standard deviations were calculated for the 57 EA traits for the whole sample, for males and females separately (Figure 2), and for five sex and zygosity groups: monozygotic (MZ) males, dizygotic (DZ) males, MZ females, DZ females and DZ opposite‐sex twin pairs (Table S2). ANOVA results confirm the visual near‐identity in the graph, by indicating that sex and zygosity together explain only 2% of the variance on average for psychological traits (Table S2). These results are based on one twin randomly selected from each pair so that the data points are independent. We repeated these analyses with the other twin from each pair selected and results were highly similar (see Tables S4 and S5).

### Wellbeing, adverse mental and physical health composite scores

As indicated in Figure 1B, the variance explained by the first principal components of the functional outcomes was 43% for wellbeing, 40% for adverse mental health, and 37% for adverse physical health. All measures loaded on their respective first PC > 0.43. The first PC scores for wellbeing, adverse mental health and adverse physical health were saved for subsequent analyses. We did not include the Conner's impulsivity and inattention subscales in this final mental health composite because these loaded <0.4 on the first PC and removing the Conner's subscales increased the variance explained by the 1st PC from 34% to 41%. Additionally, we included only the lifetime self‐harm item, not the item about self‐harm in the last year because the two self‐harm items correlated highly (*r* = 0.55, *p* < 0.001, *N* = 3887). We selected the lifetime item to maximise power (lifetime *N* = 4727 vs. last year *N* = 3887).

### Phenotypic analyses

**FIGURE 3:**
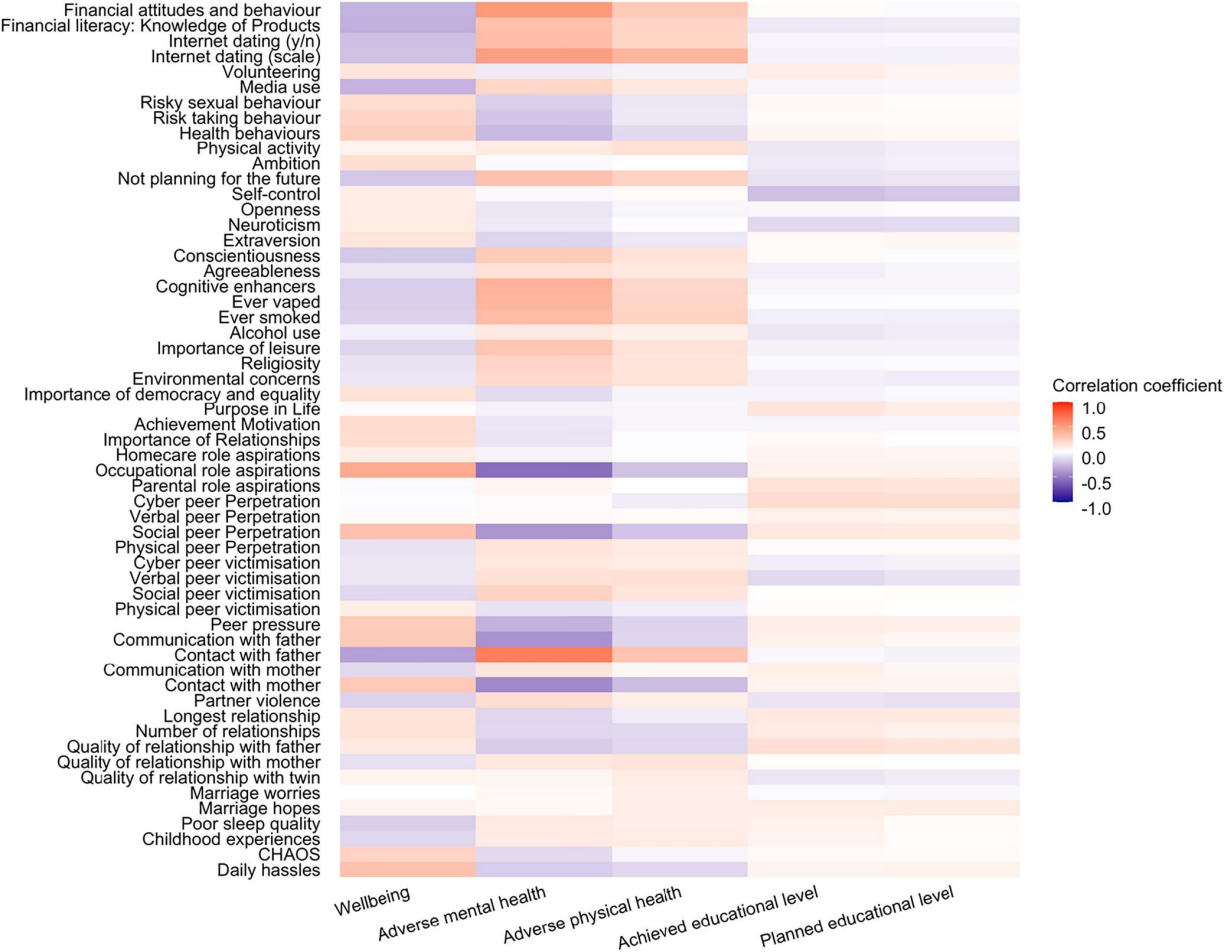
Phenotypic correlations between emerging adulthood (EA) traits and key outcomes (indicated by the total length of the bar). Proportion of each correlation explained by additive genetic (*A*), shared environmental (*C*) and non‐shared environmental factors are indicated by the red, dark blue and light blue bars, respectively

Figure 3 shows Pearson correlations between the EA traits and the composite scores of adverse physical health, adverse mental health, wellbeing and educational outcomes as indicated by the total length of the bars (see Table S6 for correlation coefficients with 95% CI). These phenotypic correlations were mostly significant and moderate (the average correlation was 0.16 for wellbeing, 0.20 for adverse mental health, 0.12 for adverse physical health, and education 0.06). For wellbeing and adverse mental and adverse physical health, the strongest correlations emerged for life experience traits such as poor sleep quality and daily hassles, as well as for purpose in life, importance of leisure and neuroticism. Correlations were similar when calculated separately for males and females (see Figure S2A,B). We also calculated these correlations after excluding opposite‐sex DZ pairs from the analyses and the results are highly similar (see Figure S2C).

**FIGURE 4:**
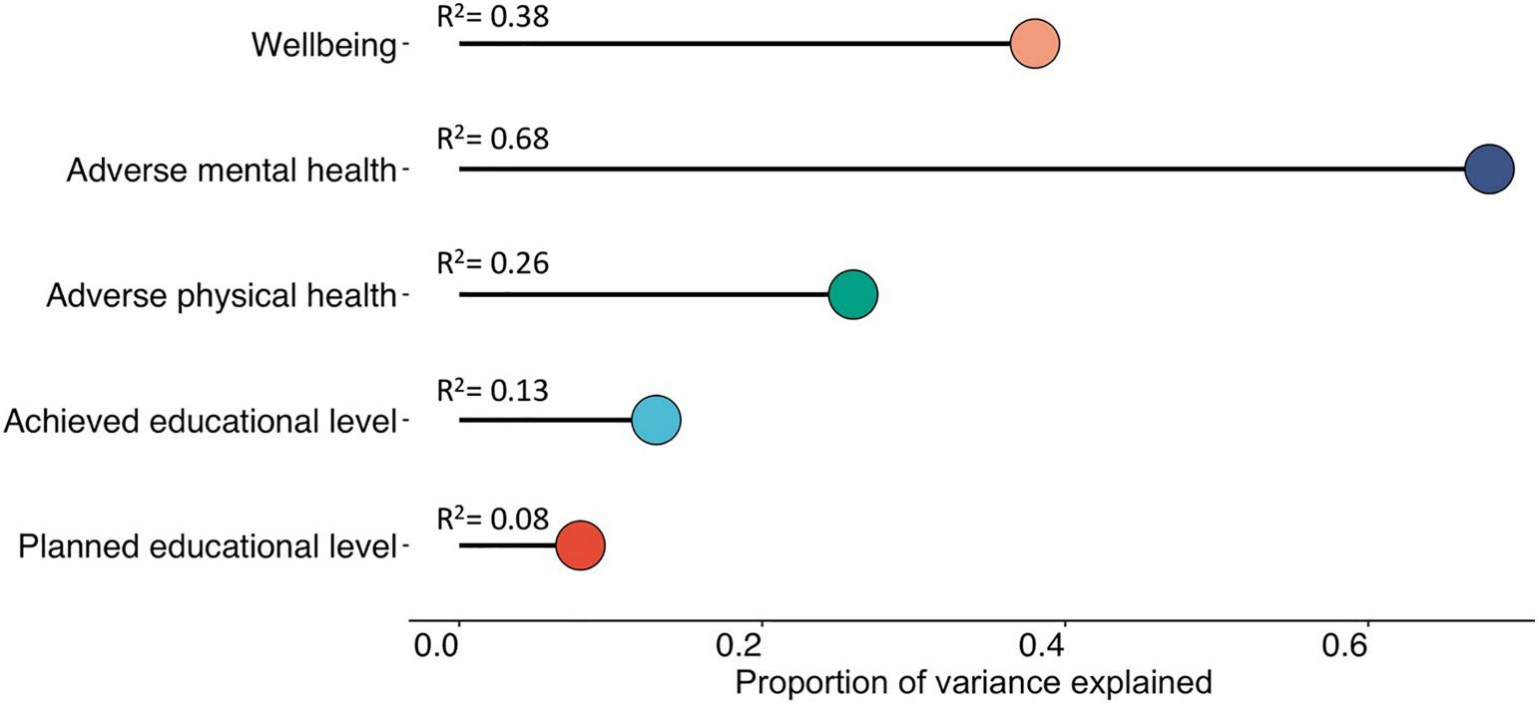
Summary of multiple regression analyses: variance explained (adjusted *R* ^2^) in composite scores of adverse physical health, adverse mental health, wellbeing and educational attainment psychological traits

Figure 4 summarises the results in Figure 3 using multiple regression to predict adverse physical and adverse mental health, wellbeing and achieved and planned educational level. Together, the emerging adulthood traits on average account for 31% (range = 8%–68%) of the variance in the outcomes. The emerging adulthood traits are most strongly associated with adverse mental health, accounting for 68% of the variance. They were least strongly associated with achieved (13%) and planned (8%) educational level. Full results of the multiple regression analyses, including results separately for males and females, are presented in Table S7.

### Genetic analyses

#### Univariate twin analyses

**FIGURE 5:**
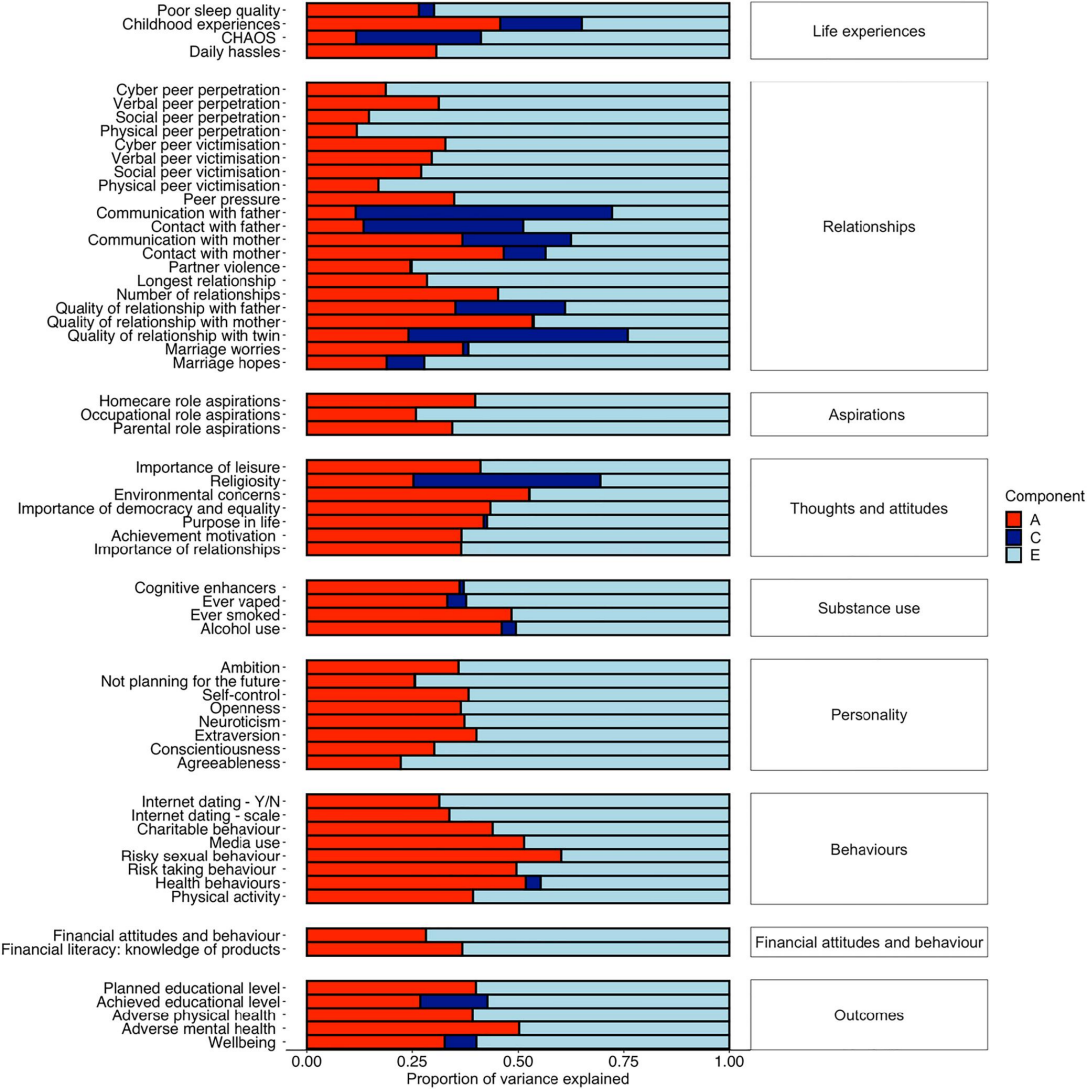
Univariate twin analyses of additive genetic (*A*), shared environmental (*C*), and non‐shared environmental (*E*) components of variance for emerging adulthood (EA) traits

All EA traits as well as functional outcomes showed significant heritability as presented in Figure 5. All twin correlations are presented in Figure S3 and are presented in Table S8 with 95% confidence intervals. Full ACE model‐fitting results, including the 95% confidence intervals, are shown in Table S9 and model‐fit statistics in Table S10. While for some variables the AE model showed better fit than the full ACE model, and some twin correlations indicated the presence of non‐additive genetic variance, ACE model results are shown for completeness and to facilitate comparisons between variables.

Across EA traits and functional outcomes, heritability (*A*) was modest on average (mean 34%, range 12% ‐ 60%). The most heritable trait in emerging adulthood is risky sexual behaviour (0.60; 95% CI: 0.52–0.63). Other traits with substantial heritability include general risk‐taking behaviour (0.50; 95% CI: 0.42–0.53), media use (0.51; 95% CI: 0.48–0.55), health behaviours (0.52; 95% CI: 0.41–0.58), environmental concerns (0.53; 95% CI: 0.43–0.56) and quality of relationship with mother (0.53; 95% CI: 0.43–0.57). These heritabilities are higher than those for the Big Five personality traits, which range from 0.22 (95% CI: 0.18–0.27) to 0.40 (95% CI:.36–0.44). For the outcome variables, the adverse mental health composite is more heritable (0.50, 95% CI: 0.42–0.54) than the adverse physical health composite (0.39, 95% CI: 0.27–0.43).

Shared environmental effects (C) were modest on average (mean 5%, range 0%–61%), but for a few variables substantial effects were observed. The greatest shared environmental effect was found for communication with father (0.61; 95% CI: 0.53–0.68). Substantial shared environmental influence was also found for contact with father (0.38; 95% CI: 0.24–0.50) and quality of relationship with father (0.26; 95% CI: 0.19–0.33). Two other traits showing substantial shared environmental effects were relationship with twin (0.52; 95% CI: 0.47–0.56) and religiosity (0.44; 95% CI: 0.39–0.49).

The majority of the individual differences were explained by non‐shared environment (*E*) factors that accounted for 59% of the variance on average (E range 24%–88%). The largest non‐shared environmental effects were observed for physical and social peer perpetration (0.88; 95% CI 0.83–0.93 and 0.85; 95% CI 0.80–0.91). Importantly, this component of variance also includes measurement error.

These univariate results were similar when computed separately for males and females (Figure S4A) and when DZ opposite‐sex twin pairs were excluded (Figure S4B).

#### Sex‐limitation analyses

A few qualitative and quantitative sex differences emerged, for example, in childhood experiences, quality of relationship with mother and physical victimisation. Full model‐fit statistics with nested models are presented in Table S11; ACE estimates for males and females are presented in Figure S4A and estimates with 95% confidence intervals in Table S8. Two of the largest sex differences in heritability were found for chaos and marriage hopes. For chaos, heritability was 0.29 (95% CI: 0.10–0.47) for males and 0.03 (95% CI: 0.00–0.15) for females; for marriage hopes, heritability was 0.00 (95% CI: 0.00–0.21) for males and 0.25 (95% CI: 0.11–0.39) for females. However, as indicated by the overlapping confidence intervals for these estimates, limited power warrants caution in interpreting these results; they are presented here for completeness. As an additional sensitivity analysis, because we observed significant quantitative sex differences in the sex‐limitation model‐fitting analyses, we also calculated all estimates when excluding opposite‐sex DZ twin pairs from analyses (presented in Supporting Information S1) and the results remained highly similar.

#### Bivariate twin analyses

As mentioned earlier, Figure 3 showed the phenotypic correlations between EA traits and adverse physical health, adverse mental health, wellbeing and education. Figure 6B presents results for bivariate genetic analyses, which estimate the extent to which the phenotypic correlations between emerging adulthood traits and functional outcomes are mediated by genetic and environmental factors. The full results with 95% confidence intervals are presented in Table S12.

FIGURE 6(A) Genetic correlations between EA traits and outcomes derived from bivariate twin analyses; (B) phenotypic correlations between EA traits and key outcomes (indicated by the total length of the bar). Proportion of each correlation explained by additive genetic (*A*), shared environmental (*C*) and non‐shared environmental factors are indicated by the red, dark blue and light blue bars, respectively

**Figure:**
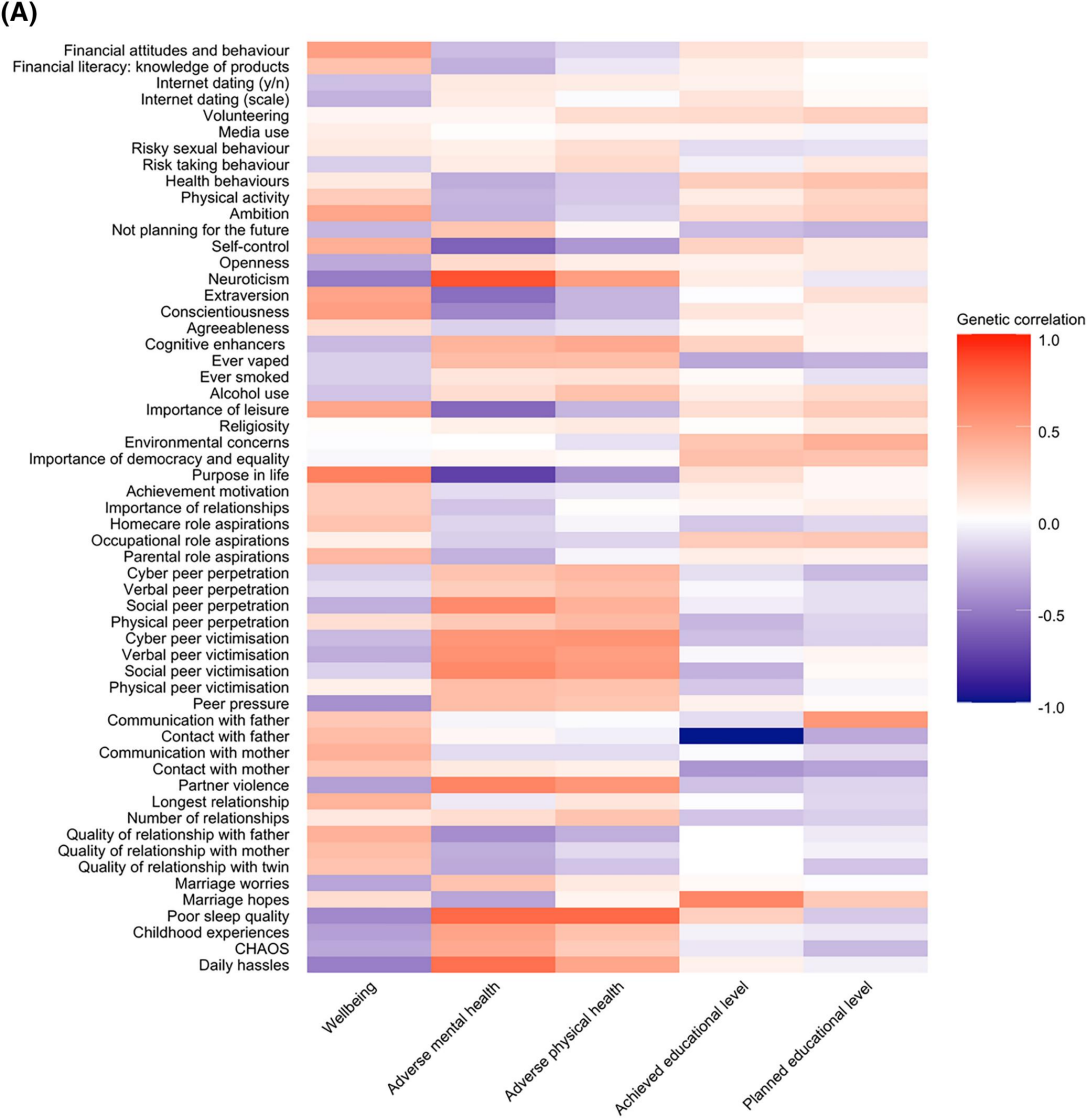

**Figure:**
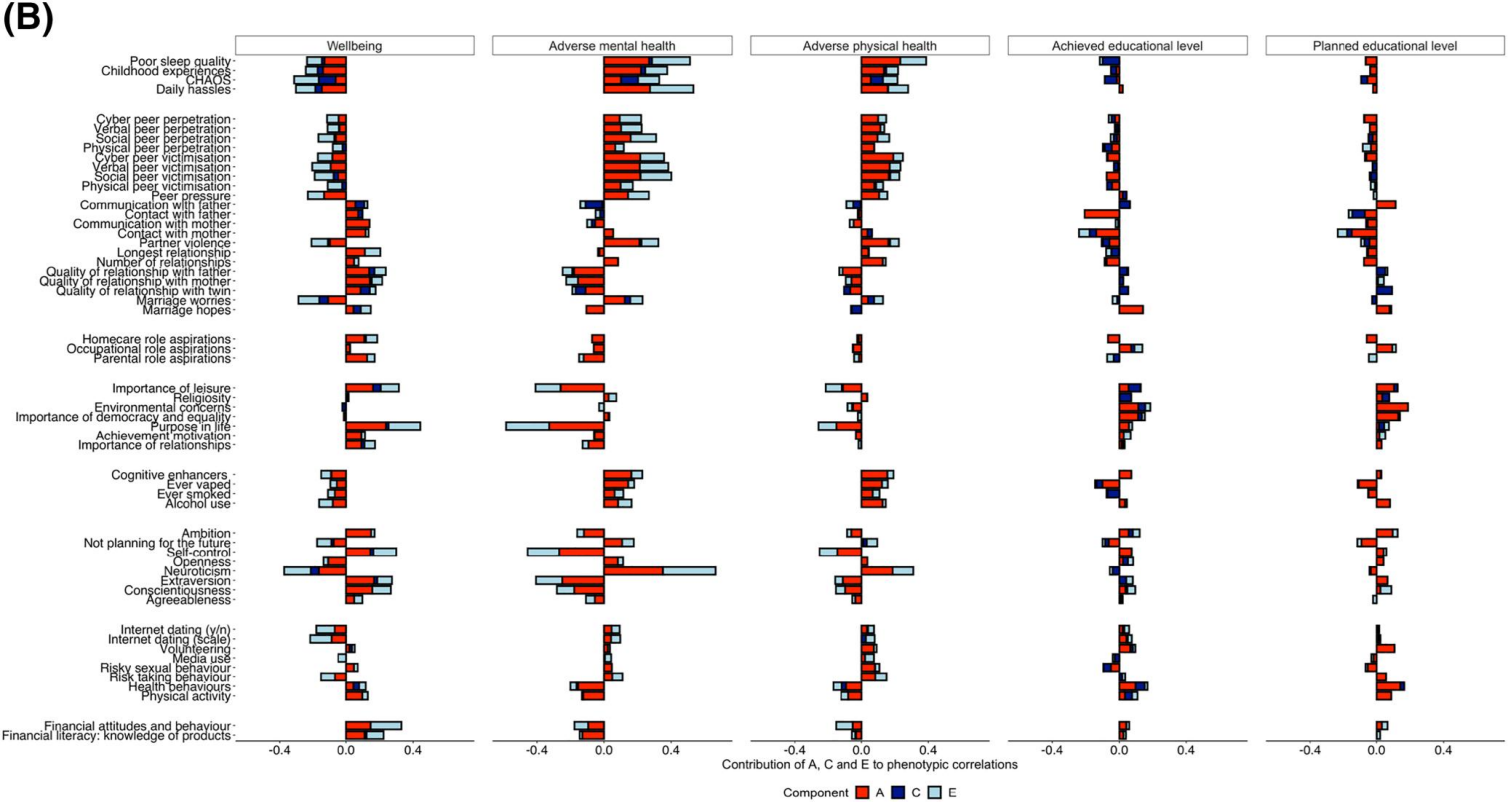

On average, more than 50% of these phenotypic correlations were mediated by genetic factors, 60% for mental health, 56% for physical health, 50% for wellbeing and 52% for the two education outcomes. For example, the first row of Figure 3, shows that poor sleep quality is negatively correlated with wellbeing (*r* = −0.23) and positively correlated with adverse mental health (0.50) and adverse physical health (0.38). These three correlations are largely mediated genetically—56%, 54% and 60%, respectively. The rest of these correlations are mediated by nonshared environmental factors. For all of the substantial phenotypic correlations between EA traits and outcomes, genetic factors account for the majority of the correlations. An equally important finding is that a large proportion of the correlations between EA traits and functional outcomes is explained by non‐shared environmental factors. For example, daily hassles correlated with wellbeing (*r* = −0.31), these are almost in equal parts mediated by genetic and non‐shared environmental factors (48% and 39%, respectively). Shared environment does not significantly contribute to any of these correlations.

Genetic contributions to the phenotypic correlations are based on the genetic correlation weighted by the heritability of the two traits. The genetic correlation estimates the extent to which the same genetic variants influence the two traits, regardless of their heritabilities. Figure 6 presents the genetic correlations between the EA traits and the outcome variables. Table S13 shows the estimates with 95% confidence intervals. The average genetic correlation was 0.23 ignoring the sign of the correlation. Because all EA traits are moderately heritable, the pattern of results for genetic correlations in Figure 6 corresponds to the results in Figure 3 showing the genetic contribution to the phenotypic correlations. For example, the genetic correlations between poor sleep quality and the outcome variables are −0.45 for wellbeing, 0.74 for adverse mental health, and 0.74 for adverse physical health, all of which are statistically significant. Most of the other highest genetic correlations between EA traits and outcomes were observed for adverse mental health: daily hassles (0.71), partner violence (0.62), social peer victimisation (0.60), Big 5 neuroticism (0.83), and self‐control (−0.61). It should be noted that when the phenotypic correlation is negligible, the genetic correlation can be substantial but signifies little. For example, marriage hopes yields a genetic correlation of 0.61 with educational attainment but the phenotypic correlation is only 0.10 and it yields modest genetic correlations with other outcomes. These results are highly similar for males and females (Figure S5A,B) and also similar when excluding opposite‐sex twin pairs (Figure S5C).

The shared environmental correlation (rC) and non‐shared environmental correlation (rE) can also be estimated. The shared environmental correlations are illustrated in Figure S6 and non‐shared environmental correlations in Figure S7. Table S13 presents these estimates with 95% confidence intervals. None of the shared environmental correlations are statistically significant. Because shared environment contributes so little to the phenotypic correlations between EA traits and outcomes, the shared environmental correlations can be large, but the 95% confidence intervals indicate that these estimates are not significant.

#### Univariate GREML‐GCTA

Although the sample size with genotyped DNA limited power to detect SNP heritability, for completeness we present these estimates in Table S14, which indicates the sample sizes for each measure. The mean SNP heritability was 10%, ranging up to 26%, as illustrated in Figure S8. In addition, we conducted bivariate GREML analysis, again for completeness (Table S15). However, little confidence can be placed in these results due to limited power.

#### Sensitivity analyses

Univariate twin and GREML analyses reported above were repeated with van der Waerden transformed variables. The results of these analyses are presented in the Supporting Information S1 (Figure S9 for univariate twin analyses; Figure S10 for univariate GREML analyses). Results do not differ with and without transformation.

## DISCUSSION

Societal changes have stretched the transition from adolescence to adulthood, which has created a new developmental period called emerging adulthood. The prolonged uncertainties about identity, relationships and occupations can create stress but they also offer flexibility and unprecedented opportunities for young people to find their adult selves. These opportunities are amplified by the Internet and social media for this first generation of digital natives.

Here we reported the results of the first systematic investigation of genetic and environmental influences on 57 psychological traits covering major issues in emerging adulthood such as aspirations, thoughts and attitudes, relationships and personality. All 57 traits showed significant genetic influence, with an average heritability of 34% while the majority of individual differences (59% on average) were explained by non‐shared environmental influences. These diverse traits were associated with mental health (average correlation 0.20), wellbeing (0.16), physical health (0.12) and educational attainment (0.06). Shared genetic factors explained the majority of these correlations (∼50%). Together, these emerging adulthood traits explained on average 30% of variance in the outcomes (range = 8%–69%), suggesting that these traits relate to the outcomes additively.

As mentioned in the Introduction, the ever‐expanding menu of idiosyncratic experiences for self‐exploration during emerging adulthood suggests that environmental influence is likely to be critical for emerging adulthood traits. The present results from this first systematic analysis of emerging adulthood traits in a genetically sensitive design are consistent with this hypothesis in that the environment is the major source of individual differences in EA traits. Moreover, more than 90% of the environmental influence is due to non‐shared environment, which includes measurement error. Non‐shared environment could reflect idiosyncratic experiences in the chaotic world of emerging adulthood, experiences not likely to be shared by siblings. This non‐shared environment is especially relevant when examining the differences between MZ twin pairs, who share their genes and home environment, are the same age and same sex. Shared environmental influences account for only 5% of the variance of the 57 EA traits on average. In other words, most environmental effects on EA traits are not shared by siblings, even twins, who grow up in the same family. As siblings leave the family home in early adulthood, it seems likely that they each make their own independent way in the world, having trait‐relevant experiences not shared with their sibling. Part of our ongoing research is to investigate the environmental experiences that account for individual differences in emerging adulthood using the longitudinal data collected in TEDS.

Another possibility is that the heterogeneity of experience in early adulthood provides an opportunity for genetic potential to be realised as individuals select, modify and create environments correlated with their genetic propensities. This is gene–environment correlation, which contributes to heritability. In support of this hypothesis, we found significant genetic influence for all 57 EA traits, many of which have not been investigated previously in emerging adulthood. On average, genetic influences accounted for 34% of the variance. Moreover, the most highly heritable traits in our study are traits that are especially relevant to emerging adulthood: risky sexual behaviour (60% heritability), general risk‐taking behaviour (50%), media use (51%) and concerns about the environment (53%). An additional possibility is gene–environment interactions, in which individuals' reactions to environmental influences depend on genetic factors.

Although our results can be viewed as providing support for both hypotheses, it is also possible to interpret the results as support for neither hypothesis because both moderate genetic influence and substantial non‐shared environmental influence are found for nearly all traits throughout development. These findings are consistent with meta‐anayses of over 50 years of twin studies showing that the majority of the twin resemblance is due to genetic factors, with the average heritability of all traits around 50%, and the rest of the variance is explained by non‐shared environmental factors, not shared by twins growing up in the same family (Polderman et al., 2015). Two of the 10 most replicated findings from behavioural genetics are the findings that all psychological traits show significant and substantial genetic influence and most environmental effects are not shared by children growing up in the same family (Plomin et al., 2016). Nonetheless, at the least it is useful to confirm these findings in emerging adulthood and especially for the many traits that have not been investigated previously at this age.

Moreover, it is possible that some EA traits show greater genetic or non‐shared environmental influence in emerging adulthood as compared to earlier ages. Because many of these traits have not been studied previously in genetically sensitive designs, precise comparisons are not possible. One exception in TEDS is personality—the same measure of the Big Five personality traits was administered at age 16 and ages 21–25. The heritability of the Big Five personality traits was around 30% at age 16 (Rimfeld et al., 2016) and 33% at age 21. This slight increase in heritability is not statistically significant and none of the five individual traits yielded significantly greater heritability at age 21 than at age 16. However, personality is highly stable across development (Turkheimer et al., 2014) and may be the least likely domain to show developmental changes in heritability.

TEDS also has some relevant data from a study of the psychological effects of COVID‐19 (Rimfeld et al., 2016). In a subsample of TEDS, nine EA traits from the present study were assessed using the same measures 2 years later. For these EA traits, the average heritability was 34% at both ages. However, this study also found that change across the 2‐year period was significantly heritable (15% on average), suggesting that genetic influences change even during this 2‐year period in the early years of emerging adulthood. It remains to be seen whether the heritability of EA traits increases as the sample fully emerges into what has been called established adulthood (Mehta et al., 2020). The importance of non‐shared environment in emerging adulthood is clear: it accounted for 82% of the variance on average for change across the 2‐year period.

Our finding of high heritability (50%–60%) for traits most relevant to emerging adulthood and the evidence for genetic change during emerging adulthood leads us to predict that the heritability of EA traits will increase as we follow the twins into established adulthood. In any case, it seems that the turmoil of emerging adulthood does not shuffle the deck of individual differences. One firm conclusion is that heritability is not swamped by the environmental heterogeneity of emerging adulthood.

Our results also indicate that EA traits are associated with wellbeing, adverse mental and adverse physical health and educational outcomes. Although the associations were modest to moderate for individual EA traits and outcomes, the EA traits acting additively and together accounted for 31% of the variance in the outcomes on average. We show that adverse mental and physical health, wellbeing and educational attainment reflect a package of heritable EA traits. These findings also indicate that when studying individual differences in health, wellbeing or socioeconomic phenotypes in emerging adulthood, diverse traits should be considered such as life goals and aspirations and relationships. The additional implication of the results points to genetic confounding when studying the links between these functional outcomes and diverse psychological traits, as our results from bivariate genetic analyses show that these correlations are mediated mostly by genetic factors. Future research should use genetically sensitive designs when studying the correlates of functional life outcomes. However, these findings underscore the adage that correlation does not imply causation. For example, perceived daily hassles correlates negatively with wellbeing and positively with adverse mental and adverse physical health, but it cannot be assumed that daily hassles cause wellbeing or health.

Compared to health and wellbeing outcomes, we explained much less variance in educational outcomes (or hopes/aspirations for educational outcomes), compared to our previous reports in earlier childhood or even late adolescence (Krapohl et al., 2014). This can be explained in terms of the precision of the phenotype. Here we use achieved educational outcome, which is basically years of education, while previously we have used much finer‐grained measures of educational achievement, such as teacher‐graded performance or standardised examination results. However, similar to our previous report (Krapohl et al., 2014), we show that educational achievement and attainment can be partially explained by a range of behavioural and psychological traits, such as personality, poor sleep quality or risk‐taking, all of which are also substantially heritable.

The limitations of this study include the usual limitations of twin design, which are described in detail elsewhere (Knopik et al., 2017; Rijsdijk & Sham, 2002). In addition, attrition and selection bias might have influenced the results. It is well documented that participants in most studies tend to be slightly healthier and more educated than the general population, and therefore, the associations observed between study variables might be biased by collider effects (Munafò et al., 2018). However, the TEDS twins in their early twenties remain reasonably representative of their birth cohort in terms of ethnicity and socioeconomic status (Rimfeld et al., 2019). An additional limitation is missing data and here we only used twins having all available data points. Missingness was minimal per data collection phase (e.g., it was less than 4% for the wellbeing composite) but was larger when both phase 1 and phase 2 data were used (e.g., around 18% for physical health). The representativeness of the sample, however, was very similar for phase 1 and phase 2 data collection. We repeated the analyses using mean scores of wellbeing, mental and physical health as a sensitivity analysis, allowing for missing data, and the patterns of associations and the aetiology of these composite measures remained highly similar. A conceptual limitation is that our EA traits were assessed at the same time as adverse mental and adverse physical health, wellbeing and education. Although we view the latter variables as outcomes, we acknowledge that longitudinal assessment of these outcomes is needed to investigate the extent to which EA traits predict them, which is part of our research plan for TEDS. It should also be acknowledged that the adverse physical and mental health, as well as wellbeing, were composite measures of many diverse constructs, so further finer‐grained research is warranted. As with every study, these results should be replicated in other studies using diverse samples. The TEDS study is predominantly white British, which is representative for this birth cohort, and might not replicate in other ancestry groups. An additional consideration is that the developmental stage of emerging adulthood might not appear in all countries, and where it does occur, cultural differences might affect emerging adulthood.

We conclude that non‐shared environment is a major source of individual variation in emerging adulthood, however, the environmental uncertainties of emerging adulthood in the 21st century do not diminish the importance of genetics. As adolescents travel down long and winding roads to adulthood, their trip is substantially influenced by genetic proclivities that nudge them down different paths that lead to different destinations.

## CONFLICT OF INTEREST

Thalia C. Eley & Kaili Rimfeld are members of the Editorial Advisory Board for JCPP *Advances*. The remaining authors have declared that they have no competing or potential conflicts of interest. [Corrections made on 22 June 2022, after first online publication: This Conflict of Interest statement has been updated in this version.]

## ETHICS STATEMENT

Ethical approval was provided by the King's College London Research Ethics Committee (reference number: PNM/09/10‐104) and informed written consent was received from all participants. No exclusions were applied.

## Supporting information

Supporting Information S1

## Data Availability

Data for this study came from the Twins Early Development Study (TEDS). Researchers can apply for access to the data: http://www.teds.ac.uk/researchers/teds-data-access-policy.

## References

[jcv212053-bib-0001] Anglim, J. , Horwood, S. , Smillie, L. D. , Marrero, R. J. , & Wood, J. K. (2020). Predicting psychological and subjective well‐being from personality: A meta‐analysis. Psychological Bulletin, 146(4), 279–323. 10.1037/bul0000226 31944795

[jcv212053-bib-0002] Arnett, J. J. (2004). Emerging adulthood: The winding road from the late teens through the twenties. In Emerging adulthood: The winding road from the late teens through the twenties. 10.1093/acprof:oso/9780195309379.001.0001

[jcv212053-bib-0003] Arnett, J. J. (2015). Emerging adulthood: The winding road from the late teens through the twenties. Oxford University Press.

[jcv212053-bib-0004] Arnett, J. J. (2016). Does emerging adulthood theory apply across social classes? National data on a persistent question. Emerging Adulthood, 4(4), 227–235. 10.1177/2167696815613000

[jcv212053-bib-0005] Bartels, M. (2015). Genetics of wellbeing and its components satisfaction with life, happiness, and quality of life: A review and meta‐analysis of heritability studies. Calcified Tissue International, 45, 137–156. 10.1007/s10519-015-9713-y PMC4346667 25715755

[jcv212053-bib-0006] Baselmans, B. M. L. , van de Weijer, M. P. , Abdellaoui, A. , Vink, J. M. , Hottenga, J. J. , Willemsen, G. , Nivard, M. G. , de Geus, E. J. C. , Boomsma, D. I. , & Bartels, M. (2019). A genetic investigation of the well‐being spectrum. Behavior Genetics, 49(3), 286–297. 10.1007/s10519-019-09951- 30810878 PMC6497622

[jcv212053-bib-0007] Bergen, S. E. , Gardner, C. O. , Kendler, K. S. , Estabrook, R. , Kenny, S. , Bates, T. , Mehta, P. , & Fox, J. (2007). Age‐related changes in heritability of behavioral phenotypes over adolescence and young adulthood: A meta‐analysis. Twin Research and Human Genetics. 10.1375/twin.10.3.423 17564500

[jcv212053-bib-0008] Boker, S. , Neale, M. , Maes, H. , Wilde, M. , Spiegel, M. , Brick, T. , & Fox, J. (2011). OpenMx: An open source extended structural equation modeling framework. Psychometrika, 76, 306–317. 10.1007/s11336-010-9200-6 23258944 PMC3525063

[jcv212053-bib-0009] Bonnie, R. J. , Stroud, C. , & Breiner, H. (2015). Investing in the health and well‐being of young adults. National Academies Press. 10.17226/18869 25855847

[jcv212053-bib-0010] Briley, D. a. , & Tucker‐Drob, E. M. (2013). Explaining the increasing heritability of cognitive ability across development: A meta‐analysis of longitudinal twin and adoption studies. Psychological Science, 24, 1704–1713. 10.1177/0956797613478618 23818655 PMC3954471

[jcv212053-bib-0011] Cutler, D. M. , & Lleras‐Muney, A. (2012). Education and health: Insights from international comparisons (Working Papers No. 17738). NBER.

[jcv212053-bib-0012] Harris, K. M. , Gordon‐Larsen, P. , Chantala, K. , & Udry, J. R. (2006). Longitudinal trends in race/ethnic disparities in leading health indicators from adolescence to young adulthood. Archives of Pediatrics and Adolescent Medicine, 28(1), 525–533. 10.1001/archpedi.160.1.74 16389215

[jcv212053-bib-0013] Hatemi, P. K. , & McDermott, R. (2012). The genetics of politics: Discovery, challenges, and progress. Trends in Genetics, 118, 762–776. 10.1016/j.tig.2012.07.004 22951140

[jcv212053-bib-0014] Hufer, A. , Kornadt, A. E. , Kandler, C. , & Riemann, R. (2020). Genetic and environmental variation in political orientation in adolescence and early adulthood: A nuclear twin family analysis. Journal of Personality and Social Psychology. 10.1037/pspp0000258 31259578

[jcv212053-bib-0015] Johnson, M. K. , Crosnoe, R. , & Elder, G. H. (2011). Insights on adolescence from a life course perspective. Journal of Research on Adolescence, 21(1), 273–280. 10.1111/j.1532-7795.2010.00728.x 21483644 PMC3072576

[jcv212053-bib-0016] Kessler, R. C. , Berglund, P. , Demler, O. , Jin, R. , Merikangas, K. R. , & Walters, E. E. (2005). Lifetime prevalence and age‐of‐onset distributions of DSM‐IV disorders in the national comorbidity survey replication. Archives of General Psychiatry, 62(6), 593–602. 10.1001/archpsyc.62.6.593 15939837

[jcv212053-bib-0017] Knopik, V. S. , Neiderhiser, J. M. , DeFries, J. C. , & Plomin, R. (2017). Behavioral genetics (7th ed.). Worth Publishers.

[jcv212053-bib-0018] Koenig, L. B. , McGue, M. , & Iacono, W. G. (2008). Stability and change in religiousness during emerging adulthood. Developmental Psychology, 44(2), 532. 10.1037/0012-1649.44.2.532 18331142

[jcv212053-bib-0019] Kornadt, A. E. , Hufer, A. , Kandler, C. , Riemann, R. , Harlaar, N. , Kovas, Y. , Dale, P. S. , & Plomin, R. On the genetic and environmental sources of social and political participation in adolescence and early adulthood. PLoS One, 13(8), e0202518. 10.1371/journal.pone.0202518 PMC6108469 30142159

[jcv212053-bib-0020] Krapohl, E. , Rimfeld, K. , Shakeshaft, N. G. , Trzaskowski, M. , McMillan, A. , Pingault, J.‐B. , & Plomin, R. (2014). The high heritability of educational achievement reflects many genetically influenced traits, not just intelligence. Proceedings of the National Academy of Sciences of the United States of America, 111(42), 15273–15278. 10.1073/pnas.1408777111 25288728 PMC4210287

[jcv212053-bib-0021] Lee, S. H. , Yang, J. , Goddard, M. E. , Visscher, P. M. , & Wray, N. R. (2012). Estimation of pleiotropy between complex diseases using single‐nucleotide polymorphism‐derived genomic relationships and restricted maximum likelihood. Bioinformatics, 28, 2540–2542. 10.1093/bioinformatics/bts474 22843982 PMC3463125

[jcv212053-bib-0022] Lehmann, E. (1975). Nonparametric statistical methods based on ranks. Holden‐Day.

[jcv212053-bib-0023] McGue, M. , & Bouchard, T. J. (1984). Adjustment of twin data for the effects of age and sex. Behavior Genetics, 14, 325–343. 10.1007/BF01080045 6542356

[jcv212053-bib-0024] Medland, S. E. (2004). Alternate parameterization for scalar and non‐scalar sex‐limitation models in Mx. Twin Research, 7(3), 299–444. 10.1375/twin.7.3.299 15193175

[jcv212053-bib-0025] Mehta, C. M. , Arnett, J. J. , Palmer, C. G. , & Nelson, L. J. (2020). Established adulthood: A new conception of ages 30 to 45. American Psychologist, 75(4), 431. 10.1037/amp0000600 32378940

[jcv212053-bib-0026] Munafò, M. R. , Tilling, K. , Taylor, A. E. , Evans, D. M. , & Smith, G. D. (2018). Collider scope: When selection bias can substantially influence observed associations. International Journal of Epidemiology, 47(1), 226–235. 10.1093/ije/dyx206 29040562 PMC5837306

[jcv212053-bib-0027] Neale, M. C. , Boker, S. M. , Bergeman, C. S. , & Maes, H. H. (2005). The utility of genetically informative data in the study of development. Methodological Issues in Aging Research, 59, P117–P122. 10.4324/9781315820989-14

[jcv212053-bib-0028] Ong, A. D. , & Bergeman, C. S. (2004). The complexity of emotions in later life. Journals of Gerontology Series B: Psychological Sciences and Social Sciences. 10.1093/geronb/59.3.P117 15118014

[jcv212053-bib-0029] Ong, A. D. , Bergeman, C. S. , Bisconti, T. L. , & Wallace, K. A. (2006). Psychological resilience, positive emotions, and successful adaptation to stress in later life. Journal of Personality and Social Psychology, 91(4), 730. 10.1037/0022-3514.91.4.730 17014296

[jcv212053-bib-0030] Pain, O. , Dudbridge, F. , & Ronald, A. (2018). Are your covariates under control? How normalization can re‐introduce covariate effects. European Journal of Human Genetics, 26(8), 1194–1201. 10.1038/s41431-018-0159-6 29706643 PMC6057994

[jcv212053-bib-0031] Park, M. J. , Paul Mulye, T. , Adams, S. H. , Brindis, C. D. , & Irwin, C. E. (2006). The health status of young adults in the United States. Journal of Adolescent Health, 39(3), 305–317. 10.1016/j.jadohealth.2006.04.017 16919791

[jcv212053-bib-0032] Plomin, R. , DeFries, J. C. , Knopik, V. S. , & Neiderhiser, J. M. (2016). Top 10 replicated findings from behavioral genetics. Perspectives on Psychological Science, 11(1), 3–23. 10.1177/1745691615617439 26817721 PMC4739500

[jcv212053-bib-0033] Polderman, T. J. C. , Benyamin, B. , de Leeuw, C. A. , Sullivan, P. F. , van Bochoven, A. , Visscher, P. M. , & Posthuma, D. (2015). Meta‐analysis of the heritability of human traits based on fifty years of twin studies. Nature Genetics, 47(7), 702–709. 10.1038/ng.3285 25985137

[jcv212053-bib-0034] Price, T. S. , Freeman, B. , Craig, I. , Petrill, S. A. , Ebersole, L. , & Plomin, R. (2000). Infant zygosity can be assigned by parental report questionnaire data. Twin Research, 3, 129–133. 10.1375/twin.3.3.129 11035484

[jcv212053-bib-0035] R Core Team . (2020). R: A language and environment for statistical computing. R Foundation for statistical computing. https://www.R‐project.org/

[jcv212053-bib-0036] Rijsdijk, F. V. , & Sham, P. C. , Eley T. C. , Plomin R. (2002). Analytic approaches to twin data using structural equation models. Briefings in Bioinformatics, 3(2), 119–133. 10.1093/bib/3.2.119 12139432

[jcv212053-bib-0037] Rimfeld, K. , Kovas, Y. , Dale, P. S. , Plomin, R. , Shakeshaft, N. G. , Schofield, K. L. , Pingault, J.‐B. , Stringaris, A. , Stumm, S. v. , & Plomin, R. (2016). True grit and genetics: Predicting academic achievement from personality. Journal of Personality and Social Psychology, 111(5), 780–789. 10.1037/pspp0000089 26867111 PMC4981570

[jcv212053-bib-0039] Rimfeld, K. , Malanchini, M. , Arathimos, R. , Gidziela, A. , Pain, O. , McMillan, A. , Ogden, R. , Webster, L. , Packer, A. E. , Shakeshaft, N. G. , & Schofield, K. L. (2020). The consequences of a year of the COVID‐19 pandemic for the mental health of young adult twins in England and Wale. medRxiv, 1–28. 10.1192/bjo.2022.506 PMC9304950 35860899

[jcv212053-bib-0038] Rimfeld, K. , Malanchini, M. , Spargo, T. , Spickernell, G. , Selzam, S. , McMillan, A. , & Plomin, R. (2019). Twins early development study: A genetically sensitive investigation into behavioral and cognitive development from infancy to emerging adulthood. Twin Research and Human Genetics, 22(6), 508–513. 10.1017/thg.2019.56 31544730 PMC7056571

[jcv212053-bib-0040] Selzam, S. , McAdams, T. A. , Coleman, J. R. I. , Carnell, S. , O’Reilly, P. F. , Plomin, R. , & Llewellyn, C. H. (2018). Evidence for gene‐environment correlation in child feeding: Links between common genetic variation for BMI in children and parental feeding practices. PLoS Genetics, 14(11), e1007757. 10.1371/journal.pgen.1007757 30457987 PMC6245504

[jcv212053-bib-0041] Tanner, J. L. (2016). Mental health in emerging adulthood. In The Oxford Handbook of emerging adulthood. Oxford University Press. 10.1093/oxfordhb/9780199795574.013.30

[jcv212053-bib-0042] Turkheimer, E. , Pettersson, E. , & Horn, E. E. (2014). A phenotypic null hypothesis for the genetics of personality. Annual Review of Psychology, 65, 515–540. 10.1146/annurev-psych-113011-143752 24050184

[jcv212053-bib-0043] van de Weijer, M. P. , de Vries, L. P. , & Bartels, M. (2020). Happiness and Wellbeing; the value and findings from genetic studies. PsyArXiv.

[jcv212053-bib-0044] Van Der Waerden, B. L. (1975). On the sources of my book Moderne Algebra. Historia Mathematica, 2, 31–40.

[jcv212053-bib-0045] Veenhoven, R. (2008). Healthy happiness: Effects of happiness on physical health and the consequences for preventive health care. Journal of Happiness Studies, 9(3), 449–469. 10.1007/s10902-006-9042-1

[jcv212053-bib-0046] Wood, D. , Crapnell, T. , Lau, L. , Bennett, A. , Lotstein, D. , Ferris, M. , & Kuo, A. (2018). Emerging adulthood as a critical stage in the life course. In E. M. F. N. Halfon , C. B. Forrest , & R. M. Lerner (Eds.), Handbook of life course health development (pp. 123–143). Springer International Publishing. 31314293

[jcv212053-bib-0047] Yaffe, K. , Vittinghoff, E. , Pletcher, M. J. , Hoang, T. D. , Launer, L. J. , Whitmer, R. , & Sidney, S. (2014). Early adult to midlife cardiovascular risk factors and cognitive function. Circulation, 129(15), 1560–1567. 10.1161/CIRCULATIONAHA.113.004798 24687777 PMC4700881

[jcv212053-bib-0048] Yang, J. , Lee, S. H. , Goddard, M. E. , & Visscher, P. M. (2013). Genome‐wide complex trait analysis (GCTA): Methods, data analyses, and interpretations. Methods in Molecular Biology, 1019, 215–236. 10.1007/978-1-62703-447-0-9 23756893

