# Supplementary material for: The winding roads to adulthood: A twin study
Source: JCPP Adv. 2021 Dec 11;1(4):e12053. doi: 10.1002/jcv2.12053 (PMC10242946; doi:10.1002/jcv2.12053)
Supplement: Supplementary file 1 — Supporting Information S1 [file JCV2-1-e12053-s001.pdf]

Supporting information

The winding roads to adulthood: a twin study

Kaili Rimfeld, Margherita Malancini, Amy E. Packer, Agnieszka Gidziela, Andrea Allegrini, Ziada Ayorech, Emily Smith-Woolley, Andrew McMillan, Rachel Ogden, Philip S. Dale, Thalia C. Eley and Robert Plomin

Contents

Appendix S1 3

Quality control 3

Supplementary Tables 4

Table S1. Descriptive statistics for psychological traits, for the whole and males and females separately. 4

Table S2. Descriptive statistics for psychological traits, for five sex and zygosity groups. 8

Table S3. Summary of measured variables used 11

Table S4. Descriptive statistics for psychological traits, for the whole and males and females separately (repeated with the twin that was not randomly selected from each pair in the main analyses). 21

Table S5. Descriptive statistics for psychological traits, for five sex and zygosity groups (repeated with the twin that was not randomly selected from each pair in the main analyses). 25

Table S6. Phenotypic correlations between psychological traits and composite scores of adverse physical and adverse mental health, wellbeing and educational attainment, for the whole sample (a) and then separately for males (b) and females (c) (95% confidence intervals are in parentheses). 28

Table S7. Summary of multiple regression analyses: variance explained in composite scores of adverse physical and adverse mental health, wellbeing and educational attainment psychological traits, for the whole sample (a) and then separately for males (b) and females (c) (95% confidence intervals are in parentheses). 38

Table S8. Twin intraclass correlations and Falconer ACE estimates, and model fitting results for univariate analyses of additive genetic (A), shared environmental (C), and non-shared environmental (E) components of variance for variables for psychological traits and functional outcomes (95% confidence intervals are in parentheses). (a) for the whole sample; (b) males only; (c) females only; (d) MZ and DZ same sex twin pairs (opposite sex DZ twins excluded). 49

Table S9. Univariate twin analyses presenting additive genetic (A), shared environmental (C), and non-shared environmental (E) components of variance EA traits and functional outcomes (95% confidence intervals are in parentheses). 60

Table S10. Model-fit statistics for the univariate twin analyses 64

Table S11. Model-fit statistics for the sex limitation twin analyses. 75

Table S12. Model fitting results for bivariate analyses of the correlation between psychological traits and functional outcomes explained by genetic (A), shared environmental (C), and non-shared environmental (E) factors (95% confidence intervals are in parentheses). 83

Table S13. Model fitting results for bivariate analyses of genetic correlation (rA), shared environmental correlation (rC), and non-shared environmental correlation (rE) for psychological traits and functional outcomes (95% confidence intervals are in parentheses). 97

Table S14. Univariate GREML estimates 112

Table S15. Bivariate GREML results. 115

Supplementary Figures 118

Figure S1. The Bivariate Cholesky Decomposition. 118

Figure S2. Phenotypic correlations between key outcomes and emerging adulthood variables (indicated by the total length of the bar); bivariate genetic estimates for additive genetic (A), shared environmental (C) and non-shared environmental (E) contributions to these correlations for males (a), females (b) and when excluding DZ opposite sex twin pairs (c). 119

Figure S3. Twin correlations for psychological and behavioural traits and key functional outcomes for the whole sample. 122

Figure S4. Univariate analyses of additive genetic (A), shared environmental (C), and non-shared environmental (E) components of variance for variables for psychological traits and functional outcomes for when calculated for males and females separately (a) and for the whole sample when opposite sex DZ twin pairs were excluded (b). 123

Figure S5. Genetic correlations between psychological and behavioural traits and key functional outcomes for the males (a), females (b) and for the whole sample when opposite sex DZ twin were excluded (c) 125

Figure S6. Shared environmental correlations between psychological and behavioural traits and key functional outcomes (a) for the whole sample; (b) MZ and DZ same sex; (c) males only; (d) females only. 126

Figure S7. Non-shared environmental correlations between psychological and behavioural traits and key functional outcomes (a) for the whole sample; (b) MZ and DZ same sex twin pairs only; (c) males only; (d) females only 127

Figure S8. Univariate GREML analyses; SNP heritabilities for psychological traits and functional outcomes for the whole sample (standard errors represented in error bars; reml-no-constrain option indicated by the point estimates below 0). 128

Figure S9. Univariate twin analyses of additive genetic (A), shared environmental (C), and non-shared environmental (E) components of variance for variables for psychological traits and functional outcomes for the whole sample for untransformed data and when using Van der Waerden transformation 129

Figure S10. Univariate GREML analyses; SNP heritabilities for psychological traits and functional outcomes for the whole sample for untransformed data and when using Van der Waerden transformation (standard errors represented in error bars). 129

Appendix S1

Quality control

Two methods of identifying likely "clickers" are used in the TEDS-21 twin questionnaires:

1. *Quality control item error with uniform responding*. For a measure containing a quality control
2. (QC) item, if a twin's response in the QC item was incorrect *and* the twin gave the same response in at least 3 of the adjacent 4 items within the measure, then the twin was labelled as a clicker and the theme was excluded. This definition could be applied in paper questionnaires as well as in web/app questionnaires. The rule had to be modified in some measures depending on the nature of the questions, etc.

3. *Quality control item error with rapid responding*. For a measure containing a QC item, if a twin's response in the QC item was incorrect *and* the mean time taken per item across the entire theme was in the lowest 20%-ile of the distribution, then the twin was labelled as a clicker and the theme was excluded. This definition could only be applied for twins using web/app, where times could be measured, not for twins using paper. This rule was relaxed in only one theme where different conditions seemed to apply.

Hence, twins responding on paper could be excluded by the first criterion but not the second.

The rules above could not be applied uniformly across all themes in the two questionnaires. Some themes had no measures with QC items
(hence no rule could be applied), some themes had one measure with a QC item, and some themes had more than one measure with a QC item.

The % excluded varies according to exclusion method, measure and theme. Below is an approximation of the % of twins excluded:

- Uniform responding (with QC item error): typically around 1% of twins were excluded per measure, but this varied from .01% to 6% in different measures.

- Rapid responding (with QC item error): typically around 1% of twins were excluded per measure, but this varied from .5% to 3%.

- Overall per theme: typically around 1% of twins were excluded per theme, but this varied from .3% to 5.3% (and of course 0% in themes without any measure containing a QC item).

- Overall exclusion for the entire questionnaire (questionnaire excluded if two or more themes excluded): 1.7% of twins in phase 1,.5% of twins in phase 2.

- Overall % of clickers (twin deemed to be a clicker and hence excluded from at least one theme): 5.5% of twins in phase 1, 4.4% of twins in phase 2.

As well as the varying number of measures with QC items, other factors caused variations in the percentages. For example, QC item errors were more likely in measures that were long, repetitive and perhaps "boring" to twins. The use of QC items was less well planned in phase 1, with the result that they were unevenly spread through the questionnaire and, in certain measures, the % of QC errors was higher than perhaps it should have been.

Supplementary Tables

Table S1. Descriptive statistics for psychological traits, for the whole and males and females separately**.**

Trait (possible range)	All					Males					Females					ANOVA[a]		
	N	Min	Max	*M*	*SD*	*n*	Min	Max	*M*	*SD*	*n*	Min	Max	*M*	*SD*	Sex	*p*	R^2
Daily hassles (0-28)	4324	.00	28.00	12.54	4.63	1609	.00	28.00	11.96	4.58	2715	.00	28.00	12.88	4.62	4.40	<.001	.01
CHAOS (0-12)	4875	.00	12.00	3.81	2.22	1860	.00	12.00	3.73	2.05	3015	.00	12.00	3.86	2.32	4.05	.044	.00
Childhood experiences (0-32)	4321	.00	32.00	5.31	4.59	1607	.00	32.00	5.27	4.11	2714	.00	32.00	5.33	4.85	.19	.665	.00
Poor sleep quality (0-24)	4250	.00	24.00	4.85	3.84	1578	.00	22.00	4.09	3.52	2672	.00	24.00	5.29	3.96	104.74	<.001	.02
Marriage hopes (1-5)	4800	1.00	5.00	3.85	.60	1829	1.00	5.00	3.85	.59	2971	1.00	5.00	3.84	.60	.39	.532	.00
Marriage worries (1-5)	4801	1.00	5.00	2.37	.68	1829	1.00	5.00	2.51	.69	2972	1.00	5.00	2.29	.66	126.53	<.001	.03
Quality of relationship with twin (1-5)	4710	1.00	5.00	3.94	.88	1785	1.00	5.00	3.72	.87	2925	1.00	5.00	4.07	.86	179.85	<.001	.04
Quality of relationship with mother (1-5)	4658	1.00	5.00	3.83	.96	1764	1.00	5.00	3.71	.89	2894	1.00	5.00	3.91	1.00	52.03	<.001	.01
Quality of relationship with father (1-5)	4541	1.00	5.00	3.27	1.06	1716	1.00	5.00	3.26	1.01	2825	1.00	5.00	3.27	1.09	.20	.654	.00
Number of relationships (0-4)	4798	.00	4.00	1.22	.80	1827	.00	4.00	1.24	.85	2971	.00	4.00	1.20	.76	2.47	.116	.00
Longest relationship (1-5)	4022	1.00	5.00	3.13	1.20	1510	1.00	5.00	2.91	1.20	2512	1.00	5.00	3.26	1.19	81.70	<.001	.02
Partner violence (1-5)	4642	1.00	5.00	1.77	.96	1749	1.00	5.00	1.75	.82	2893	1.00	5.00	1.78	1.03	1.39	.239	.00
Contact with mother (1-6)	1960	1.00	6.00	3.17	1.19	708	1.00	6.00	2.91	1.10	1252	1.00	6.00	3.31	1.22	54.86	<.001	.03
Communication with mother (0-6)	1999	.00	6.00	4.72	1.13	722	.00	6.00	4.33	1.10	1277	.00	6.00	4.94	1.10	143.39	<.001	.07

Communication with mother (1-6)	2140	1.00	6.00	3.02	1.19	772	1.00	6.00	2.88	1.13	1368	1.00	6.00	3.11	1.21	19.06	<.001	.01
Communication with father (0-6)	2335	.00	6.00	3.72	1.56	840	.00	6.00	3.57	1.51	1495	.00	6.00	3.80	1.59	12.40	<.001	.01
Peer pressure (1-5)	4745	1.00	5.00	1.96	.72	1812	1.00	4.57	2.08	.74	2933	1.00	5.00	1.90	.70	7.32	<.001	.01
Physical peer victimisation (0-8)	4196	.00	8.00	.23	.88	1566	.00	8.00	.39	1.12	2630	.00	8.00	.14	.68	63.59	<.001	.02
Social peer victimisation (0-8)	4195	.00	8.00	.74	1.55	1566	.00	8.00	.70	1.47	2629	.00	8.00	.77	1.60	1.69	.194	.00
Verbal peer victimisation (0-8)	4195	.00	8.00	2.03	2.53	1565	.00	8.00	2.47	2.69	2630	.00	8.00	1.77	2.39	71.07	<.001	.02
Cyber peer victimisation (0-8)	4195	.00	8.00	.59	1.43	1565	.00	8.00	.50	1.36	2630	.00	8.00	.63	1.47	8.82	.003	.00
Physical peer Perpetration (0-8)	4195	.00	8.00	.16	.74	1564	.00	8.00	.32	1.06	2631	.00	7.00	.07	.44	79.75	<.001	.03
Social peer Perpetration (0-8)	4195	.00	8.00	.42	.90	1564	.00	8.00	.41	.88	2631	.00	8.00	.43	.92	.88	.349	.00
Verbal peer Perpetration (0-8)	4195	.00	8.00	1.53	2.14	1564	.00	8.00	2.05	2.49	2631	.00	8.00	1.22	1.84	129.84	<.001	.03
Cyber peer Perpetration (0-8)	4195	.00	8.00	.27	.89	1564	.00	8.00	.29	.95	2631	.00	8.00	.26	.85	1.17	.280	.00
Parental role aspirations (1-5)	4334	1.00	5.00	4.18	.76	1612	1.00	5.00	4.06	.77	2722	1.00	5.00	4.25	.74	62.14	<.001	.01
Occupational role aspirations (1-5)	4336	1.00	5.00	3.96	.57	1614	1.00	5.00	3.94	.58	2722	1.25	5.00	3.97	.56	2.21	.137	.00
Homecare role aspirations (1-5)	4336	1.00	5.00	3.98	.64	1614	1.00	5.00	3.86	.68	2722	1.75	5.00	4.05	.60	89.96	<.001	.02

Importance of Relationships (0-20)	4859	.00	2.00	15.93	3.49	1860	.00	2.00	14.81	3.79	2999	.00	2.00	16.62	3.09	301.17	<.001	.06
Achievement Motivation (0-16)	4859	.00	16.00	1.24	2.85	1860	.00	16.00	1.16	3.00	2999	.00	16.00	1.28	2.75	2.27	.132	.00
Purpose in Life (1-5)	4854	1.00	5.00	3.46	.80	1858	1.00	5.00	3.38	.81	2996	1.00	5.00	3.51	.80	26.79	<.001	.01
Importance of democracy and equality (1-5)	4846	1.00	5.00	4.28	.55	1854	1.00	5.00	4.20	.58	2992	1.00	5.00	4.32	.53	52.04	<.001	.01
Environmental concerns (1-5)	4851	1.00	5.00	3.72	.69	1857	1.00	5.00	3.71	.72	2994	1.00	5.00	3.73	.67	1.06	.303	.00
Religiosity (0-25)	4751	.00	25.00	4.43	4.98	1816	.00	25.00	3.99	4.85	2935	.00	25.00	4.70	5.04	23.80	<.001	.00
Importance of leisure (1-5)	4243	1.00	5.00	3.83	.77	1569	1.00	5.00	3.94	.75	2674	1.00	5.00	3.76	.77	6.63	<.001	.01
Alcohol use (0-40)	3889	.00	36.00	8.13	5.01	1461	.00	36.00	8.76	5.29	2428	.00	34.00	7.75	4.79	35.78	<.001	.01
Ever smoked (0-1)	4254	.00	1.00	.57	.50	1579	.00	1.00	.57	.50	2675	.00	1.00	.56	.50	.42	.518	.00
Ever vaped (0-1)	4254	.00	1.00	.27	.44	1578	.00	1.00	.31	.46	2676	.00	1.00	.24	.43	24.64	<.001	.01
Cognitive enhancers (0-12)	4213	.00	12.00	1.19	1.95	1560	.00	12.00	1.42	2.14	2653	.00	12.00	1.06	1.82	32.00	<.001	.01
Big 5 Agreeableness (1-5)	4708	1.00	5.00	3.71	.57	1834	1.00	5.00	3.55	.58	2874	1.33	5.00	3.81	.54	246.80	<.001	.05
Big 5 Conscientiousness (1-5)	4702	1.00	5.00	3.87	.62	1832	1.00	5.00	3.76	.65	2870	1.17	5.00	3.93	.59	78.99	<.001	.02
Big 5 Extraversion (1-5)	4726	1.00	5.00	3.50	.66	1843	1.00	5.00	3.42	.68	2883	1.00	5.00	3.55	.63	4.33	<.001	.01
Big 5 Neuroticism (1-5)	4733	1.00	4.83	2.64	.66	1847	1.00	4.67	2.51	.63	2886	1.00	4.83	2.72	.67	116.17	<.001	.02
Big 5 Openness (1-5)	4715	1.00	5.00	3.42	.61	1838	1.00	5.00	3.39	.63	2877	1.25	5.00	3.44	.60	7.29	.007	.00
Self-control (0-24)	4699	.00	24.00	14.33	4.20	1831	.00	24.00	13.73	4.36	2868	.00	24.00	14.72	4.05	6.13	<.001	.01

Not planning for the future (1-5)	4697	1.00	5.00	2.47	.94	1831	1.00	5.00	2.64	.95	2866	1.00	5.00	2.35	.92	105.81	<.001	.02
Ambition (0-20)	4326	.00	2.00	13.81	3.61	1610	1.00	2.00	13.80	3.74	2716	.00	2.00	13.82	3.52	.03	.875	.00
Physical activity (1-5)	4758	1.00	5.00	2.80	1.08	1804	1.00	5.00	2.93	1.10	2954	1.00	5.00	2.71	1.06	44.39	<.001	.01
Health behaviours (0-48)	4769	1.00	48.00	25.27	6.65	1811	1.00	48.00	24.81	6.75	2958	1.00	46.00	25.55	6.57	13.75	<.001	.00
Risk taking behaviour (0-24)	4697	.00	23.00	6.51	3.58	1830	.00	21.00	7.54	3.82	2867	.00	23.00	5.86	3.25	244.01	<.001	.05
Risky sexual behaviour (0-16)	4554	.00	12.67	4.22	2.75	1736	.00	12.67	4.02	2.75	2818	.00	12.00	4.34	2.75	14.52	<.001	.00
Media use (0-20)	4719	.00	2.00	12.90	3.24	1794	.00	2.00	11.99	3.53	2925	.00	2.00	13.45	2.91	217.54	<.001	.05
Volunteering (0-20)	4754	.00	19.00	5.65	3.24	1817	.00	18.00	5.44	3.22	2937	.00	19.00	5.77	3.24	11.97	.001	.00
Internet dating (scale) (0-5)	4706	.00	5.00	2.79	2.17	1788	.00	5.00	3.04	2.11	2918	.00	5.00	2.64	2.19	39.25	<.001	.01
Internet dating (y/n) (0-1)	4717	.00	1.00	.46	.50	1793	.00	1.00	.51	.50	2924	.00	1.00	.44	.50	21.50	<.001	.00
Financial literacy: Knowledge of Products (0-4)	4637	.00	4.00	1.38	.69	1778	.00	4.00	1.55	.73	2859	.00	4.00	1.27	.64	182.00	<.001	.04
Financial attitudes and behaviour (1-5)	4636	1.17	5.00	3.54	.61	1778	1.17	5.00	3.48	.64	2858	1.17	5.00	3.59	.59	34.52	<.001	.01

Note. Raw uncorrected scores were used. These results are based on one twin randomly selected from each pair so that the data points are independent.

[a] *F* and *p* values are reported with Welch's correction (Welch, 1951) as the assumption of homogeneity of variance was violated for several variables.

Table S2. Descriptive statistics for psychological traits, for five sex and zygosity groups.

Trait (possible range)	MZ males			DZ males			MZ females			DZ females			DZ opposite sex			ANOVA		
	n	*M*	*SD*	*n*	*M*	*SD*	*n*	*M*	*SD*	*n*	*M*	*SD*	*n*	*M*	*SD*	Zygosity	Sex* Zygosity	R^2 (adjusted)
Daily hassles (0-28)	543	11.76	4.63	478	11.94	4.59	1055	12.88	4.69	893	12.97	4.64	1313	12.51	4.53	.80	.21	.013 (.010)
CHAOS (0-12)	606	3.69	2.09	555	3.80	2.10	1152	3.76	2.27	993	3.95	2.35	1514	3.76	2.17	.29	3.65	.004 (.001)
Childhood experiences (0-32)	544	5.36	3.80	475	5.06	4.19	1052	5.50	5.22	892	5.23	4.52	1316	5.22	4.48	.26	.34	.001 (-.002)
Poor sleep quality (0-24)	534	4.04	3.70	470	4.03	3.29	1031	5.45	4.08	882	5.17	3.82	1291	4.75	3.78	.00	1.24	.025 (.022)
Marriage hopes (1-5)	593	3.85	.58	548	3.89	.60	1134	3.85	.61	978	3.82	.58	1494	3.85	.60	1.55	.05	.002 (-.001)
Marriage worries (1-5)	593	2.49	.68	548	2.50	.66	1134	2.27	.67	978	2.29	.66	1495	2.40	.70	.03	.04	.033 (.030)
Quality of relationship with twin (1-5)	580	4.07	.79	537	3.57	.84	1114	4.35	.78	962	4.04	.83	1465	3.64	.89	8.22**	.00	.106 (.103)
Quality of relationship with mother (1-5)	569	3.68	.93	534	3.73	.87	1105	3.91	1.01	948	3.91	.99	1452	3.84	.95	.19	.14	.009 (.006)
Quality of relationship with father (1-5)	559	3.25	1.07	514	3.32	.96	1075	3.25	1.11	923	3.24	1.09	1421	3.28	1.05	.09	.92	.001 (-.002)
Number of relationships (0-4)	593	1.18	.86	546	1.28	.84	1132	1.18	.78	979	1.20	.73	1495	1.26	.81	.00	2.41	.005 (.003)
Longest relationship (1-5)	473	2.94	1.18	467	2.97	1.22	939	3.26	1.18	839	3.26	1.22	1260	3.08	1.19	.36	.10	.019 (.016)
Partner violence (1-5)	569	1.73	.82	523	1.74	.84	1099	1.76	1.02	956	1.78	1.03	1442	1.79	.94	.04	.03	.001 (-.002)
Contact with mother (1-6)	254	3.00	1.15	195	2.87	1.02	482	3.31	1.22	419	3.38	1.27	598	3.07	1.15	.99	.34	.023 (.020)
Communication with mother (0-6)	258	4.40	1.10	199	4.34	1.06	494	4.91	1.18	425	5.02	1.07	610	4.63	1.10	.00	.08	.103 (.100)
Communication with mother (1-6)	270	2.97	1.21	218	2.90	1.10	516	3.05	1.18	460	3.22	1.29	658	2.94	1.12	.08	1.08	.009 (.006)
Communication with father (0-6)	297	3.62	1.57	233	3.64	1.46	571	3.73	1.61	498	3.90	1.60	718	3.65	1.53	.26	.13	.019 (.016)
Peer pressure (1-5)	585	1.98	.71	546	2.06	.74	1118	1.87	.68	968	1.89	.70	1479	2.04	.74	3.50	.53	.015 (.012)
Physical peer victimisation (0-8)	528	.39	1.16	469	.44	1.12	1020	.14	.70	869	.11	.55	1269	.25	.94	.41	.53	.035 (.032)
Social peer victimisation (0-8)	528	.63	1.33	469	.69	1.50	1020	.74	1.58	869	.78	1.60	1268	.79	1.59	.56	.90	.001 (-.001)
Verbal peer victimisation (0-8)	528	2.28	2.59	468	2.57	2.71	1020	1.64	2.34	869	1.77	2.35	1269	2.23	2.64	2.56	1.18	.036 (.034)

Cyber peer victimisation (0-8)	528	.49	1.35	468	.54	1.37	1020	.63	1.47	869	.58	1.41	1269	.60	1.47	.03	.40	.001 (-.001)
Physical peer Perpetration (0-8)	526	.34	1.14	469	.36	1.07	1020	.07	.44	870	.07	.42	1269	.17	.73	1.15	2.85	.044 (.041)
Social peer Perpetration (0-8)	526	.40	.83	469	.40	.94	1020	.42	.92	870	.43	.93	1269	.43	.88	.01	.02	.000 (-.003)
Verbal peer Perpetration (0-8)	526	1.92	2.45	469	2.01	2.48	1020	1.08	1.73	870	1.28	1.87	1269	1.74	2.26	2.75	.00	.040 (.038)
Cyber peer Perpetration (0-8)	526	.24	.82	469	.33	1.07	1020	.25	.85	870	.23	.76	1269	.30	.96	2.12	.21	.006 (.003)
Parental role aspirations (1-5)	548	4.07	.76	476	4.03	.74	1057	4.27	.71	895	4.25	.73	1316	4.16	.80	.03	.00	.007 (.004)
Occupational role aspirations (1-5)	548	3.94	.58	476	3.91	.60	1057	3.98	.55	895	3.96	.57	1318	3.97	.57	.08	.10	.001 (-.002)
Homecare role aspirations (1-5)	548	3.90	.68	476	3.81	.69	1057	4.08	.60	895	4.04	.58	1318	3.96	.65	.45	.20	.012 (.010)
Importance of Relationships (0-20)	603	14.74	3.90	556	14.85	3.73	1141	16.65	3.11	993	16.53	3.06	1512	15.87	3.51	.96	1.31	.060 (.057)
Achievement Motivation (0-16)	603	1.26	3.01	556	1.06	3.06	1141	1.30	2.77	993	1.31	2.69	1512	1.20	2.87	1.87	3.96*	.004 (.002)
Purpose in Life (1-5)	602	3.40	.83	556	3.39	.80	1139	3.52	.80	993	3.48	.78	1510	3.45	.80	.06	.80	.005 (.002)
Importance of democracy and equality (1-5)	602	4.22	.57	554	4.22	.54	1135	4.31	.51	992	4.31	.54	1508	4.27	.59	1.48	.01	.021 (.018)
Environmental concerns (1-5)	602	3.70	.73	556	3.74	.70	1137	3.72	.65	992	3.75	.69	1509	3.70	.70	1.36	.10	.002 (-.001)
Religiosity (0-25)	586	4.28	5.30	548	4.07	4.83	1119	4.87	5.25	968	4.45	4.73	1480	4.22	4.79	1.00	.27	.004 (.001)
Importance of leisure (1-5)	528	3.93	.76	471	3.97	.73	1031	3.72	.77	885	3.75	.78	1286	3.88	.76	1.60	1.37	.018 (.016)
Alcohol use (0-40)	484	8.56	5.29	436	8.76	5.24	932	7.49	4.68	807	7.88	4.94	1195	8.41	5.05	.11	1.63	.008 (.005)
Ever smoked (0-1)	530	.52	.50	471	.58	.49	1034	.54	.50	883	.57	.50	1295	.61	.49	2.33	1.01	.006 (.003)
Ever vaped (0-1)	530	.30	.46	472	.29	.45	1035	.22	.42	884	.24	.43	1292	.30	.46	1.76	2.09	.005 (.003)
Cognitive enhancers (0-12)	521	1.30	2.07	467	1.36	2.11	1029	1.07	1.88	876	1.05	1.76	1280	1.29	2.03	.33	.05	.003 (.001)
Big 5 Agreeableness (1-5)	598	3.56	.55	551	3.55	.58	1095	3.83	.53	956	3.80	.54	1459	3.68	.59	.57	.18	.046 (.044)

Big 5 Conscientiousness (1-5)	598	3.83	.62	550	3.74	.67	1094	3.96	.57	954	3.91	.59	1457	3.83	.63	2.05	.35	.012 (.009)
Big 5 Extraversion (1-5)	601	3.39	.70	552	3.44	.67	1101	3.52	.63	956	3.54	.64	1466	3.53	.65	.00	.13	.010 (.008)
Big 5 Neuroticism (1-5)	602	2.50	.63	553	2.53	.64	1104	2.73	.66	956	2.73	.67	1468	2.60	.66	.43	.13	.034 (.032)
Big 5 Openness (1-5)	600	3.39	.61	552	3.39	.63	1098	3.42	.60	956	3.45	.60	1460	3.41	.62	3.32	.44	.007 (.004)
Self-control (0-24)	598	14.02	4.21	550	13.73	4.43	1092	14.92	4.00	954	14.47	4.12	1456	14.18	4.25	1.89	4.02*	.019 (.016)
Not planning for the future (1-5)	598	2.60	.95	550	2.64	.94	1092	2.34	.93	952	2.37	.92	1456	2.51	.95	.21	.12	.021 (.018)
Ambition (0-20)	544	13.93	3.69	478	13.65	3.84	1055	13.86	3.55	893	13.79	3.52	1314	13.81	3.61	2.32	1.36	.003 (.001)
Physical activity (1-5)	585	2.95	1.11	543	2.92	1.09	1129	2.67	1.07	970	2.68	1.03	1478	2.87	1.09	.83	.22	.011 (.008)
Health behaviours (0-48)	588	24.85	6.56	543	24.89	6.85	1129	25.41	6.62	973	25.49	6.48	1483	25.30	6.73	.62	.89	.003 (.000)
Risk taking behaviour (0-24)	598	7.26	3.86	548	7.44	3.73	1093	5.59	3.10	953	5.92	3.43	1457	6.96	3.61	5.71*	2.78	.046 (.044)
Risky sexual behaviour (0-16)	561	3.91	2.85	522	4.02	2.71	1071	4.16	2.80	923	4.38	2.71	1427	4.37	2.71	.93	9.93*	.018 (.015)
Media use (0-20)	581	11.91	3.67	540	11.88	3.61	1122	13.48	2.89	958	13.45	2.82	1466	12.85	3.22	3.17	2.13	.042 (.039)
Volunteering (0-20)	586	5.57	3.24	548	5.51	3.19	1120	5.66	3.29	969	5.82	3.21	1481	5.61	3.21	1.86	6.48*	.010 (.008)
Internet dating (scale) (0-5)	579	3.00	2.14	541	2.92	2.09	1116	2.57	2.19	959	2.59	2.21	1459	2.98	2.13	.29	.98	.014 (.012)
Internet dating (y/n) (0-1)	581	.50	.50	542	.47	.50	1120	.42	.49	960	.43	.50	1462	.50	.50	.86	1.79	.011 (.008)
Financial literacy: Knowledge of Products (0-4)	582	1.60	.75	539	1.56	.73	1088	1.26	.63	936	1.30	.65	1440	1.38	.67	.79	.79	.046 (.043)
Financial attitudes and behaviour (1-5)	582	3.53	.60	539	3.45	.65	1088	3.59	.59	936	3.59	.59	1439	3.52	.63	.20	.80	.018 (.015)

Note. MZ=monozygotic; DZ=dizygotic. Raw uncorrected scores were used. These results are based on one twin randomly selected from each pair so that the data points are independent. Group difference = *F* statistics; **p < .05. **p < .001.

Table S3. Summary of measured variables used

Theme	Variable name	Scale/item[a]	No. of items used	Reference[b]
**Emerging adulthood trait variables**				
**Life experiences**	Daily hassles	Hassles	7	Delongis, A., Folkman, S. and Lazarus, R. S. (1988)
	CHAOS	CHAOS<br>(Confusion, Hubbub and Order Scale) at home	6	Matheny, Wachs, Ludwig and Phillips (1995)
	Childhood experiences	Childhood experiences	8	Taken from the ALSPAC "Life at 22+" questionnaire<br>(section H) with some minor changes in wording:<br>http://www.bristol.ac.uk/media-library/sites/alspac/documents/questionnaires/YPB-life-at-22-plus.pdf
	Sleep quality	The Pittsburgh Sleep Quality Index<br>(PSQI)	8	Buysse D.J., Reynolds C.F., Monk T.H., Berman S.R. & Kupfer D.J. (1989)
**Relationships**	Marriage hopes	Marriage hopes	4	Kahn, S. (2007)
	Marriage worries	Marriage worries	4	Kahn, S. (2007)
	Quality of relationship with twin	Relationship with twin	5	Riggio, H. R. (2000)

	Quality of relationship with mother	Relationship with mother	5	Fraley, R. C., Heffernan, M. E., Vicary, A. M., & Brumbaugh, C. C. (2011)
	Quality of relationship with father	Relationship with father	5	Fraley, R. C., Heffernan, M. E., Vicary, A. M., & Brumbaugh, C. C. (2011)
	Number of relationships	Number of relationships	1	Item created by the TEDS team.
	Longest relationship	Longest relationship	1	Item created by the TEDS team.
	Partner violence	Intimate Partner Violence Questions	6	Adapted from the 'Intimate Partner Violence Questions' section of the CDC (Centers for Disease Control and Prevention) Violence Prevention questionnaire: https://www.cdc.gov/violenceprevention/pdf/ipv/ipvandsvscreening.pdf
	Contact with mother	Parent contact	3	Items created by TEDS researchers.
	Communication with mother	Parent contact	2	Items created by TEDS researchers.
	Contact with father	Parent contact	3	Items created by TEDS researchers.
	Communication with father	Parent contact	2	Items created by TEDS researchers.
	Peer pressure	Peer pressure	7	Shortened from Santor, D. A., Messervey, D., & Kusumakar, V. (2000)

	Physical peer victimisation	Multidimensional Peer Victimisation Scale	4	Mynard, H., and Joseph (2000)
	Social peer victimisation	Multidimensional Peer Victimisation Scale	4	Mynard, H., and Joseph (2000)
	Verbal peer victimisation	Multidimensional Peer Victimisation Scale	4	Mynard, H., and Joseph (2000)
	Cyber peer victimisation	Multidimensional Peer Victimisation Scale	4	Mynard, H., and Joseph (2000)
	Physical peer Perpetration	Multidimensional Peer Victimisation Scale	4	Mynard, H., and Joseph (2000)
	Social peer Perpetration	Multidimensional Peer Victimisation Scale	4	Mynard, H., and Joseph (2000)
	Verbal peer Perpetration	Multidimensional Peer Victimisation Scale	4	Mynard, H., and Joseph (2000)
	Cyber peer Perpetration	Multidimensional Peer Victimisation Scale	4	Mynard, H., and Joseph (2000)
**Aspirations**	Parental role aspirations	Life Role Salience (LRSS), Parental role	4	Amatea, E.S., Cross, E.G., Clark, J.E. and Bobby, C.L. (1986)
	Occupational role aspirations	Life Role Salience (LRSS), Occupational role	4	Amatea, E.S., Cross, E.G., Clark, J.E. and Bobby, C.L. (1986)

	Homecare role aspirations	Life Role Salience (LRSS), Homecare role	4	Amatea, E.S., Cross, E.G., Clark, J.E. and Bobby, C.L. (1986)
**Thoughts and attitudes**	Importance of Relationships	GOALS – relationships	5	Pöhlmann and Brunstein (1997)
	Achievement Motivation	GOALS – self-fulfilment	4	Pöhlmann and Brunstein (1997)
	Purpose in Life	Purpose in Life	5	Crumbaugh and Maholick (1964)
	Importance of democracy and equality	BSA Democracy and Government	5	Taken from the NatCen Social Research's British Social Attitudes (BSA) survey (http://natcen.ac.uk/our-research/research/british-social-attitudes/).
	Environmental concerns	BSA Environmental concerns	6	Taken from the NatCen Social Research's British Social Attitudes (BSA) Survey (http://natcen.ac.uk/our-research/research/british-social-attitudes/).
	Religiosity	Religiosity: the Centrality of Religiosity Scale (CRS)	5	Huber, S., & Huber, O. W. (2012)
	Importance of leisure	Leisure and Hobbies	5	Lavallee, L. F., Hatch, P. M., Michalos, A. C., & McKinley, T. (2007).
**Substance use**	Alcohol use	Alcohol Use Disorders Identification Test (AUDIT) scale	10	Adapted from items 1-10 of the AUDIT scale: https://www.drugabuse.gov/sites/default/files/files/AUDIT.pdf
	Ever smoked	Smoking (inc. vaping)	1	Adapted from Heatherton, Kozlowski, Frecker, and Fagerstrom (1991)

	Ever vaped	Smoking<br><br>(inc. vaping)	1	Adapted from Heatherton, Kozlowski, Frecker, and Fagerstrom (1991)
	Cognitive enhancers	Cognitive Enhancers	3	Items created by the TEDS team.
**Personality**	Big 5 personality	Big 5 Personality scale<br><br>(Agreeableness, conscientiousness, extraversion, neuroticism, and openness subscales).	30	Mullins-Sweatt, S. N., Jamerson, J. E., Samuel, D. B., Olson, D. R., Widiger, T. A. (2006).
	Self-control	Self-Control.	6	Tangney, J. P., Boone, A. L., & Baumeister, R. F. (2018)
	Not planning for the future	Consideration of Future Consequences Scale.	4	Strathman, A., Gleicher, F., Boninger, D. S., & Edwards, C. S. (1994).
	Ambition	Grit: Perseverance and<br><br>passion for long-term goals.	5	Duckworth, A.L., Peterson, C., Matthews, M.D., & Kelly, D.R. (2007)
**Behaviours**	Physical activity	Activity questionnaire	3	Questions devised by TEDS researchers.
	Health behaviours	Rapid Eating and Activity Assessment for Patients<br><br>(REAP) Health Behaviours	12	Adapted from Gans, K. M., Risica, P. M., Wylie-Rosett, J., Ross, E. M., Strolla, L. O., McMurray, J., & Eaton, C. B. (2006)
	Risk taking behaviour	Risk taking index	6	Nicholson, N., Soane, E., Fenton-O'Creevy, M., & Willman, P. (2005)

	Risky sexual behaviour	Sexual Behaviours and Experience	5	Adapted from the ALSPAC measure: http://www.bristol.ac.uk/media-library/sites/alspac/documents/questionnaires/YPC-me-at-23.pdf
	Media use	Media and Technology Usage and Attitudes Scale	4	Rosen, Whaling, Carrier, Cheever, & Rokkum (2013)
	Volunteering	Volunteering	5	Adapted from page 42 of the ALSPAC questionnaire "It's all about you" at age 20: http://www.bristol.ac.uk/media-library/sites/alspac/migrated/documents/ ques-yp20-all-about-you.pdf
	Internet dating	Internet dating	2	Items created by TEDS researchers, in order to understand the proportion of emerging adults that take part in internet dating.
**Financial attitudes and behaviour**	Financial literacy: Knowledge of Products	OECD Financial literacy/products	13	Adapted from sections of the OECD instrument for measuring financial literacy: https://www.oecd.org/finance/financial-education/49319977.pdf
	Financial attitudes and behaviour	OECD Attitudes towards money	6	Adapted from sections of the OECD instrument for measuring financial literacy: https://www.oecd.org/finance/financial-education/49319977.pdf
**Wellbeing variables**				
**-**	Love and Relationships	CLAS, Love and Relationships	3	Lavallee, Hatch, Michalos, and McKinley (2007)
**-**	Community satisfaction	CLAS Life Satisfaction Scale, Community.	5	Lavallee, Hatch, Michalos, and McKinley (2007)

-	Financial wellbeing	CLAS Financial wellbeing	5	Lavallee, Hatch, Michalos, and McKinley (2007)
**Mental health variables**				
-	Conners	Conners, 3rd edition. Impulsivity and inattention subscales.	20	Conners, C. K. 2008)
-	Aggression	Brief Aggression Questionnaire (BAQ).	8	Webster, G. D., DeWall, C. N., Pond Jr, R. S., Deckman, T., Jonason, P. K., Le, B. M., ... & Smith, C. V. (2014)
-	Conduct problems	SDQ, Conduct problems	5	Goodman (1997)
-	Emotional problems	SDQ, Emotional problems	5	Goodman (1997)
-	Hyperactivity	SDQ, Hyperactivity	5	Goodman (1997)
-	Peer problems	SDQ, Peer problems	5	Goodman (1997)
-	General anxiety	General Anxiety: the Severity Measure for Generalized Anxiety Disorder	10	Craske et al. (2013)
-	Depression	Short Mood and Feeling Questionnaire (SMFQ)	8	Adapted from Angold et al. (1995)
-	Self-harm	CASE, Self-harm	2	Adapted from Madge et al. (2008)

-	Psychotic experiences, paranoia	SPEQ: Specific Psychotic Experiences Questionnaire: Paranoia subscale	12	Ronald, A., Sieradzka, D., Cardno, A. G., Haworth, C. M. A, McGuire, P., Freeman, D. (2014)
-	Psychotic experiences, hallucinations	SPEQ: Specific Psychotic Experiences Questionnaire: Hallucinations subscale	12	Ronald, A., Sieradzka, D., Cardno, A. G., Haworth, C. M. A, McGuire, P., Freeman, D. (2014)
**Physical health variables**				
-	Use of painkillers	Use of over-the-counter painkillers	1	Devised by TEDS researchers.
-	Use of antibiotics	Use of antibiotics	1	Devised by TEDS researchers.
-	General self-reported health	RAND Short-Form Health Survey	5	Adapted from Ware, J., Jr., & Sherbourne, C.D. The MOS 36-Item Short-Form Health Survey<br>(SF-36): I. Conceptual Framework and Item Selection, in Medical Care, June 1992, Vol. 30, No. 6, pp 473-483
-	Hospitalisation	RAND Short-Form Health Survey.	2	Adapted from Ware, J., Jr., & Sherbourne, C.D. The MOS 36-Item Short-Form Health Survey (SF-36): I. Conceptual Framework and Item Selection, in Medical Care, June 1992, Vol. 30, No. 6, pp 473-483
-	Short Health Economics	Short Health Economics.<br>Questions from the EQ-5D-5L measure.	4	Herdman M., Gudex, C., Lloyd, A. (2011).

**Education variables**				
-	Achieved educational level	Educational level (current)	1	Questions devised by TEDS researchers.
-	Planned education level	Educational level (aspirational)	1	Questions devised by TEDS researchers.

[a] More information about the variables and the references can be found in the TEDS data dictionary (http://www.teds.ac.uk/datadictionary/studies/measures/21yr_measures.htm).

[b] Note that measures were shortened and adapted from the referenced measures.

Table S4. Descriptive statistics for psychological traits, for the whole and males and females separately (repeated with the twin that was not randomly selected from each pair in the main analyses).

Trait (possible range)	All					Males					Females					ANOVA[a]		
	N	Min	Max	*M*	*SD*	*n*	Min	Max	*M*	*SD*	*n*	Min	Max	*M*	*SD*	Sex	*p*	R^2
Daily hassles (0-28)	4327	.00	28.00	12.58	4.70	1553	.00	28.00	11.96	4.67	2774	.00	28.00	12.92	4.68	4.40	.000	.01
CHAOS (0-12)	4856	.00	12.00	3.85	2.22	1795	.00	12.00	3.82	2.13	3061	.00	12.00	3.87	2.27	4.05	.044	.00
Childhood experiences (0-32)	4323	.00	32.00	5.34	4.56	1548	.00	31.00	5.19	4.06	2775	.00	32.00	5.43	4.81	.19	.665	.00
Poor sleep quality (0-24)	4239	.00	24.00	4.95	3.90	1515	.00	21.00	4.10	3.49	2724	.00	24.00	5.43	4.04	104.74	.000	.02
Marriage hopes (1-5)	4776	1.00	5.00	3.85	.59	1772	1.00	5.00	3.84	.58	3004	1.00	5.00	3.86	.60	.39	.532	.00
Marriage worries (1-5)	4775	1.00	5.00	2.38	.69	1771	1.00	5.00	2.55	.69	3004	1.00	5.00	2.29	.67	126.53	.000	.03
Quality of relationship with twin (1-5)	4680	1.00	5.00	3.93	.90	1722	1.00	5.00	3.73	.87	2958	1.00	5.00	4.04	.90	179.85	.000	.04
Quality of relationship with mother (1-5)	4646	1.00	5.00	3.83	.96	1706	1.00	5.00	3.65	.90	2940	1.00	5.00	3.93	.97	52.03	.000	.01
Quality of relationship with father (1-5)	4539	1.00	5.00	3.21	1.08	1658	1.00	5.00	3.24	.99	2881	1.00	5.00	3.20	1.13	.20	.654	.00
Number of relationships (0-4)	4776	.00	4.00	1.21	.79	1772	.00	4.00	1.25	.85	3004	.00	4.00	1.18	.75	2.47	.116	.00
Longest relationship (1-5)	4024	1.00	5.00	3.09	1.22	1479	1.00	5.00	2.86	1.21	2545	1.00	5.00	3.22	1.21	81.70	.000	.02
Partner violence (1-5)	4618	1.00	5.00	1.77	.97	1693	1.00	5.00	1.73	.84	2925	1.00	5.00	1.79	1.03	1.39	.239	.00
Contact with mother (1-6)	1960	1.00	6.00	3.19	1.20	676	1.00	6.00	2.94	1.09	1284	1.00	6.00	3.32	1.23	54.86	.000	.03
Communication with mother (0-6)	2007	.00	6.00	4.67	1.19	690	.00	6.00	4.30	1.12	1317	.00	6.00	4.86	1.18	143.39	.000	.07

Communication with mother (1-6)	2121	1.00	6.00	3.02	1.18	728	1.00	6.00	2.92	1.14	1393	1.00	6.00	3.07	1.20	19.06	.000	.01
Communication with father (0-6)	2328	.00	6.00	3.70	1.55	788	.00	6.00	3.62	1.43	1540	.00	6.00	3.75	1.61	12.40	.000	.01
Peer pressure (1-5)	4725	1.00	4.86	1.97	.71	1758	1.00	4.86	2.07	.73	2967	1.00	4.86	1.91	.70	7.32	.000	.01
Physical peer victimisation (0-8)	4187	.00	8.00	.22	.86	1503	.00	8.00	.39	1.14	2684	.00	8.00	.12	.63	63.59	.000	.02
Social peer victimisation (0-8)	4187	.00	8.00	.75	1.57	1503	.00	8.00	.72	1.52	2684	.00	8.00	.77	1.60	1.69	.194	.00
Verbal peer victimisation (0-8)	4186	.00	8.00	2.07	2.53	1502	.00	8.00	2.49	2.73	2684	.00	8.00	1.84	2.39	71.07	.000	.02
Cyber peer victimisation (0-8)	4185	.00	8.00	.59	1.44	1503	.00	8.00	.57	1.44	2682	.00	8.00	.60	1.44	8.82	.003	.00
Physical peer Perpetration (0-8)	4186	.00	8.00	.16	.76	1503	.00	8.00	.31	1.07	2683	.00	8.00	.07	.49	79.75	.000	.03
Social peer Perpetration (0-8)	4184	.00	8.00	.42	.88	1502	.00	8.00	.43	.95	2682	.00	8.00	.42	.83	.88	.349	.00
Verbal peer Perpetration (0-8)	4185	.00	8.00	1.54	2.12	1503	.00	8.00	2.08	2.49	2682	.00	8.00	1.24	1.81	129.84	.000	.03
Cyber peer Perpetration (0-8)	4185	.00	8.00	.27	.92	1503	.00	8.00	.31	1.02	2682	.00	8.00	.25	.85	1.17	.280	.00
Parental role aspirations (1-5)	4328	1.00	5.00	4.16	.77	1551	1.00	5.00	4.04	.76	2777	1.00	5.00	4.23	.76	62.14	.000	.01
Occupational role aspirations (1-5)	4331	1.00	5.00	3.95	.61	1551	1.00	5.00	3.96	.60	2780	1.00	5.00	3.94	.61	2.21	.137	.00
Homecare role aspirations (1-5)	4331	1.00	5.00	4.00	.63	1551	1.00	5.00	3.91	.65	2780	1.25	5.00	4.04	.61	89.96	.000	.02

Importance of Relationships (0-20)	4822	.00	2.00	15.92	3.56	1790	.00	2.00	14.88	3.87	3032	.00	2.00	16.53	3.21	301.17	.000	.06
Achievement Motivation (0-16)	4821	.00	16.00	1.19	2.91	1789	.00	16.00	1.17	3.09	3032	.00	16.00	1.21	2.80	2.27	.132	.00
Purpose in Life (1-5)	4819	1.00	5.00	3.47	.82	1788	1.00	5.00	3.42	.82	3031	1.00	5.00	3.49	.81	26.79	.000	.01
Importance of democracy and equality (1-5)	4808	1.00	5.00	4.28	.54	1785	1.00	5.00	4.19	.56	3023	1.00	5.00	4.33	.51	52.04	.000	.01
Environmental concerns (1-5)	4815	1.00	5.00	3.70	.70	1787	1.00	5.00	3.64	.74	3028	1.17	5.00	3.73	.67	1.06	.303	.00
Religiosity (0-25)	4730	.00	25.00	4.36	4.96	1762	.00	25.00	3.91	4.91	2968	.00	25.00	4.64	4.97	23.80	.000	.00
Importance of leisure (1-5)	4243	1.00	5.00	3.82	.76	1518	1.00	5.00	3.93	.71	2725	1.00	5.00	3.76	.77	6.63	.000	.01
Alcohol use (0-40)	3899	.00	37.00	8.05	5.06	1425	.00	32.00	8.79	5.29	2474	.00	37.00	7.61	4.88	35.78	.000	.01
Ever smoked (0-1)	4262	.00	1.00	.56	.50	1528	.00	1.00	.57	.50	2734	.00	1.00	.56	.50	.42	.518	.00
Ever vaped (0-1)	4259	.00	1.00	.25	.44	1524	.00	1.00	.29	.45	2735	.00	1.00	.24	.43	24.64	.000	.01
Cognitive enhancers (0-12)	4217	.00	12.00	1.19	1.96	1506	.00	12.00	1.38	2.16	2711	.00	12.00	1.08	1.83	32.00	.000	.01
Big 5 Agreeableness (1-5)	4674	1.00	5.00	3.72	.57	1760	1.00	5.00	3.55	.57	2914	1.00	5.00	3.82	.55	246.80	.000	.05
Big 5 Conscientiousness (1-5)	4666	1.00	5.00	3.88	.62	1757	1.00	5.00	3.78	.64	2909	1.00	5.00	3.94	.60	78.99	.000	.02
Big 5 Extraversion (1-5)	4694	1.00	5.00	3.52	.65	1772	1.00	5.00	3.45	.68	2922	1.00	5.00	3.56	.63	4.33	.000	.01
Big 5 Neuroticism (1-5)	4700	1.00	5.00	2.62	.67	1774	1.00	5.00	2.48	.66	2926	1.00	5.00	2.70	.66	116.17	.000	.02
Big 5 Openness (1-5)	4688	1.00	5.00	3.40	.62	1769	1.00	5.00	3.37	.64	2919	1.00	5.00	3.42	.61	7.29	.007	.00
Self-control (0-24)	4661	.00	24.00	14.46	4.21	1753	.00	24.00	13.86	4.30	2908	.00	24.00	14.83	4.11	6.13	.000	.01

Not planning for the future (1-5)	4655	1.00	5.00	2.47	.92	1749	1.00	5.00	2.58	.92	2906	1.00	5.00	2.40	.91	105.81	.000	.02
Ambition (0-20)	4328	1.00	2.00	13.85	3.61	1553	1.00	2.00	14.00	3.67	2775	1.00	2.00	13.77	3.58	.03	.875	.00
Physical activity (1-5)	4699	1.00	5.00	2.83	1.09	1733	1.00	5.00	2.96	1.10	2966	1.00	5.00	2.75	1.08	44.39	.000	.01
Health behaviours (0-48)	4721	2.00	48.00	25.24	6.67	1742	4.00	48.00	24.61	6.63	2979	2.00	47.00	25.61	6.66	13.75	.000	.00
Risk taking behaviour (0-24)	4651	.00	24.00	6.49	3.61	1748	.00	21.00	7.46	3.74	2903	.00	24.00	5.91	3.40	244.01	.000	.05
Risky sexual behaviour (0-16)	4568	.00	14.00	4.17	2.74	1706	.00	12.33	3.92	2.71	2862	.00	14.00	4.32	2.76	14.52	.000	.00
Media use (0-20)	4686	.00	2.00	12.98	3.23	1732	.00	2.00	12.04	3.66	2954	.00	2.00	13.54	2.79	217.54	.000	.05
Volunteering (0-20)	4735	.00	2.00	5.66	3.23	1763	.00	18.00	5.52	3.32	2972	.00	2.00	5.74	3.17	11.97	.001	.00
Internet dating (scale) (0-5)	4669	.00	5.00	2.81	2.17	1726	.00	5.00	3.09	2.09	2943	.00	5.00	2.64	2.20	39.25	.000	.01
Internet dating (y/n) (0-1)	4689	.00	1.00	.47	.50	1734	.00	1.00	.52	.50	2955	.00	1.00	.44	.50	21.50	.000	.00
Financial literacy: Knowledge of Products (0-4)	4612	.00	4.00	1.39	.70	1717	.00	4.00	1.58	.73	2895	.00	4.00	1.28	.65	182.00	.000	.04
Financial attitudes and behaviour (1-5)	4610	1.00	5.00	3.55	.62	1717	1.00	5.00	3.48	.65	2893	1.00	5.00	3.59	.61	34.52	.000	.01

Note. Raw uncorrected scores were used. These results are based on one twin randomly selected from each pair so that the data points are independent.

[a] *F* and *p* values are reported with Welch's correction (Welch, 1951) as the assumption of homogeneity of variance was violated for several variables.

Table S5. Descriptive statistics for psychological traits, for five sex and zygosity groups (repeated with the twin that was not randomly selected from each pair in the main analyses).

Trait (possible range)	MZ males			DZ males			MZ females			DZ females			DZ opposite sex			ANOVA		
	n	*M*	*SD*	*n*	*M*	*SD*	*n*	*M*	*SD*	*n*	*M*	*SD*	*n*	*M*	*SD*	Zygosity	Sex* Zygosity	R^2 (adjusted)
Daily hassles (0-28)	528	11.86	4.82	474	11.75	4.42	1058	12.82	4.82	912	12.97	4.71	1312	12.66	4.57	.13	.07	.022 (.019)
CHAOS (0-12)	594	3.66	1.99	543	3.89	2.20	1159	3.82	2.23	998	3.93	2.37	1514	3.86	2.19	5.66*	.89	.005 (.003)
Childhood experiences (0-32)	524	5.36	3.91	473	4.98	3.96	1058	5.57	5.02	910	5.50	4.83	1314	5.13	4.32	4.24*	.29	.004 (.001)
Poor sleep quality (0-24)	512	4.23	3.70	463	3.92	3.50	1036	5.43	4.07	902	5.35	4.06	1286	4.91	3.72	.42	2.87	.049 (.047)
Marriage hopes (1-5)	595	3.86	.58	531	3.84	.58	1145	3.86	.58	971	3.82	.61	1491	3.85	.59	4.09*	.72	.004 (.001)
Marriage worries (1-5)	594	2.51	.71	531	2.55	.70	1145	2.28	.67	971	2.29	.65	1491	2.41	.69	4.09*	.05	.034 (.032)
Quality of relationship with twin (1-5)	578	4.03	.84	520	3.56	.86	1125	4.35	.75	957	4.04	.87	1457	3.62	.89	67.88**	.03	.087 (.084)
Quality of relationship with mother (1-5)	569	3.71	.87	517	3.65	.92	1118	3.89	.98	951	3.92	.99	1449	3.84	.95	1.07	3.89*	.038 (.036)
Quality of relationship with father (1-5)	550	3.23	1.00	502	3.23	.98	1095	3.18	1.14	929	3.21	1.13	1421	3.24	1.05	.08	.29	.001 (-.002)
Number of relationships (0-4)	595	1.25	.85	532	1.24	.86	1145	1.18	.74	971	1.18	.77	1490	1.21	.77	1.00	1.85	.010 (.008)
Longest relationship (1-5)	495	2.88	1.21	439	2.92	1.23	970	3.22	1.25	816	3.26	1.18	1270	3.02	1.22	3.20	.14	.013 (.011)
Partner violence (1-5)	563	1.76	.88	511	1.70	.86	1115	1.81	1.05	946	1.76	1.00	1442	1.76	.94	.65	.71	.005 (.002)
Contact with mother (1-6)	230	2.90	1.03	214	2.99	1.10	502	3.27	1.23	428	3.34	1.24	579	3.20	1.22	.52	1.11	.018 (.015)
Communication with mother (0-6)	233	4.37	1.09	220	4.25	1.17	516	4.83	1.22	434	4.89	1.12	597	4.64	1.19	.64	.40	.072 (.070)
Communication with mother (1-6)	246	2.80	1.09	225	3.00	1.10	545	3.07	1.25	450	3.09	1.19	645	3.01	1.17	4.01*	.31	.009 (.007)
Communication with father (0-6)	270	3.55	1.46	241	3.66	1.35	608	3.71	1.67	495	3.78	1.61	703	3.73	1.50	2.04	.00	.009 (.007)
Peer pressure (1-5)	586	2.04	.75	534	2.06	.73	1120	1.88	.68	963	1.91	.68	1482	2.00	.73	1.92	.53	.012 (.009)
Physical peer victimisation (0-8)	513	.36	1.14	458	.45	1.24	1026	.12	.67	885	.13	.62	1264	.21	.77	.37	.27	.016 (.014)
Social peer victimisation (0-8)	513	.65	1.40	458	.78	1.62	1026	.73	1.60	885	.78	1.61	1264	.78	1.55	.50	1.95	.007 (.004)
Verbal peer victimisation (0-8)	513	2.36	2.71	457	2.51	2.73	1025	1.72	2.34	886	1.78	2.36	1264	2.28	2.60	.08	7.07*	.019 (.016)

Cyber peer victimisation (0-8)	513	.49	1.36	458	.63	1.51	1024	.57	1.42	886	.59	1.40	1263	.63	1.51	1.09	.55	.003 (.000)
Physical peer Perpetration (0-8)	513	.30	1.09	458	.36	1.15	1025	.06	.45	886	.06	.46	1263	.17	.75	.36	.68	.020 (.017)
Social peer Perpetration (0-8)	512	.41	.89	458	.45	.96	1024	.40	.80	886	.40	.79	1263	.44	.94	.55	.81	.002 (.000)
Verbal peer Perpetration (0-8)	513	1.97	2.53	458	2.10	2.41	1024	1.17	1.77	886	1.15	1.71	1263	1.75	2.24	.45	1.98	.027 (.024)
Cyber peer Perpetration (0-8)	513	.29	1.01	458	.27	.88	1024	.25	.82	886	.23	.80	1263	.32	1.04	1.04	.11	.001 (-.002)
Parental role aspirations (1-5)	525	4.04	.76	473	4.03	.79	1058	4.24	.76	911	4.21	.80	1317	4.16	.73	.01	.08	.008 (.005)
Occupational role aspirations (1-5)	525	3.95	.63	473	3.94	.60	1059	3.93	.63	912	3.97	.60	1318	3.94	.59	.04	.10	.008 (.006)
Homecare role aspirations (1-5)	525	3.93	.67	473	3.88	.63	1059	4.05	.62	912	4.04	.62	1318	3.99	.62	.32	.59	.004 (.002)
Importance of Relationships (0-20)	605	15.10	3.77	536	14.83	3.84	1146	16.43	3.22	980	16.60	3.17	1510	15.80	3.67	3.95*	4.36*	.066 (.064)
Achievement Motivation (0-16)	605	1.26	3.02	536	1.18	3.01	1146	1.24	2.79	980	1.19	2.77	1509	1.15	3.01	.58	.53	.006 (.003)
Purpose in Life (1-5)	605	3.45	.82	536	3.42	.81	1146	3.52	.79	979	3.48	.83	1508	3.44	.83	.01	.12	.000 (-.002)
Importance of democracy and equality (1-5)	605	4.15	.59	534	4.19	.55	1143	4.33	.51	976	4.33	.53	1506	4.28	.52	.21	.51	.028 (.025)
Environmental concerns (1-5)	606	3.61	.75	535	3.64	.73	1143	3.71	.66	978	3.74	.67	1508	3.71	.71	.23	.47	.003 (.000)
Religiosity (0-25)	589	4.11	4.98	534	3.99	4.94	1121	4.87	5.18	964	4.45	4.83	1482	4.13	4.82	4.57*	.29	.008 (.005)
Importance of leisure (1-5)	515	3.94	.70	463	3.93	.74	1035	3.76	.77	902	3.77	.78	1287	3.84	.74	.63	.57	.014 (.011)
Alcohol use (0-40)	483	8.46	5.21	433	8.95	5.31	953	7.38	4.66	814	7.55	4.83	1176	8.45	5.22	.75	.56	.026 (.023)
Ever smoked (0-1)	521	.54	.50	467	.57	.50	1048	.54	.50	894	.54	.50	1292	.59	.49	.06	.65	.001 (-.002)
Ever vaped (0-1)	521	.28	.45	464	.30	.46	1047	.24	.43	895	.23	.42	1291	.25	.43	.02	.03	.001 (-.002)
Cognitive enhancers (0-12)	515	1.28	2.13	461	1.53	2.21	1037	1.01	1.73	887	1.06	1.82	1276	1.26	2.04	2.27	.33	.004 (.001)
Big 5 Agreeableness (1-5)	590	3.56	.57	527	3.57	.58	1087	3.85	.52	936	3.80	.57	1488	3.69	.58	.06	.32	.075 (.072)

Big 5 Conscientiousness (1-5)	588	3.80	.65	527	3.80	.61	1086	3.97	.57	933	3.91	.63	1487	3.86	.62	.72	.04	.009 (.006)
Big 5 Extraversion (1-5)	592	3.45	.68	537	3.45	.68	1087	3.55	.64	942	3.56	.63	1490	3.51	.66	1.12	4.63*	.014 (.011)
Big 5 Neuroticism (1-5)	592	2.50	.67	539	2.46	.68	1089	2.69	.67	943	2.69	.68	1491	2.61	.64	.61	.03	.031 (.028)
Big 5 Openness (1-5)	592	3.38	.66	534	3.38	.61	1087	3.40	.60	940	3.41	.62	1489	3.41	.62	.16	5.12*	.006 (.003)
Self-control (0-24)	588	13.93	4.26	524	14.17	4.33	1086	14.95	4.08	933	14.72	4.10	1485	14.32	4.25	2.54	.04	.006 (.004)
Not planning for the future (1-5)	587	2.56	.91	523	2.58	.91	1086	2.39	.89	932	2.44	.92	1482	2.46	.93	.18	.77	.005 (.002)
Ambition (0-20)	528	14.08	3.72	474	13.88	3.57	1058	13.80	3.57	913	13.77	3.63	1312	13.86	3.62	2.66	1.45	.010 (.007)
Physical activity (1-5)	583	2.94	1.13	523	2.92	1.08	1131	2.73	1.08	958	2.75	1.09	1461	2.87	1.09	.03	.85	.009 (.006)
Health behaviours (0-48)	587	24.71	6.56	525	24.50	6.83	1133	25.48	6.61	964	25.48	6.74	1469	25.37	6.63	1.66	.00	.005 (.002)
Risk taking behaviour (0-24)	585	7.39	3.97	525	7.43	3.47	1085	5.82	3.39	933	5.93	3.41	1478	6.64	3.61	2.44	.17	.049 (.046)
Risky sexual behaviour (0-16)	573	3.98	2.79	513	3.88	2.65	1090	4.27	2.78	932	4.30	2.76	1419	4.20	2.71	.04	2.59	.010 (.008)
Media use (0-20)	584	12.10	3.63	523	11.87	3.62	1125	13.42	2.75	953	13.58	2.87	1458	13.01	3.27	.93	.68	.048 (.045)
Volunteering (0-20)	589	5.43	3.30	534	5.45	3.40	1123	5.71	3.21	964	5.70	3.16	1485	5.76	3.19	.03	.55	.001 (-.002)
Internet dating (scale) (0-5)	582	3.08	2.10	522	3.02	2.13	1121	2.47	2.17	950	2.60	2.21	1451	2.99	2.14	3.12	1.72	.013 (.010)
Internet dating (y/n) (0-1)	586	.52	.50	524	.51	.50	1124	.40	.49	954	.44	.50	1458	.50	.50	2.29	2.40	.011 (.008)
Financial literacy: Knowledge of Products (0-4)	586	1.56	.75	516	1.57	.71	1106	1.30	.64	931	1.28	.68	1430	1.42	.69	.00	.52	.067 (.064)
Financial attitudes and behaviour (1-5)	586	3.49	.64	516	3.49	.65	1105	3.63	.59	931	3.59	.60	1430	3.52	.64	.04	.89	.009 (.006)

Note. MZ=monozygotic; DZ=dizygotic. Raw uncorrected scores were used. These results are based on one twin randomly selected from each pair so that the data points are independent. Group difference = *F* statistics; **p < .05. **p < .001.

Table S6. Phenotypic correlations between psychological traits and composite scores of adverse physical and adverse mental health, wellbeing and educational attainment, for the whole sample (a) and then separately for males (b) and females (c) (95% confidence intervals are in parentheses).

(a)	All														
	Wellbeing			Adverse mental health			Adverse physical health			Achieved educational level			Planned educational level		
Trait	*r*	*p*	*n*	*r*	*p*	*n*	*r*	*p*	*n*	*r*	*p*	*n*	*r*	*p*	*n*
Daily hassles	-.29 (-.31,-.26)	<.001	3890	.52 (.49,.54)	<.001	3607	.28 (.25,.31)	<.001	3877	.01 (-.02,.04)	.573	3965	-.01 (-.05,.03)	.510	2656
CHAOS	-.29 (-.33, -.27)	<.001	4625	.32 (.29,.35)	<.001	3609	.22 (.18,.24)	<.001	3879	-.09 (-.12,-.06)	<.001	4728	-.08 (-.11,-.04)	<.001	3121
Childhood experiences	-.24 (-.27,-.21)	<.001	3885	.35 (.32,.37)	<.001	3591	.22 (.19,.25)	<.001	3854	-.04 (-.07,-.01)	.007	3959	-.06 (-.10,-.02)	.002	2654
Poor sleep quality	-.23 (-.26,-.20)	<.001	3832	.50 (.47,.52)	<.001	3590	.38 (.35,.41)	<.001	3875	-.04 (-.07,-.01)	.019	3908	-.03 (-.07,.01)	.160	2615
Marriage hopes	.14 (.11,.17)	<.001	4625	-.08 (-.11,-.05)	<.001	3610	-.05 (-.08,-.02)	.002	3879	.10 (.07,.13)	<.001	4727	.05 (.02,.09)	.005	3121
Marriage worries	-.28 (-.31,-.25)	<.001	4626	.22 (.18,.25)	<.001	3611	.12 (.09,.15)	<.001	3880	-.03 (-.06,.00)	.044	4728	-.05 (-.08,-.01)	.013	3121
Quality of relationship with twin	.18 (.15,.21)	<.001	4555	-.18 (-.21,-.14)	<.001	3571	-.09 (-.12,-.06)	<.001	3832	.03 (.00,.06)	.035	4658	.00 (-.03,.04)	.904	3080
Quality of relationship with mother	.21 (.18,.24)	<.001	4510	-.22 (-.25,-.18)	<.001	3538	-.08 (-.11,-.05)	<.001	3794	.00 (-.03,.03)	.830	4612	.00 (-.04,.03)	.872	3048
Quality of relationship with father	.25 (.22,.27)	<.001	4401	-.26 (-.29,-.23)	<.001	3465	-.14 (-.17,-.11)	<.001	3719	.04 (.01,.07)	.005	4497	.01 (-.03,.04)	.726	2969
Number of relationships	.06 (.04,.09)	<.001	4623	.11 (.07,.14)	<.001	3608	.16 (.13,.19)	<.001	3877	-.07 (-.09,-.04)	<.001	4726	-.06 (-.10,-.03)	.001	3119
Longest relationship	.17 (.14,.20)	<.001	3881	-.02 (-.06,.01)	.205	3005	.00 (-.03,.04)	.859	3232	-.07 (-.10,-.03)	<.001	3963	-.04 (-.08,.00)	.061	2602
Partner violence	-.21 (-.24,-.18)	<.001	4476	.32 (.29,.35)	<.001	3496	.22 (.19,.25)	<.001	3758	-.10 (-.12,-.07)	<.001	4573	-.07 (-.10,-.03)	<.001	3034
Contact with mother	.10 (.05,.14)	<.001	1798	-.02 (-.07,.03)	.372	1669	.03 (-.02,.07)	.271	1772	-.25 (-.29,-.20)	<.001	1817	-.19 (-.24,-.14)	<.001	1263
Communication with mother	.10 (.05,.14)	<.001	1831	-.09 (-.14,-.05)	<.001	1698	-.04 (-.08,.01)	.138	1803	-.04 (-.09,.00)	.066	1849	-.03 (-.08,.03)	.302	1283

Contact with father	.10 (.06,.14)	<.001	1957	-.08 (-.13,-.04)	<.001	1819	-.01 (-.06,.03)	.614	1934	-.14 (-.18,-.09)	<.001	1980	-.15 (-.20,-.10)	<.001	1340
Communication with father	.13 (.09,.18)	<.001	2138	-.16 (-.20,-.11)	<.001	1977	-.09 (-.13,-.04)	<.001	2107	.05 (.01,.09)	.024	2164	.02 (-.03,.07)	.416	1471
Peer pressure	-.20 (-.23,-.17)	<.001	4537	.27 (.23,.30)	<.001	3608	.15 (.11,.18)	<.001	3820	.02 (.00,.05)	.102	4642	-.02 (-.05,.02)	.338	3067
Physical peer victimisation	-.09 (-.13,-.06)	<.001	3781	.16 (.12,.19)	<.001	3535	.11 (.08,.15)	<.001	3792	-.04 (-.07,.00)	.028	3852	-.03 (-.07,.01)	.114	2584
Social peer victimisation	-.19 (-.22,-.16)	<.001	3780	.39 (.37,.42)	<.001	3534	.21 (.18,.24)	<.001	3791	-.03 (-.06,.00)	.084	3851	-.05 (-.09,-.01)	.013	2584
Verbal peer victimisation	-.18 (-.21,-.15)	<.001	3780	.38 (.35,.41)	<.001	3534	.22 (.18,.25)	<.001	3791	.00 (-.03,.03)	.870	3851	.00 (-.04,.04)	.876	2583
Cyber peer victimisation	-.17 (-.20,-.13)	<.001	3780	.35 (.32,.38)	<.001	3534	.23 (.20,.26)	<.001	3791	-.05 (-.08,-.01)	.005	3851	-.07 (-.11,-.03)	.001	2583
Physical peer Perpetration	-.06 (-.09,-.02)	.001	3781	.12 (.08,.15)	<.001	3537	.08 (.05,.11)	<.001	3793	-.07 (-.10,-.04)	<.001	3852	-.10 (-.14,-.06)	<.001	2584
Social peer Perpetration	-.16 (-.19,-.13)	<.001	3781	.30 (.26,.32)	<.001	3537	.15 (.12,.18)	<.001	3793	-.06 (-.09,-.03)	<.001	3852	-.06 (-.10,-.03)	.001	2584
Verbal peer Perpetration	-.10 (-.14,-.07)	<.001	3781	.22 (.19,.25)	<.001	3537	.13 (.10,.16)	<.001	3793	-.02 (-.05,.01)	.285	3852	-.02 (-.06,.01)	.226	2584
Cyber peer Perpetration	-.09 (-.12,-.06)	<.001	3781	.19 (.16,.22)	<.001	3537	.14 (.11,.17)	<.001	3793	-.05 (-.08,-.02)	.003	3852	-.08 (-.11,-.04)	<.001	2584
Parental role aspirations	.15 (.12,.18)	<.001	3897	-.14 (-.17,-.10)	<.001	3598	-.04 (-.07,-.01)	.009	3861	-.05 (-.08,-.01)	.004	3970	-.02 (-.06,.02)	.294	2661
Occupational role aspirations	.02 (-.01,.05)	.223	3897	-.05 (-.08,-.02)	.004	3598	-.03 (-.06,.00)	.046	3861	.12 (.09,.15)	<.001	3970	.07 (.04,.11)	<.001	2661
Homecare role aspirations	.19 (.16,.22)	<.001	3897	-.09 (-.12,-.06)	<.001	3598	-.04 (-.07,-.01)	.011	3861	-.05 (-.08,-.02)	.002	3970	-.05 (-.09,-.01)	.011	2661
Importance of Relationships	.18 (.15,.20)	<.001	4577	-.10 (-.13,-.07)	<.001	3590	-.01 (-.04,.02)	.549	3841	.04 (.01,.07)	.004	4681	.02 (-.02,.05)	.417	3094
Achievement Motivation	.09 (.06,.12)	<.001	4577	-.04 (-.08,-.01)	.010	3590	-.01 (-.04,.02)	.470	3841	.05 (.02,.07)	.002	4681	.04 (.00,.07)	.033	3094
Purpose in Life	.44 (.41,.46)	<.001	4576	-.56 (-.58,-.54)	<.001	3590	-.23 (-.26,-.20)	<.001	3841	.06 (.03,.09)	<.001	4680	.08 (.05,.12)	<.001	3094
Importance of democracy and equality	-.02 (-.05,.01)	.205	4580	.05 (.01,.08)	.007	3587	.00 (-.03,.03)	.988	3841	.16 (.13,.18)	<.001	4684	.11 (.08,.15)	<.001	3096

Environmental concerns	-.01 (-.04,.02)	.514	4581	-.02 (-.05,.02)	.322	3588	-.07 (-.10,-.04)	<.001	3842	.18 (.16,.21)	<.001	4685	.19 (.15,.22)	<.001	3096
Religiosity	.02 (-.01,.05)	.212	4543	.03 (.00,.06)	.095	3610	.02 (-.01,.05)	.229	3823	.06 (.03,.09)	<.001	4647	.07 (.03,.10)	<.001	3068
Importance of leisure	.32 (.29,.35)	<.001	3830	-.40 (-.43,-.37)	<.001	3610	-.22 (-.25,-.19)	<.001	3857	.11 (.08,.14)	<.001	3905	.10 (.06,.14)	<.001	2614
Alcohol use	-.11 (-.14,-.07)	<.001	3516	.14 (.11,.18)	<.001	3283	.10 (.07,.14)	<.001	3517	.03 (.00,.06)	.059	3580	.03 (-.01,.06)	.211	2430
Ever smoked	-.09 (-.12,-.06)	<.001	3834	.12 (.08,.15)	<.001	3574	.10 (.07,.13)	<.001	3837	-.07 (-.10,-.04)	<.001	3906	-.06 (-.10,-.02)	.002	2622
Ever vaped	-.10 (-.13,-.06)	<.001	3835	.16 (.12,.19)	<.001	3577	.17 (.13,.20)	<.001	3837	-.14 (-.17,-.10)	<.001	3907	-.11 (-.15,-.08)	<.001	2624
Cognitive enhancers	-.15 (-.18,-.12)	<.001	3809	.23 (.20,.26)	<.001	3550	.14 (.11,.17)	<.001	3816	.00 (-.03,.03)	.899	3877	.02 (-.02,.06)	.280	2609
Big 5 Agreeableness	.10 (.07,.13)	<.001	4360	-.10 (-.14,-.07)	<.001	3608	-.07 (-.10,-.03)	<.001	3665	.02 (-.01,.05)	.188	4449	.01 (-.03,.05)	.547	2961
Big 5 Conscientiousness	.26 (.23,.29)	<.001	4361	-.29 (-.32,-.26)	<.001	3608	-.16 (-.19,-.13)	<.001	3665	.09 (.06,.12)	<.001	4450	.08 (.04,.11)	<.001	2961
Big 5 Extraversion	.27 (.25,.30)	<.001	4360	-.41 (-.44,-.39)	<.001	3608	-.16 (-.19,-.13)	<.001	3665	.07 (.04,.10)	<.001	4449	.03 (-.01,.07)	.101	2961
Big 5 Neuroticism	-.36 (-.38,-.33)	<.001	4361	.65 (.63,.67)	<.001	3608	.30 (.27,.33)	<.001	3665	-.04 (-.06,-.01)	.020	4450	-.03 (-.07,.00)	.067	2961
Big 5 Openness	-.14 (-.17,-.11)	<.001	4360	.13 (.09,.16)	<.001	3608	.04 (.01,.07)	.012	3665	.07 (.04,.10)	<.001	4449	.02 (-.02,.05)	.358	2961
Self-control	.28 (.25,.31)	<.001	4361	-.45 (-.48,-.43)	<.001	3608	-.25 (-.28,-.22)	<.001	3665	.07 (.04,.10)	<.001	4450	.07 (.03,.10)	<.001	2961
Not planning for the future	-.16 (-.19,-.13)	<.001	4361	.17 (.14,.20)	<.001	3608	.08 (.05,.12)	<.001	3665	-.10 (-.13,-.07)	<.001	4450	-.11 (-.15,-.08)	<.001	2961
Ambition	.14 (.11,.17)	<.001	3890	-.15 (-.18,-.12)	<.001	3607	-.07 (-.10,-.04)	<.001	3877	.11 (.08,.14)	<.001	3965	.09 (.06,.13)	<.001	2656
Physical activity	.15 (.12,.18)	<.001	4616	-.15 (-.18,-.11)	<.001	3606	-.14 (-.17,-.11)	<.001	3874	.10 (.07,.13)	<.001	4720	.07 (.04,.11)	<.001	3118
Health behaviours	.11 (.08,.14)	<.001	4623	-.19 (-.22,-.16)	<.001	3610	-.15 (-.18,-.11)	<.001	3881	.17 (.14,.19)	<.001	4728	.15 (.12,.19)	<.001	3122
Risk taking behaviour	-.12 (-.15,-.09)	<.001	4363	.12 (.09,.15)	<.001	3611	.14 (.11,.17)	<.001	3665	.01 (-.02,.04)	.429	4452	-.01 (-.05,.02)	.460	2958
Risky sexual behaviour	.06 (.03,.09)	<.001	4386	.04 (.01,.08)	.010	3445	.10 (.06,.13)	<.001	3690	-.08 (-.11,-.05)	<.001	4487	-.05 (-.09,-.02)	.004	2970

Media use	.00 (-.03,.03)	.925	4605	.04 (.00,.07)	.025	3587	.08 (.05,.11)	<.001	3851	.00 (-.03,.03)	.982	4696	-.01 (-.04,.03)	.684	3105
Volunteering	.06 (.04,.09)	<.001	4543	.03 (.00,.07)	.048	3611	.09 (.06,.12)	<.001	3825	.11 (.08,.13)	<.001	4647	.09 (.05,.12)	<.001	3070
Internet dating (scale)	-.18 (-.21,-.15)	<.001	4587	.11 (.07,.14)	<.001	3578	.09 (.06,.13)	<.001	3844	.08 (.06,.11)	<.001	4683	.03 (-.01,.06)	.121	3102
Internet dating (y/n)	-.15 (-.18,-.12)	<.001	4597	.10 (.07,.14)	<.001	3586	.10 (.07,.13)	<.001	3852	.07 (.04,.10)	<.001	4694	.02 (-.02,.05)	.301	3105
Financial literacy: Knowledge of Products	.22 (.20,.25)	<.001	4626	-.14 (-.17,-.11)	<.001	3535	-.04 (-.07,-.01)	.017	3789	.02 (-.01,.05)	.239	4616	.01 (-.02,.05)	.548	3059
Financial attitudes and behaviour	.32 (.29,.34)	<.001	4625	-.19 (-.22,-.16)	<.001	3534	-.14 (-.18,-.11)	<.001	3788	.07 (.04,.09)	<.001	4615	.06 (.02,.09)	.002	3058

(b)	Males														
	Wellbeing			Adverse mental health			Adverse physical health			Achieved educational level			Planned educational level		
Trait	*r*	*p*	*n*	*r*	*p*	*n*	*r*	*p*	*n*	*r*	*p*	*n*	*r*	*p*	*n*
Daily hassles	-.29 (-.34,-.25)	<.001	1424	.51 (.47,.55)	<.001	1312	.28 (.23,.32)	<.001	1395	-.02 (-.07,.03)	.563	1439	-.06 (-.12,.00)	.050	939
CHAOS	-.26 (-.30,-.21)	<.001	1774	.28 (.23,.33)	<.001	1312	.17 (.12,.22)	<.001	1395	-.11 (-.16,-.06)	<.001	1798	-.08 (-.14,-.02)	.006	1149
Childhood experiences	-.22 (-.27,-.17)	<.001	1418	.32 (.27,.37)	<.001	1305	.17 (.12,.22)	<.001	1383	-.07 (-.12,-.02)	.013	1434	-.10 (-.16,-.04)	.003	936
Poor sleep quality	-.22 (-.27,-.17)	<.001	1401	.45 (.40,.49)	<.001	1303	.32 (.28,.37)	<.001	1394	-.05 (-.10,.00)	.083	1416	-.06 (-.12,.00)	.075	924
Marriage hopes	.12 (.08,.17)	<.001	1774	-.08 (-.14,-.03)	.003	1312	-.02 (-.07,.04)	.576	1395	.11 (.06,.16)	<.001	1798	.02 (-.04,.08)	.442	1149
Marriage worries	-.20 (-.24,-.15)	<.001	1774	.16 (.10,.21)	<.001	1312	.05 (.00,.11)	.047	1395	-.02 (-.07,.03)	.375	1798	-.06 (-.12,.00)	.044	1149
Quality of relationship with twin	.16 (.11,.20)	<.001	1740	-.09 (-.14,-.03)	.001	1297	-.08 (-.13,-.03)	.003	1373	.03 (-.02,.08)	.173	1764	-.03 (-.09,.03)	.364	1129
Quality of relationship with mother	.17 (.12,.22)	<.001	1722	-.13 (-.19,-.08)	<.001	1282	-.09 (-.14,-.03)	.002	1357	.03 (-.02,.08)	.236	1746	-.03 (-.09,.03)	.369	1116
Quality of relationship with father	.27 (.23,.31)	<.001	1676	-.25 (-.30,-.20)	<.001	1257	-.14 (-.19,-.09)	<.001	1330	.05 (.00,.10)	.050	1698	.03 (-.03,.09)	.409	1086

Number of relationships	.10 (.06,.15)	<.001	1772	.04 (-.02,.09)	.173	1310	.11 (.06,.16)	<.001	1393	-.06 (-.11,-.01)	.013	1796	-.09 (-.15,-.03)	.002	1148
Longest relationship	.16 (.11,.21)	<.001	1468	-.06 (-.12,.00)	.049	1070	-.02 (-.08,.04)	.568	1137	-.03 (-.08,.02)	.192	1485	.03 (-.03,.09)	.439	955
Partner violence	-.19 (-.23,-.14)	<.001	1700	.28 (.23,.33)	<.001	1255	.14 (.09,.19)	<.001	1334	-.10 (-.15,-.05)	<.001	1720	-.07 (-.13,-.01)	.015	1108
Contact with mother	.11 (.03,.19)	.005	634	.00 (-.08,.08)	.971	591	.00 (-.08,.08)	.997	614	-.21 (-.28,-.13)	<.001	637	-.12 (-.21,-.03)	.015	435
Communication with mother	.07 (-.01,.15)	.072	645	-.02 (-.10,.06)	.633	601	-.05 (-.13,.03)	.236	625	-.04 (-.12,.04)	.350	648	.00 (-.09,.09)	.942	443
Contact with father	.11 (.04,.19)	.003	689	-.10 (-.17,-.02)	.015	642	-.03 (-.10,.05)	.496	669	-.10 (-.17,-.03)	.010	693	-.11 (-.20,-.02)	.025	458
Communication with father	.16 (.09,.23)	<.001	750	-.18 (-.25,-.11)	<.001	694	-.04 (-.11,.03)	.256	727	.07 (.00,.14)	.073	754	-.02 (-.11,.07)	.657	500
Peer pressure	-.17 (-.21,-.12)	<.001	1740	.23 (.18,.28)	<.001	1311	.15 (.10,.20)	<.001	1375	-.01 (-.06,.04)	.609	1766	-.04 (-.10,.02)	.141	1128
Physical peer victimisation	-.10 (-.15,-.05)	<.001	1388	.17 (.12,.22)	<.001	1291	.11 (.05,.16)	<.001	1369	-.02 (-.07,.03)	.449	1402	-.05 (-.11,.01)	.165	917
Social peer victimisation	-.17 (-.22,-.12)	<.001	1388	.35 (.30,.40)	<.001	1291	.16 (.11,.21)	<.001	1369	.01 (-.04,.06)	.840	1402	-.05 (-.11,.01)	.128	917
Verbal peer victimisation	-.13 (-.18,-.07)	<.001	1387	.28 (.23,.33)	<.001	1290	.13 (.08,.18)	<.001	1368	.04 (-.01,.09)	.145	1401	.01 (-.05,.07)	.766	916
Cyber peer victimisation	-.14 (-.19,-.09)	<.001	1387	.27 (.22,.32)	<.001	1290	.12 (.07,.17)	<.001	1368	.01 (-.04,.06)	.681	1401	-.05 (-.11,.01)	.146	916
Physical peer Perpetration	-.07 (-.12,-.01)	.012	1386	.13 (.07,.18)	<.001	1291	.08 (.03,.13)	.002	1368	-.07 (-.12,-.02)	.006	1400	-.14 (-.20,-.08)	<.001	916
Social peer Perpetration	-.16 (-.21,-.11)	<.001	1386	.31 (.26,.36)	<.001	1291	.13 (.07,.18)	<.001	1368	-.04 (-.09,.01)	.120	1400	-.07 (-.13,-.01)	.051	916
Verbal peer Perpetration	-.08 (-.13,-.02)	.004	1386	.17 (.12,.22)	<.001	1291	.10 (.05,.15)	<.001	1368	.02 (-.03,.07)	.572	1400	.00 (-.06,.06)	.955	916
Cyber peer Perpetration	-.08 (-.14,-.03)	.002	1386	.17 (.11,.22)	<.001	1291	.10 (.04,.15)	<.001	1368	-.03 (-.08,.02)	.277	1400	-.06 (-.12,.00)	.097	916
Parental role aspirations	.14 (.09,.19)	<.001	1423	-.13 (-.18,-.08)	<.001	1309	-.05 (-.10,.01)	.088	1387	.01 (-.04,.06)	.781	1438	-.01 (-.07,.05)	.676	938
Occupational role aspirations	.02 (-.03,.07)	.464	1423	-.09 (-.15,-.04)	.001	1309	-.05 (-.10,.01)	.082	1387	.09 (.04,.14)	<.001	1438	.08 (.02,.14)	.017	938
Homecare role aspirations	.20 (.15,.25)	<.001	1423	-.08 (-.13,-.02)	.005	1309	-.07 (-.12,-.02)	.010	1387	-.02 (-.07,.03)	.398	1438	-.02 (-.08,.04)	.630	938

Importance of Relationships	.15 (.10,.20)	<.001	1755	-.04 (-.10,.01)	.143	1305	.03 (-.02,.09)	.215	1380	.06 (.01,.11)	.012	1780	.03 (-.03,.09)	.253	1139
Achievement Motivation	.10 (.05,.14)	<.001	1755	-.03 (-.09,.02)	.259	1305	.01 (-.04,.06)	.705	1380	.07 (.02,.12)	.005	1780	.04 (-.02,.10)	.182	1139
Purpose in Life	.43 (.39,.46)	<.001	1755	-.50 (-.54,-.46)	<.001	1305	-.20 (-.25,-.15)	<.001	1380	.07 (.02,.12)	.002	1780	.12 (.06,.18)	<.001	1139
Importance of democracy and equality	-.01 (-.05,.04)	.748	1755	.04 (-.02,.09)	.189	1303	.02 (-.03,.07)	.465	1379	.12 (.07,.17)	<.001	1780	.07 (.01,.13)	.013	1140
Environmental concerns	.00 (-.04,.05)	.913	1756	-.03 (-.09,.02)	.256	1304	-.06 (-.11,.00)	.035	1380	.19 (.14,.23)	<.001	1781	.22 (.16,.27)	<.001	1140
Religiosity	.04 (-.01,.08)	.110	1744	-.01 (-.06,.05)	.756	1312	.01 (-.04,.06)	.728	1377	.04 (-.01,.09)	.133	1769	.04 (-.02,.10)	.203	1128
Importance of leisure	.32 (.27,.36)	<.001	1394	-.39 (-.43,-.34)	<.001	1312	-.19 (-.24,-.13)	<.001	1380	.10 (.05,.15)	<.001	1408	.11 (.05,.17)	.001	919
Alcohol use	-.08 (-.13,-.02)	.004	1297	.11 (.05,.16)	<.001	1209	.10 (.04,.15)	<.001	1279	.06 (.01,.11)	.037	1313	.04 (-.03,.11)	.197	863
Ever smoked	-.06 (-.11,.00)	.041	1400	.09 (.03,.14)	.002	1298	.10 (.05,.16)	<.001	1380	-.06 (-.11,-.01)	.016	1415	-.06 (-.12,.00)	.056	929
Ever vaped	-.06 (-.11,-.01)	.019	1400	.13 (.07,.18)	<.001	1300	.15 (.09,.20)	<.001	1378	-.13 (-.18,-.08)	<.001	1415	-.13 (-.19,-.07)	<.001	927
Cognitive enhancers	-.10 (-.16,-.05)	<.001	1387	.15 (.09,.20)	<.001	1285	.12 (.07,.17)	<.001	1369	.00 (-.05,.05)	.929	1400	.04 (-.02,.10)	.274	921
Big 5 Agreeableness	.06 (.01,.11)	.013	1677	-.03 (-.08,.03)	.368	1312	-.07 (-.12,-.01)	.015	1325	.05 (.00,.10)	.065	1702	.00 (-.06,.06)	.996	1095
Big 5 Conscientiousness	.26 (.21,.30)	<.001	1677	-.29 (-.34,-.24)	<.001	1312	-.15 (-.20,-.10)	<.001	1325	.09 (.04,.14)	<.001	1702	.05 (-.01,.11)	.091	1095
Big 5 Extraversion	.25 (.21,.30)	<.001	1677	-.35 (-.40,-.31)	<.001	1312	-.13 (-.18,-.07)	<.001	1325	.04 (-.01,.09)	.093	1702	-.01 (-.07,.05)	.695	1095
Big 5 Neuroticism	-.34 (-.39,-.30)	<.001	1677	.61 (.57,.64)	<.001	1312	.31 (.26,.35)	<.001	1325	-.08 (-.13,-.03)	.002	1702	-.05 (-.11,.01)	.086	1095
Big 5 Openness	-.15 (-.20,-.10)	<.001	1677	.17 (.12,.22)	<.001	1312	.06 (.01,.12)	.024	1325	.07 (.02,.12)	.006	1702	.02 (-.04,.08)	.538	1095
Self-control	.28 (.23,.32)	<.001	1677	-.46 (-.50,-.41)	<.001	1312	-.22 (-.27,-.17)	<.001	1325	.08 (.03,.13)	.001	1702	.09 (.03,.15)	.004	1095
Not planning for the future	-.16 (-.21,-.12)	<.001	1677	.20 (.15,.26)	<.001	1312	.10 (.05,.16)	<.001	1325	-.10 (-.15,-.05)	<.001	1702	-.10 (-.16,-.04)	.001	1095

Ambition	.16 (.11,.21)	<.001	1424	-.21 (-.26,-.15)	<.001	1312	-.09 (-.14,-.04)	.001	1395	.09 (.04,.14)	.001	1439	.09 (.03,.15)	.009	939
Physical activity	.20 (.15,.24)	<.001	1767	-.20 (-.25,-.15)	<.001	1309	-.16 (-.21,-.11)	<.001	1390	.08 (.03,.13)	.002	1791	.04 (-.02,.10)	.142	1147
Health behaviours	.09 (.05,.14)	<.001	1771	-.16 (-.21,-.10)	<.001	1311	-.14 (-.19,-.08)	<.001	1395	.15 (.10,.19)	<.001	1796	.14 (.08,.20)	<.001	1149
Risk taking behaviour	-.08 (-.13,-.04)	.001	1677	.08 (.02,.13)	.005	1312	.17 (.12,.23)	<.001	1324	-.01 (-.06,.04)	.722	1702	-.06 (-.12,.00)	.057	1093
Risky sexual behaviour	.09 (.04,.13)	<.001	1682	.03 (-.02,.09)	.261	1252	.11 (.05,.16)	<.001	1329	-.08 (-.13,-.03)	.001	1708	-.05 (-.11,.01)	.088	1094
Media use	.02 (-.02,.07)	.305	1763	-.01 (-.06,.05)	.852	1301	.06 (.00,.11)	.037	1382	.04 (-.01,.09)	.125	1785	.02 (-.04,.08)	.456	1142
Volunteering	.08 (.03,.12)	.001	1744	-.03 (-.08,.03)	.336	1312	.06 (.00,.11)	.037	1377	.09 (.04,.14)	<.001	1769	.03 (-.03,.09)	.252	1128
Internet dating (scale)	-.15 (-.20,-.10)	<.001	1756	.10 (.04,.15)	.001	1298	.03 (-.02,.08)	.257	1379	.10 (.05,.15)	<.001	1779	-.01 (-.07,.05)	.840	1141
Internet dating (y/n)	-.11 (-.16,-.06)	<.001	1760	.08 (.03,.14)	.003	1301	.03 (-.02,.08)	.276	1382	.08 (.03,.13)	.001	1784	-.02 (-.08,.04)	.554	1142
Financial literacy: Knowledge of Products	.23 (.19,.28)	<.001	1774	-.15 (-.20,-.10)	<.001	1293	-.05 (-.11,.00)	.046	1374	.05 (.00,.10)	.044	1769	.04 (-.02,.10)	.181	1137
Financial attitudes and behaviour	.31 (.27,.36)	<.001	1774	-.22 (-.27,-.17)	<.001	1293	-.18 (-.23,-.13)	<.001	1374	.06 (.01,.11)	.010	1769	.08 (.02,.14)	.006	1137
(c)	Females														
	Wellbeing			Adverse mental health			Adverse physical health			Achieved educational level			Planned educational level		
Trait	*r*	*p*	*n*	*r*	*p*	*n*	*r*	*p*	*n*	*r*	*p*	*n*	*r*	*p*	*n*
Daily hassles	-.28 (-.32,-.24)	<.001	2466	.52 (.49,.55)	<.001	2295	.28 (.24,.32)	<.001	2482	.02 (-.02,.06)	.239	2526	.02 (-.03,.07)	.535	1717
CHAOS	-.33 (-.36,-.29)	<.001	2851	.34 (.31,.38)	<.001	2297	.23 (.19,.27)	<.001	2484	-.08 (-.12,-.04)	<.001	2930	-.08 (-.12,-.04)	.001	1972
Childhood experiences	-.25 (-.28,-.21)	<.001	2467	.36 (.32,.39)	<.001	2286	.23 (.20,.27)	<.001	2471	-.03 (-.07,.01)	.113	2525	-.05 (-.10,.00)	.058	1718
Poor sleep quality	-.24 (-.27,-.20)	<.001	2431	.52 (.49,.55)	<.001	2287	.40 (.37,.44)	<.001	2481	-.03 (-.07,.01)	.098	2492	-.01 (-.06,.04)	.614	1691
Marriage hopes	.15 (.11,.18)	<.001	2851	-.08 (-.12,-.04)	<.001	2298	-.07 (-.10,-.03)	.001	2484	.10 (.06,.14)	<.001	2929	.07 (.03,.11)	.003	1972

Marriage worries	-.33 (-.37,-.30)	<.001	2852	.25 (.21,.28)	<.001	2299	.16 (.12,.19)	<.001	2485	-.04 (-.08,.00)	.059	2930	-.04 (-.08,.00)	.114	1972
Quality of relationship with twin	.19 (.16,.23)	<.001	2815	-.22 (-.26,-.18)	<.001	2274	-.09 (-.13,-.05)	<.001	2459	.03 (-.01,.07)	.107	2894	.02 (-.02,.06)	.374	1951
Quality of relationship with mother	.24 (.20,.27)	<.001	2788	-.25 (-.29,-.21)	<.001	2256	-.08 (-.12,-.04)	<.001	2437	-.01 (-.05,.03)	.551	2866	.01 (-.03,.05)	.668	1932
Quality of relationship with father	.23 (.19,.27)	<.001	2725	-.26 (-.30,-.22)	<.001	2208	-.14 (-.18,-.10)	<.001	2389	.04 (.00,.08)	.039	2799	.00 (-.05,.05)	.873	1883
Number of relationships	.04 (.00,.07)	.047	2851	.14 (.10,.18)	<.001	2298	.19 (.15,.22)	<.001	2484	-.07 (-.11,-.03)	<.001	2930	-.04 (-.08,.00)	.076	1971
Longest relationship	.17 (.14,.21)	<.001	2413	-.01 (-.05,.04)	.820	1935	.01 (-.03,.05)	.572	2095	-.09 (-.13,-.05)	<.001	2478	-.08 (-.13,-.03)	.002	1647
Partner violence	-.22 (-.26,-.19)	<.001	2776	.33 (.29,.37)	<.001	2241	.25 (.21,.29)	<.001	2424	-.10 (-.14,-.06)	<.001	2853	-.07 (-.11,-.03)	.004	1926
Contact with mother	.09 (.03,.14)	.003	1164	-.03 (-.09,.03)	.323	1078	.04 (-.02,.09)	.216	1158	-.27 (-.32,-.22)	<.001	1180	-.23 (-.29,-.16)	<.001	828
Communication with mother	.11 (.05,.17)	<.001	1186	-.13 (-.18,-.07)	<.001	1097	-.03 (-.09,.03)	.311	1178	-.05 (-.11,.01)	.122	1201	-.04 (-.11,.03)	.237	840
Contact with father	.10 (.04,.15)	.001	1268	-.08 (-.13,-.02)	.009	1177	-.01 (-.06,.05)	.859	1265	-.16 (-.21,-.11)	<.001	1287	-.17 (-.23,-.11)	<.001	882
Communication with father	.12 (.07,.17)	<.001	1388	-.15 (-.20,-.09)	<.001	1283	-.10 (-.15,-.05)	<.001	1380	.04 (-.01,.09)	.124	1410	.04 (-.02,.10)	.190	971
Peer pressure	-.22 (-.26,-.19)	<.001	2797	.29 (.25,.32)	<.001	2297	.14 (.11,.18)	<.001	2445	.05 (.01,.09)	.009	2876	.00 (-.04,.04)	1<.001	1939
Physical peer victimisation	-.10 (-.13,-.06)	<.001	2393	.16 (.12,.20)	<.001	2244	.14 (.10,.17)	<.001	2423	-.05 (-.09,-.01)	.009	2450	-.02 (-.07,.03)	.428	1667
Social peer victimisation	-.20 (-.24,-.16)	<.001	2392	.41 (.38,.45)	<.001	2243	.23 (.19,.26)	<.001	2422	-.05 (-.09,-.01)	.022	2449	-.05 (-.10,.00)	.047	1667
Verbal peer victimisation	-.22 (-.25,-.18)	<.001	2393	.44 (.40,.47)	<.001	2244	.26 (.23,.30)	<.001	2423	-.03 (-.07,.01)	.127	2450	-.01 (-.06,.04)	.632	1667
Cyber peer victimisation	-.18 (-.22,-.14)	<.001	2393	.39 (.35,.42)	<.001	2244	.27 (.23,.31)	<.001	2423	-.08 (-.12,-.04)	<.001	2450	-.08 (-.13,-.03)	.002	1667
Physical peer Perpetration	-.05 (-.09,-.01)	.013	2395	.14 (.10,.18)	<.001	2246	.10 (.06,.14)	<.001	2425	-.08 (-.12,-.04)	<.001	2452	-.06 (-.11,-.01)	.010	1668
Social peer Perpetration	-.16 (-.20,-.12)	<.001	2395	.29 (.25,.33)	<.001	2246	.17 (.13,.21)	<.001	2425	-.07 (-.11,-.03)	.001	2452	-.06 (-.11,-.01)	.010	1668

Verbal peer Perpetration	-.13 (-.17,-.09)	<.001	2395	.26 (.22,.30)	<.001	2246	.16 (.12,.19)	<.001	2425	-.04 (-.08,.00)	.030	2452	-.04 (-.09,.01)	.088	1668
Cyber peer Perpetration	-.10 (-.13,-.06)	<.001	2395	.21 (.17,.25)	<.001	2246	.17 (.13,.21)	<.001	2425	-.06 (-.10,-.02)	.003	2452	-.09 (-.14,-.04)	<.001	1668
Parental role aspirations	.15 (.11,.19)	<.001	2474	-.14 (-.18,-.10)	<.001	2289	-.04 (-.08,.00)	.045	2474	-.08 (-.12,-.04)	<.001	2532	-.03 (-.08,.02)	.309	1723
Occupational role aspirations	.02 (-.02,.06)	.337	2474	-.03 (-.07,.02)	.210	2289	-.03 (-.06,.01)	.208	2474	.14 (.10,.18)	<.001	2532	.07 (.02,.12)	.003	1723
Homecare role aspirations	.18 (.14,.22)	<.001	2474	-.09 (-.13,-.05)	<.001	2289	-.03 (-.07,.01)	.202	2474	-.07 (-.11,-.03)	.001	2532	-.07 (-.12,-.02)	.004	1723
Importance of Relationships	.20 (.16,.23)	<.001	2822	-.14 (-.18,-.09)	<.001	2285	-.03 (-.07,.01)	.106	2461	.03 (-.01,.07)	.135	2901	.00 (-.04,.04)	.990	1955
Achievement Motivation	.09 (.05,.12)	<.001	2822	-.05 (-.09,-.01)	.018	2285	-.02 (-.06,.02)	.296	2461	.03 (-.01,.07)	.095	2901	.04 (.00,.08)	.096	1955
Purpose in Life	.44 (.41,.47)	<.001	2821	-.59 (-.61,-.56)	<.001	2285	-.25 (-.28,-.21)	<.001	2461	.05 (.01,.09)	.006	2900	.06 (.02,.10)	.007	1955
Importance of democracy and equality	-.03 (-.06,.01)	.161	2825	.05 (.01,.09)	.018	2284	-.01 (-.05,.03)	.567	2462	.18 (.14,.22)	<.001	2904	.14 (.10,.18)	<.001	1956
Environmental concerns	-.02 (-.05,.02)	.352	2825	-.01 (-.05,.03)	.650	2284	-.08 (-.12,-.04)	<.001	2462	.18 (.14,.22)	<.001	2904	.17 (.13,.21)	<.001	1956
Religiosity	.01 (-.03,.04)	.721	2799	.05 (.00,.09)	.030	2298	.02 (-.02,.06)	.242	2446	.07 (.03,.11)	<.001	2878	.08 (.04,.12)	<.001	1940
Importance of leisure	.32 (.28,.35)	<.001	2436	-.41 (-.44,-.37)	<.001	2298	-.24 (-.27,-.20)	<.001	2477	.12 (.08,.16)	<.001	2497	.10 (.05,.15)	<.001	1695
Alcohol use	-.13 (-.17,-.08)	<.001	2219	.16 (.12,.21)	<.001	2074	.11 (.07,.15)	<.001	2238	.01 (-.03,.05)	.514	2267	.02 (-.03,.07)	.557	1567
Ever smoked	-.11 (-.15,-.07)	<.001	2434	.13 (.09,.17)	<.001	2276	.10 (.06,.14)	<.001	2457	-.07 (-.11,-.03)	.001	2491	-.06 (-.11,-.01)	.014	1693
Ever vaped	-.12 (-.16,-.08)	<.001	2435	.18 (.13,.21)	<.001	2277	.18 (.14,.22)	<.001	2459	-.14 (-.18,-.10)	<.001	2492	-.10 (-.15,-.05)	<.001	1697
Cognitive enhancers	-.18 (-.22,-.14)	<.001	2422	.28 (.24,.32)	<.001	2265	.15 (.11,.19)	<.001	2447	-.01 (-.05,.03)	.799	2477	.01 (-.04,.06)	.624	1688
Big 5 Agreeableness	.12 (.08,.16)	<.001	2683	-.15 (-.18,-.10)	<.001	2296	-.07 (-.11,-.02)	.002	2340	.00 (-.04,.04)	.928	2747	.02 (-.03,.07)	.421	1866
Big 5 Conscientiousness	.27 (.23,.30)	<.001	2684	-.29 (-.33,-.25)	<.001	2296	-.17 (-.21,-.13)	<.001	2340	.09 (.05,.13)	<.001	2748	.09 (.04,.13)	<.001	1866

Big 5 Extraversion	.29 (.25,.32)	<.001	2683	-.45 (-.48,-.42)	<.001	2296	-.17 (-.21,-.13)	<.001	2340	.09 (.05,.13)	<.001	2747	.06 (.01,.11)	.012	1866
Big 5 Neuroticism	-.36 (-.39,-.33)	<.001	2684	.67 (.64,.69)	<.001	2296	.30 (.27,.34)	<.001	2340	-.01 (-.05,.03)	.626	2748	-.02 (-.07,.03)	.311	1866
Big 5 Openness	-.13 (-.17,-.10)	<.001	2683	.10 (.06,.14)	<.001	2296	.03 (-.01,.07)	.120	2340	.07 (.03,.11)	<.001	2747	.02 (-.03,.07)	.493	1866
Self-control	.29 (.25,.32)	<.001	2684	-.45 (-.48,-.42)	<.001	2296	-.26 (-.30,-.23)	<.001	2340	.07 (.03,.11)	<.001	2748	.05 (.00,.10)	.031	1866
Not planning for the future	-.16 (-.20,-.13)	<.001	2684	.16 (.11,.19)	<.001	2296	.07 (.03,.11)	<.001	2340	-.10 (-.14,-.06)	<.001	2748	-.12 (-.16,-.08)	<.001	1866
Ambition	.13 (.10,.17)	<.001	2466	-.12 (-.16,-.08)	<.001	2295	-.06 (-.10,-.02)	.001	2482	.12 (.08,.16)	<.001	2526	.10 (.05,.15)	<.001	1717
Physical activity	.12 (.08,.16)	<.001	2849	-.12 (-.16,-.08)	<.001	2297	-.13 (-.17,-.10)	<.001	2484	.12 (.08,.16)	<.001	2929	.09 (.05,.13)	<.001	1971
Health behaviours	.12 (.08,.15)	<.001	2852	-.21 (-.24,-.17)	<.001	2299	-.15 (-.19,-.11)	<.001	2486	.17 (.13,.20)	<.001	2932	.16 (.12,.20)	<.001	1973
Risk taking behaviour	-.14 (-.18,-.11)	<.001	2686	.15 (.11,.19)	<.001	2299	.13 (.09,.17)	<.001	2341	.03 (-.01,.07)	.150	2750	.02 (-.03,.07)	.439	1865
Risky sexual behaviour	.04 (.01,.08)	.025	2704	.05 (.01,.09)	.019	2193	.10 (.06,.14)	<.001	2361	-.08 (-.12,-.04)	<.001	2779	-.05 (-.10,.00)	.021	1876
Media use	-.02 (-.06,.02)	.267	2842	.06 (.02,.10)	.003	2286	.10 (.06,.14)	<.001	2469	-.03 (-.07,.01)	.118	2911	-.03 (-.07,.01)	.196	1963
Volunteering	.06 (.02,.09)	.003	2799	.06 (.02,.10)	.003	2299	.10 (.06,.14)	<.001	2448	.12 (.08,.16)	<.001	2878	.12 (.08,.16)	<.001	1942
Internet dating (scale)	-.20 (-.23,-.16)	<.001	2831	.11 (.07,.15)	<.001	2280	.12 (.08,.16)	<.001	2465	.08 (.04,.12)	<.001	2904	.05 (.01,.09)	.035	1961
Internet dating (y/n)	-.18 (-.21,-.14)	<.001	2837	.12 (.08,.16)	<.001	2285	.13 (.09,.17)	<.001	2470	.06 (.02,.10)	.001	2910	.04 (.00,.08)	.071	1963
Financial literacy: Knowledge of Products	.22 (.18,.25)	<.001	2852	-.13 (-.17,-.09)	<.001	2242	-.03 (-.07,.01)	.126	2415	-.01 (-.05,.03)	.773	2847	-.01 (-.05,.03)	.679	1922
Financial attitudes and behaviour	.32 (.29,.35)	<.001	2851	-.17 (-.21,-.13)	<.001	2241	-.13 (-.17,-.09)	<.001	2414	.07 (.03,.11)	<.001	2846	.04 (.00,.08)	.078	1921

Note. Scores were corrected for mean age and sex differences (see Methods). These results are based on one twin randomly selected from each pair so that the data points are independent.

Table S7. Summary of multiple regression analyses: variance explained in composite scores of adverse physical and adverse mental health, wellbeing and educational attainment psychological traits, for the whole sample (a) and then separately for males (b) and females (c) (95% confidence intervals are in parentheses).

(a)	All																								
	Wellbeing					Adverse mental health					Adverse physical health					Achieved educational level					Planned educational level				
Trait	*B*	*SE B*	*β*	sr^2	*p*	*B*	*SE B*	*β*	sr^2	*p*	*B*	*SE B*	*β*	sr^2	*p*	*B*	*SE B*	*β*	sr^2	*p*	*B*	*SE B*	*β*	sr^2	*p*
Daily hassles	-.05 (-.11, .01)	.03	-.05	.001	.106	.14 (.10, .18)	.02	.14	.013	.000	.07 (.00, .13)	.03	.06	.003	.048	.00 (-.06, .07)	.03	.00	.000	.910	-.02 (-.09, .06)	.04	-.02	.000	.683
CHAOS	-.12 (-.17, -.07)	.03	-.12	.011	.000	.07 (.03, .10)	.02	.07	.003	.001	.11 (.06, .17)	.03	.11	.010	.000	-.03 (-.08, .03)	.03	-.03	.001	.388	-.01 (-.07, .06)	.03	-.01	.000	.879
Childhood experiences	-.05 (-.11, .01)	.03	-.05	.002	.072	.06 (.02, .10)	.02	.06	.003	.002	.03 (-.03, .09)	.03	.03	.001	.292	.01 (-.05, .07)	.03	.01	.000	.727	.04 (-.03, .11)	.04	.04	.001	.281
Poor sleep quality	-.04 (-.10, .02)	.03	-.04	.001	.162	.17 (.13, .21)	.02	.17	.021	.000	.21 (.15, .27)	.03	.21	.029	.000	.02 (-.04, .08)	.03	.02	.000	.514	.01 (-.06, .08)	.04	.01	.000	.871
Marriage hopes	.00 (-.06, .05)	.03	.00	.000	.929	.02 (-.02, .05)	.02	.02	.000	.388	.02 (-.04, .07)	.03	.02	.000	.504	.03 (-.02, .09)	.03	.04	.001	.262	.02 (-.04, .09)	.03	.03	.001	.471
Marriage worries	-.04 (-.10, .01)	.03	-.04	.001	.125	-.02 (-.06, .01)	.02	-.03	.000	.218	.02 (-.04, .08)	.03	.02	.000	.532	.01 (-.05, .07)	.03	.01	.000	.729	.01 (-.06, .07)	.04	.01	.000	.861
Quality of relationship with twin	.00 (-.05, .05)	.03	.00	.000	.996	.02 (-.02, .06)	.02	.02	.000	.277	-.01 (-.07, .04)	.03	-.01	.000	.665	-.01 (-.07, .05)	.03	-.01	.000	.718	.00 (-.07, .06)	.03	.00	.000	.953
Quality of relationship with mother	.04 (-.02, .09)	.03	.04	.001	.164	.01 (-.03, .05)	.02	.01	.000	.692	.05 (-.01, .10)	.03	.05	.002	.123	-.03 (-.09, .03)	.03	-.04	.001	.302	.00 (-.07, .07)	.03	.00	.000	.944
Quality of relationship with father	.06 (.00, .12)	.03	.06	.002	.049	-.05 (-.10, -.01)	.02	-.06	.002	.013	-.03 (-.09, .04)	.03	-.03	.000	.409	-.05 (-.11, .02)	.03	-.05	.001	.172	-.01 (-.09, .06)	.04	-.02	.000	.720
Number of relationships	.04 (-.03, .10)	.03	.03	.001	.240	.04 (.00, .09)	.02	.04	.001	.061	.03 (-.04, .10)	.04	.02	.000	.436	.02 (-.05, .09)	.04	.02	.000	.543	.00 (-.08, .09)	.04	.00	.000	.933
Longest relationship	.05 (.00, .11)	.03	.05	.002	.060	.04 (.00, .07)	.02	.04	.001	.071	.07 (.02, .13)	.03	.07	.004	.014	.02 (-.04, .08)	.03	.03	.000	.422	.00 (-.07, .07)	.04	.01	.000	.906
Partner violence	-.08 (-.14, -.03)	.03	-.09	.005	.002	.08 (.04, .12)	.02	.09	.005	.000	.06 (.01, .12)	.03	.07	.003	.028	-.05 (-.11, .00)	.03	-.06	.003	.056	-.02 (-.09, .04)	.03	-.03	.001	.502

Contact with mother	.08 (.00, .15)	.04	.07	.002	.052	-.03 (-.08, .03)	.03	-.03	.000	.338	-.05 (-.13, .04)	.04	-.04	.001	.278	-.14 (-.23, -.06)	.04	-.15	.009	.001	-.19 (-.29, -.10)	.05	-.22	.017	.000
Communication with mother	-.03 (-.10, .05)	.04	-.02	.000	.488	.00 (-.05, .05)	.03	.00	.000	.894	.01 (-.06, .09)	.04	.01	.000	.740	.03 (-.05, .10)	.04	.03	.000	.489	.06 (-.03, .15)	.04	.06	.002	.169
Contact with father	-.01 (-.08, .07)	.04	-.01	.000	.865	.02 (-.04, .07)	.03	.02	.000	.541	.04 (-.04, .13)	.04	.05	.001	.284	-.01 (-.09, .07)	.04	-.01	.000	.778	.00 (-.10, .10)	.05	.01	.000	.932
Communication with father	-.05 (-.13, .04)	.04	-.04	.001	.280	.02 (-.04, .08)	.03	.01	.000	.589	.00 (-.09, .09)	.05	.00	.000	.971	.08 (-.01, .18)	.05	.07	.003	.072	.01 (-.09, .12)	.05	.01	.000	.817
Peer pressure	-.02 (-.08, .04)	.03	-.02	.000	.488	.03 (-.01, .06)	.02	.03	.000	.195	.03 (-.03, .09)	.03	.03	.001	.321	.03 (-.03, .09)	.03	.03	.001	.311	-.02 (-.09, .04)	.03	-.03	.001	.488
Physical peer victimisation	-.02 (-.08, .05)	.03	-.02	.000	.637	-.04 (-.08, .00)	.02	-.04	.001	.077	-.02 (-.09, .05)	.04	-.02	.000	.609	.00 (-.07, .07)	.04	.00	.000	.959	.01 (-.07, .09)	.04	.01	.000	.794
Social peer victimisation	-.04 (-.11, .03)	.04	-.04	.001	.276	.09 (.04, .14)	.02	.10	.004	.000	-.07 (-.14, .01)	.04	-.07	.002	.072	.04 (-.03, .12)	.04	.05	.001	.278	-.01 (-.10, .07)	.04	-.02	.000	.793
Verbal peer victimisation	-.04 (-.11, .04)	.04	-.04	.001	.314	.07 (.02, .12)	.03	.08	.002	.004	.08 (.00, .15)	.04	.08	.003	.052	-.01 (-.09, .07)	.04	-.01	.000	.791	.00 (-.08, .09)	.04	.01	.000	.930
Cyber peer victimisation	.02 (-.06, .09)	.04	.02	.000	.682	.01 (-.04, .07)	.03	.01	.000	.603	.00 (-.08, .08)	.04	.00	.000	.998	-.04 (-.12, .04)	.04	-.04	.001	.323	.03 (-.07, .13)	.05	.03	.000	.545
Physical peer Perpetration	.05 (-.02, .12)	.04	.05	.001	.158	.06 (.01, .10)	.03	.05	.001	.029	.04 (-.03, .12)	.04	.04	.001	.267	.00 (-.08, .07)	.04	.00	.000	.960	-.07 (-.16, .02)	.05	-.07	.003	.142
Social peer Perpetration	.04 (-.02, .10)	.03	.04	.001	.218	.03 (-.01, .07)	.02	.03	.000	.194	.01 (-.05, .08)	.03	.01	.000	.681	-.06 (-.13, .00)	.03	-.07	.003	.066	-.07 (-.15, .00)	.04	-.09	.004	.061
Verbal peer Perpetration	.00 (-.06, .07)	.03	.00	.000	.901	-.05 (-.10, -.01)	.02	-.06	.002	.015	-.02 (-.08, .05)	.03	-.02	.000	.663	.03 (-.04, .10)	.03	.04	.001	.371	.03 (-.05, .10)	.04	.03	.000	.517
Cyber peer Perpetration	-.06 (-.13, .01)	.03	-.06	.002	.070	.03 (-.02, .07)	.02	.03	.000	.205	.02 (-.05, .09)	.04	.02	.000	.542	-.01 (-.08, .06)	.04	-.01	.000	.775	.02 (-.07, .10)	.04	.02	.000	.692
Parental role aspirations	-.03 (-.08, .03)	.03	-.03	.000	.350	-.02 (-.06, .01)	.02	-.03	.000	.216	-.02 (-.08, .04)	.03	-.02	.000	.559	-.04 (-.10, .02)	.03	-.04	.001	.196	.02 (-.05, .09)	.04	.02	.000	.548
Occupational role aspirations	-.05 (-.11, .01)	.03	-.05	.001	.121	.03 (-.01, .07)	.02	.03	.001	.181	.04 (-.03, .10)	.03	.04	.001	.252	.09 (.03, .15)	.03	.10	.006	.007	-.02 (-.10, .06)	.04	-.02	.000	.623
Homecare role aspirations	.05 (.00, .10)	.03	.05	.002	.071	.01 (-.03, .04)	.02	.01	.000	.653	-.01 (-.07, .04)	.03	-.01	.000	.623	-.01 (-.07, .05)	.03	-.01	.000	.724	-.06 (-.12, .01)	.03	-.07	.003	.084
Importance of Relationships	.04 (-.03, .11)	.03	.04	.001	.252	.00 (-.04, .05)	.02	.00	.000	.881	-.02 (-.09, .06)	.04	-.01	.000	.680	.05 (-.02, .12)	.04	.05	.002	.147	.01 (-.07, .10)	.04	.01	.000	.786
Achievement Motivation	-.04 (-.10, .02)	.03	-.04	.001	.239	.03 (-.01, .07)	.02	.03	.001	.120	.03 (-.04, .09)	.03	.03	.000	.414	-.01 (-.08, .05)	.03	-.02	.000	.659	.01 (-.06, .09)	.04	.01	.000	.753
Purpose in Life	.20 (.14, .27)	.03	.19	.020	.000	-.19 (-.23, -.14)	.02	-.18	.019	.000	-.03 (-.10, .04)	.04	-.03	.000	.454	.00 (-.07, .07)	.04	.00	.000	.932	-.01 (-.09, .08)	.04	-.01	.000	.865

Importance of democracy and equality	-.04 (-.10, .02)	.03	-.04	.001	.166	.01 (-.03, .05)	.02	.01	.000	.634	.00 (-.05, .06)	.03	.00	.000	.882	.08 (.02, .14)	.03	.08	.005	.008	.04 (-.03, .11)	.04	.04	.001	.264
Environmental concerns	.05 (.00, .11)	.03	.05	.002	.069	-.03 (-.07, .00)	.02	-.04	.001	.078	-.02 (-.08, .04)	.03	-.02	.000	.535	.07 (.01, .13)	.03	.08	.004	.017	.06 (-.02, .13)	.04	.06	.003	.133
Religiosity	.00 (-.06, .05)	.03	.00	.000	.950	.05 (.01, .08)	.02	.05	.002	.010	.01 (-.05, .07)	.03	.01	.000	.739	.06 (.00, .12)	.03	.06	.003	.038	.00 (-.06, .07)	.03	.00	.000	.914
Importance of leisure	.11 (.05, .17)	.03	.11	.007	.000	-.07 (-.11, -.03)	.02	-.07	.003	.001	-.07 (-.13, .00)	.03	-.07	.003	.044	.04 (-.03, .10)	.03	.04	.001	.242	.09 (.01, .16)	.04	.10	.006	.024
Alcohol use	-.02 (-.08, .04)	.03	-.02	.000	.521	-.01 (-.05, .04)	.02	-.01	.000	.741	-.06 (-.13, .01)	.03	-.06	.002	.070	.00 (-.07, .06)	.03	.00	.000	.900	.03 (-.05, .11)	.04	.03	.001	.445
Ever smoked	-.01 (-.07, .05)	.03	-.01	.000	.703	.00 (-.04, .04)	.02	.00	.000	.855	-.04 (-.10, .02)	.03	-.04	.001	.215	-.05 (-.11, .01)	.03	-.06	.002	.088	-.05 (-.12, .02)	.04	-.06	.003	.144
Ever vaped	-.01 (-.06, .04)	.03	-.01	.000	.656	.01 (-.03, .04)	.02	.01	.000	.769	.09 (.03, .14)	.03	.09	.006	.002	-.09 (-.14, -.03)	.03	-.10	.008	.002	-.03 (-.10, .03)	.03	-.04	.001	.313
Cognitive enhancers	-.05 (-.10, .00)	.03	-.06	.003	.036	.02 (-.02, .05)	.02	.02	.000	.334	-.02 (-.08, .03)	.03	-.03	.001	.380	.07 (.02, .12)	.03	.08	.005	.011	.03 (-.03, .09)	.03	.04	.001	.387
Big 5 Agreeableness	.00 (-.05, .05)	.03	.00	.000	.992	.04 (.00, .07)	.02	.04	.001	.055	-.02 (-.08, .03)	.03	-.02	.000	.426	-.05 (-.10, .01)	.03	-.05	.002	.117	-.01 (-.08, .05)	.03	-.02	.000	.682
Big 5 Conscientiousness	-.04 (-.10, .03)	.03	-.03	.001	.311	.06 (.01, .10)	.02	.06	.002	.018	-.04 (-.11, .04)	.04	-.04	.001	.335	.07 (.00, .14)	.04	.08	.003	.052	.06 (-.02, .15)	.04	.08	.003	.126
Big 5 Extraversion	.06 (-.01, .13)	.04	.06	.002	.089	-.12 (-.17, -.07)	.02	-.12	.007	.000	.03 (-.04, .11)	.04	.03	.000	.413	-.02 (-.10, .05)	.04	-.02	.000	.570	-.11 (-.20, -.03)	.05	-.13	.008	.011
Big 5 Neuroticism	-.02 (-.09, .04)	.03	-.02	.000	.468	.26 (.22, .31)	.02	.27	.038	.000	.13 (.06, .19)	.04	.13	.008	.000	-.03 (-.10, .04)	.04	-.04	.001	.362	-.04 (-.12, .04)	.04	-.05	.001	.324
Big 5 Openness	-.04 (-.10, .01)	.03	-.04	.001	.112	.05 (.01, .09)	.02	.05	.002	.006	.01 (-.05, .06)	.03	.01	.000	.799	.01 (-.04, .07)	.03	.02	.000	.636	.00 (-.06, .07)	.03	.00	.000	.953
Self-control	-.02 (-.09, .05)	.04	-.02	.000	.542	-.11 (-.16, -.07)	.02	-.12	.007	.000	.02 (-.05, .10)	.04	.03	.000	.510	-.01 (-.08, .07)	.04	-.01	.000	.885	-.08 (-.16, .01)	.04	-.09	.003	.085
Not planning for the future	.06 (.01, .12)	.03	.07	.003	.024	.07 (.03, .11)	.02	.07	.003	.001	-.01 (-.07, .05)	.03	-.01	.000	.790	-.01 (-.07, .05)	.03	-.01	.000	.674	-.06 (-.12, .01)	.03	-.07	.003	.112
Ambition	.02 (-.04, .09)	.03	.02	.000	.505	.00 (-.05, .04)	.02	.00	.000	.862	-.04 (-.11, .03)	.04	-.04	.001	.223	-.05 (-.12, .03)	.04	-.05	.001	.214	.05 (-.04, .13)	.04	.06	.002	.250
Physical activity	.01 (-.05, .06)	.03	.01	.000	.839	.05 (.01, .09)	.02	.05	.002	.016	-.14 (-.20, -.08)	.03	-.14	.014	.000	.01 (-.05, .07)	.03	.01	.000	.667	.02 (-.05, .09)	.04	.02	.000	.581
Health behaviours	-.07 (-.13, -.02)	.03	-.07	.003	.012	.00 (-.04, .04)	.02	.00	.000	.990	-.01 (-.07, .05)	.03	-.01	.000	.776	.08 (.02, .13)	.03	.08	.005	.010	.10 (.03, .16)	.04	.11	.009	.006

Risk taking behaviour	-.01 (-.08, .05)	.03	-.01	.000	.719	-.02 (-.06, .02)	.02	-.02	.000	.370	.04 (-.03, .10)	.04	.04	.001	.318	.00 (-.07, .07)	.04	.00	.000	.974	-.03 (-.11, .05)	.04	-.04	.001	.444
Risky sexual behaviour	.04 (.02, .07)	.01	.10	.007	.000	.00 (-.02, .02)	.01	.00	.000	.937	.01 (-.01, .04)	.01	.03	.001	.363	.00 (-.02, .03)	.01	.00	.000	.948	-.02 (-.05, .01)	.02	-.06	.002	.155
Media use	.01 (-.05, .07)	.03	.01	.000	.710	.03 (-.01, .07)	.02	.03	.001	.131	.05 (-.02, .11)	.03	.04	.001	.142	-.02 (-.08, .04)	.03	-.02	.000	.468	.00 (-.07, .07)	.04	.00	.000	.981
Volunteering	.08 (.02, .13)	.03	.08	.004	.004	.00 (-.04, .04)	.02	.00	.000	.970	.11 (.05, .16)	.03	.11	.009	.000	.02 (-.03, .08)	.03	.02	.000	.459	.02 (-.05, .08)	.03	.02	.000	.614
Internet dating (scale)	-.11 (-.16, -.05)	.03	-.11	.008	.000	-.05 (-.09, -.02)	.02	-.06	.002	.005	.03 (-.02, .09)	.03	.03	.001	.262	.12 (.07, .18)	.03	.14	.014	.000	.04 (-.02, .11)	.03	.05	.002	.194
Financial literacy: Knowledge of Products	.09 (.04, .15)	.03	.09	.007	.000	.00 (-.03, .04)	.02	.00	.000	.883	.04 (-.02, .09)	.03	.04	.001	.203	-.04 (-.10, .01)	.03	-.05	.002	.114	-.03 (-.10, .04)	.03	-.04	.001	.366
Financial attitudes and behaviour	.20 (.14, .26)	.03	.20	.024	.000	.05 (.01, .09)	.02	.05	.002	.018	-.02 (-.08, .04)	.03	-.02	.000	.487	.00 (-.06, .06)	.03	.00	.000	.969	-.06 (-.14, .01)	.04	-.08	.003	.093
Overall model fit	*F*(56,1061) = 13.06, *p* <.001, R^2 (adjusted) = .41 (.38)					*F*(56,1050) = 43.65, *p* <.001, R^2 (adjusted) = .70 (.68)					*F*(56,1052) = 7.80, *p* <.001, R^2 (adjusted) = .29 (.26)					*F*(56,1058) = 3.87, *p* <.001, R^2 (adjusted) = .17 (.13)					*F*(56,727) = 2.15, *p* <.001, R^2 (adjusted) = .14 (.08)				
(b)	Males																								
	Wellbeing					Adverse mental health					Adverse physical health					Achieved educational level					Planned educational level				
Trait	*B*	*SE B*	*β*	sr^2	*p*	*B*	*SE B*	*β*	sr^2	*p*	*B*	*SE B*	*β*	sr^2	*p*	*B*	*SE B*	*β*	sr^2	*p*	*B*	*SE B*	*β*	sr^2	*p*
Daily hassles	-.02 (-.13, .10)	.06	-.01	.000	.790	.14 (.08, .21)	.03	.17	.017	.000	.07 (-.04, .18)	.05	.07	.003	.201	-.06 (-.18, .06)	.06	-.06	.002	.316	-.08 (-.21, .06)	.07	-.08	.004	.259
CHAOS	-.11 (-.22, .00)	.05	-.10	.007	.043	.09 (.03, .15)	.03	.10	.007	.005	.06 (-.04, .15)	.05	.06	.003	.240	.02 (-.09, .13)	.06	.02	.000	.714	.11 (-.02, .23)	.06	.11	.009	.094
Childhood experiences	-.10 (-.21, .01)	.06	-.09	.005	.077	.00 (-.06, .07)	.03	.00	.000	.918	-.10 (-.20, .00)	.05	-.10	.008	.053	-.05 (-.16, .07)	.06	-.05	.002	.404	.00 (-.13, .13)	.07	.00	.000	.984
Poor sleep quality	-.05 (-.17, .06)	.06	-.05	.001	.357	.14 (.08, .21)	.03	.16	.018	.000	.12 (.02, .23)	.05	.13	.011	.019	.04 (-.08, .15)	.06	.03	.001	.546	.01 (-.11, .14)	.06	.02	.000	.818
Marriage hopes	-.03 (-.13, .07)	.05	-.03	.000	.588	-.01 (-.07, .05)	.03	-.01	.000	.775	.08 (-.01, .17)	.05	.09	.006	.095	-.08 (-.19, .02)	.05	-.08	.005	.127	-.11 (-.23, .00)	.06	-.13	.011	.056
Marriage worries	.02 (-.08, .11)	.05	.02	.000	.731	-.04 (-.10, .02)	.03	-.05	.002	.177	-.04 (-.12, .05)	.05	-.04	.001	.434	.06 (-.04, .16)	.05	.06	.003	.256	.09 (-.03, .20)	.06	.11	.007	.127
Quality of relationship with twin	-.04 (-.14, .06)	.05	-.04	.001	.462	.02 (-.04, .07)	.03	.02	.000	.556	-.01 (-.10, .08)	.05	-.01	.000	.790	.04 (-.07, .14)	.05	.04	.001	.510	.05 (-.06, .17)	.06	.06	.003	.359

Quality of relationship with mother	-.03 (-.14, .08)	.06	-.03	.000	.625	.01 (-.05, .08)	.03	.02	.000	.661	.03 (-.07, .13)	.05	.03	.001	.583	-.01 (-.12, .11)	.06	-.01	.000	.909	.00 (-.13, .13)	.07	.00	.000	.966
Quality of relationship with father	.07 (-.05, .20)	.06	.07	.003	.229	-.04 (-.11, .03)	.04	-.05	.001	.229	-.08 (-.19, .03)	.06	-.08	.004	.178	-.11 (-.24, .02)	.06	-.11	.006	.090	-.09 (-.22, .05)	.07	-.10	.005	.200
Number of relationships	-.03 (-.14, .08)	.06	-.02	.000	.607	.02 (-.05, .09)	.03	.02	.000	.580	.01 (-.09, .12)	.05	.01	.000	.825	.00 (-.12, .12)	.06	.00	.000	.958	-.01 (-.14, .12)	.07	-.01	.000	.831
Longest relationship	.07 (-.04, .17)	.05	.06	.003	.204	-.01 (-.07, .05)	.03	-.01	.000	.765	.02 (-.08, .11)	.05	.02	.000	.755	.03 (-.08, .13)	.05	.03	.001	.620	.02 (-.10, .13)	.06	.02	.000	.806
Partner violence	-.12 (-.24, .00)	.06	-.10	.007	.046	.05 (-.02, .12)	.04	.05	.002	.164	.07 (-.04, .18)	.06	.07	.003	.205	-.11 (-.24, .01)	.06	-.10	.007	.076	-.04 (-.18, .10)	.07	-.04	.001	.572
Contact with mother	.14 (-.02, .29)	.08	.12	.005	.082	-.04 (-.13, .05)	.05	-.05	.001	.350	.00 (-.14, .14)	.07	.00	.000	.966	-.22 (-.38, -.06)	.08	-.21	.016	.007	-.27 (-.45, -.10)	.09	-.30	.029	.003
Communication with mother	-.03 (-.16, .10)	.07	-.03	.000	.672	.01 (-.06, .09)	.04	.02	.000	.701	-.10 (-.22, .02)	.06	-.11	.006	.096	.13 (.00, .27)	.07	.13	.008	.056	.14 (-.01, .29)	.08	.15	.010	.074
Contact with father	-.03 (-.18, .12)	.08	-.02	.000	.736	.04 (-.04, .13)	.04	.05	.001	.311	.09 (-.04, .23)	.07	.10	.004	.171	-.06 (-.22, .10)	.08	-.06	.001	.467	.00 (-.16, .17)	.09	.00	.000	.969
Communication with father	.03 (-.13, .19)	.08	.02	.000	.726	-.06 (-.15, .03)	.05	-.06	.002	.193	.03 (-.12, .17)	.07	.03	.000	.709	.10 (-.07, .27)	.09	.08	.003	.248	.02 (-.17, .21)	.10	.01	.000	.873
Peer pressure	-.02 (-.12, .09)	.05	-.02	.000	.739	.02 (-.04, .08)	.03	.02	.000	.595	.06 (-.04, .15)	.05	.06	.003	.262	-.02 (-.13, .09)	.06	-.02	.000	.776	-.01 (-.12, .11)	.06	-.01	.000	.881
Physical peer victimisation	-.08 (-.19, .03)	.06	-.10	.003	.156	-.02 (-.08, .04)	.03	-.03	.000	.538	.04 (-.06, .14)	.05	.06	.001	.428	-.03 (-.14, .09)	.06	-.04	.000	.643	-.01 (-.13, .12)	.06	-.01	.000	.936
Social peer victimisation	-.02 (-.16, .11)	.07	-.02	.000	.742	.07 (-.01, .14)	.04	.08	.003	.100	.04 (-.09, .16)	.06	.04	.001	.548	.01 (-.13, .16)	.07	.01	.000	.842	.09 (-.05, .24)	.07	.12	.005	.200
Verbal peer victimisation	-.06 (-.18, .07)	.06	-.06	.001	.380	.04 (-.03, .11)	.04	.05	.001	.293	-.02 (-.13, .09)	.06	-.03	.000	.695	.05 (-.07, .18)	.07	.06	.001	.406	-.04 (-.18, .09)	.07	-.06	.001	.517
Cyber peer victimisation	-.02 (-.16, .11)	.07	-.02	.000	.740	.14 (.06, .22)	.04	.17	.011	.001	-.05 (-.18, .07)	.06	-.06	.001	.405	-.02 (-.17, .12)	.07	-.02	.000	.757	-.02 (-.18, .14)	.08	-.02	.000	.797
Physical peer Perpetration	.10 (.00, .20)	.05	.14	.007	.046	.06 (.00, .12)	.03	.11	.004	.038	.01 (-.08, .10)	.05	.02	.000	.789	.03 (-.08, .13)	.05	.04	.000	.637	-.08 (-.19, .04)	.06	-.13	.005	.194
Social peer Perpetration	-.04 (-.16, .08)	.06	-.04	.001	.498	.05 (-.02, .12)	.04	.06	.002	.127	-.02 (-.13, .09)	.06	-.03	.000	.675	-.03 (-.16, .09)	.06	-.03	.001	.613	-.02 (-.15, .11)	.06	-.02	.000	.771
Verbal peer Perpetration	.07 (-.04, .17)	.05	.09	.003	.201	-.08 (-.14, -.02)	.03	-.13	.006	.011	-.01 (-.11, .08)	.05	-.02	.000	.803	.05 (-.06, .16)	.06	.07	.002	.373	.10 (-.02, .21)	.06	.15	.009	.092
Cyber peer Perpetration	-.06 (-.17, .06)	.06	-.06	.002	.331	-.05 (-.11, .02)	.03	-.06	.002	.146	.10 (-.01, .20)	.05	.12	.007	.068	-.07 (-.18, .05)	.06	-.07	.003	.265	.03 (-.10, .16)	.07	.03	.001	.662

Parental role aspirations	.01 (-.10, .12)	.06	.01	.000	.907	.01 (-.06, .07)	.03	.01	.000	.864	-.02 (-.12, .08)	.05	-.02	.000	.724	.09 (-.02, .21)	.06	.09	.005	.112	.08 (-.05, .20)	.06	.08	.004	.241
Occupational role aspirations	-.11 (-.23, .01)	.06	-.10	.006	.071	.08 (.01, .15)	.04	.10	.005	.019	.06 (-.05, .17)	.06	.06	.002	.278	.09 (-.03, .22)	.07	.09	.004	.151	.13 (-.02, .27)	.07	.13	.009	.085
Homecare role aspirations	.08 (-.01, .18)	.05	.09	.005	.083	.00 (-.05, .06)	.03	.00	.000	.944	-.05 (-.13, .04)	.04	-.06	.002	.282	-.06 (-.16, .04)	.05	-.07	.003	.228	-.01 (-.12, .10)	.06	-.01	.000	.839
Importance of Relationships	-.06 (-.18, .05)	.06	-.06	.002	.267	.06 (.00, .13)	.03	.08	.003	.056	.08 (-.03, .18)	.05	.09	.004	.140	.11 (-.01, .23)	.06	.12	.008	.060	.09 (-.05, .22)	.07	.10	.005	.202
Achievement Motivation	.04 (-.07, .15)	.06	.04	.001	.454	.04 (-.02, .10)	.03	.05	.002	.207	-.02 (-.12, .08)	.05	-.02	.000	.716	.03 (-.09, .14)	.06	.03	.000	.627	-.08 (-.20, .05)	.07	-.09	.004	.251
Purpose in Life	.24 (.12, .36)	.06	.22	.026	.000	-.12 (-.19, -.05)	.04	-.14	.010	.001	-.03 (-.13, .08)	.06	-.03	.000	.656	.03 (-.09, .16)	.06	.03	.001	.624	.02 (-.12, .16)	.07	.03	.000	.759
Importance of democracy and equality	.06 (-.03, .16)	.05	.06	.003	.212	-.06 (-.12, -.01)	.03	-.08	.005	.021	-.01 (-.09, .08)	.04	-.01	.000	.914	.05 (-.05, .14)	.05	.05	.002	.365	.05 (-.07, .16)	.06	.06	.002	.405
Environmental concerns	.01 (-.10, .11)	.05	.01	.000	.915	-.02 (-.08, .04)	.03	-.02	.000	.607	-.04 (-.13, .06)	.05	-.04	.001	.469	.13 (.02, .24)	.06	.14	.012	.018	.12 (-.01, .24)	.06	.14	.011	.066
Religiosity	.06 (-.04, .16)	.05	.06	.002	.265	.00 (-.06, .06)	.03	.00	.000	.976	-.01 (-.10, .08)	.05	-.01	.000	.821	.05 (-.06, .16)	.05	.05	.002	.355	.05 (-.06, .17)	.06	.06	.002	.382
Importance of leisure	.12 (.00, .23)	.06	.10	.007	.045	-.06 (-.12, .01)	.03	-.06	.002	.108	-.06 (-.16, .05)	.05	-.06	.002	.273	.06 (-.06, .18)	.06	.06	.002	.328	.11 (-.02, .25)	.07	.12	.008	.104
Alcohol use	-.12 (-.24, .00)	.06	-.11	.007	.048	.02 (-.05, .09)	.04	.02	.000	.630	-.05 (-.16, .06)	.06	-.05	.001	.411	-.04 (-.17, .08)	.06	-.04	.001	.506	.02 (-.12, .16)	.07	.02	.000	.784
Ever smoked	.03 (-.08, .13)	.05	.03	.000	.622	.01 (-.05, .07)	.03	.01	.000	.712	.00 (-.10, .09)	.05	.00	.000	.970	-.10 (-.21, .01)	.06	-.11	.007	.065	-.10 (-.22, .02)	.06	-.13	.009	.085
Ever vaped	.00 (-.09, .09)	.05	.00	.000	.983	.00 (-.05, .06)	.03	.00	.000	.929	.13 (.04, .21)	.04	.16	.017	.004	-.10 (-.19, .00)	.05	-.11	.008	.054	-.11 (-.22, -.01)	.05	-.15	.013	.039
Cognitive enhancers	-.04 (-.12, .05)	.04	-.04	.001	.398	-.01 (-.05, .04)	.02	-.01	.000	.815	.00 (-.08, .07)	.04	-.01	.000	.928	.10 (.02, .19)	.04	.12	.012	.019	.07 (-.02, .16)	.05	.10	.007	.133
Big 5 Agreeableness	.05 (-.04, .15)	.05	.06	.002	.270	.03 (-.02, .09)	.03	.04	.001	.246	-.08 (-.17, .01)	.04	-.10	.007	.073	-.06 (-.16, .04)	.05	-.07	.003	.220	.01 (-.10, .12)	.06	.02	.000	.815
Big 5 Conscientiousness	-.03 (-.15, .09)	.06	-.03	.000	.591	.03 (-.04, .10)	.04	.04	.001	.368	.09 (-.02, .20)	.06	.11	.005	.103	-.01 (-.13, .12)	.06	-.01	.000	.922	.01 (-.12, .14)	.07	.01	.000	.915
Big 5 Extraversion	.10 (-.03, .23)	.07	.10	.004	.116	-.13 (-.20, -.05)	.04	-.16	.011	.001	-.03 (-.14, .09)	.06	-.03	.000	.668	-.02 (-.15, .12)	.07	-.02	.000	.790	-.12 (-.27, .03)	.08	-.14	.007	.125

Big 5 Neuroticism	-.05 (-.17, .07)	.06	-.04	.001	.431	.24 (.17, .31)	.04	.28	.041	.000	.26 (.15, .37)	.06	.28	.041	.000	-.03 (-.16, .10)	.07	-.03	.001	.621	-.17 (-.32, -.02)	.08	-.18	.015	.031
Big 5 Openness	-.03 (-.12, .07)	.05	-.03	.001	.569	.03 (-.02, .09)	.03	.04	.001	.270	.02 (-.07, .10)	.04	.02	.000	.726	-.04 (-.14, .06)	.05	-.04	.001	.445	.04 (-.07, .15)	.06	.05	.002	.461
Self-control	-.05 (-.18, .07)	.06	-.05	.001	.411	-.09 (-.16, -.02)	.04	-.11	.006	.014	.10 (-.02, .21)	.06	.11	.006	.093	.03 (-.10, .16)	.07	.03	.000	.678	-.05 (-.19, .10)	.07	-.05	.001	.529
Not planning for the future	-.01 (-.11, .10)	.05	-.01	.000	.920	.05 (-.01, .10)	.03	.06	.002	.131	.02 (-.07, .12)	.05	.03	.000	.627	.01 (-.09, .12)	.05	.01	.000	.814	-.03 (-.14, .08)	.06	-.04	.001	.616
Ambition	-.08 (-.20, .05)	.06	-.08	.003	.216	-.06 (-.13, .01)	.04	-.08	.003	.094	.01 (-.11, .12)	.06	.01	.000	.929	-.04 (-.17, .09)	.07	-.04	.001	.573	.01 (-.14, .16)	.08	.01	.000	.883
Physical activity	-.03 (-.13, .08)	.05	-.02	.000	.648	.05 (-.01, .11)	.03	.07	.003	.090	-.04 (-.13, .06)	.05	-.04	.001	.454	-.10 (-.21, .01)	.06	-.10	.006	.083	-.05 (-.18, .07)	.06	-.06	.002	.402
Health behaviours	-.14 (-.24, -.05)	.05	-.14	.014	.004	-.07 (-.13, -.01)	.03	-.09	.006	.015	-.10 (-.19, -.01)	.05	-.12	.009	.033	.12 (.02, .22)	.05	.13	.011	.022	.15 (.04, .26)	.06	.18	.023	.007
Risk taking behaviour	.00 (-.11, .12)	.06	.00	.000	.944	.01 (-.05, .08)	.03	.02	.000	.704	.09 (-.01, .20)	.05	.12	.006	.074	-.04 (-.16, .07)	.06	-.05	.001	.468	-.03 (-.16, .10)	.07	-.04	.001	.625
Risky sexual behaviour	.07 (.02, .11)	.02	.16	.016	.002	.01 (-.01, .04)	.01	.04	.001	.294	.02 (-.02, .06)	.02	.06	.002	.322	-.01 (-.05, .04)	.02	-.02	.000	.780	.00 (-.05, .05)	.03	.01	.000	.947
Media use	.02 (-.09, .12)	.05	.02	.000	.731	.05 (-.01, .11)	.03	.06	.002	.110	.02 (-.08, .11)	.05	.02	.000	.719	-.06 (-.17, .04)	.06	-.07	.003	.241	-.09 (-.22, .03)	.06	-.11	.007	.125
Volunteering	.06 (-.04, .16)	.05	.06	.002	.245	.04 (-.02, .10)	.03	.05	.001	.210	.10 (.01, .19)	.05	.11	.009	.033	.01 (-.09, .12)	.05	.01	.000	.815	-.07 (-.19, .04)	.06	-.08	.005	.209
Internet dating (scale)	-.09 (-.19, .02)	.05	-.09	.005	.096	-.04 (-.10, .02)	.03	-.06	.002	.140	-.02 (-.11, .08)	.05	-.02	.000	.723	.22 (.12, .33)	.06	.23	.035	.000	.09 (-.03, .21)	.06	.11	.007	.139
Financial literacy: Knowledge of Products	.11 (.02, .20)	.05	.11	.009	.022	-.01 (-.06, .05)	.03	-.01	.000	.857	.03 (-.06, .12)	.04	.04	.001	.512	-.05 (-.15, .05)	.05	-.06	.002	.286	-.06 (-.18, .05)	.06	-.08	.004	.267
Financial attitudes and behaviour	.19 (.08, .30)	.06	.20	.020	.001	.05 (-.01, .12)	.03	.07	.003	.104	-.02 (-.12, .08)	.05	-.02	.000	.724	.01 (-.10, .13)	.06	.01	.000	.836	-.02 (-.16, .11)	.07	-.03	.000	.730
Overall model fit	$F(56,347) = 4.25$, $p <.001$, R^2 (adjusted) = .41 (.31)					$F(56,341) = 12.94$, $p <.001$, R^2 (adjusted) = .68 (.63)					$F(56,342) = 2.77$, $p <.001$, R^2 (adjusted) = .31 (.20)					$F(56,346) = 2.17$, $p <.001$, R^2 (adjusted) = .26 (.14)					$F(56,223) = 1.79$, $p = .002$, R^2 (adjusted) = .31 (.14)				
(c)	Females																								
	Wellbeing					Adverse mental health					Adverse physical health					Achieved educational level					Planned educational level				
Trait	*B*	*SE B*	*β*	sr^2	*p*	*B*	*SE B*	*β*	sr^2	*p*	*B*	*SE B*	*β*	sr^2	*p*	*B*	*SE B*	*β*	sr^2	*p*	*B*	*SE B*	*β*	sr^2	*p*

Daily hassles	-.08 (-.15, -.01)	.04	-.08	.004	.035	.15 (.10, .21)	.03	.14	.013	.000	.07 (-.01, .16)	.04	.07	.003	.081	.06 (-.02, .14)	.04	.07	.003	.117	.02 (-.08, .12)	.05	.03	.000	.657
CHAOS	-.12 (-.18, -.05)	.03	-.12	.011	.000	.06 (.01, .10)	.02	.06	.003	.013	.11 (.04, .18)	.04	.11	.009	.003	-.03 (-.10, .04)	.03	-.04	.001	.362	-.05 (-.13, .04)	.04	-.06	.002	.279
Childhood experiences	-.04 (-.11, .03)	.03	-.04	.001	.259	.07 (.02, .12)	.03	.07	.003	.004	.08 (.01, .16)	.04	.08	.004	.034	.04 (-.03, .11)	.04	.05	.001	.273	.05 (-.04, .13)	.04	.06	.002	.301
Poor sleep quality	-.03 (-.10, .04)	.04	-.03	.001	.390	.18 (.13, .23)	.03	.18	.020	.000	.25 (.17, .33)	.04	.24	.037	.000	.01 (-.07, .08)	.04	.01	.000	.879	.01 (-.08, .10)	.05	.01	.000	.865
Marriage hopes	.02 (-.05, .08)	.03	.02	.000	.601	.03 (-.02, .07)	.02	.03	.000	.266	.01 (-.06, .08)	.04	.01	.000	.818	.07 (.01, .14)	.03	.09	.006	.031	.06 (-.02, .14)	.04	.07	.004	.154
Marriage worries	-.06 (-.13, .01)	.04	-.06	.003	.068	-.02 (-.07, .03)	.03	-.02	.000	.441	.04 (-.04, .12)	.04	.04	.001	.326	-.02 (-.09, .06)	.04	-.02	.000	.684	-.02 (-.11, .07)	.05	-.03	.000	.615
Quality of relationship with twin	.03 (-.04, .09)	.03	.03	.001	.387	.02 (-.03, .07)	.02	.02	.000	.416	-.01 (-.08, .06)	.04	-.01	.000	.817	-.02 (-.08, .05)	.03	-.02	.000	.628	-.02 (-.11, .06)	.04	-.03	.001	.582
Quality of relationship with mother	.05 (-.01, .12)	.03	.06	.002	.108	.01 (-.04, .05)	.02	.01	.000	.775	.06 (-.01, .14)	.04	.07	.003	.089	-.03 (-.10, .04)	.04	-.04	.001	.421	.01 (-.08, .09)	.04	.01	.000	.883
Quality of relationship with father	.07 (.00, .15)	.04	.08	.003	.050	-.06 (-.11, .00)	.03	-.06	.002	.034	.01 (-.07, .10)	.04	.01	.000	.734	-.05 (-.13, .03)	.04	-.06	.002	.200	.00 (-.10, .11)	.05	.01	.000	.931
Number of relationships	.08 (.00, .17)	.04	.06	.003	.048	.05 (-.01, .11)	.03	.04	.001	.087	.05 (-.04, .14)	.05	.04	.001	.279	.03 (-.07, .12)	.05	.02	.000	.589	.02 (-.09, .13)	.06	.02	.000	.731
Longest relationship	.08 (.01, .15)	.04	.08	.004	.022	.07 (.02, .12)	.03	.06	.003	.010	.14 (.06, .22)	.04	.13	.012	.000	.00 (-.07, .08)	.04	.00	.000	.979	-.03 (-.12, .07)	.05	-.03	.001	.573
Partner violence	-.07 (-.13, -.01)	.03	-.08	.004	.027	.09 (.05, .13)	.02	.10	.007	.000	.06 (-.01, .13)	.03	.06	.003	.089	-.05 (-.12, .01)	.03	-.07	.003	.106	-.02 (-.10, .06)	.04	-.03	.000	.609
Contact with mother	.06 (-.04, .15)	.05	.05	.001	.237	-.01 (-.07, .06)	.03	-.01	.000	.877	-.08 (-.18, .03)	.05	-.07	.002	.153	-.13 (-.22, -.03)	.05	-.15	.008	.011	-.18 (-.31, -.05)	.07	-.21	.014	.007
Communication with mother	-.03 (-.11, .06)	.04	-.02	.000	.580	.00 (-.06, .07)	.03	.00	.000	.943	.06 (-.04, .16)	.05	.05	.001	.235	.00 (-.10, .09)	.05	.00	.000	.946	.04 (-.08, .15)	.06	.04	.001	.515
Contact with father	-.02 (-.11, .07)	.05	-.02	.000	.660	-.02 (-.08, .05)	.03	-.02	.000	.663	.04 (-.07, .14)	.05	.04	.001	.466	.01 (-.08, .11)	.05	.02	.000	.791	.02 (-.11, .15)	.07	.02	.000	.805
Communication with father	-.07 (-.18, .03)	.05	-.06	.002	.163	.04 (-.04, .12)	.04	.03	.000	.317	-.03 (-.15, .09)	.06	-.03	.000	.593	.09 (-.02, .20)	.06	.08	.003	.112	.00 (-.14, .14)	.07	.00	.000	.978
Peer pressure	-.02 (-.09, .05)	.03	-.02	.000	.544	.03 (-.02, .07)	.03	.02	.000	.312	.02 (-.05, .10)	.04	.02	.000	.545	.06 (-.01, .13)	.04	.07	.004	.082	-.03 (-.12, .05)	.05	-.04	.001	.457
Physical peer victimisation	.06 (-.03, .15)	.05	.04	.001	.181	-.02 (-.09, .04)	.03	-.02	.000	.510	-.05 (-.15, .05)	.05	-.04	.001	.337	.02 (-.07, .12)	.05	.02	.000	.648	-.01 (-.12, .11)	.06	-.01	.000	.880

Social peer victimisation	-.05 (-.14, .04)	.04	-.05	.001	.247	.11 (.04, .17)	.03	.11	.004	.001	-.09 (-.19, .00)	.05	-.10	.004	.057	.05 (-.04, .14)	.05	.06	.002	.259	-.07 (-.18, .05)	.06	-.09	.003	.242
Verbal peer victimisation	-.02 (-.11, .07)	.05	-.02	.000	.705	.11 (.05, .18)	.03	.11	.004	.001	.14 (.04, .24)	.05	.13	.007	.009	-.07 (-.16, .03)	.05	-.08	.002	.182	.03 (-.09, .15)	.06	.03	.000	.651
Cyber peer victimisation	.03 (-.07, .12)	.05	.02	.000	.603	-.07 (-.14, .00)	.04	-.06	.002	.053	.01 (-.10, .12)	.06	.01	.000	.865	-.03 (-.13, .07)	.05	-.03	.000	.587	.09 (-.04, .22)	.07	.10	.003	.182
Physical peer Perpetration	.01 (-.16, .18)	.09	.00	.000	.916	.07 (-.06, .19)	.06	.02	.000	.297	-.05 (-.24, .15)	.10	-.02	.000	.651	.13 (-.06, .31)	.09	.05	.002	.180	-.03 (-.26, .21)	.12	-.01	.000	.817
Social peer Perpetration	.07 (-.01, .15)	.04	.07	.003	.066	.02 (-.04, .07)	.03	.02	.000	.564	.00 (-.09, .09)	.05	.00	.000	.946	-.06 (-.14, .03)	.04	-.07	.002	.171	-.08 (-.18, .02)	.05	-.10	.005	.106
Verbal peer Perpetration	-.03 (-.11, .05)	.04	-.03	.000	.491	-.03 (-.09, .03)	.03	-.03	.000	.280	.00 (-.10, .09)	.05	.00	.000	.993	.00 (-.09, .09)	.05	.00	.000	.980	-.04 (-.15, .08)	.06	-.04	.001	.528
Cyber peer Perpetration	-.05 (-.14, .04)	.05	-.04	.001	.244	.08 (.01, .14)	.03	.06	.002	.023	.00 (-.10, .10)	.05	.00	.000	.942	.02 (-.08, .11)	.05	.02	.000	.725	.00 (-.11, .12)	.06	.00	.000	.945
Parental role aspirations	-.04 (-.10, .03)	.03	-.04	.001	.247	-.03 (-.08, .02)	.03	-.03	.001	.210	-.02 (-.10, .05)	.04	-.02	.000	.543	-.10 (-.17, -.03)	.04	-.11	.009	.007	.02 (-.08, .11)	.05	.02	.000	.728
Occupational role aspirations	-.02 (-.09, .05)	.04	-.02	.000	.579	.00 (-.05, .05)	.03	.00	.000	.985	.04 (-.04, .12)	.04	.04	.001	.366	.09 (.01, .16)	.04	.10	.006	.026	-.06 (-.16, .04)	.05	-.07	.003	.217
Homecare role aspirations	.05 (-.02, .12)	.03	.05	.002	.130	.00 (-.05, .05)	.03	.00	.000	.886	-.01 (-.08, .07)	.04	-.01	.000	.854	.01 (-.06, .09)	.04	.02	.000	.707	-.09 (-.18, .00)	.05	-.10	.007	.047
Importance of Relationships	.09 (.01, .18)	.05	.08	.003	.037	-.03 (-.09, .04)	.03	-.02	.000	.410	-.09 (-.18, .01)	.05	-.07	.003	.092	.03 (-.07, .12)	.05	.03	.000	.580	-.03 (-.14, .09)	.06	-.02	.000	.678
Achievement Motivation	-.06 (-.14, .01)	.04	-.06	.002	.102	.03 (-.03, .08)	.03	.03	.000	.301	.07 (-.02, .16)	.04	.06	.003	.106	-.06 (-.14, .02)	.04	-.07	.003	.118	.02 (-.08, .12)	.05	.03	.000	.654
Purpose in Life	.18 (.10, .26)	.04	.17	.015	.000	-.22 (-.28, -.16)	.03	-.20	.021	.000	-.06 (-.16, .03)	.05	-.06	.002	.176	.00 (-.08, .09)	.05	.00	.000	.934	.00 (-.11, .11)	.06	.00	.000	.989
Importance of democracy and equality	-.10 (-.17, -.03)	.04	-.09	.006	.007	.05 (-.01, .10)	.03	.04	.001	.078	.02 (-.06, .10)	.04	.02	.000	.662	.10 (.02, .18)	.04	.10	.007	.014	.02 (-.08, .12)	.05	.02	.000	.687
Environmental concerns	.06 (-.01, .13)	.04	.06	.002	.098	-.04 (-.09, .01)	.03	-.04	.001	.125	-.02 (-.10, .06)	.04	-.02	.000	.618	.04 (-.04, .11)	.04	.04	.001	.322	.03 (-.07, .13)	.05	.03	.001	.577
Religiosity	-.03 (-.09, .03)	.03	-.03	.001	.349	.06 (.01, .11)	.02	.05	.002	.016	.00 (-.08, .07)	.04	.00	.000	.924	.06 (-.01, .13)	.04	.07	.004	.070	.01 (-.08, .09)	.04	.01	.000	.852
Importance of leisure	.11 (.04, .18)	.04	.11	.007	.003	-.06 (-.11, .00)	.03	-.05	.002	.039	-.06 (-.15, .02)	.04	-.06	.002	.125	.02 (-.06, .09)	.04	.02	.000	.705	.06 (-.03, .16)	.05	.07	.003	.211
Alcohol use	.05 (-.03, .12)	.04	.04	.001	.230	-.02 (-.07, .04)	.03	-.01	.000	.601	-.06 (-.14, .03)	.04	-.05	.002	.205	-.02 (-.10, .07)	.04	-.02	.000	.689	.02 (-.08, .11)	.05	.02	.000	.760

Ever smoked	-.04 (-.11, .03)	.04	-.04	.001	.277	.00 (-.05, .05)	.03	.00	.000	.927	-.05 (-.13, .02)	.04	-.05	.002	.169	-.02 (-.10, .05)	.04	-.03	.000	.530	-.03 (-.12, .07)	.05	-.03	.001	.585
Ever vaped	-.02 (-.08, .05)	.03	-.02	.000	.562	.00 (-.05, .04)	.02	.00	.000	.882	.06 (-.02, .13)	.04	.06	.002	.130	-.08 (-.15, -.01)	.04	-.10	.007	.020	-.01 (-.09, .08)	.04	-.01	.000	.860
Cognitive enhancers	-.08 (-.15, -.02)	.03	-.09	.005	.012	.03 (-.02, .07)	.02	.03	.000	.269	-.07 (-.15, .00)	.04	-.07	.004	.054	.04 (-.03, .11)	.04	.05	.001	.277	.02 (-.07, .10)	.04	.02	.000	.712
Big 5 Agreeableness	-.03 (-.10, .04)	.04	-.03	.001	.408	.04 (-.01, .10)	.03	.04	.001	.087	.04 (-.03, .12)	.04	.04	.001	.267	-.06 (-.13, .02)	.04	-.06	.003	.130	-.04 (-.13, .05)	.05	-.04	.001	.417
Big 5 Conscientiousness	-.04 (-.12, .05)	.04	-.04	.001	.370	.04 (-.02, .11)	.03	.04	.001	.161	-.14 (-.23, -.04)	.05	-.13	.008	.005	.10 (.01, .19)	.05	.11	.006	.027	.11 (-.01, .22)	.06	.12	.006	.066
Big 5 Extraversion	-.01 (-.09, .08)	.04	-.01	.000	.856	-.13 (-.19, -.07)	.03	-.12	.006	.000	.08 (-.02, .18)	.05	.07	.003	.105	.02 (-.08, .11)	.05	.02	.000	.743	-.08 (-.20, .03)	.06	-.09	.004	.146
Big 5 Neuroticism	-.05 (-.13, .03)	.04	-.05	.001	.204	.25 (.19, .31)	.03	.24	.027	.000	.07 (-.02, .16)	.05	.07	.002	.129	-.05 (-.14, .04)	.04	-.06	.002	.250	-.01 (-.11, .10)	.05	-.01	.000	.911
Big 5 Openness	-.04 (-.11, .03)	.03	-.04	.001	.234	.05 (.00, .10)	.03	.05	.002	.044	.02 (-.06, .10)	.04	.02	.000	.594	.03 (-.05, .10)	.04	.03	.001	.495	.00 (-.09, .09)	.05	.00	.000	.942
Self-control	-.03 (-.12, .05)	.04	-.03	.000	.457	-.12 (-.19, -.06)	.03	-.12	.006	.000	-.02 (-.11, .08)	.05	-.02	.000	.698	-.03 (-.12, .06)	.05	-.03	.000	.549	-.10 (-.22, .01)	.06	-.12	.006	.079
Not planning for the future	.09 (.02, .15)	.04	.09	.005	.015	.08 (.03, .13)	.03	.07	.004	.003	-.04 (-.11, .04)	.04	-.03	.001	.375	-.03 (-.11, .04)	.04	-.04	.001	.378	-.07 (-.16, .02)	.05	-.09	.005	.113
Ambition	.08 (.00, .16)	.04	.08	.003	.042	.03 (-.03, .08)	.03	.02	.000	.409	-.06 (-.15, .03)	.05	-.06	.002	.212	-.05 (-.13, .04)	.04	-.05	.001	.288	.07 (-.04, .17)	.05	.07	.003	.218
Physical activity	.04 (-.03, .11)	.04	.04	.001	.289	.04 (-.02, .09)	.03	.03	.001	.170	-.20 (-.28, -.12)	.04	-.18	.023	.000	.07 (-.01, .14)	.04	.07	.004	.083	.06 (-.04, .15)	.05	.07	.003	.222
Health behaviours	-.03 (-.09, .04)	.03	-.03	.000	.461	.04 (-.01, .09)	.03	.04	.001	.141	.04 (-.04, .11)	.04	.04	.001	.345	.05 (-.03, .12)	.04	.06	.002	.199	.07 (-.02, .16)	.05	.08	.004	.150
Risk taking behaviour	-.05 (-.13, .03)	.04	-.05	.001	.241	-.05 (-.11, .01)	.03	-.04	.001	.131	.01 (-.08, .10)	.05	.01	.000	.806	.03 (-.06, .12)	.05	.03	.001	.513	-.03 (-.14, .08)	.05	-.03	.001	.589
Risky sexual behaviour	.03 (.00, .06)	.02	.07	.003	.059	-.01 (-.03, .02)	.01	-.01	.000	.597	.01 (-.02, .04)	.02	.03	.000	.490	.01 (-.02, .04)	.02	.04	.001	.421	-.03 (-.06, .02)	.02	-.07	.003	.217
Media use	.00 (-.07, .07)	.04	.00	.000	.950	.01 (-.04, .07)	.03	.01	.000	.653	.06 (-.03, .14)	.04	.05	.002	.188	-.01 (-.09, .07)	.04	-.01	.000	.811	.04 (-.06, .14)	.05	.04	.001	.414
Volunteering	.09 (.03, .15)	.03	.10	.007	.004	-.02 (-.06, .03)	.02	-.02	.000	.526	.10 (.03, .17)	.04	.10	.007	.007	.03 (-.04, .10)	.03	.03	.001	.404	.06 (-.02, .14)	.04	.07	.004	.154
Internet dating (scale)	-.09 (-.15, -.03)	.03	-.10	.006	.005	-.05 (-.09, .00)	.02	-.05	.002	.045	.05 (-.02, .12)	.04	.05	.002	.199	.07 (.01, .14)	.03	.09	.006	.033	.01 (-.08, .09)	.04	.01	.000	.851

Financial literacy: Knowledge of Products	.07 (.01, .14)	.03	.07	.004	.025	-.01 (-.06, .04)	.02	-.01	.000	.607	.01 (-.06, .08)	.04	.01	.000	.768	-.03 (-.10, .04)	.04	-.04	.001	.332	.01 (-.08, .09)	.04	.01	.000	.881
Financial attitudes and behaviour	.20 (.13, .27)	.04	.19	.024	.000	.06 (.01, .11)	.03	.06	.002	.025	-.01 (-.09, .07)	.04	-.01	.000	.829	.00 (-.08, .07)	.04	-.01	.000	.910	-.09 (-.18, .01)	.05	-.10	.005	.089
Overall model fit	$F(56,657) = 1.51$, $p < .001$, R^2 (adjusted) = .47 (.43)					$F(56,652) = 32.82$, $p < .001$, R^2 (adjusted) = .74 (.72)					$F(56,653) = 6.60$, $p < .001$, R^2 (adjusted) = .36 (.31)					$F(56,655) = 2.92$, $p < .001$, R^2 (adjusted) = .20 (.13)					$F(56,447) = 1.49$, $p = .016$, R^2 (adjusted) = .16 (.05)				

Note. Scores were corrected for mean age and sex differences (see Methods). These results are based on one twin randomly selected from each pair so that the data points are independent.

Note. The binary (yes/no) internet dating variable was excluded from these analyses as it formed part of the internet dating scale variable.

Table S8. Twin intraclass correlations and Falconer ACE estimates, and model fitting results for univariate analyses of additive genetic (A), shared environmental (C), and non-shared environmental (E) components of variance for variables for psychological traits and functional outcomes (95% confidence intervals are in parentheses). (a) for the whole sample; (b) males only; (c) females only; (d) MZ and DZ same sex twin pairs (opposite sex DZ twins excluded**).**

(a)

	MZ			DZ[a]			Falconer ACE estimates		
Trait	*r*	*p*	*n*	*r*	*p*	*n*	A	C	E
Daily hassles	.31 (.26,.36)	<.001	1376	.15 (.10, .19)	<.001	2050	.31[b]	.00	.69
CHAOS	.40 (.36,.44)	<.001	1588	.36 (.32, .39)	<.001	2516	.07	.32	.61
Childhood experiences	.66 (.63,.69)	<.001	1367	.40 (.36, .43)	<.001	2039	.52	.14	.34
Poor sleep quality	.31 (.26,.36)	<.001	1334	.15 (.11, .20)	<.001	2001	.31[b]	.00	.69
Marriage hopes	.28 (.23,.33)	<.001	1550	.18 (.14, .22)	<.001	2417	.19	.09	.73
Marriage worries	.38 (.34,.42)	<.001	1549	.19 (.15, .23)	<.001	2418	.37	.01	.62
Quality of relationship with twin	.69 (.66,.72)	<.001	1497	.62 (.59, .64)	<.001	2339	.14	.55	.31
Quality of relationship with mother	.53 (.49,.57)	<.001	1478	.26 (.22, .29)	<.001	2308	.53[b]	.00	.47
Quality of relationship with father	.61 (.58,.64)	<.001	1422	.41 (.38, .44)	<.001	2229	.39	.22	.39
Number of relationships	.46 (.42,.50)	<.001	1548	.19 (.15, .23)	<.001	2416	.46[b]	.00	.54
Longest relationship	.32 (.27,.37)	<.001	1152	.08 (.03, .12)	.002	1736	.32[b]	.00	.68
Partner violence	.25 (.20,.30)	<.001	1457	.12 (.07, .16)	<.001	2264	.25[b]	.00	.75
Contact with mother	.55 (.48,.61)	<.001	426	.30 (.22, .37)	<.001	559	.50	.05	.45

Communication with mother	.64 (.58,.69)	<.001	440	.38 (.30, .45)	<.001	574	.54	.11	.36
Contact with father	.50 (.43,.56)	<.001	511	.40 (.33, .46)	<.001	694	.21	.29	.50
Communication with father	.73 (.69,.77)	<.001	575	.64 (.59, .68)	<.001	764	.18	.54	.27
Peer pressure	.35 (.30,.39)	<.001	1494	.16 (.12, .19)	<.001	2371	.35[b]	.00	.65
Physical peer victimisation	.20 (.15,.25)	<.001	1308	.02 (-.03, .06)	.417	1935	.20[b]	.00	.80
Social peer victimisation	.24 (.19,.29)	<.001	1308	.14 (.10, .18)	<.001	1934	.21	.03	.76
Verbal peer victimisation	.29 (.24,.34)	<.001	1308	.13 (.09, .18)	<.001	1934	.29[b]	.00	.71
Cyber peer victimisation	.33 (.28,.38)	<.001	1307	.12 (.07, .16)	<.001	1934	.33[b]	.00	.67
Physical peer Perpetration	.14 (.09,.19)	<.001	1308	.01 (-.03, .06)	.569	1936	.14[b]	.00	.86
Social peer Perpetration	.14 (.09,.19)	<.001	1307	.05 (.00, .09)	.030	1936	.14[b]	.00	.86
Verbal peer Perpetration	.32 (.27,.37)	<.001	1307	.12 (.07, .16)	<.001	1936	.32[b]	.00	.68
Cyber peer Perpetration	.20 (.15,.25)	<.001	1307	.03 (-.02, .07)	.247	1936	.20[b]	.00	.80
Parental role aspirations	.35 (.30,.40)	<.001	1373	.16 (.11, .20)	<.001	2042	.35[b]	.00	.65
Occupational role aspirations	.27 (.22,.32)	<.001	1374	.11 (.07, .15)	<.001	2045	.27[b]	.00	.73
Homecare role aspirations	.42 (.38,.46)	<.001	1374	.17 (.13, .21)	<.001	2045	.42[b]	.00	.58
Importance of Relationships	.41 (.37,.45)	<.001	1543	.10 (.06, .13)	<.001	2434	.41[b]	.00	.59
Achievement Motivation	.37 (.33,.41)	<.001	1543	.18 (.14, .21)	<.001	2434	.37 [b]	.00	.63

Purpose in Life	.42 (.38,.46)	<.001	1542	.21 (.18, .25)	<.001	2432	.41	.01	.58
Importance of democracy and equality	.43 (.39,.47)	<.001	1541	.18 (.14, .22)	<.001	2424	.43[b]	.00	.57
Environmental concerns	.51 (.47,.55)	<.001	1542	.27 (.23, .30)	<.001	2428	.49	.02	.49
Religiosity	.72 (.69,.74)	<.001	1498	.56 (.54, .59)	<.001	2374	.32	.41	.28
Importance of leisure	.41 (.36,.45)	<.001	1338	.19 (.15, .23)	<.001	1997	.41[b]	.00	.59
Alcohol use	.45 (.40,.50)	<.001	1126	.26 (.22, .31)	<.001	1717	.38	.07	.55
Ever smoked	.49 (.45,.53)	<.001	1342	.23 (.19, .27)	<.001	1995	.49[b]	.00	.51
Ever vaped	.36 (.31,.41)	<.001	1342	.20 (.16, .24)	<.001	1990	.31	.04	.64
Cognitive enhancers	.33 (.28,.38)	<.001	1322	.18 (.14, .23)	<.001	1955	.29	.04	.67
Big 5 Agreeableness	.27 (.22,.32)	<.001	1419	.03 (-.01, .07)	.117	2266	.27[b]	.00	.73
Big 5 Conscientiousness	.34 (.29,.39)	<.001	1418	.07 (.03, .11)	.001	2262	.34[b]	.00	.66
Big 5 Extraversion	.44 (.40,.48)	<.001	1424	.13 (.09, .17)	<.001	2276	.44[b]	.00	.56
Big 5 Neuroticism	.37 (.32,.41)	<.001	1426	.18 (.14, .22)	<.001	2280	.37[b]	.00	.63
Big 5 Openness	.40 (.36,.44)	<.001	1421	.11 (.07, .15)	<.001	2271	.40[b]	.00	.60
Self-control	.40 (.36,.44)	<.001	1417	.14 (.10, .18)	<.001	2259	.40[b]	.00	.60
Not planning for the future	.25 (.20,.30)	<.001	1416	.13 (.09, .17)	<.001	2258	.24	.01	.75
Ambition	.37 (.32,.41)	<.001	1376	.16 (.11, .20)	<.001	2051	.37[b]	.00	.63
Physical activity	.40 (.36,.44)	<.001	1528	.17 (.13, .21)	<.001	2371	.40[b]	.00	.60
Health behaviours	.54 (.50,.57)	<.001	1533	.29 (.25, .33)	<.001	2391	.50	.04	.46

Risk taking behaviour	.48 (.44,.52)	<.001	1413	.23 (.19, .27)	<.001	2258	.48[b]	.00	.52
Risky sexual behaviour	.61 (.58,.64)	<.001	1413	.29 (.25, .33)	<.001	2206	.61[b]	.00	.39
Media use	.53 (.49,.57)	<.001	1520	.19 (.15, .22)	<.001	2350	.53[b]	.00	.47
Volunteering	.45 (.41,.49)	<.001	1500	.20 (.16, .24)	<.001	2378	.45[b]	.00	.55
Internet dating (scale)	.34 (.29,.38)	<.001	1512	.17 (.13, .21)	<.001	2337	.34[b]	.00	.66
Internet dating (y/n)	.32 (.27,.36)	<.001	1519	.15 (.11, .18)	<.001	2351	.32[b]	.00	.68
Financial literacy: Knowledge of Products	.38 (.34,.42)	<.001	1481	.15 (.11, .19)	<.001	2266	.38[b]	.00	.62
Financial attitudes and behaviour	.30 (.25,.35)	<.001	1480	.09 (.05, .13)	<.001	2266	.30[b]	.00	.70
	MZ			DZ[a]			Falconer ACE estimates		
Functional outcome	*r*	*p*	*n*	*r*	*p*	*n*	A	C	E
Wellbeing factor (1st PC)	.39 (.35, .43)	<.001	1477	.23 (.19, .27)	<.001	2249	.32	.07	.61
Adverse mental health factor (1st PC)	.50 (.45, .54)	<.001	1042	.21 (.17, .26)	<.001	1578	.50[b]	.00	.50
Adverse physical health factor (1st PC)	.40 (.35, .45)	<.001	1234	.17 (.13, .22)	<.001	1786	.40[b]	.00	.60
Achieved educational level	.40 (.36, .44)	<.001	1520	.28 (.24, .32)	<.001	2365	.24	.16	.60
Planned educational level	.37 (.31, .43)	<.001	735	.11 (.05, .17)	<.001	1068	.37[b]	.00	.63

Note. MZ=monozygotic; DZ=dizygotic; and genetic (A), shared environmental (C) and non-shared environmental (E) estimates. Scores were corrected for mean age and sex differences (see Methods). Falconer's ACE estimates were derived from the intraclass twin correlations using Falconer's formula (Rijsdijk & Sham, 2002).

[a] Same-sex and opposite-sex DZ twins combined.

[b] rMZ reported as A would otherwise exceed rMZ.

(b) Males only

Trait	MZ			DZ[a]			Falconer ACE estimates		
	r	*p*	*n*	*r*	*p*	*n*	A	C	E
Daily hassles	.31 (.22, .39)	<.001	445	.07 (-.04, .17)	.230	344	.31[b]	.00	.69
CHAOS	.38 (.30, .45)	<.001	523	.24 (.15, .33)	<.001	438	.28	.11	.62
Childhood experiences	.52 (.45, .59)	<.001	439	.35 (.25, .44)	<.001	336	.35	.17	.48
Poor sleep quality	.26 (.17, .35)	<.001	432	.19 (.08, .29)	<.001	334	.13	.12	.74
Marriage hopes	.14 (.05, .22)	.001	512	.14 (.05, .23)	.005	424	.01	.13	.86
Marriage worries	.37 (.29, .44)	<.001	511	.15 (.06, .24)	.002	424	.37[b]	.00	.63
Quality of relationship with twin	.70 (.65, .74)	<.001	488	.62 (.56, .68)	<.001	409	.16	.54	.30
Quality of relationship with mother	.48 (.41, .55)	<.001	479	.15 (.05, .24)	.003	407	.48[b]	.00	.53
Quality of relationship with father	.56 (.49, .62)	<.001	461	.34 (.25, .43)	<.001	386	.44	.12	.44
Number of relationships	.50 (.43, .56)	<.001	512	.21 (.12, .30)	<.001	423	.50[b]	.00	.50
Longest relationship	.26 (.16, .35)	<.001	368	.13 (.02, .24)	.030	298	.26[b]	.00	.74
Partner violence	.24 (.15, .32)	<.001	471	.10 (.00, .20)	.060	389	.24[b]	.00	.76
Contact with mother	.48 (.34, .60)	<.001	132	.40 (.21, .56)	<.001	88	.15	.33	.52
Communication with mother	.58 (.46, .68)	<.001	136	.44 (.26, .59)	<.001	91	.29	.29	.42
Contact with father	.46 (.33, .57)	<.001	159	.43 (.26, .57)	<.001	106	.07	.39	.54
Communication with father	.69 (.60, .76)	<.001	179	.49 (.34, .62)	<.001	116	.41	.28	.31
Peer pressure	.40 (.32, .47)	<.001	495	.18 (.09, .27)	<.001	422	.40[b]	.00	.60

Physical peer victimisation	.25 (.16, .34)	<.001	427	.04 (-.07, .15)	.486	331	.25[b]	.00	.75
Social peer victimisation	.13 (.04, .22)	.005	427	.26 (.16, .36)	<.001	331	.00	.13	.87
Verbal peer victimisation	.24 (.15, .33)	<.001	427	.13 (.02, .23)	.017	329	.22	.02	.76
Cyber peer victimisation	.23 (.14, .32)	<.001	427	.13 (.02, .23)	.017	330	.19	.04	.77
Physical peer Perpetration	.17 (.08, .26)	.001	426	.09 (-.02, .20)	.115	331	.16	.01	.84
Social peer Perpetration	.16 (.07, .25)	.001	426	.00 (-.11, .11)	.993	331	.16[b]	.00	.85
Verbal peer Perpetration	.36 (.27, .44)	<.001	426	.11 (.00, .22)	.053	331	.36[b]	.00	.64
Cyber peer Perpetration	.18 (.09, .27)	<.001	426	-.04 (-.15, .07)	.482	331	.18[b]	.00	.82
Parental role aspirations	.29 (.20, .37)	<.001	442	.02 (-.09, .13)	.660	337	.29[b]	.00	.71
Occupational role aspirations	.23 (.14, .32)	<.001	442	.10 (-.01, .20)	.067	337	.23[b]	.00	.77
Homecare role aspirations	.46 (.38, .53)	<.001	442	.20 (.10, .30)	<.001	337	.46[b]	.00	.54
Importance of Relationships	.47 (.40, .54)	<.001	510	.06 (-.04, .15)	.199	425	.47[b]	.00	.53
Achievement Motivation	.34 (.26, .41)	<.001	510	.24 (.15, .33)	<.001	425	.20	.14	.66
Purpose in Life	.43 (.36, .50)	<.001	510	.30 (.21, .38)	<.001	425	.26	.17	.57
Importance of democracy and equality	.44 (.37, .51)	<.001	511	.24 (.15, .33)	<.001	423	.40	.04	.56
Environmental concerns	.55 (.49, .61)	<.001	511	.29 (.20, .37)	<.001	425	.54	.02	.45
Religiosity	.74 (.70, .78)	<.001	497	.62 (.56, .68)	<.001	424	.24	.50	.26

Importance of leisure	.38 (.30, .46)	<.001	432	.18 (.07, .28)	.001	331	.38[b]	.00	.63
Alcohol use	.40 (.31, .48)	<.001	369	.22 (.11, .33)	<.001	292	.36	.04	.60
Ever smoked	.48 (.40, .55)	<.001	433	.24 (.14, .34)	<.001	333	.47	.01	.53
Ever vaped	.36 (.28, .44)	<.001	432	.26 (.16, .36)	<.001	332	.20	.16	.64
Cognitive enhancers	.31 (.22, .39)	<.001	420	.17 (.06, .27)	.003	325	.28	.02	.69
Big 5 Agreeableness	.32 (.24, .40)	<.001	483	.10 (.00, .20)	.049	404	.32[b]	.00	.68
Big 5 Conscientiousness	.38 (.30, .45)	<.001	482	.13 (.03, .22)	.009	404	.38[b]	.00	.62
Big 5 Extraversion	.47 (.40, .54)	<.001	485	.15 (.05, .24)	.003	408	.47[b]	.00	.53
Big 5 Neuroticism	.31 (.23, .39)	<.001	485	.20 (.11, .29)	<.001	410	.21	.10	.69
Big 5 Openness	.41 (.33, .48)	<.001	484	.17 (.07, .26)	<.001	407	.41[b]	.00	.59
Self-control	.37 (.29, .44)	<.001	482	.14 (.04, .23)	.006	401	.37[b]	.00	.63
Not planning for the future	.31 (.23, .39)	<.001	481	.13 (.03, .23)	.011	401	.31[b]	.00	.69
Ambition	.37 (.29, .45)	<.001	445	.15 (.05, .25)	.005	344	.37[b]	.00	.63
Physical activity	.41 (.33, .48)	<.001	501	.17 (.08, .26)	.001	416	.41[b]	.00	.59
Health behaviours	.53 (.46, .59)	<.001	504	.25 (.16, .34)	<.001	417	.53[b]	.00	.47
Risk taking behaviour	.53 (.46, .59)	<.001	479	.24 (.15, .33)	<.001	400	.53[b]	.00	.47
Risky sexual behaviour	.61 (.55, .66)	<.001	468	.31 (.22, .40)	<.001	389	.59	.01	.39
Media use	.57 (.51, .63)	<.001	498	.12 (.02, .21)	.014	413	.57[b]	.00	.44
Volunteering	.49 (.42, .55)	<.001	497	.27 (.18, .36)	<.001	424	.44	.05	.51

Internet dating (scale)	.36 (.28, .43)	<.001	498	.19 (.10, .28)	<.001	414	.34	.02	.64
Internet dating (y/n)	.33 (.25, .41)	<.001	499	.21 (.12, .30)	<.001	416	.25	.08	.67
Financial literacy: Knowledge of Products	.48 (.41, .54)	<.001	500	.27 (.18, .36)	<.001	407	.42	.06	.52
Financial attitudes and behaviour	.30 (.22, .38)	<.001	500	.14 (.04, .23)	.004	407	.30[b]	.00	.70
	MZ			DZ[a]			Falconer ACE estimates		
Functional outcome	*r*	*p*	*n*	*r*	*p*	*n*	A	C	E
Wellbeing factor (1st PC)	.34 (.26, .42)	<.001	497	.25 (.16, .34)	<.001	404	.18	.16	.66
Adverse mental health factor (1st PC)	.37 (.27, .46)	<.001	338	.22 (.10, .33)	<.001	271	.29	.08	.64
Adverse physical health factor (1st PC)	.39 (.30, .47)	<.001	383	.13 (.02, .24)	.020	302	.39[b]	.00	.61
Achieved educational level	.40 (.32, .47)	<.001	502	.19 (.10, .28)	<.001	412	.40[b]	.00	.60
Planned educational level	.43 (.32, .53)	<.001	247	.16 (.01, .30)	.033	172	.43[b]	.00	.57

Note. MZ=monozygotic; DZ=dizygotic; and genetic (A), shared environmental (C) and non-shared environmental (E) estimates. Scores were corrected for mean age and sex differences (see Methods). Falconer's ACE estimates were derived from the intraclass twin correlations using Falconer's formula (Rijsdijk & Sham, 2002).

[a] Same-sex male DZ twins.

[b] rMZ reported as A would otherwise exceed rMZ.

(c) females only

	MZ			DZ[a]			Falconer ACE estimates		
Trait	*r*	*p*	*n*	*r*	*p*	*n*	A	C	E
Daily hassles	.32 (.26, .38)	<.001	931	.16 (.09, .23)	<.001	757	.32[b]	.00	.68
CHAOS	.40 (.35, .45)	<.001	1065	.43 (.37, .48)	<.001	873	.00	.40	.60

Childhood experiences	.70 (.67, .73)	<.001	928	.47 (.41, .52)	<.001	752	.46	.24	.30
Poor sleep quality	.33 (.27, .39)	<.001	902	.21 (.14, .28)	<.001	740	.26	.08	.67
Marriage hopes	.34 (.29, .39)	<.001	1038	.23 (.16, .29)	<.001	833	.21	.13	.66
Marriage worries	.38 (.33, .43)	<.001	1038	.24 (.17, .30)	<.001	833	.29	.10	.62
Quality of relationship with twin	.69 (.66, .72)	<.001	1009	.64 (.60, .68)	<.001	813	.09	.60	.32
Quality of relationship with mother	.56 (.52, .60)	<.001	999	.35 (.29, .41)	<.001	799	.41	.15	.45
Quality of relationship with father	.63 (.59, .67)	<.001	961	.47 (.41, .52)	<.001	770	.32	.31	.37
Number of relationships	.44 (.39, .49)	<.001	1036	.26 (.20, .32)	<.001	833	.34	.09	.57
Longest relationship	.34 (.28, .40)	<.001	784	.12 (.04, .20)	.005	600	.34[b]	.00	.66
Partner violence	.25 (.19, .31)	<.001	986	.11 (.04, .18)	.003	794	.25[b]	.00	.75
Contact with mother	.57 (.49, .64)	<.001	294	.34 (.22, .45)	<.001	224	.46	.11	.43
Communication with mother	.67 (.60, .73)	<.001	304	.33 (.21, .44)	<.001	228	.67[b]	.00	.33
Contact with father	.52 (.44, .59)	<.001	352	.45 (.35, .54)	<.001	271	.15	.37	.48
Communication with father	.74 (.69, .78)	<.001	396	.70 (.64, .75)	<.001	298	.08	.66	.26
Peer pressure	.31 (.25, .37)	<.001	999	.15 (.08, .22)	<.001	812	.31[b]	.00	.69
Physical peer victimisation	.09 (.02, .16)	.006	881	.01 (-.06, .08)	.776	712	.09[b]	.00	.91
Social peer victimisation	.29 (.23, .35)	<.001	881	.14 (.07, .21)	<.001	712	.29[b]	.00	.71
Verbal peer victimisation	.32 (.26, .38)	<.001	881	.16 (.09, .23)	<.001	713	.32	.00	.68

Cyber peer victimisation	.37 (.31, .43)	<.001	880	.06 (-.01, .13)	.129	713	.37[b]	.00	.63
Physical peer Perpetration	.08 (.01, .15)	.016	882	-.02 (-.09, .05)	.551	714	.08[b]	.00	.92
Social peer Perpetration	.13 (.06, .19)	<.001	881	.15 (.08, .22)	<.001	714	.00	.13	.87
Verbal peer Perpetration	.27 (.21, .33)	<.001	881	.16 (.09, .23)	<.001	714	.22	.05	.73
Cyber peer Perpetration	.22 (.16, .28)	<.001	881	.14 (.07, .21)	<.001	714	.17	.05	.78
Parental role aspirations	.38 (.32, .43)	<.001	931	.21 (.14, .28)	<.001	754	.33	.04	.63
Occupational role aspirations	.30 (.24, .36)	<.001	932	.15 (.08, .22)	<.001	755	.29	.00	.70
Homecare role aspirations	.39 (.33, .44)	<.001	932	.24 (.17, .31)	<.001	755	.29	.10	.61
Importance of Relationships	.38 (.33, .43)	<.001	1033	.13 (.06, .20)	<.001	840	.38[b]	.00	.63
Achievement Motivation	.38 (.33, .43)	<.001	1033	.15 (.08, .22)	<.001	840	.38[b]	.00	.62
Purpose in Life	.41 (.36, .46)	<.001	1032	.20 (.13, .26)	<.001	839	.41[b]	.00	.59
Importance of democracy and equality	.42 (.37, .47)	<.001	1030	.14 (.07, .21)	<.001	836	.42[b]	.00	.58
Environmental concerns	.49 (.44, .54)	<.001	1031	.30 (.24, .36)	<.001	838	.38	.11	.51
Religiosity	.71 (.68, .74)	<.001	1001	.56 (.51, .61)	<.001	813	.32	.40	.29
Importance of leisure	.43 (.38, .48)	<.001	906	.23 (.16, .30)	<.001	743	.39	.04	.57
Alcohol use	.49 (.43, .54)	<.001	757	.31 (.24, .38)	<.001	624	.34	.14	.52
Ever smoked	.50 (.45, .55)	<.001	909	.23 (.16, .30)	<.001	733	.50[b]	.00	.50
Ever vaped	.36 (.30, .42)	<.001	910	.20 (.13, .27)	<.001	734	.32	.04	.65

Cognitive enhancers	.34 (.28, .40)	<.001	902	.24 (.17, .31)	<.001	725	.20	.14	.66
Big 5 Agreeableness	.24 (.18, .30)	<.001	936	-.02 (-.09, .05)	.643	768	.24[b]	.00	.76
Big 5 Conscientiousness	.32 (.26, .38)	<.001	936	.03 (-.04, .10)	.368	766	.32[b]	.00	.68
Big 5 Extraversion	.43 (.38, .48)	<.001	939	.09 (.02, .16)	.009	771	.43[b]	.00	.58
Big 5 Neuroticism	.40 (.34, .45)	<.001	941	.21 (.14, .28)	<.001	772	.37	.02	.61
Big 5 Openness	.40 (.34, .45)	<.001	937	.10 (.03, .17)	.007	770	.40[b]	.00	.60
Self-control	.41 (.36, .46)	<.001	935	.15 (.08, .22)	<.001	766	.41[b]	.00	.59
Not planning for the future	.22 (.16, .28)	<.001	935	.11 (.04, .18)	.002	766	.22	.00	.78
Ambition	.37 (.31, .42)	<.001	931	.16 (.09, .23)	<.001	757	.37[b]	.00	.63
Physical activity	.40 (.35, .45)	<.001	1027	.21 (.14, .27)	<.001	823	.40	.01	.60
Health behaviours	.55 (.51, .59)	<.001	1029	.34 (.28, .40)	<.001	831	.41	.14	.45
Risk taking behaviour	.45 (.40, .50)	<.001	934	.23 (.16, .30)	<.001	768	.45[b]	.00	.55
Risky sexual behaviour	.61 (.57, .65)	<.001	945	.33 (.26, .39)	<.001	755	.57	.04	.39
Media use	.51 (.46, .55)	<.001	1022	.21 (.14, .27)	<.001	813	.51[b]	.00	.49
Volunteering	.43 (.38, .48)	<.001	1003	.19 (.12, .26)	<.001	814	.43[b]	.00	.57
Internet dating (scale)	.32 (.26, .37)	<.001	1014	.19 (.12, .26)	<.001	811	.27	.05	.68
Internet dating (y/n)	.31 (.25, .36)	<.001	1020	.18 (.11, .25)	<.001	815	.25	.06	.69
Financial literacy: Knowledge of Products	.31 (.25, .37)	<.001	981	.15 (.08, .22)	<.001	777	.31[b]	.00	.69
Financial attitudes and behaviour	.30 (.24, .36)	<.001	980	.08 (.01, .15)	.025	777	.30[b]	.00	.70

Functional outcome	MZ			DZ[a]			Falconer ACE estimates		
	r	*p*	*n*	*r*	*p*	*n*	A	C	E
Wellbeing factor (1st PC)	.41 (.36, .46)	<.001	980	.31 (.24, .37)	<.001	770	.21	.21	.59
Adverse mental health factor (1st PC)	.54 (.49, .59)	<.001	704	.26 (.18, .33)	<.001	584	.54[b]	.00	.46
Adverse physical health factor (1st PC)	.40 (.34, .46)	<.001	851	.25 (.18, .32)	<.001	673	.31	.09	.60
Achieved educational level	.40 (.35, .45)	<.001	1018	.31 (.25, .37)	<.001	816	.17	.23	.60
Planned educational level	.33 (.25, .41)	<.001	488	.14 (.04, .23)	.007	399	.33[b]	.00	.67

Note. MZ=monozygotic; DZ=dizygotic; and genetic (A), shared environmental (C) and non-shared environmental (E) estimates. Scores were corrected for mean age and sex differences (see Methods). Falconer's ACE estimates were derived from the intraclass twin correlations using Falconer's formula (Rijsdijk & Sham, 2002).

[a] Same-sex female DZ twins.

[b] rMZ reported as A would otherwise exceed rMZ.

Table S9. Univariate twin analyses presenting additive genetic (A), shared environmental (C), and non-shared environmental (E) components of variance EA traits and functional outcomes (95% confidence intervals are in parentheses).

Trait	Model fitting results											
	Whole sample			Males only			Females only			Same-sex MZ and DZ twins		
	A	C	E	A	C	E	A	C	E	A	C	E
Daily hassles	.31 (.19,.35)	.00 (.00,.09)	.69 (.65,.74)	.28 (.13,.35)	.00 (.00,.00)	.72 (.65,.79)	.28 (.13,.37)	.03 (.00,.15)	.69 (.63,.74)	.31 (.19,.35)	.00 (.00,.10)	.69 (.65,.74)
CHAOS	.12 (.01,.22)	.30 (.22,.37)	.59 (.55,.63)	.28 (.10,.45)	.13 (.00,.26)	.59 (.52,.66)	.03 (.00,.15)	.39 (.29,.44)	.58 (.54,.62)	.11 (.00,.22)	.31 (.21,.40)	.58 (.54,.62)
Childhood experiences	.46 (.37,.54)	.19 (.12,.27)	.35 (.32,.38)	.50 (.33,.63)	.08 (.00,.20)	.43 (.37,.49)	.40 (.30,.50)	.29 (.19,.37)	.32 (.29,.35)	.39 (.29,.49)	.27 (.17,.36)	.34 (.31,.37)
Poor sleep quality	.27 (.14,.35)	.04 (.00,.14)	.70 (.65,.75)	.23 (.01,.33)	.02 (.00,.18)	.75 (.67,.84)	.27 (.11,.38)	.05 (.00,.18)	.67 (.62,.73)	.16 (.01,.32)	.14 (.01,.27)	.69 (.65,.74)
Marriage hopes	.19 (.07,.31)	.09 (.00,.18)	.72 (.68,.77)	.00 (.00,.21)	.15 (.00,.20)	.85 (.76,.90)	.25 (.11,.38)	.08 (.00,.19)	.67 (.62,.72)	.15 (.01,.29)	.13 (.01,.24)	.72 (.68,.77)

Marriage worries	.37 (.26,.42)	.01 (.00,.10)	.62 (.58,.66)	.36 (.22,.42)	.00 (.00,.10)	.64 (.58,.71)	.31 (.17,.43)	.07 (.00,.18)	.61 (.57,.67)	.32 (.19,.42)	.06 (.00,.17)	.62 (.58,.66)
Quality of relationship with twin	.24 (.19,.30)	.52 (.47,.56)	.24 (.22,.26)	.20 (.11,.29)	.55 (.48,.62)	.24 (.21,.28)	.26 (.19,.34)	.49 (.43,.55)	.24 (.22,.27)	.18 (.12,.25)	.55 (.49,.61)	.27 (.25,.29)
Quality of relationship with mother	.53 (.43,.57)	.00 (.00,.08)	.46 (.43,.50)	.47 (.37,.52)	.00 (.00,.06)	.53 (.48,.60)	.48 (.36,.59)	.08 (.00,.18)	.44 (.40,.48)	.48 (.36,.57)	.06 (.00,.16)	.46 (.43,.49)
Quality of relationship with father	.35 (.27,.44)	.26 (.19,.33)	.39 (.36,.42)	.38 (.22,.53)	.19 (.07,.31)	.43 (.38,.49)	.33 (.23,.43)	.30 (.21,.39)	.37 (.33,.40)	.33 (.23,.44)	.28 (.19,.37)	.38 (.35,.41)
Number of relationships	.45 (.40,.49)	.00 (.00,.00)	.55 (.51,.58)	.46 (.38,.52)	.00 (.00,.05)	.54 (.48,.60)	.44 (.35,.48)	.00 (.00,.00)	.56 (.52,.60)	.44 (.32,.50)	.03 (.00,.13)	.53 (.50,.57)
Longest relationship	.28 (.23,.33)	.00 (.00,.00)	.72 (.67,.76)	.21 (.09,.29)	.00 (.00,.00)	.79 (.71,.87)	.32 (.25,.38)	.00 (.00,.00)	.68 (.62,.73)	.32 (.22,.36)	.00 (.00,.08)	.68 (.64,.73)
Partner violence	.25 (.12,.29)	0 (.00,.10)	.75 (.71,.80)	.27 (.05,.35)	.00 (.00,.00)	.73 (.65,.82)	.24 (.09,.30)	.00 (.00,.12)	.76 (.70,.81)	.25 (.12,.29)	.00 (.00,.10)	.75 (.71,.80)
Contact with mother	.47 (.28,.62)	.10 (.00,.25)	.44 (.38,.50)	.44 (.09,.63)	.09 (.00,.35)	.47 (.37,.61)	.48 (.25,.64)	.10 (.00,.29)	.42 (.36,.49)	.38 (.17,.60)	.19 (.00,.37)	.43 (.38,.50)
Communication with mother	.37 (.21,.54)	.26 (.10,.39)	.37 (.33,.43)	.37 (.08,.66)	.24 (.00,.46)	.39 (.31,.50)	.35 (.15,.57)	.28 (.08,.45)	.37 (.31,.43)	.41 (.21,.63)	.22 (.01,.40)	.37 (.32,.42)
Contact with father	.13 (.00,.30)	.38 (.24,.50)	.49 (.43,.55)	.03 (.00,.33)	.41 (.16,.50)	.56 (.46,.65)	.19 (.00,.39)	.36 (.19,.52)	.45 (.39,.52)	.10 (.00,.29)	.43 (.25,.54)	.48 (.42,.54)
Communication with father	.12 (.02,.21)	.61 (.53,.68)	.28 (.25,.31)	.18 (.00,.38)	.48 (.32,.63)	.33 (.27,.41)	.07 (.00,.18)	.68 (.59,.75)	.25 (.22,.29)	.09 (.00,.20)	.64 (.54,.73)	.27 (.24,.31)
Peer pressure	.35 (.27,.39)	.00 (.00,.05)	.65 (.61,.69)	.40 (.27,.46)	.00 (.00,.09)	.60 (.54,.67)	.31 (.21,.36)	.00 (.00,.08)	.69 (.64,.74)	.35 (.24,.39)	.00 (.00,.09)	.65 (.61,.69)
Physical peer victimisation	.17 (.11,.22)	.00 (.00,.00)	.83 (.78,.88)	.19 (.06,.26)	.00 (.00,.00)	.81 (.74,.88)	.09 (.00,.17)	.00 (.00,.07)	.91 (.83,.99)	.19 (.10,.25)	.00 (.00,.00)	.81 (.75,.86)
Social peer victimisation	.27 (.14,.32)	.00 (.00,.09)	.73 (.68,.78)	.00 (.00,.23)	.17 (.02,.22)	.83 (.73,.88)	.29 (.20,.34)	.00 (.00,.06)	.71 (.66,.77)	.20 (.05,.32)	.07 (.00,.19)	.73 (.68,.79)
Verbal peer victimisation	.30 (.21,.34)	.00 (.00,.06)	.70 (.66,.75)	.18 (.00,.33)	.07 (.00,.22)	.76 (.67,.85)	.32 (.24,.37)	.00 (.00,.00)	.68 (.63,.74)	.30 (.14,.34)	.00 (.00,.13)	.70 (.66,.75)
Cyber peer victimisation	.33 (.26,.37)	.00 (.00,.04)	.67 (.63,.72)	.06 (.00,.29)	.18 (.02,.27)	.75 (.66,.83)	.34 (.29,.40)	.00 (.00,.03)	.66 (.60,.71)	.32 (.25,.37)	.00 (.00,.05)	.68 (.63,.73)
Physical peer Perpetration	.12 (.05,.17)	.00 (.00,.00)	.88 (.83,.93)	.13 (.00,.20)	.00 (.00,.13)	.87 (.80,.95)	.05 (.00,.12)	.00 (.00,.05)	.95 (.88,1.00)	.13 (.00,.21)	.02 (.00,.16)	.85 (.79,.91)
Social peer Perpetration	.15 (.06,.2)	.00 (.00,.06)	.85 (.80,.91)	.15 (.01,.25)	.00 (.00,.00)	.85 (.75,.94)	.14 (.00,.20)	.00 (.00,.11)	.86 (.80,.92)	.14 (.00,.22)	.02 (.00,.15)	.84 (.78,.90)

Verbal peer Perpetration	.31<br>(.26,.36)	.00<br>(.00,.03)	.69<br>(.64,.73)	.32<br>(.19,.38)	.00<br>(.00,.00)	.68<br>(.62,.75)	.29<br>(.21,.35)	.00<br>(.00,.05)	.71<br>(.65,.77)	.32<br>(.21,.36)	.00<br>(.00,.09)	.68<br>(.64,.73)
Cyber peer Perpetration	.19<br>(.13,.24)	0<br>(.00,.03)	.81<br>(.76,.87)	.14<br>(.02,.23)	.00<br>(.00,.00)	.86<br>(.77,.95)	.21<br>(.13,.27)	.00<br>(.00,.06)	.79<br>(.73,.85)	.20<br>(.10,.25)	.00<br>(.00,.08)	.80<br>(.75,.85)
Parental role aspirations	.34<br>(.26,.38)	.00<br>(.00,.06)	.66<br>(.62,.70)	.25<br>(.14,.32)	.00<br>(.00,.00)	.75<br>(.68,.83)	.35<br>(.20,.44)	.03<br>(.00,.15)	.61<br>(.56,.67)	.35<br>(.26,.39)	.00<br>(.00,.00)	.65<br>(.61,.70)
Occupational role aspirations	.26<br>(.18,.3)	.00<br>(.00,.06)	.74<br>(.70,.78)	.20<br>(.06,.27)	.00<br>(.00,.10)	.80<br>(.73,.87)	.29<br>(.16,.34)	.00<br>(.00,.10)	.71<br>(.66,.76)	.27<br>(.13,.32)	.00<br>(.00,.12)	.73<br>(.68,.78)
Homecare role aspirations	.40<br>(.33,.44)	.00<br>(.00,.05)	.60<br>(.56,.64)	.41<br>(.31,.47)	.00<br>(.00,.07)	.59<br>(.53,.65)	.39<br>(.27,.43)	.00<br>(.00,.09)	.61<br>(.57,.66)	.36<br>(.22,.45)	.05<br>(.00,.17)	.59<br>(.55,.63)
Importance of Relationships	.37<br>(.33,.4)	.00<br>(.00,.02)	.63<br>(.60,.67)	.38<br>(.32,.44)	.00<br>(.00,.04)	.62<br>(.56,.68)	.34<br>(.29,.39)	.00<br>(.00,.03)	.66<br>(.61,.71)	.39<br>(.34,.43)	.00<br>(.00,.03)	.61<br>(.57,.65)
Achievement Motivation	.37<br>(.28,.4)	.00<br>(.00,.06)	.63<br>(.60,.67)	.31<br>(.12,.41)	.03<br>(.00,.17)	.66<br>(.59,.74)	.38<br>(.29,.42)	.00<br>(.00,.06)	.62<br>(.58,.67)	.35<br>(.22,.41)	.01<br>(.00,.13)	.63<br>(.59,.68)
Purpose in Life	.42<br>(.31,.46)	.01<br>(.00,.09)	.57<br>(.54,.61)	.39<br>(.21,.49)	.04<br>(.00,.17)	.57<br>(.51,.64)	.42<br>(.30,.47)	.00<br>(.00,.00)	.58<br>(.53,.63)	.37<br>(.25,.46)	.05<br>(.00,.16)	.57<br>(.54,.62)
Importance of democracy and equality	.43<br>(.38,.47)	.00<br>(.00,.03)	.57<br>(.53,.60)	.42<br>(.24,.49)	.02<br>(.00,.15)	.56<br>(.51,.63)	.43<br>(.37,.47)	.00<br>(.00,.00)	.57<br>(.53,.62)	.44<br>(.37,.47)	.00<br>(.00,.05)	.56<br>(.53,.60)
Environmental concerns	.53<br>(.43,.56)	.00<br>(.00,.08)	.47<br>(.44,.51)	.54<br>(.41,.59)	.00<br>(.00,.00)	.46<br>(.41,.52)	.48<br>(.35,.55)	.03<br>(.00,.13)	.49<br>(.45,.54)	.47<br>(.35,.55)	.06<br>(.00,.15)	.48<br>(.45,.51)
Religiosity	.25<br>(.19,.32)	.44<br>(.39,.49)	.31<br>(.28,.33)	.22<br>(.12,.32)	.48<br>(.39,.55)	.30<br>(.26,.34)	.28<br>(.20,.36)	.41<br>(.34,.48)	.31<br>(.28,.34)	.22<br>(.14,.30)	.48<br>(.41,.55)	.30<br>(.28,.32)
Importance of leisure	.41<br>(.31,.45)	.00<br>(.00,.08)	.59<br>(.55,.63)	.38<br>(.17,.45)	.01<br>(.00,.16)	.61<br>(.55,.70)	.42<br>(.30,.47)	.00<br>(.00,.00)	.58<br>(.53,.63)	.40<br>(.26,.46)	.02<br>(.00,.14)	.58<br>(.54,.63)
Alcohol use	.46<br>(.34,.54)	.03<br>(.00,.13)	.51<br>(.46,.55)	.29<br>(.07,.49)	.13<br>(.00,.29)	.58<br>(.50,.66)	.53<br>(.40,.57)	.00<br>(.00,.00)	.47<br>(.43,.52)	.40<br>(.26,.53)	.09<br>(.00,.21)	.51<br>(.47,.56)
Ever smoked	.48<br>(.41,.52)	.00<br>(.00,.06)	.52<br>(.48,.55)	.45<br>(.34,.51)	.00<br>(.00,.08)	.55<br>(.49,.61)	.50<br>(.36,.54)	.00<br>(.00,.11)	.50<br>(.46,.55)	.49<br>(.38,.52)	.00<br>(.00,.00)	.51<br>(.48,.55)
Ever vaped	.33<br>(.21,.42)	.04<br>(.00,.14)	.62<br>(.58,.67)	.35<br>(.14,.44)	.01<br>(.00,.17)	.63<br>(.56,.72)	.30<br>(.15,.43)	.08<br>(.00,.20)	.62<br>(.57,.68)	.28<br>(.13,.41)	.09<br>(.00,.21)	.63<br>(.58,.68)
Cognitive enhancers	.36<br>(.23,.42)	.01<br>(.00,.11)	.63<br>(.58,.68)	.34<br>(.14,.41)	.00<br>(.00,.14)	.66<br>(.59,.75)	.32<br>(.16,.43)	.06<br>(.00,.18)	.62<br>(.57,.68)	.26<br>(.10,.40)	.10<br>(.00,.23)	.64<br>(.59,.69)
Big 5 Agreeableness	.22<br>(.18,.27)	.00<br>(.00,.00)	.78<br>(.73,.82)	.30<br>(.19,.37)	.00<br>(.00,.00)	.70<br>(.63,.77)	.17<br>(.11,.23)	.00<br>(.00,.02)	.83<br>(.77,.89)	.24<br>(.19,.29)	.00<br>(.00,.03)	.76<br>(.71,.80)
Big 5 Conscientiousness	.30<br>(.26,.34)	.00<br>(.00,.02)	.70<br>(.66,.74)	.33<br>(.24,.39)	.00<br>(.00,.04)	.67<br>(.61,.74)	.28<br>(.22,.34)	.00<br>(.00,.00)	.72<br>(.66,.77)	.33<br>(.28,.37)	.00<br>(.00,.03)	.67<br>(.63,.72)

Big 5 Extraversion	.40 (.36,.44)	.00 (.00,.02)	.60 (.56,.64)	.41 (.33,.47)	.00 (.00,.05)	.59 (.53,.65)	.39 (.34,.44)	.00 (.00,.03)	.61 (.56,.66)	.42 (.37,.46)	.00 (.00,.00)	.58 (.54,.62)
Big 5 Neuroticism	.37 (.26,.41)	.00 (.00,.09)	.63 (.59,.67)	.25 (.04,.39)	.07 (.00,.21)	.68 (.61,.77)	.39 (.29,.44)	.00 (.00,.08)	.61 (.56,.66)	.35 (.22,.42)	.03 (.00,.14)	.62 (.58,.67)
Big 5 Openness	.36 (.32,.4)	0 (.00,.02)	.64 (.60,.68)	.37 (.28,.43)	.00 (.00,.06)	.63 (.57,.70)	.36 (.30,.41)	.00 (.00,.00)	.64 (.59,.69)	.38 (.33,.42)	.00 (.00,.04)	.62 (.58,.66)
Self-control	.38 (.34,.42)	.00 (.00,.03)	.62 (.58,.66)	.35 (.24,.41)	.00 (.00,.07)	.65 (.59,.72)	.40 (.34,.45)	.00 (.00,.04)	.60 (.55,.65)	.39 (.34,.44)	.00 (.00,.04)	.61 (.56,.65)
Not planning for the future	.26 (.13,.3)	.00 (.00,.09)	.74 (.70,.79)	.29 (.16,.35)	.00 (.00,.08)	.71 (.65,.78)	.17 (.01,.29)	.06 (.00,.18)	.77 (.71,.84)	.25 (.13,.30)	.00 (.00,.00)	.75 (.70,.80)
Ambition	.36 (.29,.4)	.00 (.00,.05)	.64 (.60,.68)	.34 (.22,.41)	.00 (.00,.00)	.66 (.59,.73)	.37 (.26,.41)	.00 (.00,.08)	.63 (.59,.68)	.37 (.27,.41)	.00 (.00,.08)	.63 (.59,.68)
Physical activity	.39 (.33,.43)	.00 (.00,.05)	.61 (.57,.64)	.39 (.26,.45)	.00 (.00,.09)	.61 (.55,.68)	.40 (.30,.44)	.00 (.00,.08)	.60 (.56,.65)	.40 (.28,.44)	.00 (.00,.10)	.60 (.56,.64)
Health behaviours	.52 (.42,.58)	.03 (.00,.11)	.45 (.42,.48)	.55 (.42,.60)	.00 (.00,.10)	.45 (.40,.51)	.47 (.35,.58)	.08 (.00,.18)	.45 (.41,.49)	.48 (.37,.58)	.07 (.00,.17)	.45 (.42,.48)
Risk taking behaviour	.50 (.42,.53)	.00 (.00,.06)	.50 (.47,.54)	.47 (.30,.56)	.04 (.00,.17)	.49 (.44,.55)	.47 (.40,.52)	.00 (.00,.06)	.53 (.48,.57)	.50 (.39,.53)	.00 (.00,.09)	.50 (.47,.54)
Risky sexual behaviour	.60 (.52,.63)	.00 (.00,.07)	.40 (.37,.43)	.59 (.47,.63)	.00 (.00,.09)	.41 (.37,.47)	.61 (.49,.64)	.00 (.00,.10)	.39 (.36,.43)	.55 (.44,.63)	.06 (.00,.16)	.39 (.36,.43)
Media use	.51 (.48,.55)	.00 (.00,.02)	.49 (.45,.52)	.48 (.42,.53)	.00 (.00,.00)	.52 (.47,.58)	.53 (.47,.57)	.00 (.00,.04)	.47 (.43,.51)	.52 (.48,.56)	.00 (.00,.00)	.48 (.44,.51)
Volunteering	.44 (.36,.47)	.00 (.00,.06)	.56 (.53,.60)	.47 (.34,.53)	.00 (.00,.10)	.53 (.47,.59)	.42 (.32,.46)	.00 (.00,.00)	.58 (.54,.63)	.45 (.32,.48)	.00 (.00,.11)	.55 (.52,.59)
Internet dating (scale)	.34 (.22,.37)	.00 (.00,.09)	.66 (.63,.71)	.36 (.17,.42)	.00 (.00,.14)	.64 (.58,.71)	.32 (.19,.37)	.00 (.00,.11)	.68 (.63,.73)	.30 (.16,.38)	.04 (.00,.15)	.66 (.62,.71)
Internet dating (y/n)	.31 (.23,.35)	.00 (.00,.00)	.69 (.65,.73)	.33 (.15,.39)	.00 (.00,.13)	.67 (.61,.74)	.30 (.20,.35)	.00 (.00,.08)	.70 (.65,.75)	.26 (.12,.36)	.06 (.00,.18)	.68 (.64,.73)
Financial literacy: Knowledge of Products	.37 (.31,.41)	.00 (.00,.00)	.63 (.59,.67)	.44 (.30,.50)	.00 (.00,.11)	.56 (.50,.62)	.31 (.23,.36)	.00 (.00,.00)	.69 (.64,.75)	.38 (.25,.43)	.01 (.00,.12)	.61 (.57,.66)
Financial attitudes and behaviour	.28 (.23,.32)	.00 (.00,.00)	.72 (.68,.76)	.28 (.13,.35)	.00 (.00,.00)	.72 (.65,.79)	.28 (.21,.33)	.00 (.00,.04)	.72 (.67,.77)	.29 (.22,.34)	.00 (.00,.05)	.71 (.66,.75)
	Model fitting results			Males only			Females only			Same-sex MZ and DZ twins		
Functional outcome	A	C	E	A	C	E	A	C	E	A	C	E
Wellbeing factor (1st PC)	.33 (.21,.44)	.07 (.00,.16)	.60 (.56,.64)	.36 (.16,.42)	.00 (.00,.14)	.64 (.58,.72)	.28 (.15,.42)	.14 (.02,.24)	.58 (.53,.63)	.22 (.09,.35)	.18 (.07,.29)	.60 (.56,.64)
Adverse mental health factor (1st PC)	.50 (.42,.54)	.00 (.00,.07)	.50 (.46,.54)	.33 (.09,.49)	.07 (.00,.24)	.60 (.51,.70)	.54 (.46,.59)	.00 (.00,.07)	.46 (.41,.51)	.52 (.37,.56)	.00 (.00,.13)	.48 (.44,.53)

Adverse physical health factor (1st PC)	.39 (.27,.43)	.00 (.00,.10)	.61 (.57,.65)	.37 (.23,.45)	.00 (.00,.10)	.63 (.55,.70)	.36 (.20,.45)	.04 (.00,.17)	.60 (.55,.66)	.32 (.17,.44)	.08 (.00,.21)	.60 (.55,.64)
Achieved educational level	.27 (.16,.38)	.16 (.07,.24)	.57 (.53,.62)	.25 (.07,.43)	.15 (.01,.28)	.60 (.53,.67)	.27 (.14,.41)	.17 (.06,.27)	.56 (.51,.61)	.29 (.16,.42)	.14 (.03,.25)	.57 (.53,.61)
Planned educational level	.40 (.29,.46)	.00 (.00,.08)	.60 (.54,.66)	.41 (.21,.50)	.00 (.00,.16)	.59 (.50,.69)	.39 (.24,.47)	.00 (.00,.12)	.61 (.53,.69)	.43 (.25,.49)	.00 (.00,.16)	.57 (.51,.64)

Note. MZ=monozygotic; DZ=dizygotic; and genetic (A), shared environmental (C) and non-shared environmental (E) estimates. Scores were corrected for mean age and sex differences (see Methods).

Table S10. Model-fit statistics for the univariate twin analyses

Trait	Model fit statistics								
	base	comparison	ep	minus2LL	df	AIC	diffLL	diffdf	p
Daily hassles	Sat	NA	10	203748.14	8555	186638.14	NA	NA	NA
	Sat	ACE	4	24105.26	8561	6983.26	-179642.90	6	1
	ACE	AE	3	24105.26	8562	6981.26	.00	1	1
	ACE	CE	3	24128.72	8562	7004.72	23.46	1	<.001
	ACE	E	2	24293.46	8563	7167.46	188.20	2	<.001
CHAOS	Sat	NA	10	26663.68	9618	7427.68	NA	NA	NA
	Sat	ACE	4	26673.53	9624	7425.53	9.85	6	.13
	ACE	AE	3	26728.84	9625	7478.84	55.31	1	<.001
	ACE	CE	3	26678.52	9625	7428.52	4.99	1	.03
	ACE	E	2	2729.57	9626	8038.57	617.04	2	<.001
Childhood experiences	Sat	NA	10	23058.40	8547	5964.40	NA	NA	NA
	Sat	ACE	4	2308.59	8553	5974.59	22.19	6	<.001
	ACE	AE	3	23103.77	8554	5995.77	23.18	1	<.001
	ACE	CE	3	23187.71	8554	6079.71	107.12	1	<.001
	ACE	E	2	24216.00	8555	7106.01	1135.41	2	<.001
Poor sleep quality	Sat	NA	10	199518.65	8396	182726.65	NA	NA	NA
	Sat	ACE	4	23635.06	8402	6831.06	-175883.60	6	1
	ACE	AE	3	23635.54	8403	6829.54	.47	1	.49
	ACE	CE	3	23651.37	8403	6845.37	16.30	1	<.001

	ACE	E	2	2382.84	8404	7012.84	185.78	2	<.001
Marriage hopes	Sat	NA	10	103445.93	9470	84505.93	NA	NA	NA
	Sat	ACE	4	26698.55	9476	7746.55	-76747.38	6	1
	ACE	AE	3	26702.42	9477	7748.42	3.87	1	.05
	ACE	CE	3	26708.06	9477	7754.06	9.51	1	<.001
	ACE	E	2	2690.73	9478	7944.73	202.18	2	<.001
Marriage worries	Sat	NA	10	86643.00	9470	67703.00	NA	NA	NA
	Sat	ACE	4	26591.80	9476	7639.80	-60051.20	6	1
	ACE	AE	3	26591.88	9477	7637.88	.08	1	.78
	ACE	CE	3	26632.14	9477	7678.14	4.34	1	<.001
	ACE	E	2	26922.15	9478	7966.15	33.35	2	<.001
Quality of relationship with twin	Sat	NA	10	23541.58	9285	4971.58	NA	NA	NA
	Sat	ACE	4	24035.00	9291	5453.00	493.42	6	<.001
	ACE	AE	3	24323.76	9292	5739.76	288.76	1	<.001
	ACE	CE	3	24107.12	9292	5523.12	72.12	1	<.001
	ACE	E	2	26367.97	9293	7781.97	2332.97	2	<.001
Quality of relationship with mother	Sat	NA	10	25477.43	9202	7073.43	NA	NA	NA
	Sat	ACE	4	25483.13	9208	7067.13	5.70	6	.46
	ACE	AE	3	25483.13	9209	7065.13	.00	1	.94
	ACE	CE	3	25584.97	9209	7166.97	101.85	1	<.001
	ACE	E	2	26132.29	9210	7712.29	649.16	2	<.001
Quality of relationship with father	Sat	NA	10	24415.63	8979	6457.63	NA	NA	NA
	Sat	ACE	4	24431.95	8985	6461.95	16.32	6	.01
	ACE	AE	3	24476.15	8986	6504.15	44.20	1	<.001
	ACE	CE	3	24493.95	8986	6521.95	62.00	1	<.001
	ACE	E	2	25498.44	8987	7524.45	1066.50	2	<.001
Number of relationships	Sat	NA	10	92061.68	9468	73125.68	NA	NA	NA
	Sat	ACE	4	26426.43	9474	7478.43	-65635.25	6	1
	ACE	AE	3	26426.43	9475	7476.43	.00	1	1
	ACE	CE	3	26522.11	9475	7572.11	95.68	1	<.001

	ACE	E	2	26876.34	9476	7924.34	449.92	2	<.001
Longest relationship	Sat	NA	10	210814.72	7958	194898.72	NA	NA	NA
	Sat	ACE	4	22501.62	7964	6573.62	-188313.10	6	1
	ACE	AE	3	22501.62	7965	6571.62	.00	1	1
	ACE	CE	3	22537.70	7965	6607.70	36.08	1	<.001
	ACE	E	2	22623.52	7966	6691.52	121.90	2	<.001
Partner violence	Sat	NA	10	232812.01	9156	21450.01	NA	NA	NA
	Sat	ACE	4	25889.35	9162	7565.35	-206922.70	6	1
	ACE	AE	3	25889.35	9163	7563.35	.00	1	.95
	ACE	CE	3	25903.32	9163	7577.32	13.97	1	<.001
	ACE	E	2	26013.51	9164	7685.51	124.16	2	<.001
Contact with mother	Sat	NA	10	10855.31	3891	3073.31	NA	NA	NA
	Sat	ACE	4	10859.30	3897	3065.30	3.98	6	.68
	ACE	AE	3	1086.69	3898	3064.70	1.40	1	.24
	ACE	CE	3	10882.63	3898	3086.63	23.33	1	<.001
	ACE	E	2	11071.70	3899	3273.70	212.40	2	<.001
Communication with mother	Sat	NA	10	10973.81	3976	3021.81	NA	NA	NA
	Sat	ACE	4	1099.62	3982	3026.62	16.81	6	.01
	ACE	AE	3	1100.65	3983	3034.65	1.03	1	<.001
	ACE	CE	3	1101.62	3983	3044.62	2.00	1	<.001
	ACE	E	2	11315.30	3984	3347.30	324.68	2	<.001
Contact with father	Sat	NA	10	11723.09	4223	3277.09	NA	NA	NA
	Sat	ACE	4	11726.58	4229	3268.58	3.49	6	.75
	ACE	AE	3	11752.19	4230	3292.19	25.61	1	<.001
	ACE	CE	3	11729.11	4230	3269.11	2.53	1	.11
	ACE	E	2	12002.87	4231	354.87	276.29	2	<.001
Communication with father	Sat	NA	10	12312.96	4624	3064.96	NA	NA	NA
	Sat	ACE	4	1232.26	4630	306.26	7.30	6	.29
	ACE	AE	3	12446.82	4631	3184.82	126.57	1	<.001
	ACE	CE	3	12326.09	4631	3064.09	5.83	1	.02

	ACE	E	2	13146.29	4632	3882.29	826.03	2	<.001
Peer pressure	Sat	NA	10	221899.64	9371	203157.64	NA	NA	NA
	Sat	ACE	4	26349.03	9377	7595.03	-19555.60	6	1
	ACE	AE	3	26349.03	9378	7593.03	.00	1	1
	ACE	CE	3	26392.15	9378	7636.15	43.13	1	<.001
	ACE	E	2	26599.08	9379	7841.08	25.06	2	<.001
Physical peer victimisation	Sat	NA	10	271563.35	8290	254983.35	NA	NA	NA
	Sat	ACE	4	23449.11	8296	6857.11	-248114.23	6	1
	ACE	AE	3	23449.11	8297	6855.11	.00	1	1
	ACE	CE	3	23464.92	8297	687.92	15.81	1	<.001
	ACE	E	2	23492.48	8298	6896.48	43.36	2	<.001
Social peer victimisation	Sat	NA	10	199948.06	8289	18337.06	NA	NA	NA
	Sat	ACE	4	23404.92	8295	6814.92	-176543.10	6	1
	ACE	AE	3	23404.92	8296	6812.92	.00	1	1
	ACE	CE	3	2342.37	8296	6828.37	15.46	1	<.001
	ACE	E	2	23524.93	8297	693.93	12.01	2	<.001
Verbal peer victimisation	Sat	NA	10	199897.34	8288	183321.34	NA	NA	NA
	Sat	ACE	4	23403.06	8294	6815.06	-176494.28	6	1
	ACE	AE	3	23403.06	8295	6813.06	.00	1	1
	ACE	CE	3	23429.65	8295	6839.65	26.59	1	<.001
	ACE	E	2	23552.61	8296	696.61	149.56	2	<.001
Cyber peer victimisation	Sat	NA	10	20792.94	8287	191346.94	NA	NA	NA
	Sat	ACE	4	23387.41	8293	6801.41	-184533.50	6	1
	ACE	AE	3	23387.41	8294	6799.41	.00	1	1
	ACE	CE	3	23425.32	8294	6837.33	37.91	1	<.001
	ACE	E	2	23558.45	8295	6968.45	171.04	2	<.001
Physical peer Perpetration	Sat	NA	10	288891.57	8288	272315.57	NA	NA	NA
	Sat	ACE	4	23459.54	8294	6871.54	-265432.00	6	1
	ACE	AE	3	23459.54	8295	6869.54	.00	1	1
	ACE	CE	3	23467.76	8295	6877.76	8.22	1	<.001

	ACE	E	2	23481.27	8296	6889.27	21.74	2	<.001
Social peer Perpetration	Sat	NA	10	252019.20	8286	235447.20	NA	NA	NA
	Sat	ACE	4	23455.67	8292	6871.67	-228563.50	6	1
	ACE	AE	3	23455.67	8293	6869.67	.00	1	1
	ACE	CE	3	23463.30	8293	6877.30	7.63	1	.01
	ACE	E	2	23485.42	8294	6897.42	29.75	2	<.001
Verbal peer Perpetration	Sat	NA	10	92029.05	8287	75455.05	NA	NA	NA
	Sat	ACE	4	23404.15	8293	6818.15	-68624.90	6	1
	ACE	AE	3	23404.15	8294	6816.15	.00	1	1
	ACE	CE	3	23443.75	8294	6855.75	39.60	1	<.001
	ACE	E	2	23564.36	8295	6974.36	16.21	2	<.001
Cyber peer Perpetration	Sat	NA	10	257365.80	8287	240791.80	NA	NA	NA
	Sat	ACE	4	23514.86	8293	6928.86	-23385.90	6	1
	ACE	AE	3	23514.86	8294	6926.86	.00	1	1
	ACE	CE	3	23533.88	8294	6945.88	19.02	1	<.001
	ACE	E	2	23562.62	8295	6972.62	47.77	2	<.001
Parental role aspirations	Sat	NA	10	188961.60	8565	171831.60	NA	NA	NA
	Sat	ACE	4	24085.84	8571	6943.84	-164875.80	6	1
	ACE	AE	3	24085.84	8572	6941.84	.00	1	1
	ACE	CE	3	24124.17	8572	698.17	38.33	1	<.001
	ACE	E	2	2431.62	8573	7164.62	224.78	2	<.001
Occupational role aspirations	Sat	NA	10	213057.49	8570	195917.49	NA	NA	NA
	Sat	ACE	4	24217.93	8576	7065.93	-188839.60	6	1
	ACE	AE	3	24217.93	8577	7063.93	.00	1	1
	ACE	CE	3	2424.52	8577	7086.52	22.59	1	<.001
	ACE	E	2	24347.02	8578	7191.02	129.09	2	<.001
Homecare role aspirations	Sat	NA	10	97251.28	8570	80111.28	NA	NA	NA
	Sat	ACE	4	24007.24	8576	6855.24	-73244.04	6	1
	ACE	AE	3	24007.24	8577	6853.24	.00	1	1
	ACE	CE	3	24066.39	8577	6912.39	59.15	1	<.001

	ACE	E	2	24326.41	8578	717.41	319.17	2	<.001
Importance of Relationships	Sat	NA	10	243327.33	9572	224183.33	NA	NA	NA
	Sat	ACE	4	26886.81	9578	773.81	-21644.50	6	1
	ACE	AE	3	26886.81	9579	7728.81	.00	1	1
	ACE	CE	3	26977.15	9579	7819.15	9.34	1	<.001
	ACE	E	2	2717.85	9580	801.86	284.05	2	<.001
Achievement Motivation	Sat	NA	10	102552.96	9571	8341.96	NA	NA	NA
	Sat	ACE	4	26913.42	9577	7759.42	-75639.54	6	1
	ACE	AE	3	26913.42	9578	7757.42	.00	1	1
	ACE	CE	3	26959.43	9578	7803.43	46.01	1	<.001
	ACE	E	2	27209.09	9579	8051.09	295.68	2	<.001
Purpose in Life	Sat	NA	10	26721.08	9564	7593.08	NA	NA	NA
	Sat	ACE	4	26729.33	9570	7589.33	8.25	6	.22
	ACE	AE	3	26729.36	9571	7587.36	.03	1	.85
	ACE	CE	3	26783.73	9571	7641.73	54.40	1	<.001
	ACE	E	2	27138.07	9572	7994.07	408.74	2	<.001
Importance of democracy and equality	Sat	NA	10	92815.68	9545	73725.68	NA	NA	NA
	Sat	ACE	4	26708.38	9551	7606.38	-66107.31	6	1
	ACE	AE	3	26708.38	9552	7604.38	.00	1	1
	ACE	CE	3	26792.29	9552	7688.29	83.92	1	<.001
	ACE	E	2	27097.20	9553	7991.20	388.83	2	<.001
Environmental concerns	Sat	NA	10	26476.05	9556	7364.05	NA	NA	NA
	Sat	ACE	4	26486.36	9562	7362.36	1.31	6	.11
	ACE	AE	3	26486.36	9563	736.36	.00	1	.98
	ACE	CE	3	26588.83	9563	7462.83	102.47	1	<.001
	ACE	E	2	27134.74	9564	8006.74	648.38	2	<.001
Religiosity	Sat	NA	10	24543.75	9381	5781.75	NA	NA	NA
	Sat	ACE	4	24577.91	9387	5803.91	34.15	6	<.001
	ACE	AE	3	24764.16	9388	5988.16	186.25	1	<.001
	ACE	CE	3	24636.37	9388	586.37	58.46	1	<.001

	ACE	E	2	26598.44	9389	782.44	202.54	2	<.001
Importance of leisure	Sat	NA	10	81758.02	8392	64974.02	NA	NA	NA
	Sat	ACE	4	23518.89	8398	6722.89	-58239.13	6	1
	ACE	AE	3	23518.89	8399	672.89	.00	1	1
	ACE	CE	3	23567.01	8399	6769.01	48.12	1	<.001
	ACE	E	2	2384.90	8400	704.90	322.01	2	<.001
Alcohol use	Sat	NA	10	21462.52	7702	6058.52	NA	NA	NA
	Sat	ACE	4	21476.33	7708	606.33	13.80	6	.03
	ACE	AE	3	21476.80	7709	6058.80	.47	1	.49
	ACE	CE	3	21527.10	7709	6109.10	5.77	1	<.001
	ACE	E	2	21861.45	7710	6441.45	385.12	2	<.001
Ever smoked	Sat	NA	10	2343.90	8424	6582.90	NA	NA	NA
	Sat	ACE	4	23444.20	8430	6584.20	13.30	6	.04
	ACE	AE	3	23444.20	8431	6582.20	.00	1	1
	ACE	CE	3	23527.94	8431	6665.94	83.74	1	<.001
	ACE	E	2	2393.92	8432	7066.92	486.71	2	<.001
Ever vaped	Sat	NA	10	23634.84	8420	6794.84	NA	NA	NA
	Sat	ACE	4	23644.76	8426	6792.77	9.92	6	.13
	ACE	AE	3	23645.58	8427	6791.58	.81	1	.37
	ACE	CE	3	23671.06	8427	6817.06	26.30	1	<.001
	ACE	E	2	23911.70	8428	7055.70	266.93	2	<.001
Cognitive enhancers	Sat	NA	10	94272.52	8338	77596.52	NA	NA	NA
	Sat	ACE	4	23486.58	8344	6798.58	-70785.94	6	1
	ACE	AE	3	23486.62	8345	6796.62	.04	1	.84
	ACE	CE	3	23514.74	8345	6824.74	28.15	1	<.001
	ACE	E	2	2371.52	8346	7018.52	223.94	2	<.001
Big 5 Agreeableness	Sat	NA	10	280057.95	9277	261503.95	NA	NA	NA
	Sat	ACE	4	26268.86	9283	7702.87	-253789.10	6	1
	ACE	AE	3	26268.86	9284	770.87	.00	1	1
	ACE	CE	3	26305.49	9284	7737.49	36.62	1	<.001

	ACE	E	2	26356.65	9285	7786.65	87.79	2	<.001
Big 5 Conscientiousness	Sat	NA	10	25000.84	9264	231472.84	NA	NA	NA
	Sat	ACE	4	26102.90	9270	7562.90	-223897.94	6	1
	ACE	AE	3	26102.90	9271	756.90	.00	1	1
	ACE	CE	3	26163.02	9271	7621.02	6.12	1	<.001
	ACE	E	2	26266.96	9272	7722.96	164.06	2	<.001
Big 5 Extraversion	Sat	NA	10	225376.56	9314	206748.56	NA	NA	NA
	Sat	ACE	4	26115.69	9320	7475.69	-19926.87	6	1
	ACE	AE	3	26115.69	9321	7473.69	.00	1	1
	ACE	CE	3	26208.45	9321	7566.45	92.76	1	<.001
	ACE	E	2	26442.47	9322	7798.47	326.78	2	<.001
Big 5 Neuroticism	Sat	NA	10	98332.34	9327	79678.34	NA	NA	NA
	Sat	ACE	4	26209.03	9333	7543.03	-72123.32	6	1
	ACE	AE	3	26209.03	9334	7541.03	.00	1	1
	ACE	CE	3	26246.70	9334	7578.70	37.67	1	<.001
	ACE	E	2	2649.22	9335	782.22	281.20	2	<.001
Big 5 Openness	Sat	NA	10	230214.10	9298	211618.10	NA	NA	NA
	Sat	ACE	4	26155.91	9304	7547.91	-204058.20	6	1
	ACE	AE	3	26155.91	9305	7545.91	.00	1	1
	ACE	CE	3	26231.21	9305	7621.21	75.30	1	<.001
	ACE	E	2	2642.55	9306	7808.55	264.64	2	<.001
Self-control	Sat	NA	10	225125.98	9256	206613.98	NA	NA	NA
	Sat	ACE	4	25998.61	9262	7474.61	-199127.40	6	1
	ACE	AE	3	25998.61	9263	7472.61	.00	1	1
	ACE	CE	3	26069.58	9263	7543.58	7.97	1	<.001
	ACE	E	2	26282.19	9264	7754.19	283.57	2	<.001
Not planning for the future	Sat	NA	10	230139.14	9248	211643.14	NA	NA	NA
	Sat	ACE	4	26123.01	9254	7615.02	-204016.10	6	1
	ACE	AE	3	26123.02	9255	7613.02	.00	1	.98
	ACE	CE	3	26138.49	9255	7628.49	15.47	1	<.001

	ACE	E	2	26253.87	9256	7741.87	13.85	2	<.001
Ambition	Sat	NA	10	188875.72	8558	171759.72	NA	NA	NA
	Sat	ACE	4	24077.37	8564	6949.37	-164798.30	6	1
	ACE	AE	3	24077.37	8565	6947.37	.00	1	1
	ACE	CE	3	24124.24	8565	6994.24	46.88	1	<.001
	ACE	E	2	2433.38	8566	7198.38	253.01	2	<.001
Physical activity	Sat	NA	10	10281.52	9351	84108.52	NA	NA	NA
	Sat	ACE	4	26218.73	9357	7504.73	-76591.79	6	1
	ACE	AE	3	26218.73	9358	7502.73	.00	1	1
	ACE	CE	3	26278.41	9358	7562.41	59.67	1	<.001
	ACE	E	2	2656.54	9359	7842.54	341.80	2	<.001
Health behaviours	Sat	NA	10	25919.48	9384	7151.48	NA	NA	NA
	Sat	ACE	4	2592.65	9390	714.65	1.17	6	.98
	ACE	AE	3	25921.42	9391	7139.42	.77	1	.38
	ACE	CE	3	26026.43	9391	7244.43	105.78	1	<.001
	ACE	E	2	26663.04	9392	7879.04	742.39	2	<.001
Risk taking behaviour	Sat	NA	10	25711.05	9245	7221.05	NA	NA	NA
	Sat	ACE	4	25737.09	9251	7235.09	26.04	6	<.001
	ACE	AE	3	25737.09	9252	7233.09	.00	1	1
	ACE	CE	3	25825.71	9252	7321.71	88.62	1	<.001
	ACE	E	2	2624.64	9253	7734.64	503.55	2	<.001
Risky sexual behaviour	Sat	NA	10	24751.96	9021	6709.96	NA	NA	NA
	Sat	ACE	4	24767.85	9027	6713.85	15.88	6	.01
	ACE	AE	3	24767.85	9028	6711.85	.00	1	1
	ACE	CE	3	24923.44	9028	6867.45	155.60	1	<.001
	ACE	E	2	25616.64	9029	7558.64	848.80	2	<.001
Media use	Sat	NA	10	90582.66	9300	71982.66	NA	NA	NA
	Sat	ACE	4	25809.09	9306	7197.09	-64773.57	6	1
	ACE	AE	3	25809.09	9307	7195.09	.00	1	1
	ACE	CE	3	25967.72	9307	7353.72	158.63	1	<.001

	ACE	E	2	26382.51	9308	7766.51	573.42	2	<.001
Volunteering	Sat	NA	10	85527.56	9389	66749.56	NA	NA	NA
	Sat	ACE	4	26225.78	9395	7435.78	-59301.78	6	1
	ACE	AE	3	26225.78	9396	7433.78	.00	1	1
	ACE	CE	3	26296.77	9396	7504.77	7.99	1	<.001
	ACE	E	2	26663.54	9397	7869.54	437.76	2	<.001
Internet dating	Sat	NA	10	183925.32	9270	165385.32	NA	NA	NA
(scale)	Sat	ACE	4	26077.49	9276	7525.49	-157847.80	6	1
	ACE	AE	3	26077.49	9277	7523.49	.00	1	1
	ACE	CE	3	26108.88	9277	7554.88	31.39	1	<.001
	ACE	E	2	26325.62	9278	7769.62	248.14	2	<.001
Internet dating	Sat	NA	10	222561.62	9301	203959.62	NA	NA	NA
(y/n)	Sat	ACE	4	26203.83	9307	7589.83	-196357.80	6	1
	ACE	AE	3	26203.83	9308	7587.83	.00	1	1
	ACE	CE	3	26237.29	9308	7621.30	33.46	1	<.001
	ACE	E	2	26415.83	9309	7797.84	212.00	2	<.001
Financial literacy: Knowledge of Products	Sat	NA	10	215394.11	9144	197106.11	NA	NA	NA
	Sat	ACE	4	25678.98	9150	7378.98	-189715.13	6	1
	ACE	AE	3	25678.98	9151	7376.98	.00	1	1
	ACE	CE	3	25733.55	9151	7431.55	54.57	1	<.001
	ACE	E	2	25955.41	9152	7651.41	276.43	2	<.001
Financial attitudes and behaviour	Sat	NA	10	234826.91	9142	216542.91	NA	NA	NA
	Sat	ACE	4	25828.75	9148	7532.75	-208998.20	6	1
	ACE	AE	3	25828.75	9149	753.75	.00	1	1
	ACE	CE	3	25869.23	9149	7571.23	4.48	1	<.001
	ACE	E	2	2598.04	9150	768.04	151.29	2	<.001
	Model fit statistics								
Functional outcome	base	comparison	ep	minus2LL	df	AIC	diffLL	diffdf	p
Wellbeing factor (1st PC)	Sat	NA	10	25528.59	9122	7284.59	NA	NA	NA
	Sat	ACE	4	25529.45	9128	7273.45	.86	6	.99

	ACE	AE	3	25532.21	9129	7274.21	2.76	1	.1
	ACE	CE	3	2556.23	9129	7302.23	3.78	1	<.001
	ACE	E	2	25898.17	9130	7638.17	368.72	2	<.001
Adverse mental health factor (1st PC)	Sat	NA	10	20002.03	7168	5666.03	NA	NA	NA
	Sat	ACE	4	20006.85	7174	5658.85	4.82	6	.57
	ACE	AE	3	20006.85	7175	5656.85	.00	1	1
	ACE	CE	3	20074.95	7175	5724.95	68.10	1	<.001
	ACE	E	2	20376.06	7176	6024.06	369.21	2	<.001
Adverse physical health factor (1st PC)	Sat	NA	10	95542.78	7714	80114.78	NA	NA	NA
	Sat	ACE	4	21596.43	7720	6156.43	-73946.35	6	1
	ACE	AE	3	21596.43	7721	6154.43	.00	1	1
	ACE	CE	3	21631.35	7721	6189.35	34.92	1	<.001
	ACE	E	2	21867.07	7722	6423.07	27.65	2	<.001
Achieved educational level	Sat	NA	10	26008.17	9324	736.17	NA	NA	NA
	Sat	ACE	4	26012.79	9330	7352.79	4.62	6	.59
	ACE	AE	3	26025.90	9331	7363.90	13.11	1	<.001
	ACE	CE	3	26035.29	9331	7373.29	22.51	1	<.001
	ACE	E	2	26472.93	9332	7808.93	46.14	2	<.001
Planned educational level	Sat	NA	10	155685.64	6119	143447.64	NA	NA	NA
	Sat	ACE	4	17254.87	6125	5004.87	-13843.80	6	1
	ACE	AE	3	17254.87	6126	5002.87	.00	1	1
	ACE	CE	3	17281.48	6126	5029.48	26.60	1	<.001
	ACE	E	2	17379.29	6127	5125.29	124.42	2	<.001

Note. The ACE model was included for completeness even though in some cases more parsimonious models show better fit; ep= estimated parameters; -2LL= minus 2 log-likelihood; df= degrees of freedom; AIC= Akaike's information criterion; diffLL= log-likelihood-ratio χ^2 test comparing models to base; diffdf= difference in degrees of freedom; Sat= Saturated model representing the perfect fit; ACE= the full ACE model decomposing the phenotypic variation into genetic, common environmental and unique environmental influences; AE= a sub-model decomposing the phenotypic variation into genetic and unique environmental influences; CE= a sub-model decomposing the phenotypic variation into shared and unique environmental influences; E= a sub-model explaining the phenotypic variation to be accounted for by unique environmental influences; p= probability value of the model fit.

Table S11. Model-fit statistics for the sex limitation twin analyses.

Trait	Model fit statistics								
	base	comparison	ep	minus2LL	df	AIC	diffLL	diffdf	p
Daily hassles	FullHetACE		9	24102.23	8556	699.23			
	FullHetACE	HetACE	8	24102.23	8557	6988.23	-5.46E-11	1	1.00
	cFullHetACE	HetACE	8	24102.23	8557	6988.23	-1.06E+02	1	1.00
	HetACE	HomACE	5	24104.31	8560	6984.31	2.08E+00	3	.56
CHAOS	FullHetACE		9	26652.85	9619	7414.85			
	FullHetACE	HetACE	8	26652.85	9620	7412.85	.00	1	1.00
	cFullHetACE	HetACE	8	26652.85	9620	7412.85	-32.89	1	1.00
	HetACE	HomACE	5	26671.55	9623	7425.55	18.70	3	.00
Childhood experiences	FullHetACE		9	23022.83	8548	5926.83			
	FullHetACE	HetACE	8	23031.32	8549	5933.32	8.49	1	.00
	cFullHetACE	HetACE	8	23031.32	8549	5933.32	8.49	1	.00
	HetACE	HomACE	5	2308.42	8552	5976.42	49.10	3	.00
Poor sleep quality	FullHetACE		9	23605.06	8397	6811.06			
	FullHetACE	HetACE	8	23605.06	8398	6809.06	.00	1	1.00
	cFullHetACE	HetACE	8	23605.06	8398	6809.06	-78.49	1	1.00
	HetACE	HomACE	5	23634.52	8401	6832.52	29.46	3	.00
Marriage hopes	FullHetACE		9	26681.07	9471	7739.07			
	FullHetACE	HetACE	8	26681.07	9472	7737.07	.00	1	1.00
	cFullHetACE	HetACE	8	26681.07	9472	7737.07	-124.78	1	1.00
	HetACE	HomACE	5	26697.35	9475	7747.35	16.28	3	.00
Marriage worries	FullHetACE		9	26579.92	9471	7637.92			
	FullHetACE	HetACE	8	26582.95	9472	7638.95	3.03	1	.08
	cFullHetACE	HetACE	8	26582.95	9472	7638.95	3.03	1	.08
	HetACE	HomACE	5	26591.19	9475	7641.19	8.24	3	.04
Quality of relationship with twin	FullHetACE		9	2402.01	9286	5448.01			
	FullHetACE	HetACE	8	2402.01	9287	5446.01	.00	1	1.00
	cFullHetACE	HetACE	8	2402.01	9287	5446.01	-359.54	1	1.00

	HetACE	HomACE	5	24029.5	9290	5449.50	9.49	3	.02
Quality of relationship with mother	FullHetACE		9	25457.82	9203	7051.82			
	FullHetACE	HetACE	8	25463.85	9204	7055.85	6.03	1	.01
	cFullHetACE	HetACE	8	25463.85	9204	7055.85	6.03	1	.01
	HetACE	HomACE	5	25481.03	9207	7067.03	17.18	3	.00
Quality of relationship with father	FullHetACE		9	24408.14	8980	6448.14			
	FullHetACE	HetACE	8	2441.35	8981	6448.35	2.21	1	.14
	cFullHetACE	HetACE	8	2441.35	8981	6448.35	2.21	1	.14
	HetACE	HomACE	5	24428.36	8984	646.36	18.01	3	.00
Number of relationships	FullHetACE		9	2636.79	9469	7422.79			
	FullHetACE	HetACE	8	26373.2	9470	7433.20	12.41	1	.00
	cFullHetACE	HetACE	8	26373.2	9470	7433.20	-19.85	1	1.00
	HetACE	HomACE	5	26424.98	9473	7478.98	51.78	3	.00
Longest relationship	FullHetACE		9	22486.62	7959	6568.62			
	FullHetACE	HetACE	8	22486.62	7960	6566.62	.00	1	1.00
	cFullHetACE	HetACE	8	22486.62	7960	6566.62	-118.88	1	1.00
	HetACE	HomACE	5	22498.99	7963	6572.99	12.37	3	.01
Partner violence	FullHetACE		9	25823.2	9157	7509.20			
	FullHetACE	HetACE	8	25823.2	9158	7507.20	.00	1	1.00
	cFullHetACE	HetACE	8	25823.2	9158	7507.20	-39.54	1	1.00
	HetACE	HomACE	5	25888.67	9161	7566.67	65.47	3	.00
Contact with mother	FullHetACE		9	10833.96	3892	3049.96			
	FullHetACE	HetACE	8	10833.96	3893	3047.96	.00	1	1.00
	cFullHetACE	HetACE	8	10833.96	3893	3047.96	.00	1	1.00
	HetACE	HomACE	5	10857.57	3896	3065.57	23.61	3	.00
Communication with mother	FullHetACE		9	10982.59	3977	3028.59			
	FullHetACE	HetACE	8	10982.59	3978	3026.59	.00	1	1.00
	cFullHetACE	HetACE	8	10982.59	3978	3026.59	-46.96	1	1.00
	HetACE	HomACE	5	1099.61	3981	3028.62	8.02	3	.05
Contact with father	FullHetACE		9	11709.96	4224	3261.97			

	FullHetACE	HetACE	8	11709.96	4225	3259.97	.00	1	1.00
	cFullHetACE	HetACE	8	11709.96	4225	3259.97	-6.14	1	1.00
	HetACE	HomACE	5	11724.97	4228	3268.97	15.01	3	.00
Communication with father	FullHetACE		9	12305.5	4625	3055.50			
	FullHetACE	HetACE	8	12305.5	4626	3053.50	.00	1	1.00
	cFullHetACE	HetACE	8	12305.5	4626	3053.50	-133.46	1	1.00
	HetACE	HomACE	5	12319.83	4629	3061.83	14.33	3	.00
Peer pressure	FullHetACE		9	26332.77	9372	7588.77			
	FullHetACE	HetACE	8	26333.91	9373	7587.91	1.13	1	.29
	cFullHetACE	HetACE	8	26333.91	9373	7587.91	-12.47	1	1.00
	HetACE	HomACE	5	26348.63	9376	7596.63	14.72	3	.00
Physical peer victimisation	FullHetACE		9	22545.92	8291	5963.92			
	FullHetACE	HetACE	8	2255.06	8292	5966.06	4.14	1	.04
	cFullHetACE	HetACE	8	2255.06	8292	5966.06	.00	1	1.00
	HetACE	HomACE	5	23447.31	8295	6857.31	897.25	3	.00
Social peer victimisation	FullHetACE		9	23387.85	8290	6807.85			
	FullHetACE	HetACE	8	23387.85	8291	6805.85	.00	1	1.00
	cFullHetACE	HetACE	8	23387.85	8291	6805.85	-72.13	1	1.00
	HetACE	HomACE	5	23404.91	8294	6816.92	17.06	3	.00
Verbal peer victimisation	FullHetACE		9	23354.95	8289	6776.95			
	FullHetACE	HetACE	8	23355.37	8290	6775.37	.41	1	.52
	cFullHetACE	HetACE	8	23355.37	8290	6775.37	-132.86	1	1.00
	HetACE	HomACE	5	23402.92	8293	6816.92	47.56	3	.00
Cyber peer victimisation	FullHetACE		9	23381.03	8288	6805.03			
	FullHetACE	HetACE	8	23381.03	8289	6803.03	.00	1	1.00
	cFullHetACE	HetACE	8	23381.03	8289	6803.03	-85.63	1	1.00
	HetACE	HomACE	5	23387.39	8292	6803.39	6.36	3	.10
Physical peer Perpetration	FullHetACE		9	21949.96	8289	5371.96			
	FullHetACE	HetACE	8	2195.37	8290	537.37	.41	1	.52
	cFullHetACE	HetACE	8	2195.37	8290	537.37	.00	1	1.00

	HetACE	HomACE	5	23458.89	8293	6872.89	1508.52	3	.00
Social peer Perpetration	FullHetACE		9	23445.99	8287	6871.99			
	FullHetACE	HetACE	8	23445.99	8288	6869.99	.00	1	1.00
	cFullHetACE	HetACE	8	23445.99	8288	6869.99	-162.50	1	1.00
	HetACE	HomACE	5	23455.44	8291	6873.44	9.44	3	.02
Verbal peer Perpetration	FullHetACE		9	23192.44	8288	6616.44			
	FullHetACE	HetACE	8	23198.07	8289	662.08	5.63	1	.02
	cFullHetACE	HetACE	8	23198.07	8289	662.08	-49.68	1	1.00
	HetACE	HomACE	5	23403.96	8292	6819.96	205.89	3	.00
Cyber peer Perpetration	FullHetACE		9	23461.85	8288	6885.85			
	FullHetACE	HetACE	8	23461.85	8289	6883.85	.00	1	1.00
	cFullHetACE	HetACE	8	23461.85	8289	6883.85	-81.68	1	1.00
	HetACE	HomACE	5	23514.86	8292	693.86	53.01	3	.00
Parental role aspirations	FullHetACE		9	24069.01	8566	6937.01			
	FullHetACE	HetACE	8	24069.01	8567	6935.01	.00	1	1.00
	cFullHetACE	HetACE	8	24069.01	8567	6935.01	-11.25	1	1.00
	HetACE	HomACE	5	24085.72	8570	6945.72	16.71	3	.00
Occupational role aspirations	FullHetACE		9	24212.18	8571	707.18			
	FullHetACE	HetACE	8	24213.11	8572	7069.11	.93	1	.33
	cFullHetACE	HetACE	8	24213.11	8572	7069.11	-28.55	1	1.00
	HetACE	HomACE	5	24217.93	8575	7067.93	4.82	3	.19
Homecare role aspirations	FullHetACE		9	2397.03	8571	6828.03			
	FullHetACE	HetACE	8	23977.23	8572	6833.23	7.21	1	.01
	cFullHetACE	HetACE	8	23977.23	8572	6833.23	-31.77	1	1.00
	HetACE	HomACE	5	24005.48	8575	6855.48	28.24	3	.00
Importance of Relationships	FullHetACE		9	26815.63	9573	7669.63			
	FullHetACE	HetACE	8	26829.03	9574	7681.03	13.40	1	.00
	cFullHetACE	HetACE	8	26829.03	9574	7681.03	-29.01	1	1.00
	HetACE	HomACE	5	26885.05	9577	7731.05	56.02	3	.00
Achievement Motivation	FullHetACE		9	26899.51	9572	7755.51			

	FullHetACE	HetACE	8	26899.51	9573	7753.51	.00	1	1.00
	cFullHetACE	HetACE	8	26899.51	9573	7753.51	.00	1	1.00
	HetACE	HomACE	5	26913.25	9576	7761.25	13.73	3	.00
Purpose in Life	FullHetACE		9	26724.66	9565	7594.66			
	FullHetACE	HetACE	8	26724.66	9566	7592.66	.00	1	1.00
	cFullHetACE	HetACE	8	26724.66	9566	7592.66	.00	1	1.00
	HetACE	HomACE	5	26729.06	9569	7591.06	4.41	3	.22
Importance of democracy and equality	FullHetACE		9	26672.06	9546	758.06			
	FullHetACE	HetACE	8	26672.06	9547	7578.06	.00	1	1.00
	cFullHetACE	HetACE	8	26672.06	9547	7578.06	-3.50	1	1.00
	HetACE	HomACE	5	26708.37	9550	7608.37	36.31	3	.00
Environmental concerns	FullHetACE		9	26469.64	9557	7355.64			
	FullHetACE	HetACE	8	26471.93	9558	7355.94	2.30	1	.13
	cFullHetACE	HetACE	8	26471.93	9558	7355.94	-18.93	1	1.00
	HetACE	HomACE	5	26486.35	9561	7364.35	14.41	3	.00
Religiosity	FullHetACE		9	24571.78	9382	5807.78			
	FullHetACE	HetACE	8	24571.78	9383	5805.78	.00	1	1.00
	cFullHetACE	HetACE	8	24571.78	9383	5805.78	-182.81	1	1.00
	HetACE	HomACE	5	24576.91	9386	5804.91	5.13	3	.16
Importance of leisure	FullHetACE		9	23511.35	8393	6725.35			
	FullHetACE	HetACE	8	23512.04	8394	6724.04	.69	1	.41
	cFullHetACE	HetACE	8	23512.04	8394	6724.04	-12.45	1	1.00
	HetACE	HomACE	5	23516.45	8397	6722.45	4.41	3	.22
Alcohol use	FullHetACE		9	21449.92	7703	6043.92			
	FullHetACE	HetACE	8	2145.58	7704	6042.58	.66	1	.42
	cFullHetACE	HetACE	8	2145.58	7704	6042.58	.00	1	1.00
	HetACE	HomACE	5	21476.3	7707	6062.30	25.71	3	.00
Ever smoked	FullHetACE		9	23442.9	8425	6592.90			
	FullHetACE	HetACE	8	23443.07	8426	6591.07	.17	1	.68
	cFullHetACE	HetACE	8	23443.07	8426	6591.07	-51.83	1	1.00

	HetACE	HomACE	5	23443.71	8429	6585.71	.63	3	.89
Ever vaped	FullHetACE		9	2362.67	8421	6778.67			
	FullHetACE	HetACE	8	2362.67	8422	6776.67	.00	1	1.00
	cFullHetACE	HetACE	8	2362.67	8422	6776.67	-67.70	1	1.00
	HetACE	HomACE	5	2364.75	8425	679.75	2.07	3	.00
Cognitive enhancers	FullHetACE		9	23423.13	8339	6745.14			
	FullHetACE	HetACE	8	23427.72	8340	6747.72	4.58	1	.03
	cFullHetACE	HetACE	8	23427.72	8340	6747.72	-41.26	1	1.00
	HetACE	HomACE	5	23486.49	8343	680.49	58.77	3	.00
Big 5 Agreeableness	FullHetACE		9	26249.15	9278	7693.15			
	FullHetACE	HetACE	8	26256.32	9279	7698.32	7.16	1	.01
	cFullHetACE	HetACE	8	26256.32	9279	7698.32	-147.48	1	1.00
	HetACE	HomACE	5	26268.85	9282	7704.85	12.53	3	.01
Big 5 Conscientiousness	FullHetACE		9	26077.32	9265	7547.32			
	FullHetACE	HetACE	8	26086.36	9266	7554.36	9.04	1	.00
	cFullHetACE	HetACE	8	26086.36	9266	7554.36	-37.18	1	1.00
	HetACE	HomACE	5	26102.9	9269	7564.90	16.54	3	.00
Big 5 Extraversion	FullHetACE		9	2610.91	9315	747.91			
	FullHetACE	HetACE	8	26106.08	9316	7474.08	5.18	1	.02
	cFullHetACE	HetACE	8	26106.08	9316	7474.08	-37.37	1	1.00
	HetACE	HomACE	5	26114.12	9319	7476.12	8.04	3	.05
	FullHetACE		9	26206.41	9328	755.41			
	FullHetACE	HetACE	8	26206.41	9329	7548.41	.00	1	1.00
	cFullHetACE	HetACE	8	26206.41	9329	7548.41	-1.18	1	1.00
	HetACE	HomACE	5	26209.01	9332	7545.01	2.60	3	.46
Big 5 Openness	FullHetACE		9	26143.47	9299	7545.47			
	FullHetACE	HetACE	8	26149.17	9300	7549.17	5.70	1	.02
	cFullHetACE	HetACE	8	26149.17	9300	7549.17	-33.94	1	1.00
	HetACE	HomACE	5	26155.54	9303	7549.54	6.37	3	.10
Self-control	FullHetACE		9	25985.49	9257	7471.49			

	FullHetACE	HetACE	8	25988.84	9258	7472.84	3.35	1	.07
	cFullHetACE	HetACE	8	25988.84	9258	7472.84	-38.77	1	1.00
	HetACE	HomACE	5	25998.48	9261	7476.48	9.64	3	.02
Not planning for the future	FullHetACE		9	26119.56	9249	7621.56			
	FullHetACE	HetACE	8	26119.56	9250	7619.56	.00	1	1.00
	cFullHetACE	HetACE	8	26119.56	9250	7619.56	-109.58	1	1.00
	HetACE	HomACE	5	26123.01	9253	7617.01	3.45	3	.33
Ambition	FullHetACE		9	24073.56	8559	6955.57			
	FullHetACE	HetACE	8	24074.46	8560	6954.46	.89	1	.34
	cFullHetACE	HetACE	8	24074.46	8560	6954.46	-32.39	1	1.00
	HetACE	HomACE	5	24077.22	8563	6951.22	2.76	3	.43
Physical activity	FullHetACE		9	26213.51	9352	7509.51			
	FullHetACE	HetACE	8	26215.3	9353	7509.30	1.79	1	.18
	cFullHetACE	HetACE	8	26215.3	9353	7509.30	.00	1	1.00
	HetACE	HomACE	5	26218.64	9356	7506.64	3.34	3	.34
Health behaviours	FullHetACE		9	25915.07	9385	7145.07			
	FullHetACE	HetACE	8	25918.69	9386	7146.69	3.63	1	.06
	cFullHetACE	HetACE	8	25918.69	9386	7146.69	-17.04	1	1.00
	HetACE	HomACE	5	2592.43	9389	7142.44	1.74	3	.63
Risk taking behaviour	FullHetACE		9	25709.8	9246	7217.80			
	FullHetACE	HetACE	8	25709.96	9247	7215.96	.16	1	.69
	cFullHetACE	HetACE	8	25709.96	9247	7215.96	-59.27	1	1.00
	HetACE	HomACE	5	25734.29	9250	7234.29	24.33	3	.00
Risky sexual behaviour	FullHetACE		9	24759.59	9022	6715.59			
	FullHetACE	HetACE	8	2476.7	9023	6714.70	1.11	1	.29
	cFullHetACE	HetACE	8	2476.7	9023	6714.70	.00	1	1.00
	HetACE	HomACE	5	24763.31	9026	6711.32	2.62	3	.45
Media use	FullHetACE		9	25683.97	9301	7081.97			
	FullHetACE	HetACE	8	25689.58	9302	7085.58	5.60	1	.02
	cFullHetACE	HetACE	8	25689.58	9302	7085.58	-42.24	1	1.00

	HetACE	HomACE	5	25808.11	9305	7198.11	118.53	3	.00
Volunteering	FullHetACE		9	26221.24	9390	7441.24			
	FullHetACE	HetACE	8	26221.52	9391	7439.53	.29	1	.59
	cFullHetACE	HetACE	8	26221.52	9391	7439.53	.00	1	1.00
	HetACE	HomACE	5	26225.67	9394	7437.67	4.15	3	.25
Internet dating	FullHetACE		9	26071.32	9271	7529.32			
(scale)	FullHetACE	HetACE	8	26071.88	9272	7527.88	.56	1	.46
	cFullHetACE	HetACE	8	26071.88	9272	7527.88	-72.20	1	1.00
	HetACE	HomACE	5	26076.86	9275	7526.86	4.98	3	.17
Internet dating	FullHetACE		9	26195.6	9302	7591.60			
(y/n)	FullHetACE	HetACE	8	26197.26	9303	7591.26	1.65	1	.20
	cFullHetACE	HetACE	8	26197.26	9303	7591.26	-95.71	1	1.00
	HetACE	HomACE	5	26203.34	9306	7591.34	6.09	3	.11
Financial literacy: Knowledge of Products	FullHetACE		9	25642.94	9145	7352.94			
	FullHetACE	HetACE	8	25645.94	9146	7353.94	3.00	1	.08
	cFullHetACE	HetACE	8	25645.94	9146	7353.94	-17.42	1	1.00
	HetACE	HomACE	5	25677.14	9149	7379.14	31.20	3	.00
Financial attitudes and behaviour	FullHetACE		9	25819.16	9143	7533.16			
	FullHetACE	HetACE	8	25819.16	9144	7531.16	.00	1	1.00
	cFullHetACE	HetACE	8	25819.16	9144	7531.16	-138.15	1	1.00
	HetACE	HomACE	5	25828.67	9147	7534.67	9.50	3	.02
Functional outcome	Model fit statistics								
	base	comparison	ep	minus2LL	df	AIC	diffLL	diffdf	p
Wellbeing factor (1st PC)	FullHetACE		9	25518.12	9123	7272.12			
	FullHetACE	HetACE	8	25521.7	9124	7273.70	3.58	1	.06
	cFullHetACE	HetACE	8	25521.7	9124	7273.70	3.77	1	.05
	HetACE	HomACE	5	25529.45	9127	7275.45	7.75	3	.05
P factor (1st PC)	FullHetACE		9	19972.58	7169	5634.58			
	FullHetACE	HetACE	8	19972.72	7170	5632.72	.14	1	.71
	cFullHetACE	HetACE	8	19972.72	7170	5632.72	.00	1	1.00

	HetACE	HomACE	5	20006.12	7173	566.12	33.40	3	.00
Health factor (1st PC)	FullHetACE		9	21529.51	7715	6099.51			
	FullHetACE	HetACE	8	21534.05	7716	6102.05	4.54	1	.03
	cFullHetACE	HetACE	8	21534.05	7716	6102.05	.00	1	1.00
	HetACE	HomACE	5	2159.29	7719	6152.29	56.24	3	.00
Achieved educational level	FullHetACE		9	26005.97	9325	7355.97			
	FullHetACE	HetACE	8	26005.97	9326	7353.97	.00	1	1.00
	cFullHetACE	HetACE	8	26005.97	9326	7353.97	-49.90	1	1.00
	HetACE	HomACE	5	26012.72	9329	7354.72	6.75	3	.08
Planned educational level	FullHetACE		9	17251.68	6120	5011.68			
	FullHetACE	HetACE	8	17252.73	6121	501.73	1.04	1	.31
	cFullHetACE	HetACE	8	17252.73	6121	501.73	-9.05	1	1.00
	HetACE	HomACE	5	17254.65	6124	5006.65	1.93	3	.59

Note. HetACE= gender heterogeneity model not assuming equal variances across sexes and genetic correlation of unity; HomACE= gender homogeneity model assuming equal variances across sexes; ep= estimated parameters; FullHetACE= full gender heterogeneity model not assuming the genetic correlation between sexes to be 1; -2LL= minus 2 log-likelihood; df= degrees of freedom; AIC= Akaike's information criterion; diffLL= log-likelihood-ratio χ^2 test comparing the models to the base; diffdf= difference in degrees of freedom; p= the probability value of the base model fitting the data significantly worse than the comparison model, indicating significant sex differences.

Table S12. Model fitting results for bivariate analyses of the correlation between psychological traits and functional outcomes explained by genetic (A), shared environmental (C), and non-shared environmental (E) factors (95% confidence intervals are in parentheses).

	Model fitting results											
	Functional outcome: wellbeing											
	Whole sample			Males only			Females only			Same-sex MZ and DZ twins		
Trait	A	C	E	A	C	E	A	C	E	A	C	E
Daily hassles	-.15 (-.23,-.07)	-.04 (-.10 ,.02)	-.12 (-.15,-.08)	-.16 (-.24,-.05)	.00 (-.08,.04)	-.16 (-.21,-.11)	-.12 (-.22,-.03)	-.08 (-.15,.01)	-.09 (-.13,-.06)	-.09 (-.18,.00)	-.10 (-.17,-.02)	-.11 (-.15,-.08)
CHAOS	-.07 (-.14 ,.01)	-.10 (-.16,-.04)	-.15 (-.18,-.12)	-.06 (-.18,.07)	-.06 (-.15,.02)	-.17 (-.23,-.12)	-.07 (-.16,.03)	-.12 (-.20,-.05)	-.13 (-.17,-.10)	-.04 (-.13,.05)	-.13 (-.21,-.06)	-.15 (-.18,-.12)
Childhood experiences	-.14 (-.21,-.07)	-.03 (-.09 ,.03)	-.07 (-.10,-.05)	-.08 (-.19,.04)	-.03 (-.12,.05)	-.13 (-.18,-.09)	-.16 (-.24,-.07)	-.05 (-.12,.03)	-.04 (-.07,-.02)	-.13 (-.21,-.05)	-.04 (-.12,.03)	-.07 (-.09,-.04)
Poor sleep quality	-.13 (-.22,-.05)	-.02 (-.08 ,.05)	-.09 (-.12,-.06)	-.08 (-.17,.01)	-.01 (-.11,.05)	-.15 (-.21,-.10)	-.16 (-.27,-.05)	-.02 (-.11,.06)	-.06 (-.10,-.02)	-.08 (-.18,.02)	-.08 (-.17,.01)	-.09 (-.13,-.06)

Marriage hopes	.04 (-.04,.13)	.04 (-.02,.11)	.06 (.03,.09)	.05 (-.06,.18)	.06 (-.04,.13)	.05 (-.01,.10)	.05 (-.05,.15)	.03 (-.05,.11)	.06 (.03,.10)	.06 (-.03,.16)	.02 (-.06,.10)	.06 (.03,.09)
Marriage worries	-.11 (-.18,-.04)	-.05 (-.11,.00)	-.12 (-.15,-.10)	-.14 (-.20,-.02)	-.01 (-.09,.03)	-.10 (-.15,-.05)	-.09 (-.19,.01)	-.09 (-.16,-.01)	-.14 (-.18,-.11)	-.05 (-.14,.03)	-.11 (-.18,-.03)	-.13 (-.16,-.10)
Quality of relationship with twin	.09 (.03,.14)	.05 (.01,.10)	.03 (.02,.05)	.03 (-.07,.12)	.07 (.00,.15)	.05 (.02,.09)	.12 (.05,.20)	.04 (-.02,.10)	.02 (.00,.05)	.06 (.00,.13)	.10 (.04,.16)	.04 (.02,.06)
Quality of relationship with mother	.14 (.07,.21)	.01 (-.04,.07)	.06 (.04,.09)	.16 (.08,.24)	.00 (-.05,.05)	.04 (-.01,.08)	.11 (.02,.21)	.04 (-.04,.12)	.08 (.04,.11)	.14 (.05,.23)	.01 (-.06,.09)	.06 (.04,.09)
Quality of relationship with father	.14 (.07,.21)	.03 (-.03,.09)	.07 (.04,.09)	.14 (.02,.26)	.03 (-.06,.12)	.10 (.05,.14)	.13 (.04,.21)	.04 (-.03,.11)	.05 (.03,.08)	.11 (.03,.19)	.06 (-.02,.13)	.07 (.04,.09)
Number of relationships	.05 (-.01,.10)	-.01 (-.05,.03)	.03 (.00,.05)	.03 (-.05,.09)	.00 (-.04,.05)	.06 (.02,.10)	.07 (-.01,.15)	-.02 (-.08,.04)	.01 (-.03,.04)	.04 (-.05,.13)	.00 (-.08,.07)	.02 (-.01,.05)
Longest relationship	.11 (.05,.17)	-.02 (-.07,.02)	.09 (.06,.12)	.05 (-.04,.14)	-.01 (-.07,.05)	.12 (.06,.18)	.15 (.06,.23)	-.03 (-.09,.03)	.08 (.04,.12)	.11 (.02,.19)	-.02 (-.09,.06)	.07 (.04,.11)
Partner violence	-.10 (-.18,-.02)	-.01 (-.07,.04)	-.10 (-.13,-.07)	-.08 (-.17,.05)	.00 (-.09,.06)	-.12 (-.18,-.07)	-.11 (-.20,.00)	-.02 (-.10,.05)	-.09 (-.13,-.05)	-.12 (-.19,.00)	.00 (-.10,.06)	-.10 (-.14,-.07)
Contact with mother	.12 (.00,.23)	-.02 (-.11,.07)	.02 (-.02,.06)	.14 (-.05,.29)	-.03 (-.13,.10)	.04 (-.04,.13)	.09 (-.05,.23)	-.01 (-.12,.11)	.01 (-.04,.06)	.09 (-.05,.23)	.01 (-.11,.12)	.02 (-.03,.06)
Communication with mother	.14 (.03,.25)	-.01 (-.10,.08)	-.01 (-.05,.02)	.16 (-.03,.33)	-.03 (-.14,.12)	-.02 (-.10,.06)	.13 (-.01,.27)	.00 (-.12,.11)	-.01 (-.06,.03)	.14 (.00,.27)	.00 (-.12,.12)	-.01 (-.05,.03)
Contact with father	.07 (.00,.18)	.03 (-.06,.11)	.00 (-.04,.04)	-.02 (-.17,.18)	.08 (-.06,.19)	.04 (-.04,.11)	.12 (-.01,.25)	-.01 (-.11,.10)	-.02 (-.07,.03)	.08 (-.05,.20)	.03 (-.08,.14)	.00 (-.04,.05)
Communication with father	.06 (-.03,.14)	.05 (-.02,.13)	.02 (-.01,.05)	.03 (-.13,.20)	.05 (-.08,.17)	.05 (-.01,.12)	.05 (-.03,.15)	.07 (-.01,.16)	.00 (-.03,.04)	.03 (-.08,.13)	.09 (.00,.18)	.02 (-.01,.05)
Peer pressure	-.14 (-.19,-.07)	.03 (-.02,.07)	-.10 (-.13,-.07)	-.12 (-.21,-.03)	.02 (-.04,.08)	-.10 (-.15,-.05)	-.14 (-.21,-.04)	.02 (-.05,.08)	-.10 (-.14,-.06)	-.13 (-.21,-.04)	.02 (-.06,.08)	-.10 (-.13,-.07)
Physical peer victimisation	.02 (-.05,.09)	-.02 (-.07,.02)	-.09 (-.13,-.06)	.04 (-.04,.16)	-.02 (-.10,.03)	-.11 (-.17,-.05)	-.01 (-.11,.09)	-.02 (-.09,.05)	-.08 (-.13,-.03)	.05 (-.05,.14)	-.05 (-.12,.03)	-.10 (-.14,-.06)
Social peer victimisation	-.05 (-.13,.03)	-.03 (-.09,.03)	-.11 (-.15,-.08)	.00 (-.15,.12)	-.05 (-.13,.06)	-.15 (-.21,-.08)	-.06 (-.15,.03)	-.03 (-.10,.03)	-.10 (-.14,-.06)	.05 (-.05,.13)	-.12 (-.18,-.04)	-.11 (-.14,-.07)
Verbal peer victimisation	-.10 (-.16,-.02)	.02 (-.04,.06)	-.11 (-.14,-.08)	-.04 (-.15,.11)	.01 (-.09,.08)	-.14 (-.20,-.08)	-.12 (-.20,-.03)	.01 (-.06,.07)	-.10 (-.14,-.06)	-.08 (-.18,.02)	.00 (-.09,.07)	-.11 (-.14,-.07)
Cyber peer victimisation	-.09 (-.15,-.02)	.00 (-.05,.04)	-.09 (-.12,-.06)	-.01 (-.16,.13)	-.03 (-.13,.07)	-.13 (-.19,-.06)	-.10 (-.17,-.02)	.00 (-.06,.05)	-.07 (-.11,-.03)	-.05 (-.13,.03)	-.03 (-.09,.04)	-.08 (-.12,-.05)
Physical peer perpetration	.03 (-.04,.10)	-.02 (-.07,.03)	-.06 (-.10,-.03)	.05 (-.01,.15)	-.02 (-.09,.03)	-.09 (-.15,-.03)	-.01 (-.11,.09)	-.01 (-.07,.05)	-.03 (-.09,.03)	.05 (-.07,.15)	-.03 (-.12,.07)	-.07 (-.11,-.03)
Social peer perpetration	-.06 (-.14,.01)	-.01 (-.06,.04)	-.09 (-.13,-.06)	-.05 (-.15,.04)	.00 (-.05,.06)	-.14 (-.20,-.08)	-.03 (-.13,.08)	-.05 (-.13,.03)	-.08 (-.13,-.04)	-.02 (-.13,.08)	-.05 (-.14,.03)	-.09 (-.13,-.06)
Verbal peer perpetration	-.04 (-.10,.03)	-.01 (-.05,.04)	-.07 (-.10,-.04)	.00 (-.09,.09)	.00 (-.05,.07)	-.12 (-.17,-.07)	-.05 (-.14,.04)	-.03 (-.09,.03)	-.04 (-.08,.00)	-.01 (-.10,.09)	-.04 (-.11,.04)	-.07 (-.10,-.03)
Cyber peer perpetration	-.04 (-.11,.02)	.00 (-.05,.04)	-.07 (-.11,-.03)	-.02 (-.09,.09)	.00 (-.06,.04)	-.11 (-.16,-.05)	-.07 (-.16,.03)	.00 (-.07,.06)	-.05 (-.09,.00)	.00 (-.10,.09)	-.05 (-.12,.03)	-.07 (-.10,-.03)
Parental role aspirations	.13 (.05,.19)	-.01 (-.05,.05)	.04 (.01,.08)	.10 (.01,.18)	.00 (-.05,.05)	.07 (.02,.13)	.13 (.02,.23)	.00 (-.08,.08)	.03 (-.01,.07)	.12 (.03,.20)	.00 (-.06,.07)	.04 (.01,.08)

Occupational role aspirations	.02 (-.05 ,.09)	-.01 (-.06 ,.04)	.00 (-.03 ,.03)	.04 (- .06,.15)	-.02 (- .10,.04)	.01 (- .05,.07)	-.01 (- .10,.09)	.01 (- .06,.08)	.00 (- .04,.04)	.05 (- .05,.14)	-.03 (- .10,.05)	.01 (- .03,.04)
Homecare role aspirations	.11 (.04 ,.17)	.01 (-.03 ,.06)	.07 (.04 ,.10)	.13 (.05,.22)	.00 (- .06,.05)	.07 (.03,.12)	.07 (- .02,.16)	.04 (- .03,.11)	.07 (.03,.10)	.06 (- .03,.16)	.06 (- .02,.14)	.07 (.04,.10)
Importance of relationships	.09 (.04 ,.14)	.02 (-.01 ,.06)	.06 (.03 ,.09)	.06 (- .04,.13)	.02 (- .01,.10)	.07 (.02,.12)	.13 (.05,.19)	.00 (- .04,.06)	.06 (.02,.09)	.08 (.01,.15)	.02 (- .03,.08)	.07 (.04,.10)
Achievement motivation	.09 (.02 ,.16)	-.01 (-.05 ,.04)	.02 (-.01 ,.05)	.09 (- .03,.19)	.00 (- .07,.08)	.03 (- .02,.08)	.09 (.01,.17)	-.01 (- .07,.05)	.02 (- .02,.05)	.08 (- .02,.17)	.00 (- .07,.08)	.02 (- .01,.05)
Purpose in life	.24 (.16 ,.31)	.02 (-.03 ,.08)	.19 (.16 ,.22)	.19 (.06,.29)	.02 (- .03,.12)	.22 (.17,.28)	.25 (.15,.33)	.02 (- .04,.10)	.17 (.14,.21)	.22 (.13,.32)	.03 (- .05,.11)	.19 (.16,.22)
Importance of democracy and equality	-.01 (-.07 ,.05)	.00 (-.04 ,.04)	.00 (-.03 ,.03)	-.02 (- .12,.08)	.02 (- .05,.09)	.00 (- .05,.05)	-.01 (- .08,.07)	-.02 (- .07,.04)	.00 (- .03,.04)	.02 (- .05,.10)	-.03 (- .09,.03)	.00 (- .03,.03)
Environmental concerns	.00 (-.08 ,.06)	-.02 (-.07 ,.04)	.01 (-.01 ,.04)	-.03 (- .12,.06)	.02 (- .04,.08)	.02 (- .03,.06)	.04 (- .05,.12)	-.07 (- .12,.01)	.01 (- .02,.04)	.02 (- .07,.10)	-.05 (- .12,.02)	.01 (- .02,.04)
Religiosity	.00 (-.06 ,.06)	.01 (-.04 ,.06)	.01 (-.02 ,.03)	.09 (- .01,.19)	-.05 (- .13,.03)	-.01 (- .05,.02)	-.05 (- .13,.02)	.04 (- .02,.11)	.02 (- .01,.04)	-.01 (- .08,.06)	.02 (- .05,.08)	.01 (- .02,.03)
Importance of leisure	.16 (.09 ,.23)	.05 (-.01 ,.10)	.11 (.08 ,.14)	.11 (- .02,.22)	.05 (- .02,.14)	.14 (.09,.20)	.19 (.10,.28)	.04 (- .03,.11)	.09 (.05,.13)	.13 (.04,.22)	.08 (.00,.16)	.11 (.08,.15)
Alcohol use	-.08 (-.14,- .01)	.05 (.00 ,.09)	-.08 (-.11,- .05)	-.09 (- .19,.03)	.09 (.01,.16)	-.13 (-.18,- .07)	-.04 (- .13,.06)	-.01 (- .08,.07)	-.06 (-.10,- .03)	-.09 (- .18,.01)	.06 (- .02,.14)	-.08 (-.11,- .05)
Ever smoked	-.07 (-.13 ,.00)	.03 (-.02 ,.07)	-.04 (-.07,- .01)	-.03 (- .11,.06)	.00 (- .05,.06)	-.06 (-.10,- .01)	-.10 (-.18,- .01)	.05 (- .03,.11)	-.03 (- .07,.00)	-.07 (- .15,.02)	.02 (- .05,.09)	-.04 (-.07,- .01)
Ever vaped	-.06 (-.14 ,.03)	.00 (-.06 ,.07)	-.04 (-.07,- .01)	-.01 (- .11,.11)	-.02 (- .10,.05)	-.06 (- .11,.00)	-.10 (- .20,.01)	.02 (- .06,.11)	-.03 (- .07,.01)	-.09 (- .19,.01)	.04 (- .04,.13)	-.04 (- .07,.00)
Cognitive enhancers	-.09 (-.17 ,.00)	.00 (-.07 ,.05)	-.06 (-.09,- .03)	.01 (- .09,.14)	-.02 (- .11,.04)	-.11 (-.17,- .05)	-.17 (-.27,- .06)	.02 (- .06,.10)	-.03 (- .07,.01)	-.09 (- .19,.01)	-.01 (- .09,.08)	-.06 (-.09,- .03)
Big 5 Agreeableness	.05 (-.01 ,.10)	.00 (-.03 ,.04)	.05 (.02 ,.08)	.01 (- .08,.09)	.00 (- .05,.06)	.07 (.02,.12)	.06 (- .01,.13)	.01 (- .04,.05)	.04 (.00,.08)	.06 (- .01,.13)	-.01 (- .06,.04)	.05 (.02,.09)
Big 5 Conscientiousness	.16 (.09 ,.20)	.00 (-.03 ,.04)	.11 (.08 ,.14)	.13 (.05,.20)	.00 (- .04,.05)	.14 (.09,.19)	.16 (.08,.23)	.02 (- .03,.07)	.10 (.06,.14)	.16 (.09,.23)	.00 (- .05,.06)	.12 (.08,.15)
Big 5 Extraversion	.17 (.11 ,.22)	.02 (-.01 ,.06)	.09 (.06 ,.12)	.12 (.01,.20)	.02 (- .02,.10)	.12 (.07,.17)	.20 (.13,.27)	.01 (- .03,.07)	.07 (.04,.11)	.14 (.07,.21)	.05 (- .01,.11)	.09 (.06,.12)
Big 5 Neuroticism	-.17 (-.24,- .09)	-.05 (-.11 ,.01)	-.16 (-.19,- .13)	-.11 (-.23,- .11)	-.04 (- .14,.03)	-.21 (-.27,- .16)	-.18 (-.27,- .09)	-.06 (- .13,.00)	-.13 (-.17,- .10)	-.14 (-.24,- .05)	-.07 (- .15,.01)	-.16 (-.19,- .13)
Big 5 Openness	-.11 (-.16,- .06)	.00 (-.04 ,.03)	-.03 (-.06 ,.00)	-.09 (-.18,- .02)	.00 (- .05,.05)	-.06 (-.11,- .01)	-.11 (-.18,- .03)	-.01 (- .06,.04)	-.01 (- .04,.03)	-.11 (-.18,- .04)	.01 (- .05,.06)	-.02 (- .05,.00)
Self-control	.14 (.08 ,.20)	.02 (-.02 ,.06)	.14 (.11 ,.17)	.13 (.02,.19)	.00 (- .03,.08)	.16 (.12,.22)	.15 (.07,.23)	.03 (- .02,.09)	.12 (.09,.16)	.12 (.04,.20)	.05 (- .01,.11)	.14 (.11,.17)
Not planning for the future	-.08 (-.15 ,.00)	-.01 (-.07 ,.04)	-.09 (-.12,- .05)	-.09 (- .17,.00)	.00 (- .06,.05)	-.09 (-.14,- .04)	-.05 (-.16,- .01)	-.04 (- .12,.05)	-.08 (-.13,- .04)	-.09 (- .18,.00)	.00 (- .07,.07)	-.08 (-.12,- .05)
Ambition	.15 (.08 ,.21)	-.03 (-.08 ,.01)	.02 (-.01 ,.05)	.16 (.06,.25)	-.04 (- .10,.02)	.04 (- .02,.09)	.13 (.03,.21)	-.02 (- .08,.06)	.01 (- .02,.05)	.17 (.08,.24)	-.04 (- .10,.03)	.02 (- .01,.05)
Physical activity	.10 (.03 ,.16)	.00 (-.05 ,.04)	.03 (.00 ,.06)	.10 (.01,.19)	.00 (- .06,.05)	.06 (.01,.11)	.08 (.00,.17)	.00 (- .06,.07)	.02 (- .02,.05)	.09 (.00,.18)	.00 (- .07,.08)	.03 (.00,.06)

Health behaviours	.04 (-.02 ,.12)	.03 (-.02 ,.09)	.04 (.01 ,.06)	.10 (.02,.19)	.00 (-.06,.06)	.00 (-.04,.05)	.01 (-.08,.10)	.06 (.01,.13)	.06 (.03,.09)	.02 (-.07,.09)	.05 (-.01,.13)	.04 (.01,.06)
Risk taking behaviour	-.07 (-.13,-.01)	.03 (-.02 ,.07)	-.08 (-.11,-.06)	-.10 (-.19,-.01)	.07 (.00,.13)	-.08 (-.13,-.04)	-.04 (-.12,.04)	-.01 (-.07,.05)	-.09 (-.12,-.05)	-.06 (-.14,.02)	.02 (-.05,.08)	-.08 (-.11,-.05)
Risky sexual behaviour	.05 (-.02 ,.11)	-.01 (-.06 ,.04)	.02 (.00 ,.05)	.02 (-.06,.11)	-.01 (-.07,.05)	.06 (.02,.10)	.04 (-.05,.13)	.01 (-.06,.08)	.01 (-.02,.04)	-.01 (-.10,.08)	.05 (-.02,.12)	.02 (.00,.05)
Media use	.04 (-.01 ,.09)	.01 (-.02 ,.04)	-.05 (-.07,-.02)	.07 (-.02,.13)	.01 (-.02,.07)	-.06 (-.11,-.02)	.04 (-.03,.12)	-.01 (-.07,.04)	-.05 (-.08,-.01)	.04 (-.02,.11)	.01 (-.04,.06)	-.04 (-.07,-.01)
Volunteering	.02 (-.04 ,.09)	.02 (-.03 ,.06)	.02 (-.01 ,.04)	.04 (-.05,.13)	.00 (-.06,.06)	.01 (-.03,.06)	.00 (-.08,.09)	.03 (-.04,.10)	.02 (-.01,.05)	.03 (-.06,.12)	.00 (-.08,.07)	.02 (-.01,.04)
Internet dating	-.09 (-.15,-.02)	.05 (.00 ,.08)	-.13 (-.16,-.10)	-.06 (-.15,.04)	.05 (-.02,.10)	-.14 (-.20,-.10)	-.09 (-.17,.00)	.03 (-.04,.09)	-.12 (-.16,-.09)	-.05 (-.14,.05)	.01 (-.07,.08)	-.12 (-.15,-.09)
Internet dating	-.07 (-.13,-.01)	.04 (-.01 ,.08)	-.11 (-.14,-.08)	-.03 (-.12,.08)	.03 (-.04,.09)	-.13 (-.18,-.08)	-.09 (-.16,.00)	.03 (-.04,.09)	-.10 (-.14,-.07)	-.03 (-.12,.07)	.00 (-.08,.08)	-.10 (-.13,-.07)
Financial literacy: knowledge of products	.11 (.04 ,.17)	.01 (-.03 ,.06)	.10 (.07 ,.13)	.13 (.03,.21)	.00 (-.05,.08)	.08 (.04,.13)	.10 (.01,.18)	.02 (-.04,.08)	.12 (.08,.15)	.09 (-.01,.18)	.04 (-.04,.12)	.10 (.07,.13)
Financial attitudes and behaviour	.15 (.08 ,.20)	.00 (-.04 ,.04)	.18 (.15 ,.21)	.15 (.05,.22)	.00 (-.05,.06)	.20 (.14,.25)	.14 (.05,.21)	.01 (-.05,.07)	.18 (.14,.22)	.14 (.05,.21)	.01 (-.05,.08)	.17 (.14,.21)

	Model fitting results											
	Functional outcome: adverse mental health											
	Whole sample			Males only			Females only			Same-sex MZ and DZ twins		
Trait	A	C	E	A	C	E	A	C	E	A	C	E
Daily hassles	.27 (.20 ,.31)	.00 (-.01 ,.06)	.26 (.23 ,.29)	.20 (.04, .30)	.02 (-.04, .13)	.31 (.25, .38)	.29 (.19,.34)	.01 (-.02,.09)	.24 (.20,.28)	.28 (.18,.32)	.00 (-.03,.08)	.25 (.22,.29)
CHAOS	.10 (.02 ,.18)	.10 (.04 ,.16)	.13 (.10 ,.16)	.20 (.05, .34)	-.01 (-.10, .10)	.12 (.06, .18)	.06 (-.03,.15)	.15 (.08,.22)	.12 (.09,.16)	.08 (.02,.18)	.14 (.06,.21)	.13 (.10,.16)
Childhood experiences	.22 (.15 ,.29)	.02 (-.03 ,.08)	.13 (.11 ,.16)	.10 (-.05, .24)	.04 (-.06, .15)	.21 (.16, .27)	.24 (.16,.32)	.04 (-.02,.12)	.10 (.07,.13)	.24 (.15,.33)	.02 (-.06,.10)	.13 (.10,.15)
Poor sleep quality	.27 (.19 ,.32)	.02 (-.02 ,.08)	.23 (.19 ,.26)	.16 (.02, .30)	.05 (-.04, .17)	.27 (.21, .34)	.31 (.21,.36)	.02 (-.02,.09)	.21 (.17,.25)	.23 (.12,.32)	.08 (-.01,.17)	.23 (.19,.26)
Marriage hopes	-.11 (-.18,-.03)	.00 (-.05 ,.06)	.01 (-.02 ,.05)	-.10 (-.23, .06)	-.01 (-.12, .08)	.02 (-.05, .08)	-.10 (-.18,-.02)	.00 (-.06,.06)	.01 (-.03,.04)	-.08 (-.18,.02)	-.02 (-.10,.06)	.02 (-.01,.05)
Marriage worries	.12 (.05 ,.19)	.03 (-.01 ,.09)	.07 (.04 ,.10)	.10 (-.03, .20)	.03 (-.03, .12)	.07 (.01, .13)	.12 (.03,.20)	.05 (-.01,.12)	.08 (.05,.12)	.08 (-.01,.16)	.07 (.00,.14)	.08 (.05,.11)
Quality of relationship with twin	-.11 (-.17,-.05)	-.06 (-.11,-.01)	-.02 (-.04 ,.00)	-.17 (-.28,-.06)	.03 (-.06, .12)	.02 (-.02, .07)	-.09 (-.17,-.02)	-.10 (-.17,-.04)	-.04 (-.07,-.01)	-.11 (-.19,-.04)	-.09 (-.16,-.03)	-.02 (-.05,.00)
Quality of relationship with mother	-.16 (-.21,-.10)	.00 (-.04 ,.04)	-.07 (-.10,-.04)	-.17 (-.28,-.07)	.03 (-.04, .10)	-.04 (-.09, .02)	-.16 (-.23,-.07)	-.01 (-.08,.04)	-.08 (-.11,-.05)	-.17 (-.25,-.07)	.00 (-.08,.06)	-.07 (-.10,-.04)
Quality of relationship with father	-.18 (-.25,-.11)	-.01 (-.07 ,.05)	-.06 (-.08,-.03)	-.10 (-.24, .04)	-.06 (-.16, .05)	-.09 (-.14,-.04)	-.21 (-.29,-.13)	.00 (-.06,.07)	-.04 (-.07,-.01)	-.20 (-.28,-.10)	.00 (-.08,.08)	-.05 (-.08,-.03)
Number of relationships	.08 (.05 ,.12)	.00 (-.02 ,.03)	-.01 (-.03 ,.02)	.07 (-.02, .17)	-.01 (-.08, .05)	-.02 (-.07, .03)	.10 (.03,.14)	.00 (-.02,.05)	.00 (-.03,.04)	.08 (.00,.16)	.01 (-.06,.07)	.00 (-.03,.03)

Longest relationship	-.03 (-.06 ,.01)	.00 (-.02 ,.03)	-.01 (-.04 ,.02)	.00 (-.12, .12)	.00 (-.08, .07)	-.05 (-.11, .02)	-.04 (- .09,.01)	.00 (- .03,.03)	.01 (- .03,.04)	-.03 (- .09,.04)	.00 (- .06,.05)	.00 (- .03,.04)
Partner violence	.21 (.13 ,.26)	.01 (-.01 ,.07)	.10 (.07 ,.13)	.11 (-.04, .23)	.04 (-.04, .14)	.14 (.08, .21)	.25 (.16,.29)	.00 (- .02,.08)	.09 (.05,.13)	.21 (.11,.26)	.01 (- .02,.09)	.10 (.07,.13)
Contact with mother	.05 (-.05 ,.15)	-.02 (-.09 ,.06)	-.03 (-.08 ,.01)	-.03 (-.20, .20)	.10 (-.07, .21)	-.07 (-.17, .02)	.07 (- .04,.18)	-.04 (- .12,.04)	-.01 (- .06,.04)	.10 (- .04,.21)	-.07 (- .16,.04)	-.03 (- .08,.01)
Communication with mother	-.05 (-.16 ,.05)	-.02 (-.10 ,.06)	-.03 (-.07 ,.01)	-.13 (-.31,- .13)	.09 (-.09, .21)	-.05 (-.15, .04)	-.03 (- .16,.08)	-.06 (- .15,.04)	-.01 (- .06,.03)	-.01 (- .15,.11)	-.06 (- .16,.06)	-.03 (- .07,.02)
Contact with father	.01 (-.10 ,.12)	-.02 (-.11 ,.06)	-.03 (-.07 ,.02)	-.15 (-.27, .06)	.12 (-.04, .21)	-.01 (-.10, .07)	.05 (.01,.17)	-.07 (- .16,.03)	-.03 (- .08,.02)	.04 (- .10,.16)	-.07 (- .17,.05)	-.03 (- .08,.01)
Communication with father	-.01 (-.01 ,.06)	-.10 (-.17,- .03)	-.03 (-.06 ,.00)	-.10 (-.29,- .01)	-.01 (-.15, .14)	-.02 (-.10, .05)	.02 (- .08,.12)	-.14 (-.22,- .05)	-.03 (- .07,.01)	-.04 (- .14,.07)	-.08 (- .18,.01)	-.03 (- .07,.00)
Peer pressure	.14 (.10 ,.19)	.00 (-.03 ,.03)	.12 (.10 ,.15)	.16 (.03, .26)	-.02 (-.09, .07)	.11 (.05, .17)	.15 (.11,.20)	.00 (- .03,.04)	.13 (.09,.17)	.16 (.07,.21)	.00 (- .03,.08)	.12 (.09,.15)
Physical peer victimisation	.10 (.05 ,.13)	.00 (-.02 ,.03)	.07 (.04 ,.11)	.01 (-.12, .11)	.03 (-.04, .12)	.13 (.07, .19)	.16 (.11,.22)	.00 (- .03,.03)	.03 (- .02,.07)	.10 (.03,.16)	.00 (- .05,.06)	.08 (.04,.12)
Social peer victimisation	.22 (.14 ,.26)	.00 (-.02 ,.06)	.18 (.15 ,.22)	.00 (-.09, .17)	.12 (.01, .19)	.25 (.18, .31)	.25 (.20,.29)	.00 (- .03,.04)	.17 (.13,.21)	.20 (.10,.29)	.02 (- .05,.10)	.18 (.15,.22)
Verbal peer victimisation	.21 (.16 ,.25)	.00 (-.02 ,.04)	.17 (.14 ,.20)	.01 (-.11, .15)	.11 (.01, .21)	.22 (.16, .29)	.26 (.21,.32)	.00 (- .04,.03)	.16 (.12,.20)	.22 (.11,.28)	.00 (- .05,.09)	.17 (.14,.20)
Cyber peer victimisation	.22 (.18 ,.25)	.00 (-.02 ,.04)	.14 (.11 ,.17)	-.01 (-.11, .14)	.14 (.04, .22)	.19 (.12, .25)	.24 (.20,.28)	.00 (- .02,.02)	.14 (.10,.18)	.21 (.14,.26)	.00 (- .04,.06)	.14 (.11,.17)
Physical peer perpetration	.07 (.02 ,.10)	.00 (-.02 ,.03)	.05 (.02 ,.09)	.02 (-.11, .14)	.02 (-.07, .11)	.08 (.02, .14)	.11 (.06,.17)	.00 (- .03,.03)	.02 (- .04,.07)	.11 (.02,.18)	-.05 (- .10,.03)	.06 (.02,.09)
Social peer perpetration	.16 (.10 ,.20)	.00 (-.02 ,.04)	.15 (.12 ,.19)	.12 (-.04, .23)	.03 (-.03, .14)	.17 (.10, .25)	.16 (.09,.23)	.00 (- .04,.05)	.15 (.10,.19)	.18 (.07,.24)	-.02 (- .06,.07)	.15 (.11,.18)
Verbal peer perpetration	.10 (.07 ,.14)	.00 (-.02 ,.03)	.12 (.09 ,.15)	.04 (-.08, .14)	.02 (-.05, .11)	.14 (.08, .20)	.13 (.09,.17)	.00 (- .03,.03)	.12 (.08,.16)	.10 (.03,.17)	.00 (- .05,.06)	.12 (.09,.15)
Cyber peer perpetration	.09 (.05 ,.13)	.00 (-.01 ,.03)	.13 (.09 ,.16)	.04 (-.10, .15)	.03 (-.03, .13)	.13 (.06, .20)	.10 (.05,.15)	.00 (- .03,.03)	.13 (.09,.17)	.09 (.00,.14)	.00 (- .03,.08)	.12 (.09,.16)
Parental role aspirations	-.12 (-.15,- .07)	.00 (-.03 ,.03)	-.03 (-.06 ,.00)	-.11 (-.21, .01)	-.01 (-.08, .06)	-.03 (-.09, .03)	-.13 (-.20,- .05)	.00 (- .06,.05)	-.03 (- .06,.01)	-.12 (-.17,- .04)	.00 (- .07,.03)	-.02 (- .05,.01)
Occupational role aspirations	-.06 (-.13,- .01)	.02 (-.02 ,.07)	.00 (-.03 ,.03)	-.13 (-.22,- .01)	.05 (-.04, .11)	.02 (-.04, .08)	-.04 (- .12,.02)	.01 (- .03,.07)	-.01 (- .04,.03)	-.10 (-.18,- .02)	.04 (- .02,.10)	.00 (- .03,.04)
Homecare role aspirations	-.07 (-.10,- .02)	.00 (-.03 ,.02)	.01 (-.02 ,.03)	-.07 (-.17, .04)	-.01 (-.09, .05)	.01 (-.04, .07)	-.07 (-.11,- .03)	.00 (- .04,.04)	.00 (- .03,.03)	-.04 (- .13,.04)	-.03 (- .10,.04)	.00 (- .03,.03)
Importance of relationships	-.09 (-.13,- .04)	.00 (-.03 ,.01)	-.03 (-.06,- .01)	-.04 (-.13, .07)	-.04 (-.11, .02)	.00 (-.05, .06)	-.10 (-.14,- .06)	.00 (- .03,.01)	-.06 (-.10,- .03)	-.08 (- .13,.00)	-.02 (- .08,.01)	-.04 (-.07,- .01)
Achievement motivation	-.06 (-.11,- .01)	.00 (-.03 ,.04)	.00 (-.03 ,.02)	.02 (-.12, .14)	-.07 (-.15, .04)	-.01 (-.06, .05)	-.06 (-.14,- .02)	.00 (- .02,.06)	-.01 (- .04,.02)	-.07 (- .14,.02)	.00 (- .07,.06)	.00 (- .03,.03)
Purpose in life	-.33 (-.37,- .26)	.01 (-.04 ,.03)	-.26 (-.29,- .23)	-.35 (-.40,- .19)	.05 (-.07, .07)	-.24 (-.31,- .19)	-.34 (-.38,- .25)	.00 (- .07,.02)	-.26 (-.30,- .23)	-.32 (-.39,- .21)	-.01 (- .10,.04)	-.25 (-.28,- .22)
Importance of democracy and equality	.03 (-.02 ,.06)	.00 (-.02 ,.03)	.01 (-.02 ,.03)	.10 (-.04, .22)	-.05 (-.12, .05)	.00 (-.05, .06)	.02 (- .04,.06)	.00 (- .02,.04)	.00 (- .04,.03)	.02 (- .05,.07)	.00 (- .03,.06)	.00 (- .02,.03)

Environmental concerns	.00 (-.05 ,.05)	.00 (-.04 ,.04)	-.03 (-.06 ,.00)	-.02 (-.13, .10)	-.02 (-.09, .06)	.00 (-.05, .05)	.00 (- .07,.07)	.01 (- .04,.06)	-.04 (-.08,- .01)	.01 (- .07,.10)	-.01 (- .07,.06)	-.03 (- .05,.00)
Religiosity	.03 (-.04 ,.09)	-.05 (-.10 ,.00)	.05 (.02 ,.07)	-.11 (-.23, .01)	.02 (-.07, .11)	.11 (.06, .15)	.08 (.00,.15)	-.07 (-.13,- .01)	.02 (.00,.05)	.01 (- .07,.09)	-.03 (- .10,.04)	.04 (.02,.07)
Importance of leisure	-.26 (-.29,- .18)	.00 (-.07 ,.01)	-.15 (-.18,- .12)	-.18 (-.31,- .03)	-.04 (-.15, .04)	-.18 (-.25,- .12)	-.28 (-.31,- .19)	.00 (- .07,.02)	-.14 (-.17,- .10)	-.22 (-.30,- .12)	-.04 (- .12,.02)	-.15 (-.18,- .12)
Alcohol use	.08 (.01 ,.15)	-.02 (-.06 ,.03)	.08 (.05 ,.11)	.15 (-.02, .25)	-.11 (-.18, .01)	.10 (.04, .16)	.09 (.02,.13)	.00 (- .03,.05)	.07 (.03,.10)	.07 (- .04,.15)	-.01 (- .08,.08)	.07 (.04,.10)
Ever smoked	.06 (.01 ,.10)	.00 (-.02 ,.04)	.05 (.03 ,.08)	.00 (-.12, .10)	.03 (-.04, .11)	.08 (.03, .14)	.08 (.01,.14)	.00 (- .04,.05)	.04 (.01,.07)	.06 (- .01,.12)	.00 (- .06,.05)	.05 (.02,.08)
Ever vaped	.14 (.08 ,.21)	-.01 (-.05 ,.04)	.04 (.01 ,.07)	.06 (-.08, .19)	.04 (-.06, .13)	.08 (.02, .14)	.18 (.09,.25)	-.02 (- .08,.04)	.02 (- .02,.05)	.16 (.07,.24)	-.04 (- .10,.04)	.03 (.00,.06)
Cognitive enhancers	.16 (.10 ,.22)	.00 (-.04 ,.05)	.06 (.04 ,.10)	.08 (-.07, .20)	.00 (-.08, .10)	.09 (.03, .15)	.20 (.12,.27)	.01 (- .04,.07)	.06 (.02,.09)	.20 (.09,.28)	-.02 (- .08,.07)	.07 (.04,.10)
Big 5 Agreeableness	-.06 (-.09,- .02)	.00 (-.02 ,.02)	-.05 (-.08,- .02)	-.08 (-.20, .03)	.05 (-.02, .13)	-.03 (-.09, .03)	-.08 (-.12,- .03)	.00 (- .03,.01)	-.06 (-.10,- .02)	-.07 (-.12,- .02)	.00 (- .03,.04)	-.06 (-.09,- .03)
Big 5 Conscientiousness	-.18 (-.21,- .14)	.00 (-.02 ,.01)	-.10 (-.13,- .07)	-.13 (-.22,- .03)	.00 (-.07, .04)	-.13 (-.19,- .08)	-.20 (-.24,- .16)	.00 (- .02,.02)	-.09 (-.12,- .05)	-.19 (-.22,- .14)	.00 (- .04,.02)	-.10 (-.13,- .07)
Big 5 Extraversion	-.25 (-.28,- .20)	.00 (-.04 ,.01)	-.16 (-.19,- .13)	-.16 (-.25,- .04)	-.03 (-.11, .03)	-.16 (-.22,- .11)	-.28 (-.32,- .23)	.00 (- .04,.01)	-.16 (-.20,- .13)	-.26 (-.30,- .18)	.00 (- .07,.01)	-.15 (-.18,- .12)
Big 5 Neuroticism	.35 (.28 ,.39)	.00 (-.02 ,.06)	.31 (.28 ,.35)	.29 (.12, .39)	.01 (-.04, .13)	.34 (.28, .41)	.38 (.30,.42)	.00 (- .01,.06)	.30 (.26,.34)	.35 (.25,.41)	.01 (- .02,.10)	.30 (.27,.34)
Big 5 Openness	.08 (.05 ,.12)	.00 (-.02 ,.02)	.03 (.00 ,.06)	.06 (-.04, .15)	.00 (-.05, .08)	.10 (.05, .16)	.08 (.04,.12)	.00 (- .02,.03)	.00 (- .04,.03)	.07 (.01,.12)	.00 (- .04,.04)	.03 (.00,.06)
Self-control	-.27 (-.30,- .22)	.00 (-.03 ,.01)	-.19 (-.22,- .16)	-.21 (-.30,- .08)	-.02 (-.11, .04)	-.23 (-.30,- .18)	-.29 (-.33,- .24)	.00 (- .04,.01)	-.17 (-.20,- .13)	-.28 (-.32,- .20)	.00 (- .07,.01)	-.18 (-.21,- .15)
Not planning for the future	.11 (.04 ,.15)	.00 (-.03 ,.05)	.07 (.04 ,.10)	.14 (.02, .25)	.00 (-.07, .08)	.06 (.00, .12)	.08 (- .01,.15)	.01 (- .04,.07)	.07 (.04,.11)	.10 (.02,.16)	.00 (- .05,.07)	.07 (.03,.10)
Ambition	-.12 (-.18,- .08)	.00 (-.02 ,.05)	-.04 (-.07,- .01)	-.14 (-.25,- .02)	.02 (-.06, .09)	-.06 (-.12, .00)	-.11 (-.19,- .07)	.00 (- .03,.05)	-.03 (- .06,.00)	-.14 (-.21,- .07)	.02 (- .04,.07)	-.04 (- .07,.00)
Physical activity	-.12 (-.16,- .06)	.00 (-.05 ,.02)	-.01 (-.04 ,.02)	-.12 (-.22, .01)	-.01 (-.10, .06)	-.04 (-.10, .02)	-.12 (-.16,- .04)	.00 (- .06,.02)	.00 (- .03,.04)	-.08 (- .15,.00)	-.03 (- .10,.02)	-.01 (- .04,.02)
Health behaviours	-.16 (-.21,- .09)	-.01 (-.06 ,.03)	-.04 (-.06,- .01)	-.21 (-.31,- .09)	.04 (-.05, .10)	-.01 (-.06, .04)	-.13 (-.20,- .05)	-.03 (- .10,.02)	-.05 (-.08,- .02)	-.11 (-.19,- .03)	-.05 (- .11,.02)	-.03 (- .06,.00)
Risk taking behaviour	.05 (.00 ,.09)	.00 (-.03 ,.04)	.06 (.03 ,.09)	.03 (-.12, .15)	-.02 (-.11, .09)	.07 (.02, .13)	.07 (.03,.12)	.00 (- .02,.04)	.06 (.02,.09)	.05 (- .03,.11)	.01 (- .04,.07)	.06 (.03,.09)
Risky sexual behaviour	.05 (.00 ,.08)	.00 (-.03 ,.03)	-.01 (-.03 ,.02)	.12 (.01, .23)	-.04 (-.12, .04)	-.04 (-.09, .00)	.03 (- .04,.08)	.01 (- .03,.06)	.00 (- .03,.03)	.06 (- .03,.14)	-.02 (- .08,.06)	-.01 (- .04,.02)
Media use	.01 (-.03 ,.04)	.00 (-.02 ,.02)	.04 (.01 ,.06)	-.03 (-.11, .06)	-.01 (-.06, .05)	.05 (.00, .10)	.03 (- .01,.07)	.00 (- .03,.03)	.03 (.00,.06)	.00 (- .05,.05)	.00 (- .04,.03)	.03 (.00,.06)
Volunteering	.02 (-.04 ,.06)	.00 (-.02 ,.05)	.01 (-.01 ,.04)	-.09 (-.20, .03)	.06 (-.02, .14)	.06 (.00, .11)	.04 (- .02,.08)	.00 (- .04,.04)	.00 (- .03,.03)	.00 (- .08,.08)	.01 (- .04,.08)	.02 (- .01,.05)
Internet dating	.04 (-.01 ,.10)	.00 (-.04 ,.04)	.06 (.03 ,.08)	.03 (-.12, .15)	-.02 (-.10, .09)	.08 (.02, .14)	.06 (- .01,.10)	.00 (- .04,.05)	.04 (.01,.08)	.00 (- .08,.09)	.04 (- .03,.10)	.05 (.02,.08)

Internet dating	.04 (.00 ,.09)	.00 (-.03 ,.03)	.05 (.02 ,.08)	.02 (-.11, .14)	.00 (-.09, .09)	.07 (.01, .13)	.06 (.00,.11)	.00 (- .03,.04)	.04 (.00,.08)	-.01 (- .08,.09)	.05 (- .03,.11)	.04 (.01,.07)
Financial literacy: knowledge of products	-.13 (-.16,- .10)	.00 (-.03 ,.02)	-.02 (-.05 ,.01)	-.09 (-.21, .03)	-.02 (-.11, .06)	-.03 (-.09, .02)	-.14 (-.18,- .09)	.00 (- .03,.03)	-.01 (- .05,.03)	-.14 (-.21,- .05)	.01 (- .06,.06)	-.02 (- .05,.01)
Financial attitudes and behaviour	-.10 (-.13,- .06)	.00 (-.02 ,.02)	-.08 (-.11,- .05)	-.08 (-.19, .04)	.00 (-.08, .07)	-.11 (-.17,- .05)	-.10 (-.14,- .06)	.00 (- .03,.03)	-.07 (-.11,- .03)	-.09 (-.15,- .03)	.00 (- .05,.04)	-.07 (-.10,- .04)

Trait	Model fitting results											
	Functional outcome: adverse physical health											
	Whole sample			Males only			Females only			Same-sex MZ and DZ twins		
	A	C	E	A	C	E	A	C	E	A	C	E
Daily hassles	.16 (.08 ,.21)	.00 (-.04 ,.07)	.12 (.09 ,.15)	.16 (.08, .25)	.00 (-.06, .05)	.12 (.06, .17)	.11 (.01,.22)	.04 (- .04,.12)	.12 (.08,.16)	.14 (.04,.22)	.03 (- .04,.11)	.12 (.08,.15)
CHAOS	.06 (-.02 ,.14)	.07 (.01 ,.14)	.09 (.06 ,.12)	.12 (.00, .24)	-.01 (-.08, .08)	.06 (.00, .11)	.02 (- .08,.12)	.12 (.04,.20)	.10 (.06,.14)	.02 (- .08,.11)	.13 (.05,.21)	.09 (.06,.12)
Childhood experiences	.13 (.06 ,.21)	.01 (-.05 ,.08)	.07 (.05 ,.10)	.02 (-.10, .12)	.02 (-.04, .11)	.15 (.10, .20)	.16 (.07,.25)	.02 (- .06,.11)	.05 (.02,.08)	.14 (.05,.23)	.01 (- .07,.09)	.07 (.05,.10)
Poor sleep quality	.23 (.14 ,.29)	-.01 (-.05 ,.07)	.15 (.12 ,.19)	.19 (.06, .27)	.01 (-.03, .11)	.16 (.11, .22)	.27 (.15,.33)	-.03 (- .06,.07)	.15 (.11,.19)	.19 (.08,.30)	.04 (- .05,.14)	.15 (.12,.19)
Marriage hopes	.02 (-.06 ,.08)	-.06 (-.11 ,.00)	.00 (-.04 ,.03)	.00 (-.12, .11)	-.04 (-.11, .05)	.02 (-.04, .08)	.02 (- .07,.10)	-.07 (- .13,.00)	-.01 (- .05,.02)	.04 (- .06,.13)	-.08 (- .15,.01)	-.01 (- .04,.02)
Marriage worries	.04 (-.03 ,.11)	.04 (-.01 ,.09)	.05 (.02 ,.08)	.11 (.02, .17)	.00 (-.04, .06)	.00 (-.05, .05)	-.01 (- .08,.09)	.07 (- .01,.14)	.08 (.05,.12)	.01 (- .08,.11)	.06 (- .03,.13)	.06 (.03,.09)
Quality of relationship with twin	-.07 (-.13 ,.00)	-.04 (-.09 ,.02)	.01 (-.01 ,.03)	-.05 (-.15, .05)	-.02 (-.11, .06)	.02 (-.02, .06)	-.08 (- .16,.00)	-.03 (- .10,.03)	.00 (- .02,.03)	-.07 (- .14,.01)	-.07 (- .13,.00)	.00 (- .02,.03)
Quality of relationship with mother	-.06 (-.12 ,.00)	.02 (-.03 ,.07)	-.03 (-.06,- .01)	-.01 (-.07, .04)	.00 (-.03, .06)	-.04 (-.09, .01)	-.08 (- .16,.03)	.03 (- .06,.09)	-.03 (- .07,.00)	-.06 (- .15,.04)	.01 (- .07,.09)	-.03 (- .06,.00)
Quality of relationship with father	-.11 (-.19,- .03)	.03 (-.04 ,.09)	-.02 (-.05 ,.00)	-.07 (-.19, .05)	.02 (-.07, .10)	-.05 (-.10, .00)	-.12 (-.21,- .02)	.02 (- .07,.10)	-.01 (- .05,.02)	-.08 (- .18,.01)	-.01 (- .09,.07)	-.02 (- .05,.00)
Number of relationships	.13 (.07 ,.16)	.00 (-.02 ,.04)	.02 (-.01 ,.04)	.09 (.04, .15)	.00 (-.04, .03)	.02 (-.03, .06)	.11 (.02,.18)	.04 (- .02,.10)	.02 (- .01,.06)	.09 (.00,.19)	.04 (- .05,.11)	.02 (- .01,.05)
Longest relationship	.04 (-.02 ,.09)	.00 (-.02 ,.05)	-.04 (-.07,- .01)	.04 (-.06, .10)	.00 (-.03, .07)	-.07 (-.13,- .01)	.05 (- .03,.12)	.01 (- .04,.06)	-.02 (- .06,.02)	.01 (- .07,.10)	.03 (- .04,.09)	-.03 (- .06,.01)
Partner violence	.16 (.08 ,.22)	.01 (-.03 ,.07)	.05 (.02 ,.09)	.13 (.03, .23)	-.01 (-.08, .05)	.05 (.00, .11)	.15 (.05,.24)	.05 (- .03,.13)	.06 (.02,.10)	.15 (.05,.24)	.03 (- .05,.11)	.05 (.02,.09)
Contact with mother	.04 (-.06 ,.15)	.03 (-.06 ,.10)	-.02 (-.07 ,.02)	.00 (-.16, .15)	.04 (-.06, .15)	-.02 (-.10, .06)	.08 (- .07,.23)	-.01 (- .12,.11)	-.03 (- .09,.02)	.03 (- .12,.18)	.04 (- .09,.16)	-.02 (- .07,.02)
Communication with mother	-.05 (-.16 ,.08)	.04 (-.06 ,.12)	-.02 (-.06 ,.02)	-.03 (-.19, .15)	.04 (-.09, .15)	-.05 (-.13, .02)	-.02 (- .18,.13)	.01 (- .12,.14)	-.02 (- .07,.03)	-.05 (- .19,.11)	.04 (- .09,.17)	-.02 (- .06,.02)
Contact with father	-.01 (-.13 ,.11)	.02 (-.07 ,.11)	-.01 (-.05 ,.04)	.00 (-.17, .18)	.01 (-.12, .14)	-.01 (-.09, .07)	-.01 (- .16,.14)	.02 (- .10,.14)	-.01 (- .06,.04)	-.01 (- .16,.13)	.02 (- .10,.14)	-.01 (- .05,.04)
Communication with father	-.01 (-.10 ,.09)	-.04 (-.12 ,.04)	-.04 (-.07,- .01)	-.03 (-.19, .14)	-.02 (-.14, .11)	-.01 (-.07, .06)	.01 (- .07,.12)	-.06 (- .16,.04)	-.05 (-.09,- .02)	-.03 (- .15,.04)	-.02 (- .12,.08)	-.04 (-.07,- .01)

Peer pressure	.11 (.05 ,.17)	-.01 (-.05 ,.03)	.05 (.02 ,.08)	.08 (.01, .17)	.00 (-.06, .04)	.07 (.02, .12)	.12 (.03,.19)	-.01 (- .07,.05)	.04 (.00,.08)	.14 (.04,.21)	-.02 (- .08,.05)	.05 (.02,.08)
Physical peer victimisation	.08 (.00 ,.13)	.01 (-.02 ,.07)	.04 (.01 ,.08)	.01 (-.12, .10)	.02 (-.02, .12)	.07 (.02, .13)	.14 (.05,.20)	.00 (- .04,.07)	.01 (- .04,.06)	.07 (- .02,.15)	.01 (- .05,.09)	.06 (.02,.10)
Social peer victimisation	.16 (.08 ,.23)	.01 (-.04 ,.07)	.06 (.02 ,.09)	.10 (-.04, .22)	.01 (-.06, .11)	.08 (.01, .15)	.18 (.09,.26)	.01 (- .04,.09)	.05 (.01,.09)	.13 (.02,.24)	.04 (- .05,.13)	.05 (.02,.09)
Verbal peer victimisation	.17 (.10 ,.23)	.00 (-.04 ,.05)	.07 (.03 ,.10)	.11 (.00, .21)	.02 (-.05, .10)	.05 (-.01, .11)	.20 (.12,.27)	-.01 (- .06,.05)	.08 (.04,.12)	.14 (.04,.24)	.04 (- .05,.12)	.06 (.03,.10)
Cyber peer victimisation	.19 (.13 ,.25)	.00 (-.04 ,.04)	.06 (.02 ,.09)	.04 (-.10, .17)	.06 (-.03, .16)	.10 (.03, .16)	.23 (.16,.30)	.00 (- .05,.05)	.05 (.01,.09)	.19 (.11,.26)	.00 (- .05,.07)	.06 (.02,.09)
Physical peer perpetration	.08 (.01 ,.12)	.00 (-.03 ,.04)	.00 (-.04 ,.03)	.05 (-.07, .12)	.00 (-.04, .09)	.02 (-.03, .08)	.13 (.06,.18)	-.01 (- .04,.04)	-.05 (- .09,.00)	.10 (- .01,.18)	-.03 (- .10,.08)	-.01 (- .05,.03)
Social peer perpetration	.10 (.04 ,.16)	.00 (-.04 ,.04)	.07 (.03 ,.10)	.09 (.00, .17)	.00 (-.05, .05)	.06 (.00, .13)	.11 (.01,.19)	-.01 (- .06,.07)	.07 (.03,.12)	.12 (.01,.21)	-.02 (- .08,.07)	.06 (.02,.10)
Verbal peer perpetration	.11 (.07 ,.17)	.00 (-.03 ,.03)	.02 (-.01 ,.05)	.10 (.04, .15)	.00 (-.04, .05)	.00 (-.05, .05)	.14 (.06,.21)	-.01 (- .06,.05)	.03 (- .01,.08)	.10 (.01,.19)	.01 (- .06,.09)	.02 (- .01,.05)
Cyber peer perpetration	.10 (.05 ,.15)	.00 (-.03 ,.03)	.05 (.01 ,.08)	.07 (-.03, .13)	.00 (-.03, .06)	.03 (-.03, .10)	.14 (.06,.22)	-.02 (- .07,.04)	.05 (.01,.09)	.11 (.02,.19)	.00 (- .06,.07)	.05 (.01,.08)
Parental role aspirations	-.02 (-.07 ,.04)	.00 (-.04 ,.04)	-.03 (-.06 ,.00)	.05 (-.01, .14)	.00 (-.06, .03)	-.09 (-.15,- .04)	-.07 (- .15,.04)	.02 (- .06,.09)	.00 (- .04,.04)	-.01 (- .08,.07)	-.01 (- .07,.05)	-.03 (- .06,.00)
Occupational role aspirations	-.05 (-.11 ,.01)	.01 (-.03 ,.06)	.00 (-.04 ,.03)	-.02 (-.13, .05)	.01 (-.03, .09)	-.03 (-.09, .03)	-.05 (- .14,.04)	.01 (- .06,.07)	.01 (- .03,.05)	-.08 (- .16,.02)	.03 (- .06,.10)	.00 (- .03,.03)
Homecare role aspirations	-.02 (-.06 ,.05)	-.01 (-.06 ,.02)	.01 (-.02 ,.04)	-.04 (-.09, .02)	.00 (-.07, .03)	-.01 (-.06, .04)	.00 (- .08,.08)	-.02 (- .08,.05)	.02 (- .02,.05)	.01 (- .09,.10)	-.04 (- .11,.05)	.00 (- .04,.03)
Importance of relationships	.00 (-.03 ,.05)	.00 (-.04 ,.02)	-.02 (-.05 ,.01)	.05 (.00, .15)	.00 (-.07, .02)	-.03 (-.08, .01)	-.03 (- .09,.03)	.00 (- .04,.04)	-.01 (- .05,.03)	.02 (- .04,.09)	-.02 (- .08,.02)	-.02 (- .05,.01)
Achievement motivation	-.03 (-.09 ,.03)	.00 (-.04 ,.04)	.00 (-.03 ,.03)	.02 (-.07, .13)	-.03 (-.10, .04)	-.02 (-.07, .04)	-.05 (- .13,.02)	.01 (- .04,.07)	.01 (- .02,.05)	-.01 (- .11,.07)	-.02 (- .09,.07)	.00 (- .03,.04)
Purpose in life	-.15 (-.21,- .08)	.02 (-.03 ,.06)	-.11 (-.14,- .08)	-.12 (-.22,- .02)	.04 (-.04, .10)	-.12 (-.17,- .06)	-.16 (-.23,- .06)	.00 (- .07,.06)	-.11 (-.14,- .07)	-.18 (-.26,- .08)	.04 (- .05,.10)	-.11 (-.14,- .07)
Importance of democracy and equality	.01 (-.04 ,.06)	.00 (-.03 ,.03)	-.02 (-.05 ,.01)	.07 (-.02, .16)	.00 (-.06, .06)	-.06 (-.11,- .01)	-.02 (- .08,.05)	.00 (- .04,.04)	-.01 (- .04,.03)	.00 (- .06,.08)	.00 (- .07,.04)	-.02 (- .05,.01)
Environmental concerns	-.05 (-.11 ,.01)	-.01 (-.05 ,.04)	-.03 (-.05 ,.00)	-.05 (-.11, .06)	.00 (-.08, .03)	-.04 (-.08, .01)	-.08 (- .16,.02)	.01 (- .07,.08)	-.02 (- .05,.02)	-.04 (- .14,.05)	-.02 (- .10,.06)	-.03 (- .06,.00)
Religiosity	.03 (-.03 ,.10)	-.02 (-.07 ,.04)	.00 (-.02 ,.03)	.03 (-.08, .13)	-.01 (-.09, .08)	-.01 (-.05, .03)	.04 (- .04,.13)	-.03 (- .10,.04)	.01 (- .02,.03)	.05 (- .03,.13)	-.03 (- .10,.04)	.00 (- .02,.03)
Importance of leisure	-.11 (-.16,- .03)	-.01 (-.07 ,.02)	-.10 (-.13,- .07)	-.03 (-.11, .10)	-.02 (-.11, .03)	-.13 (-.19,- .08)	-.14 (-.21,- .05)	-.01 (- .08,.04)	-.09 (-.13,- .05)	-.08 (- .17,.01)	-.05 (- .12,.03)	-.10 (-.14,- .07)
Alcohol use	.13 (.05 ,.19)	-.04 (-.08 ,.02)	.02 (-.01 ,.05)	.16 (.03, .27)	-.06 (-.14, .03)	.01 (-.05, .06)	.10 (.01,.18)	-.01 (- .07,.06)	.03 (- .01,.07)	.17 (.06,.25)	-.08 (- .14,.02)	.01 (- .02,.05)
Ever smoked	.07 (.01 ,.12)	.00 (-.04 ,.04)	.04 (.01 ,.07)	.06 (-.01, .12)	.00 (-.05, .04)	.04 (-.01, .09)	.05 (- .04,.14)	.02 (- .05,.09)	.04 (.01,.08)	.07 (- .02,.14)	.00 (- .06,.07)	.04 (.01,.07)
Ever vaped	.12 (.04 ,.19)	-.01 (-.06 ,.06)	.03 (.00 ,.07)	.09 (-.01, .19)	.00 (-.06, .07)	.05 (.00, .10)	.13 (.01,.23)	.00 (- .08,.10)	.03 (- .01,.07)	.10 (- .01,.20)	.01 (- .08,.11)	.03 (.00,.06)

Cognitive enhancers	.15 (.08 ,.22)	-.02 (-.07 ,.04)	.04 (.01 ,.07)	.08 (-.01, .18)	.00 (-.07, .06)	.08 (.02, .14)	.18 (.06,.27)	-.02 (- .09,.07)	.02 (- .02,.06)	.16 (.05,.26)	-.02 (- .11,.07)	.04 (.00,.07)
Big 5 Agreeableness	-.04 (-.07 ,.01)	.00 (-.03 ,.02)	-.02 (-.05 ,.01)	.00 (-.08, .06)	.00 (-.04, .05)	-.04 (-.09, .02)	-.05 (- .10,.02)	.00 (- .05,.03)	-.01 (- .05,.03)	-.04 (- .10,.03)	.00 (- .05,.04)	-.02 (- .06,.01)
Big 5 Conscientiousness	-.10 (-.13,- .05)	.00 (-.03 ,.02)	-.06 (-.09,- .03)	-.05 (-.10, .01)	.00 (-.04, .03)	-.08 (-.13,- .02)	-.12 (-.18,- .05)	-.01 (- .05,.03)	-.05 (-.09,- .01)	-.09 (-.15,- .03)	-.01 (- .07,.03)	-.07 (-.10,- .03)
Big 5 Extraversion	-.11 (-.14,- .05)	.00 (-.04 ,.01)	-.05 (-.08,- .02)	-.05 (-.10, .03)	.00 (-.05, .02)	-.08 (-.13,- .03)	-.13 (-.18,- .05)	-.02 (- .07,.02)	-.03 (- .07,.00)	-.09 (-.15,- .02)	-.03 (- .08,.02)	-.06 (-.09,- .02)
Big 5 Neuroticism	.19 (.11 ,.24)	-.01 (-.04 ,.05)	.12 (.09 ,.15)	.15 (.03, .25)	-.01 (-.07, .08)	.15 (.10, .21)	.19 (.10,.26)	.01 (- .04,.08)	.11 (.07,.15)	.20 (.10,.28)	-.01 (- .08,.08)	.12 (.09,.15)
Big 5 Openness	.04 (.00 ,.09)	.00 (-.03 ,.02)	-.01 (-.04 ,.02)	.04 (-.02, .09)	.00 (-.05, .03)	-.01 (-.06, .04)	.03 (- .02,.10)	.00 (- .05,.04)	-.01 (- .05,.03)	.04 (- .03,.11)	.00 (- .05,.05)	-.02 (- .05,.01)
Self-control	-.15 (-.19,- .10)	.00 (-.03 ,.03)	-.11 (-.14,- .08)	-.12 (-.20,- .05)	.00 (-.04, .05)	-.10 (-.16,- .05)	-.16 (-.22,- .09)	.00 (- .06,.04)	-.11 (-.15,- .07)	-.16 (-.23,- .08)	.00 (- .06,.05)	-.11 (-.14,- .07)
Not planning for the future	.01 (-.06 ,.08)	.02 (-.03 ,.07)	.06 (.03 ,.10)	.03 (-.04, .12)	.00 (-.06, .04)	.07 (.02, .13)	-.05 (- .14,.05)	.07 (.00,.14)	.07 (.03,.11)	-.01 (- .09,.08)	.04 (- .04,.10)	.07 (.03,.10)
Ambition	-.06 (-.12,- .01)	.00 (-.03 ,.05)	-.02 (-.05 ,.01)	.00 (-.11, .07)	.00 (-.04, .07)	-.09 (-.14,- .03)	-.07 (- .15,.01)	.00 (- .06,.05)	.00 (- .04,.04)	-.07 (- .15,.01)	.00 (- .07,.06)	-.02 (- .05,.01)
Physical activity	-.08 (-.12,- .01)	.00 (-.05 ,.02)	-.04 (-.07,- .02)	-.05 (-.10, .05)	.00 (-.07, .03)	-.06 (-.11,- .01)	-.09 (-.16,- .01)	-.01 (- .07,.05)	-.04 (- .07,.00)	-.04 (- .13,.04)	-.04 (- .11,.03)	-.05 (-.08,- .02)
Health behaviours	-.09 (-.16,- .03)	-.03 (-.08 ,.02)	-.05 (-.08,- .03)	-.08 (-.14, .00)	.00 (-.06, .04)	-.08 (-.12,- .03)	-.07 (- .17,.01)	-.06 (- .12,.02)	-.04 (-.08,- .01)	-.06 (- .16,.02)	-.06 (- .13,.02)	-.05 (-.08,- .03)
Risk taking behaviour	.08 (.02 ,.14)	-.01 (-.05 ,.03)	.07 (.04 ,.10)	.10 (.00, .20)	-.03 (-.09, .05)	.06 (.01, .11)	.07 (.00,.14)	.00 (- .05,.05)	.07 (.03,.11)	.10 (.01,.17)	-.02 (- .08,.05)	.06 (.03,.09)
Risky sexual behaviour	.08 (.02 ,.13)	.00 (-.03 ,.05)	.03 (.00 ,.05)	.09 (.02, .19)	-.01 (-.08, .03)	.02 (-.03, .06)	.03 (- .04,.12)	.04 (- .02,.11)	.03 (.00,.06)	.07 (- .03,.16)	.01 (- .07,.10)	.03 (.00,.05)
Media use	.02 (-.02 ,.05)	.00 (-.02 ,.03)	.05 (.03 ,.08)	.01 (-.04, .06)	.00 (-.03, .03)	.04 (.00, .09)	.02 (- .05,.08)	.01 (- .03,.06)	.06 (.03,.09)	.00 (- .06,.06)	.02 (- .03,.07)	.05 (.02,.08)
Volunteering	.07 (.02 ,.13)	-.01 (-.05 ,.03)	.02 (-.01 ,.05)	.06 (-.03, .13)	.00 (-.04, .06)	.02 (-.03, .06)	.10 (.02,.17)	-.02 (- .08,.04)	.02 (- .02,.05)	.07 (- .03,.15)	.00 (- .07,.08)	.01 (- .02,.04)
Internet dating	-.01 (-.08 ,.06)	.03 (-.02 ,.08)	.05 (.02 ,.08)	-.01 (-.11, .07)	.00 (-.05, .08)	.04 (-.01, .09)	-.01 (- .10,.08)	.04 (- .03,.11)	.06 (.02,.10)	-.06 (- .14,.03)	.07 (.00,.14)	.06 (.02,.09)
Internet dating	.03 (-.04 ,.08)	.01 (-.03 ,.06)	.04 (.00 ,.07)	.00 (-.09, .09)	.00 (-.05, .06)	.04 (-.01, .09)	.03 (- .05,.11)	.02 (- .03,.09)	.04 (.00,.08)	-.04 (- .12,.04)	.08 (.00,.14)	.04 (.00,.07)
Financial literacy: knowledge of products	-.03 (-.08 ,.03)	.00 (-.05 ,.02)	-.02 (-.05 ,.01)	-.04 (-.09, .01)	.00 (-.06, .05)	-.04 (-.08, .01)	-.01 (- .08,.07)	-.02 (- .08,.03)	-.01 (- .05,.02)	.00 (- .09,.09)	-.04 (- .11,.04)	-.03 (- .06,.00)
Financial attitudes and behaviour	-.05 (-.09 ,.00)	.00 (-.04 ,.02)	-.10 (-.13,- .07)	-.04 (-.11, .04)	.00 (-.05, .04)	-.14 (-.20,- .09)	-.05 (- .11,.03)	-.01 (- .07,.03)	-.08 (-.12,- .05)	-.05 (- .12,.03)	.00 (- .06,.06)	-.10 (-.14,- .07)

Trait	Model fitting results											
	Functional outcome: achieved educational level											
	Whole sample			Males only			Females only			Same-sex MZ and DZ twins		
	A	C	E	A	C	E	A	C	E	A	C	E

Daily hassles	.02 (-.06 ,.10)	-.01 (-.07 ,.06)	.00 (-.03 ,.03)	.00 (-.12, .13)	.00 (-.09, .09)	-.01 (-.06, .05)	.03 (- .07,.14)	-.02 (- .10,.07)	.00 (- .04,.04)	.03 (- .07,.12)	-.01 (- .08,.07)	.00 (- .03,.04)
CHAOS	-.02 (-.09 ,.06)	-.07 (-.13,- .02)	.00 (-.02 ,.03)	-.10 (-.23, .02)	-.03 (-.12, .07)	.05 (.00, .10)	.02 (- .07,.11)	-.09 (-.16,- .02)	-.02 (- .05,.02)	-.02 (- .10,.07)	-.07 (- .14,.00)	.01 (- .02,.04)
Childhood experiences	-.02 (-.09 ,.05)	-.03 (-.09 ,.03)	.01 (-.02 ,.03)	-.09 (-.22, .04)	-.01 (-.11, .09)	.04 (-.01, .08)	.00 (- .08,.09)	-.03 (- .11,.04)	.00 (- .03,.02)	-.01 (- .10,.08)	-.04 (- .11,.04)	.01 (- .02,.03)
Poor sleep quality	.06 (-.01 ,.13)	-.10 (-.15,- .04)	-.02 (-.05 ,.01)	.07 (-.05, .17)	-.11 (-.17,- .01)	-.02 (-.07, .04)	.06 (- .04,.14)	-.10 (-.16,- .02)	-.02 (- .06,.02)	.11 (.01,.18)	-.15 (-.20,- .06)	-.02 (- .05,.01)
Marriage hopes	.14 (.06 ,.22)	-.03 (-.09 ,.03)	-.02 (-.05 ,.01)	.14 (.02, .23)	-.04 (-.10, .05)	-.02 (-.07, .03)	.12 (.02,.22)	-.01 (- .09,.07)	-.02 (- .05,.02)	.14 (.05,.24)	-.04 (- .12,.04)	-.03 (- .06,.00)
Marriage worries	.01 (-.07 ,.09)	-.01 (-.07 ,.05)	-.03 (-.06 ,.00)	-.03 (-.13, .09)	.03 (-.05, .10)	-.02 (-.07, .03)	.04 (- .06,.14)	-.05 (- .13,.03)	-.03 (- .07,.00)	.02 (- .08,.11)	-.02 (- .10,.06)	-.03 (- .06,.00)
Quality of relationship with twin	.00 (-.06 ,.06)	.05 (.00 ,.10)	-.02 (-.04 ,.00)	-.01 (-.10, .08)	.07 (-.01, .14)	-.02 (-.05, .02)	.01 (- .07,.08)	.04 (- .02,.10)	-.02 (- .04,.01)	.01 (- .06,.08)	.06 (- .01,.12)	-.01 (- .04,.01)
Quality of relationship with mother	.00 (-.07 ,.07)	.02 (-.04 ,.08)	.00 (-.03 ,.02)	.02 (-.08, .13)	.02 (-.06, .09)	-.01 (-.06, .03)	.00 (- .09,.09)	.01 (- .06,.09)	.00 (- .03,.03)	.04 (- .05,.13)	-.02 (- .10,.05)	.00 (- .03,.02)
Quality of relationship with father	.00 (-.07 ,.07)	.05 (-.01 ,.10)	.01 (-.02 ,.03)	-.01 (-.13, .11)	.07 (-.02, .16)	-.01 (-.05, .04)	.01 (- .07,.10)	.03 (- .04,.10)	.01 (- .02,.04)	.04 (- .04,.13)	.00 (- .07,.08)	.01 (- .02,.03)
Number of relationships	-.08 (-.14,- .01)	-.01 (-.05 ,.04)	-.01 (-.03 ,.02)	-.10 (-.19,- .01)	.04 (-.03, .10)	-.04 (-.08, .01)	-.03 (- .11,.05)	-.06 (- .12,.00)	.01 (- .02,.04)	-.05 (- .14,.03)	-.05 (- .12,.03)	-.01 (- .04,.02)
Longest relationship	.00 (-.07 ,.07)	-.05 (-.09 ,.00)	-.03 (-.06 ,.00)	.03 (-.09, .14)	-.06 (-.14, .02)	-.04 (-.10, .02)	-.02 (- .11,.07)	-.04 (- .10,.03)	-.03 (- .07,.01)	.00 (- .09,.08)	-.05 (- .12,.02)	-.04 (-.07,- .01)
Partner violence	-.06 (-.14 ,.02)	-.04 (-.10 ,.02)	-.01 (-.04 ,.02)	-.16 (-.27,- .03)	.02 (-.08, .09)	.03 (-.02, .09)	-.02 (- .12,.07)	-.06 (- .13,.02)	-.02 (- .06,.02)	-.07 (- .16,.02)	-.02 (- .10,.05)	.00 (- .04,.03)
Contact with mother	-.14 (-.26,- .02)	-.04 (-.14 ,.06)	-.06 (-.11,- .02)	-.10 (-.32, .10)	-.03 (-.19, .13)	-.07 (-.15, .02)	-.16 (-.30,- .01)	-.05 (- .17,.07)	-.06 (-.12,- .01)	-.07 (- .17,.07)	-.11 (-.22,- .02)	-.06 (-.10,- .02)
Communication with mother	-.01 (-.12 ,.11)	.00 (-.10 ,.10)	-.02 (-.06 ,.02)	.06 (-.15, .26)	-.07 (-.23, .09)	.00 (-.08, .07)	-.05 (- .19,.10)	.05 (- .08,.17)	-.02 (- .07,.03)	-.03 (- .18,.12)	.02 (- .11,.15)	-.02 (- .06,.03)
Contact with father	-.21 (-.29,- .12)	.06 (-.02 ,.13)	.00 (-.03 ,.04)	-.21 (-.32,- .05)	.10 (-.03, .19)	-.02 (-.08, .05)	-.19 (-.31,- .05)	.02 (- .08,.13)	.01 (- .04,.06)	-.13 (-.25,- .01)	-.02 (- .12,.09)	.01 (- .03,.05)
Communication with father	-.02 (-.11 ,.07)	.06 (-.01 ,.14)	.00 (-.04 ,.03)	-.07 (-.24,- .01)	.10 (-.03, .23)	-.01 (-.07, .05)	.00 (- .11,.10)	.05 (- .04,.14)	.00 (- .04,.04)	.06 (.00,.16)	-.01 (- .10,.09)	.00 (- .03,.03)
Peer pressure	.02 (-.05 ,.09)	.02 (-.03 ,.07)	-.02 (-.05 ,.01)	-.01 (-.12, .10)	.03 (-.06, .10)	-.01 (-.05, .04)	.04 (- .05,.13)	.02 (- .05,.09)	-.03 (- .07,.01)	.01 (- .07,.10)	.04 (- .03,.10)	-.02 (- .05,.01)
Physical peer victimisation	-.04 (-.12 ,.03)	-.03 (-.08 ,.02)	.01 (-.03 ,.04)	-.03 (-.15, .08)	-.03 (-.11, .06)	.02 (-.03, .07)	-.05 (- .16,.05)	-.04 (- .11,.04)	.00 (- .05,.05)	-.01 (- .09,.08)	-.07 (- .13,.01)	.01 (- .02,.05)
Social peer victimisation	-.08 (-.16 ,.01)	.03 (-.04 ,.09)	.01 (-.03 ,.04)	-.13 (-.23, .01)	.04 (-.06, .12)	.07 (.01, .12)	-.04 (- .13,.05)	.01 (- .06,.08)	-.03 (- .07,.02)	-.09 (- .19,.01)	.05 (- .04,.13)	.01 (- .02,.05)
Verbal peer victimisation	-.01 (-.09 ,.07)	-.02 (-.08 ,.03)	.02 (-.02 ,.05)	.02 (-.12, .15)	-.06 (-.15, .05)	.04 (-.01, .10)	-.02 (- .11,.07)	-.01 (- .07,.05)	-.01 (- .05,.03)	.02 (- .07,.11)	-.05 (- .12,.03)	.02 (- .02,.05)
Cyber peer victimisation	-.07 (-.14 ,.00)	.00 (-.06 ,.05)	.01 (-.02 ,.04)	.00 (-.15, .15)	-.06 (-.17, .05)	.03 (-.03, .09)	-.09 (-.17,- .01)	.01 (- .05,.06)	-.01 (- .05,.03)	-.07 (- .15,.01)	.00 (- .07,.06)	.01 (- .02,.04)
Physical peer perpetration	-.05 (-.12 ,.03)	-.04 (-.10 ,.01)	-.01 (-.05 ,.03)	-.04 (-.17, .08)	-.06 (-.15, .03)	.00 (-.06, .05)	-.03 (- .14,.07)	-.04 (- .10,.03)	-.03 (- .08,.03)	.02 (- .08,.10)	-.11 (-.17,- .03)	-.01 (- .05,.03)

Social peer perpetration	-.01 (-.10 ,.06)	-.02 (-.08 ,.04)	-.02 (-.06 ,.02)	-.04 (-.16, .09)	-.01 (-.09, .07)	.01 (-.06, .07)	.00 (- .11,.11)	-.03 (- .11,.05)	-.03 (- .08,.01)	-.01 (- .12,.10)	-.02 (- .10,.07)	-.02 (- .06,.02)
Verbal peer perpetration	-.01 (-.08 ,.06)	-.01 (-.06 ,.04)	-.01 (-.04 ,.03)	.01 (-.11, .12)	-.04 (-.12, .04)	.04 (-.02, .09)	-.02 (- .11,.07)	.01 (- .06,.07)	-.04 (- .08,.00)	.02 (- .07,.11)	-.04 (- .11,.03)	-.01 (- .04,.03)
Cyber peer perpetration	-.03 (-.10 ,.05)	-.02 (-.07 ,.03)	-.02 (-.05 ,.02)	-.02 (-.14, .10)	-.05 (-.13, .03)	.02 (-.05, .08)	-.04 (- .13,.06)	.01 (- .06,.07)	-.04 (- .08,.01)	-.01 (- .10,.08)	-.04 (- .11,.03)	-.02 (- .06,.01)
Parental role aspirations	.03 (-.05 ,.10)	-.03 (-.09 ,.02)	-.04 (-.07,- .01)	.02 (-.09, .13)	-.03 (-.10, .05)	-.01 (-.07, .04)	.03 (- .08,.13)	-.04 (- .12,.04)	-.06 (-.09,- .02)	.03 (- .05,.11)	-.05 (- .11,.02)	-.04 (-.08,- .01)
Occupational role aspirations	.07 (-.01 ,.15)	.02 (-.04 ,.08)	.05 (.01 ,.08)	.09 (-.04, .20)	-.01 (-.09, .08)	.03 (-.02, .09)	.05 (- .04,.15)	.04 (- .03,.12)	.06 (.02,.10)	.07 (- .03,.17)	.02 (- .06,.10)	.05 (.01,.08)
Homecare role aspirations	-.07 (-.14 ,.00)	.02 (-.04 ,.07)	.01 (-.02 ,.04)	.01 (-.10, .11)	.00 (-.08, .07)	-.01 (-.06, .04)	-.13 (-.22,- .03)	.04 (- .04,.10)	.02 (- .02,.06)	-.09 (- .19,.00)	.04 (- .04,.12)	.01 (- .02,.04)
Importance of relationships	.01 (-.04 ,.07)	.01 (-.03 ,.05)	.01 (-.02 ,.04)	.00 (-.09, .09)	.02 (-.04, .08)	.02 (-.03, .06)	.02 (- .05,.09)	.00 (- .05,.05)	.00 (- .03,.04)	.01 (- .06,.07)	.01 (- .04,.06)	.01 (- .02,.04)
Achievement motivation	.03 (-.05 ,.10)	-.01 (-.06 ,.05)	.04 (.01 ,.07)	-.02 (-.15, .11)	.03 (-.07, .12)	.06 (.01, .12)	.05 (- .04,.13)	-.02 (- .08,.04)	.03 (- .01,.06)	.07 (- .02,.14)	-.06 (- .12,.02)	.04 (.01,.07)
Purpose in life	.06 (-.02 ,.13)	-.01 (-.07 ,.05)	.02 (-.01 ,.05)	.02 (-.10, .15)	.02 (-.07, .12)	.01 (-.04, .06)	.08 (- .01,.16)	-.03 (- .09,.04)	.02 (- .01,.06)	.04 (- .05,.13)	.00 (- .08,.08)	.02 (- .01,.05)
Importance of democracy and equality	.11 (.05 ,.18)	.02 (-.02 ,.07)	.02 (-.01 ,.05)	.10 (-.02, .22)	.01 (-.08, .10)	.01 (-.04, .06)	.12 (.04,.20)	.03 (- .03,.08)	.02 (- .01,.06)	.11 (.04,.19)	.02 (- .04,.08)	.02 (- .01,.05)
Environmental concerns	.11 (.04 ,.19)	.04 (-.02 ,.09)	.03 (.00 ,.06)	.08 (-.02, .19)	.06 (-.02, .14)	.05 (.00, .09)	.14 (.05,.23)	.02 (- .05,.09)	.02 (- .01,.05)	.03 (NA, NA)	.01 (- 1.00,1.00)	.00 (NA, NA)
Religiosity	.00 (-.06 ,.06)	.07 (.02 ,.12)	.00 (-.02 ,.02)	-.01 (-.11, .08)	.06 (-.02, .14)	.00 (-.03, .04)	.01 (- .06,.09)	.08 (.01,.14)	.00 (- .03,.02)	-.02 (- .09,.05)	.09 (.03,.16)	.00 (- .02,.02)
Importance of leisure	.05 (-.02 ,.13)	.07 (.01 ,.12)	.00 (-.03 ,.03)	.06 (-.06, .19)	.07 (-.03, .15)	-.01 (-.06, .04)	.05 (- .03,.15)	.07 (.00,.14)	.01 (- .03,.05)	.05 (- .03,.15)	.08 (.00,.14)	.01 (- .02,.04)
Alcohol use	.03 (-.06 ,.12)	-.03 (-.09 ,.04)	.01 (-.02 ,.04)	-.05 (-.18, .10)	.00 (-.11, .12)	.07 (.02, .13)	.07 (- .03,.16)	-.04 (- .11,.04)	-.02 (- .06,.01)	.00 (- .11,.10)	.00 (- .09,.09)	.01 (- .02,.04)
Ever smoked	.01 (-.06 ,.08)	-.07 (-.12,- .02)	-.01 (-.03 ,.02)	-.04 (-.15, .06)	-.02 (-.10, .06)	.00 (-.05, .05)	.05 (- .03,.13)	-.11 (-.17,- .05)	-.01 (- .04,.02)	.00 (- .09,.07)	-.07 (- .13,.00)	-.01 (- .04,.02)
Ever vaped	-.10 (-.19,- .02)	-.04 (-.10 ,.03)	-.01 (-.04 ,.02)	-.10 (-.24, .03)	-.05 (-.14, .05)	.01 (-.05, .06)	-.11 (- .21,.00)	-.02 (- .10,.06)	-.02 (- .06,.02)	-.07 (- .17,.03)	-.06 (- .14,.01)	-.01 (- .04,.02)
Cognitive enhancers	.07 (-.01 ,.14)	-.06 (-.11 ,.01)	-.01 (-.04 ,.02)	.05 (-.09, .17)	-.05 (-.13, .05)	.00 (-.06, .05)	.08 (- .02,.18)	-.06 (- .14,.02)	-.02 (- .06,.02)	.07 (- .03,.17)	-.06 (- .14,.03)	-.01 (- .05,.02)
Big 5 Agreeableness	.01 (-.05 ,.07)	.01 (-.03 ,.05)	.00 (-.03 ,.03)	.01 (-.10, .12)	.02 (-.06, .09)	.01 (-.05, .06)	.01 (- .07,.08)	.01 (- .04,.06)	-.01 (- .05,.03)	.01 (- .06,.07)	.01 (- .04,.06)	.00 (- .03,.03)
Big 5 Conscientiousness	.04 (-.02 ,.10)	.01 (-.03 ,.05)	.05 (.02 ,.08)	.00 (-.10, .09)	.02 (-.04, .09)	.05 (.00, .10)	.06 (- .02,.14)	.00 (- .05,.06)	.05 (.01,.09)	.03 (- .04,.10)	.02 (- .03,.07)	.05 (.01,.08)
Big 5 Extraversion	.00 (-.07 ,.05)	.04 (.00 ,.08)	.04 (.01 ,.07)	-.04 (-.14, .06)	.05 (-.02, .12)	.03 (-.02, .08)	.02 (- .06,.09)	.03 (- .02,.09)	.05 (.01,.08)	-.01 (- .08,.06)	.04 (- .02,.09)	.04 (.01,.07)
Big 5 Neuroticism	.03 (-.05 ,.10)	-.04 (-.09 ,.02)	-.02 (-.05 ,.01)	-.01 (-.15, .13)	-.04 (-.14, .06)	.01 (-.05, .06)	.05 (- .05,.13)	-.03 (- .10,.04)	-.03 (- .06,.01)	.03 (- .07,.13)	-.03 (- .10,.06)	-.01 (- .04,.02)
Big 5 Openness	.02 (-.04 ,.08)	.03 (-.01 ,.07)	.03 (.00 ,.06)	-.03 (-.13, .07)	.05 (-.02, .12)	.05 (.00, .10)	.05 (- .03,.13)	.02 (- .03,.07)	.02 (- .02,.05)	.02 (- .05,.09)	.03 (- .02,.09)	.04 (.01,.07)

Self-control	.07 (.01 ,.14)	-.01 (-.05 ,.04)	.00 (-.03 ,.03)	.07 (-.04, .18)	-.01 (-.08, .07)	.00 (-.06, .05)	.07 (-.01,.15)	-.01 (-.07,.05)	.00 (-.03,.04)	.07 (.00,.15)	-.01 (-.07,.05)	.00 (-.03,.03)
Not planning for the future	-.06 (-.15 ,.02)	-.02 (-.08 ,.04)	-.02 (-.05 ,.02)	-.05 (-.17, .06)	-.02 (-.10, .06)	-.01 (-.07, .04)	-.08 (-.18,.03)	-.02 (-.10,.06)	-.02 (-.06,.03)	-.09 (-.18,.00)	.00 (-.08,.07)	-.02 (-.05,.01)
Ambition	.06 (-.02 ,.13)	.02 (-.03 ,.08)	.04 (.01 ,.07)	.05 (-.07, .16)	.03 (-.06, .11)	.04 (-.01, .09)	.06 (-.03,.15)	.02 (-.05,.09)	.04 (.01,.08)	.06 (-.02,.15)	.01 (-.06,.08)	.04 (.01,.07)
Physical activity	.03 (-.03 ,.10)	.04 (-.01 ,.09)	.03 (.01 ,.06)	.11 (.00, .22)	-.01 (-.09, .07)	-.01 (-.06, .03)	-.01 (-.09,.08)	.07 (.01,.14)	.06 (.03,.10)	.00 (-.08,.09)	.07 (-.01,.13)	.03 (.00,.06)
Health behaviours	.10 (.03 ,.16)	.05 (.00 ,.11)	.02 (-.01 ,.04)	.13 (.02, .24)	.04 (-.04, .12)	-.01 (-.05, .04)	.09 (.00,.18)	.05 (.00,.13)	.03 (.00,.06)	.07 (-.01,.16)	.07 (.01,.14)	.01 (-.01,.04)
Risk taking behaviour	-.02 (-.09 ,.05)	.02 (-.04 ,.07)	.02 (-.01 ,.05)	-.17 (-.99, .99)	.19 (-.99, .99)	.03 (-.98, .98)	.02 (-.06,.11)	.01 (-.06,.07)	.00 (-.04,.03)	-.05 (-.13,.03)	.05 (-.02,.11)	.02 (-.01,.04)
Risky sexual behaviour	-.05 (-.12 ,.01)	-.04 (-.09 ,.01)	.00 (-.03 ,.02)	-.11 (-.21,-.01)	.00 (-.08, .08)	.00 (-.04, .04)	.00 (-.09,.07)	-.08 (-.14,-.01)	.00 (-.03,.03)	-.02 (-.10,.05)	-.08 (-.14,-.01)	.00 (-.03,.02)
Media use	.02 (-.04 ,.07)	-.03 (-.07 ,.01)	-.01 (-.04 ,.01)	-.07 (NA, NA)	-.05 (NA, NA)	.00 (NA, NA)	.02 (-.05,.10)	-.05 (-.10,.01)	-.02 (-.05,.02)	.01 (-.05,.07)	-.02 (-.07,.03)	-.02 (-.04,.01)
Volunteering	.06 (-.01 ,.13)	.02 (-.04 ,.07)	.02 (-.01 ,.04)	.08 (-.03, .19)	.00 (-.08, .09)	-.01 (-.06, .03)	.06 (-.03,.15)	.02 (-.05,.09)	.03 (.00,.07)	.04 (-.04,.13)	.04 (-.04,.11)	.01 (-.01,.04)
Internet dating	.04 (-.04 ,.12)	.01 (-.05 ,.07)	.02 (-.01 ,.05)	.10 (-.03, .21)	-.03 (-.11, .07)	.01 (-.04, .06)	.01 (-.08,.11)	.03 (-.04,.10)	.03 (-.01,.07)	.00 (-.09,.10)	.04 (-.04,.12)	.02 (-.01,.05)
Internet dating	.02 (-.05 ,.09)	.01 (-.04 ,.06)	.03 (.00 ,.06)	.08 (-.04, .19)	-.03 (-.11, .06)	.01 (-.04, .06)	-.01 (-.10,.08)	.03 (-.04,.10)	.04 (.00,.07)	-.02 (-.11,.08)	.04 (-.04,.12)	.03 (-.01,.06)
Financial literacy: knowledge of products	.02 (-.05 ,.09)	-.01 (-.06 ,.04)	.02 (-.01 ,.04)	.02 (-.09, .13)	.01 (-.08, .09)	.03 (-.01, .08)	.02 (-.07,.10)	-.01 (-.07,.05)	.00 (-.03,.04)	.02 (-.08,.11)	-.01 (-.08,.07)	.01 (-.02,.04)
Financial attitudes and behaviour	.04 (-.03 ,.11)	.00 (-.04 ,.05)	.02 (-.01 ,.05)	.01 (-.10, .12)	.03 (-.04, .11)	.01 (-.04, .06)	.06 (-.02,.14)	-.02 (-.08,.04)	.02 (-.02,.06)	.05 (-.03,.12)	-.01 (-.07,.05)	.01 (-.02,.05)

	Model fitting results											
	Functional outcome: planned educational level											
	Whole sample			Males only			Females only			Same-sex MZ and DZ twins		
Trait	A	C	E	A	C	E	A	C	E	A	C	E
Daily hassles	-.02 (-.09 ,.02)	.00 (-.04 ,.05)	.00 (-.03 ,.04)	-.07 (-.16, .04)	.00 (-.08, .05)	.01 (-.06, .07)	-.01 (-.12,.08)	.02 (-.05,.09)	.01 (-.05,.06)	-.01 (-.11,.05)	.00 (-.05,.08)	.01 (-.03,.05)
CHAOS	-.06 (-.14 ,.03)	-.04 (-.10 ,.03)	.02 (-.02 ,.05)	-.07 (-.21, .06)	-.02 (-.12, .08)	.04 (-.03, .10)	-.05 (-.16,.06)	-.04 (-.12,.04)	.01 (-.04,.05)	-.03 (-.14,.01)	-.06 (-.14,.03)	.02 (-.02,.06)
Childhood experiences	-.04 (-.12 ,.05)	.00 (-.07 ,.07)	.00 (-.03 ,.04)	.00 (-.13, .12)	-.05 (-.14, .05)	-.01 (-.07, .04)	-.08 (-.19,.03)	.04 (-.05,.13)	.02 (-.02,.06)	-.05 (-.16,.06)	.02 (-.08,.11)	.01 (-.02,.04)
Poor sleep quality	-.06 (-.14 ,.01)	.00 (-.06 ,.05)	.01 (-.03 ,.05)	-.08 (-.20, .06)	-.01 (-.11, .07)	.03 (-.04, .09)	-.06 (-.16,.04)	.00 (-.07,.08)	.01 (-.04,.06)	-.07 (-.19,.04)	.00 (-.10,.09)	.01 (-.04,.05)
Marriage hopes	.08 (-.01 ,.16)	.01 (-.05 ,.07)	-.03 (-.06 ,.01)	.09 (-.05, .19)	-.02 (-.10, .08)	-.02 (-.08, .05)	.06 (-.04,.17)	.03 (-.05,.11)	-.03 (-.07,.02)	.03 (-.07,.14)	.06 (-.04,.14)	-.02 (-.06,.01)
Marriage worries	.00 (-.07 ,.07)	-.02 (-.08 ,.02)	.00 (-.03 ,.04)	-.02 (-.09, .09)	.00 (-.08, .05)	-.01 (-.06, .05)	.01 (-.09,.11)	-.05 (-.12,.03)	.00 (-.04,.05)	.00 (-.10,.10)	-.02 (-.10,.07)	-.01 (-.04,.03)

Quality of relationship with twin	-.07 (-.13 ,.00)	.09 (.03 ,.15)	-.02 (-.04 ,.01)	-.12 (-.23,-.01)	.14 (.05, .23)	-.02 (-.05, .02)	-.03 (-.12,.06)	.06 (-.02,.13)	-.02 (-.05,.01)	-.05 (-.14,.03)	.07 (.00,.15)	-.02 (-.05,.01)
Quality of relationship with mother	-.02 (-.09 ,.03)	.01 (-.03 ,.06)	.03 (.00 ,.07)	.00 (-.09, .06)	.00 (-.04, .07)	.01 (-.04, .07)	-.04 (-.14,.05)	.02 (-.05,.09)	.05 (.01,.09)	-.05 (-.14,.05)	.02 (-.06,.10)	.04 (.00,.07)
Quality of relationship with father	-.03 (-.11 ,.06)	.05 (-.02 ,.12)	.01 (-.02 ,.04)	-.08 (-.21, .05)	.11 (.00, .20)	.02 (-.03, .08)	.01 (-.10,.12)	.01 (-.07,.10)	.01 (-.03,.05)	-.02 (-.12,.09)	.04 (-.05,.13)	.02 (-.02,.05)
Number of relationships	-.08 (-.11,-.04)	.00 (-.03 ,.02)	.01 (-.02 ,.04)	-.06 (-.12, .03)	.00 (-.07, .03)	-.01 (-.06, .04)	-.08 (-.14,-.01)	.00 (-.05,.04)	.02 (-.02,.06)	-.05 (-.14,.03)	-.04 (-.11,.03)	.00 (-.04,.03)
Longest relationship	-.05 (-.09,-.01)	.00 (-.04 ,.02)	-.01 (-.05 ,.03)	-.04 (-.12, .05)	.00 (-.06, .05)	.03 (-.03, .10)	-.06 (-.11,.02)	.00 (-.06,.03)	-.03 (-.08,.02)	-.05 (-.12,.03)	.00 (-.06,.05)	-.02 (-.06,.02)
Partner violence	-.04 (-.11 ,.04)	-.03 (-.09 ,.02)	-.02 (-.06 ,.02)	-.13 (-.24, .00)	-.01 (-.10, .07)	.05 (-.02, .11)	.00 (-.09,.09)	-.04 (-.12,.02)	-.05 (-.10,.00)	-.03 (-.12,.06)	-.04 (-.12,.02)	-.03 (-.07,.01)
Contact with mother	-.15 (-.25,-.03)	-.03 (-.11 ,.04)	-.06 (-.12 ,.00)	-.14 (-.32, .05)	.03 (-.10, .15)	-.08 (-.18, .01)	-.15 (-.28,.00)	-.06 (-.17,.03)	-.05 (-.12,.02)	-.11 (-.27,.03)	-.08 (-.20,.04)	-.07 (-.13,-.02)
Communication with mother	-.05 (-.18 ,.07)	.04 (-.06 ,.14)	-.01 (-.06 ,.04)	.00 (-.20, .21)	.04 (-.12, .19)	-.03 (-.12, .06)	-.08 (-.23,.08)	.04 (-.09,.16)	.00 (-.06,.06)	-.09 (-.23,.08)	.05 (-.09,.17)	-.02 (-.07,.04)
Contact with father	-.07 (-.21 ,.06)	-.07 (-.17 ,.03)	-.02 (-.08 ,.03)	-.08 (-.26, .14)	.01 (-.16, .14)	-.07 (-.16, .02)	-.07 (-.24,.08)	-.11 (-.22,.01)	.01 (-.07,.08)	-.06 (-.22,.10)	-.11 (-.23,.03)	-.03 (-.09,.03)
Communication with father	.11 (.00 ,.22)	-.04 (-.13 ,.05)	-.02 (-.07 ,.02)	-.02 (-.21, .17)	.07 (-.08, .20)	-.03 (-.10, .05)	.17 (.07,.24)	-.10 (-.17,-.01)	-.02 (-.06,.02)	.19 (.09,.26)	-.13 (-.20,-.04)	-.03 (-.06,.01)
Peer pressure	.01 (-.05 ,.06)	.00 (-.03 ,.04)	-.02 (-.05 ,.02)	-.04 (-.10, .05)	.00 (-.06, .06)	.01 (-.05, .06)	.04 (-.04,.11)	.00 (-.04,.06)	-.04 (-.08,.01)	.01 (-.08,.08)	.01 (-.04,.08)	-.02 (-.05,.02)
Physical peer victimisation	-.01 (-.05 ,.06)	.00 (-.05 ,.02)	-.03 (-.07 ,.02)	-.03 (-.10, .11)	.00 (-.11, .04)	.00 (-.07, .06)	.01 (-.04,.11)	.00 (-.07,.01)	-.05 (-.11,.01)	.02 (-.06,.13)	-.02 (-.11,.03)	-.04 (-.08,.01)
Social peer victimisation	.01 (-.07 ,.09)	-.03 (-.10 ,.01)	-.01 (-.05 ,.03)	.05 (-.10, .15)	-.07 (-.15, .03)	-.01 (-.08, .06)	-.03 (-.08,.08)	.00 (-.08,.03)	-.01 (-.05,.04)	.07 (-.03,.16)	-.08 (-.16,.00)	-.02 (-.06,.03)
Verbal peer victimisation	.01 (-.05 ,.10)	-.02 (-.08 ,.02)	.00 (-.04 ,.04)	.05 (-.08, .17)	-.07 (-.16, .03)	-.01 (-.08, .06)	.00 (-.05,.10)	.00 (-.07,.02)	.00 (-.05,.05)	.06 (-.03,.16)	-.06 (-.12,.02)	-.01 (-.05,.03)
Cyber peer victimisation	-.06 (-.11 ,.03)	-.01 (-.07 ,.02)	.01 (-.03 ,.05)	.09 (-.06, .17)	-.13 (-.19,-.03)	-.01 (-.07, .06)	-.07 (-.12,.00)	.00 (-.04,.02)	.00 (-.05,.05)	-.03 (-.10,.07)	-.02 (-.10,.02)	.00 (-.04,.04)
Physical peer perpetration	-.03 (-.08 ,.02)	.00 (-.04 ,.02)	-.05 (-.09 ,.00)	-.03 (-.12, .11)	-.01 (-.12, .05)	-.09 (-.16,-.02)	-.04 (-.12,.02)	.00 (-.04,.04)	-.01 (-.07,.06)	.04 (-.07,.12)	-.08 (-.15,.02)	-.04 (-.09,.01)
Social peer perpetration	-.02 (-.09 ,.06)	-.02 (-.09 ,.02)	.00 (-.05 ,.04)	.00 (-.10, .14)	-.03 (-.13, .03)	-.03 (-.11, .05)	.01 (-.14,.10)	-.05 (-.12,.05)	.00 (-.06,.08)	.04 (-.02,.13)	-.07 (-.14,-.02)	-.01 (-.06,.03)
Verbal peer perpetration	-.04 (-.08 ,.02)	.00 (-.04 ,.02)	.02 (-.01 ,.06)	-.03 (-.09, .10)	.00 (-.10, .04)	.01 (-.05, .07)	-.05 (-.12,.02)	.00 (-.04,.04)	.04 (-.01,.09)	-.04 (-.08,.05)	.00 (-.07,.05)	.01 (-.03,.05)
Cyber peer perpetration	-.07 (-.11,-.01)	.00 (-.04 ,.02)	.00 (-.05 ,.04)	-.03 (-.11, .12)	-.01 (-.11, .03)	-.02 (-.09, .06)	-.09 (-.16,-.04)	.00 (-.04,.04)	.00 (-.05,.06)	-.05 (-.11,.04)	.00 (-.08,.04)	.00 (-.05,.04)
Parental role aspirations	.03 (-.03 ,.07)	.00 (-.03 ,.04)	-.05 (-.08,-.01)	.05 (-.01, .13)	.00 (-.05, .06)	-.09 (-.15,-.03)	.00 (-.09,.10)	.01 (-.06,.07)	-.02 (-.07,.03)	.02 (-.06,.06)	.00 (-.05,.06)	-.05 (-.09,-.01)
Occupational role aspirations	.09 (.04 ,.13)	.00 (-.04 ,.03)	.02 (-.02 ,.06)	.08 (-.01, .20)	.00 (-.09, .05)	.04 (-.03, .11)	.10 (.01,.18)	.00 (-.05,.06)	.01 (-.03,.06)	.09 (-.02,.17)	.02 (-.05,.10)	.01 (-.03,.05)
Homecare role aspirations	-.06 (-.13,-.02)	.00 (-.02 ,.05)	.02 (-.02 ,.05)	-.01 (-.11, .06)	.00 (-.05, .06)	.00 (-.05, .06)	-.10 (-.19,-.03)	.01 (-.03,.08)	.03 (-.02,.07)	-.10 (-.20,.00)	.03 (-.06,.10)	.02 (-.02,.06)

Importance of relationships	.03 (-.01 ,.07)	.00 (-.01 ,.03)	-.02 (-.06 ,.01)	.04 (-.01, .12)	.00 (-.05, .03)	-.02 (-.07, .03)	.02 (-.07,.07)	.00 (-.02,.07)	-.03 (-.07,.02)	.01 (-.07,.06)	.01 (-.02,.07)	-.03 (-.07,.00)
Achievement motivation	.02 (-.05 ,.06)	.00 (-.03 ,.05)	.03 (.00 ,.07)	.08 (-.03, .20)	.00 (-.09, .08)	-.02 (-.08, .04)	-.02 (-.11,.03)	.00 (-.03,.06)	.07 (.02,.11)	-.03 (-.12,.06)	.04 (-.03,.12)	.03 (-.01,.07)
Purpose in life	.01 (-.06 ,.08)	.03 (-.02 ,.09)	.03 (-.01 ,.06)	.03 (-.10, .14)	.04 (-.04, .14)	.04 (-.02, .10)	.01 (-.09,.08)	.02 (-.03,.09)	.02 (-.02,.07)	-.03 (-.12,.06)	.06 (-.02,.13)	.03 (-.01,.07)
Importance of democracy and equality	.13 (.09 ,.18)	.00 (-.04 ,.02)	.01 (-.02 ,.04)	.07 (-.04, .18)	.00 (-.08, .08)	.03 (-.02, .09)	.17 (.12,.22)	.00 (-.05,.03)	-.01 (-.05,.04)	.14 (.10,.20)	.00 (-.05,.04)	.00 (-.03,.04)
Environmental concerns	.19 (.13 ,.24)	.00 (-.04 ,.04)	.00 (-.03 ,.03)	.18 (.09, .23)	.00 (-.05, .07)	.01 (-.04, .06)	.20 (.11,.28)	-.01 (-.06,.06)	-.01 (-.05,.03)	.17 (.08,.26)	.03 (-.04,.10)	.00 (-.04,.03)
Religiosity	.03 (-.04 ,.11)	.04 (-.02 ,.10)	-.01 (-.04 ,.02)	.06 (-.06, .18)	.01 (-.09, .11)	-.04 (-.08, .01)	.02 (-.08,.12)	.06 (-.02,.14)	.00 (-.03,.04)	.01 (-.08,.10)	.08 (-.01,.15)	-.01 (-.04,.02)
Importance of leisure	.10 (.02 ,.17)	.02 (-.02 ,.08)	-.01 (-.04 ,.03)	.08 (-.06, .20)	.05 (-.03, .16)	-.02 (-.08, .05)	.12 (.01,.18)	.00 (-.03,.08)	.00 (-.05,.05)	.05 (-.04,.15)	.07 (-.01,.15)	.00 (-.04,.04)
Alcohol use	.08 (.00 ,.17)	-.02 (-.08 ,.03)	-.03 (-.07 ,.01)	.08 (-.09, .24)	-.01 (-.13, .11)	-.02 (-.10, .06)	.06 (-.02,.17)	-.02 (-.09,.03)	-.03 (-.08,.02)	.11 (-.01,.21)	-.05 (-.13,.05)	-.04 (-.08,.01)
Ever smoked	-.05 (-.09 ,.01)	.00 (-.04 ,.03)	.00 (-.04 ,.03)	-.03 (-.10, .08)	.00 (-.08, .04)	-.02 (-.08, .04)	-.06 (-.13,.03)	.00 (-.07,.05)	.01 (-.04,.05)	-.05 (-.11,.04)	.00 (-.08,.04)	.00 (-.04,.03)
Ever vaped	-.11 (-.18,-.03)	-.01 (-.06 ,.05)	.00 (-.04 ,.04)	-.08 (-.19, .06)	-.02 (-.11, .06)	-.01 (-.08, .05)	-.13 (-.23,-.02)	.00 (-.07,.08)	.01 (-.04,.05)	-.08 (-.19,.02)	-.03 (-.11,.06)	.00 (-.04,.04)
Cognitive enhancers	.02 (-.05 ,.09)	.00 (-.04 ,.06)	-.01 (-.05 ,.03)	.04 (-.07, .16)	.00 (-.09, .07)	-.01 (-.07, .05)	-.01 (-.11,.10)	.03 (-.05,.10)	-.01 (-.06,.04)	-.03 (-.13,.09)	.05 (-.04,.13)	-.01 (-.05,.03)
Big 5 Agreeableness	.02 (-.02 ,.06)	.00 (-.03 ,.02)	-.02 (-.06 ,.02)	.02 (-.05, .12)	.00 (-.06, .05)	-.03 (-.09, .04)	.02 (-.03,.07)	.00 (-.04,.02)	-.02 (-.07,.03)	.03 (-.02,.09)	.00 (-.04,.03)	-.04 (-.08,.00)
Big 5 Conscientiousness	.02 (-.02 ,.06)	.00 (-.02 ,.02)	.06 (.03 ,.10)	.01 (-.05, .09)	.00 (-.05, .04)	.06 (-.01, .12)	.03 (-.02,.08)	.00 (-.02,.03)	.07 (.02,.12)	.03 (-.04,.07)	.00 (-.03,.05)	.06 (.02,.10)
Big 5 Extraversion	.06 (.03 ,.10)	.00 (-.01 ,.04)	-.03 (-.06 ,.01)	.05 (-.05, .11)	.00 (-.03, .07)	-.03 (-.09, .03)	.07 (.03,.12)	.00 (-.02,.06)	-.02 (-.07,.02)	.05 (-.04,.09)	.01 (-.02,.07)	-.02 (-.06,.02)
Big 5 Neuroticism	-.03 (-.10 ,.03)	.00 (-.04 ,.04)	-.01 (-.05 ,.03)	-.09 (-.23, .05)	.00 (-.09, .10)	.03 (-.04, .10)	-.01 (-.08,.04)	.00 (-.05,.05)	-.03 (-.07,.02)	-.05 (-.15,.04)	.03 (-.05,.10)	.00 (-.05,.04)
Big 5 Openness	.04 (.00 ,.08)	.00 (-.01 ,.03)	.00 (-.04 ,.04)	.03 (-.08, .09)	.00 (-.03, .08)	.00 (-.05, .06)	.04 (-.02,.09)	.00 (-.02,.04)	.00 (-.05,.05)	.03 (-.06,.08)	.01 (-.02,.08)	.00 (-.04,.04)
Self-control	.04 (.00 ,.09)	.00 (-.03 ,.02)	.02 (-.02 ,.05)	.04 (-.04, .13)	.00 (-.05, .05)	.01 (-.05, .07)	.04 (-.01,.09)	.00 (-.05,.03)	.02 (-.03,.06)	.04 (-.01,.13)	-.01 (-.07,.03)	.02 (-.02,.05)
Not planning for the future	-.09 (-.16,-.02)	.00 (-.05 ,.05)	-.02 (-.06 ,.02)	-.09 (-.15, .00)	.00 (-.06, .06)	-.01 (-.07, .05)	-.09 (-.20,.02)	.00 (-.03,.08)	-.03 (-.09,.02)	-.10 (-.18,-.01)	.00 (-.07,.06)	-.03 (-.07,.01)
Ambition	.10 (.04 ,.14)	.00 (-.03 ,.04)	.03 (-.01 ,.07)	.13 (.05, .25)	.00 (-.09, .04)	-.01 (-.08, .05)	.08 (-.02,.13)	.00 (-.03,.07)	.05 (.01,.10)	.10 (.01,.14)	.00 (-.04,.07)	.02 (-.02,.06)
Physical activity	.09 (.02 ,.12)	.00 (-.02 ,.05)	-.01 (-.04 ,.03)	.10 (.02, .20)	.00 (-.08, .05)	-.05 (-.10, .01)	.05 (-.05,.12)	.03 (-.02,.10)	.02 (-.02,.07)	.04 (-.05,.13)	.05 (-.02,.13)	.00 (-.04,.04)
Health behaviours	.14 (.07 ,.20)	.02 (-.02 ,.08)	-.02 (-.05 ,.01)	.18 (.08, .24)	.00 (-.05, .08)	-.03 (-.08, .02)	.11 (.02,.20)	.04 (-.03,.11)	-.01 (-.05,.03)	.15 (.06,.24)	.02 (-.05,.10)	-.01 (-.04,.02)
Risk taking behaviour	.06 (.00 ,.10)	.00 (-.03 ,.04)	-.05 (-.09,-.02)	.05 (-.07, .17)	-.02 (-.11, .07)	-.06 (-.11, .00)	.08 (-.01,.12)	.00 (-.03,.06)	-.06 (-.10,-.01)	.05 (-.04,.11)	.01 (-.04,.08)	-.06 (-.09,-.02)

Risky sexual behaviour	-.05 (-.09 ,.01)	.00 (-.05 ,.03)	-.01 (-.04 ,.02)	-.05 (-.10, .06)	.00 (-.08, .05)	-.03 (-.08, .02)	-.06 (-.13,.03)	.00 (-.06,.05)	.00 (-.04,.03)	-.02 (-.11,.07)	-.04 (-.11,.04)	-.02 (-.05,.01)
Media use	-.02 (-.06 ,.02)	.00 (-.02 ,.02)	-.01 (-.04 ,.02)	.01 (-.04, .07)	.00 (-.05, .03)	-.01 (-.06, .04)	-.04 (-.09,.00)	.00 (-.03,.04)	-.01 (-.05,.03)	-.03 (-.07,.03)	.00 (-.05,.02)	-.01 (-.05,.02)
Volunteering	.11 (.06 ,.17)	.00 (-.05 ,.03)	.00 (-.03 ,.03)	.11 (.05, .23)	.00 (-.08, .05)	-.03 (-.09, .02)	.11 (.04,.19)	.00 (-.06,.04)	.01 (-.03,.06)	.13 (.05,.22)	-.02 (-.08,.05)	.00 (-.04,.03)
Internet dating	.01 (-.06 ,.07)	.00 (-.04 ,.05)	.01 (-.02 ,.05)	.00 (-.11, .10)	.00 (-.07, .08)	-.02 (-.07, .04)	.02 (-.07,.07)	.00 (-.05,.06)	.03 (-.02,.07)	.00 (-.09,.10)	.00 (-.08,.08)	.01 (-.03,.04)
Internet dating	.00 (-.05 ,.06)	.00 (-.04 ,.04)	.01 (-.02 ,.05)	.00 (-.09, .12)	.00 (-.08, .06)	-.02 (-.07, .04)	.01 (-.07,.07)	.00 (-.04,.05)	.03 (-.02,.07)	.01 (-.09,.11)	-.01 (-.09,.07)	.00 (-.04,.04)
Financial literacy: knowledge of products	.00 (-.04 ,.04)	.00 (-.02 ,.04)	.02 (-.02 ,.05)	.02 (-.10, .11)	.01 (-.04, .11)	.00 (-.05, .06)	-.03 (-.10,.03)	.00 (-.04,.04)	.04 (-.01,.08)	-.01 (-.10,.08)	.00 (-.06,.07)	.03 (-.01,.06)
Financial attitudes and behaviour	.03 (-.01 ,.07)	.00 (-.02 ,.03)	.03 (.00 ,.07)	.04 (-.06, .12)	.00 (-.04, .07)	.02 (-.04, .08)	.02 (-.04,.07)	.00 (-.03,.04)	.04 (-.01,.09)	.03 (-.04,.08)	.00 (-.04,.05)	.04 (.00,.08)

Note. A= genetic influences, C= shared environmental influences, E= unique environmental influences. Scores were corrected for mean age and sex differences (see Methods).

Table S13. Model fitting results for bivariate analyses of genetic correlation (rA), shared environmental correlation (rC), and non-shared environmental correlation (rE) for psychological traits and functional outcomes (95% confidence intervals are in parentheses).

	Model fitting results											
	Functional outcome: wellbeing											
	Whole sample			Males only			Females only			Same-sex MZ and DZ twins		
Trait	rA	rC	rE	rA	rC	rE	rA	rC	rE	rA	rC	rE
Daily hassles	- .49 (- .71,-.49)	-1.00 (-1.00, 1.00)	- .18 (-.22,- .13)	-.52 (-.86,-.22)	-.96 (-1.00, 1.00)	-.24 (-.31,-.17)	-.44 (-.76,-.12)	-1.00 (-1.00,1.00)	-.15 (-.21,-.09)	-.41 (-.73,-.15)	-1.00 (-1.00,-.99)	-.17 (-.22,-.13)
CHAOS	- .33 (- .77,-.33)	- .75 (-1.00,-.35)	- .25 (-.29,- .20)	-.20 (-.20,.33)	-1.00 (-1.00, 1.00)	-.28 (-.35,-.20)	-.68 (-1.00,1.00)	-.57 (-1.00,-.26)	-.23 (-.29,-.17)	-.24 (-1.00, 1.00)	-.59 (-.98,-.59)	-.25 (-.30,-.20)
Childhood experiences	- .36 (- .54,-.35)	- .28 (-1.00, 1.00)	- .15 (-.21,- .10)	-.19 (-.23,.12)	-1.00 (-1.00, 1.00)	-.25 (-.34,-.17)	-.46 (-.73,-.46)	-.24 (-.66,.06)	-.10 (-.17,-.04)	-.45 (-.76,-.45)	-.20 (-.53,.06)	-.15 (-.20,-.10)
Poor sleep quality	- .45 (- .75,-.17)	- .34 (-1.00, 1.00)	- .14 (-.19,- .09)	-.27 (-.70,-.27)	-1.00 (-1.00, 1.00)	-.22 (-.29,-.14)	-.57 (-1.00,-.47)	-.26 (-1.00,1.00)	-.09 (-.15,-.03)	-.41 (-1.00,.16)	-.50 (-1.00,.06)	-.15 (-.20,-.10)
Marriage hopes	.18 (.18,.45)	.55 (- .31, 1.00)	.09 (.04,.14)	1.00 (-1.00, 1.00)	1.00 (-1.00, 1.00)	.06 (-.02,.14)	.19 (.07,.54)	.28 (-1.00,1.00)	.10 (.04,.16)	.34 (-.22, 1.00)	.16 (.16,.80)	.09 (.04,.14)
Marriage worries	- .34 (- .50,-.15)	-1.00 (-1.00, 1.00)	- .20 (-.25,- .16)	-.40 (-.63,-.09)	-1.00 (-1.00, 1.00)	-.15 (-.23,-.08)	-.30 (-.60,-.30)	-.90 (-1.00,-.11)	-.24 (-.29,-.18)	-.20 (-.48,-.05)	-1.00 (-1.00,-.40)	-.21 (-.25,-.16)
Quality of relationship with twin	.31 (.11,.52)	.27 (.04, 1.00)	.09 (.04,.14)	.11 (-.18,.44)	1.00 (-1.00, 1.00)	.14 (.05,.22)	.45 (.45,.75)	.16 (-.09,.43)	.07 (.00,.13)	.33 (.00,.70)	.30 (.12,.53)	.10 (.05,.15)
Quality of relationship with mother	.34 (.18,.52)	1.00 (- .87, 1.00)	.12 (.07,.17)	.40 (.24,.73)	-1.00 (-1.00, 1.00)	.07 (-.01,.14)	.30 (.12,.55)	.41 (-1.00,1.00)	.15 (.09,.21)	.44 (.44,.75)	.14 (-1.00, 1.00)	.12 (.07,.17)

Quality of relationship with father	.40 (.21,.61)	.23 (-1.00,1.00)	.14 (.09,.19)	.39 (.39,.67)	1.00 (-1.00,1.00)	.18 (.09,.26)	.41 (.16,.71)	.19 (-.21,.58)	.12 (.06,.18)	.40 (.23,.72)	.25 (.22,.58)	.14 (.09,.19)
Number of relationships	.12 (-.03,.29)	-1.00 (-1.00,1.00)	.05 (.00,.09)	.07 (-.07,.27)	.76 (-1.00,1.00)	.10 (.03,.17)	.20 (-.04,.48)	-1.00 (-1.00,1.00)	.01 (-.05,.07)	.13 (.12,.45)	-.04 (-1.00,1.00)	.04 (-.01,.09)
Longest relationship	.39 (.23,.67)	-1.00 (-1.00,.81)	.14 (.09,.19)	.18 (.18,.72)	-1.00 (-1.00,1.00)	.17 (.09,.25)	.51 (.22,.92)	-1.00 (-1.00,1.00)	.12 (.06,.18)	.42 (.09,.86)	-1.00 (-1.00,1.00)	.12 (.06,.17)
Partner violence	- .36 (- .64,-.36)	-1.00 (-1.00,1.00)	- .15 (-.19,- .10)	-.24 (-.59,-.24)	-1.00 (-1.00,1.00)	-.18 (-.25,-.10)	-.41 (-.74,-.38)	-1.00 (-1.00,1.00)	-.14 (-.19,-.08)	-.51 (-.74,-.41)	-.01 (-1.00,1.00)	-.15 (-.20,-.11)
Contact with mother	.30 (.08,.63)	- .28 (-1.00,1.00)	.03 (-.05,.12)	.37 (.16,1.00)	-1.00 (-1.00,1.00)	.08 (-.07,.22)	.24 (-.15,.68)	-.06 (-1.00,1.00)	.02 (-.08,.11)	.30 (-.20,.83)	.03 (-1.00,1.00)	.03 (-.05,.12)
Communication with mother	.40 (.08,.78)	- .08 (-1.00,.03)	- .03 (-.11,.05)	.45 (.45,1.00)	-1.00 (-1.00,1.00)	-.04 (-.19,.11)	.41 (.41,.94)	-.02 (-.82,-.01)	-.03 (-.12,.07)	.45 (-.01,1.00)	-.01 (-.99,.66)	-.03 (-.11,.05)
Contact with father	.35 (-1.00,1.00)	.15 (-1.00,1.00)	.00 (-.08,.08)	-.15 (-1.00,1.00)	1.00 (-1.00,1.00)	.06 (-.06,.19)	.53 (.53,1.00)	-.02 (-.61,.12)	-.04 (-.13,.06)	.55 (-1.00,1.00)	.11 (-.32,.53)	.01 (-.07,.08)
Communication with father	.29 (.29,.88)	.25 (- .10,1.00)	.05 (-.03,.12)	.13 (-1.00,1.00)	1.00 (-1.00,1.00)	.11 (-.02,.24)	.32 (-1.00,1.00)	.24 (.09,.65)	.01 (-.08,.10)	.20 (-1.00,1.00)	.26 (-.01,.58)	.05 (-.03,.12)
Peer pressure	- .42 (- .66,-.22)	1.00 (-1.00,1.00)	- .16 (-.20,- .11)	-.34 (-.74,-.08)	1.00 (-1.00,1.00)	-.15 (-.23,-.08)	-.46 (-.82,-.22)	1.00 (-1.00,1.00)	-.16 (-.22,-.10)	-.49 (-.86,-.17)	1.00 (-1.00,1.00)	-.16 (-.21,-.11)
Physical peer victimisation	.08 (-.15,.51)	-1.00 (-1.00,1.00)	- .13 (-.18,- .08)	.17 (-.19,1.00)	-1.00 (-1.00,1.00)	-.15 (-.22,-.07)	-.09 (-.95,1.00)	-1.00 (-1.00,-.65)	-.11 (-.17,-.04)	.23 (-.23,1.00)	-1.00 (-1.00,1.00)	-.14 (-.19,-.09)
Social peer victimisation	- .17 (-.43,.14)	-1.00 (-1.00,1.00)	- .17 (-.22,- .12)	.07 (-1.00,1.00)	-1.00 (-1.00,-.25)	-.21 (-.29,-.12)	-.21 (-.51,.15)	-1.00 (-1.00,1.00)	-.15 (-.21,-.09)	.22 (-.24,1.00)	-1.00 (-1.00,-.34)	-.17 (-.22,-.11)
Verbal peer victimisation	- .31 (- .57,-.31)	1.00 (-1.00,1.00)	- .17 (-.22,- .12)	-.15 (-1.00,1.00)	1.00 (-1.00,1.00)	-.20 (-.28,-.12)	-.41 (-.76,-.19)	1.00 (-1.00,1.00)	-.15 (-.21,-.09)	-.32 (-.74,.10)	-.53 (-1.00,1.00)	-.17 (-.22,-.12)
Cyber peer victimisation	- .26 (- .47,-.06)	- .13 (-1.00,1.00)	- .14 (-.19,- .09)	-.08 (-1.00,1.00)	-1.00 (-1.00,1.00)	-.18 (-.27,-.09)	-.32 (-.60,-.08)	-1.00 (-1.00,1.00)	-.12 (-.18,-.05)	-.21 (-.53,.13)	-1.00 (-1.00,1.00)	-.13 (-.18,-.08)
Physical peer perpetration	.17 (.17,.84)	-1.00 (-1.00,1.00)	- .09 (-.14,- .04)	.24 (-1.00,1.00)	-1.00 (-1.00,1.00)	-.12 (-.20,-.04)	-.06 (-1.00,1.00)	-1.00 (-1.00,1.00)	-.04 (-.12,.04)	.28 (-1.00,1.00)	-.46 (-1.00,1.00)	-.09 (-.15,-.04)
Social peer perpetration	- .30 (- .66,-.30)	-1.00 (-1.00,1.00)	- .13 (-.18,- .08)	-.20 (-1.00,-.06)	.03 (-1.00,1.00)	-.19 (-.27,-.11)	-.14 (-.68,1.00)	-1.00 (-1.00,1.00)	-.11 (-.17,-.05)	-.12 (-1.00,1.00)	-.78 (-1.00,1.00)	-.13 (-.19,-.08)
Verbal peer perpetration	- .12 (-.32,.09)	-1.00 (-1.00,1.00)	- .11 (-.16,- .06)	.01 (-.21,.33)	1.00 (-1.00,1.00)	-.18 (-.25,-.10)	-.17 (-.49,.18)	-1.00 (-1.00,1.00)	-.06 (-.13,.00)	-.02 (-.20,.39)	-1.00 (-1.00,1.00)	-.11 (-.16,-.05)
Cyber peer perpetration	- .18 (-.46,.10)	-1.00 (-1.00,1.00)	- .10 (-.15,- .05)	-.07 (-.49,.66)	.06 (-1.00,1.00)	-.14 (-.22,-.06)	-.28 (-.73,.11)	-.41 (-1.00,.96)	-.07 (-.13,.00)	-.01 (-.47,.63)	-1.00 (-1.00,1.00)	-.10 (-.15,-.04)
Parental role aspirations	.37 (.37,.62)	-1.00 (-1.00,1.00)	.07 (.02,.12)	.32 (.16,.79)	-1.00 (-1.00,1.00)	.11 (.03,.18)	.41 (.41,.78)	.01 (-1.00,1.00)	.05 (-.01,.11)	.44 (.12,.82)	.07 (-1.00,1.00)	.07 (.02,.12)
Occupational role aspirations	.08 (.08,.37)	-1.00 (NA,1.00)	.00 (-.05,.05)	.15 (-.25,1.00)	-1.00 (-1.00,1.00)	.01 (-.06,.09)	-.03 (-.41,.34)	1.00 (-1.00,1.00)	.00 (-.06,.06)	.20 (-.22,.69)	-1.00 (-1.00,1.00)	.01 (-.04,.06)
Homecare role aspirations	.31 (.13,.50)	1.00 (-1.00,1.00)	.11 (.06,.16)	.36 (.36,.74)	-1.00 (-1.00,1.00)	.12 (.04,.20)	.21 (-.08,.50)	1.00 (-1.00,1.00)	.11 (.05,.17)	.22 (.22,.58)	.62 (-1.00,1.00)	.12 (.07,.17)
Importance of relationships	.27 (.12,.41)	1.00 (-1.00,1.00)	.10 (.06,.15)	.18 (-.18,.23)	1.00 (-1.00,1.00)	.11 (.04,.19)	.40 (.31,.68)	1.00 (-.48,1.00)	.09 (.03,.15)	.28 (.16,.54)	1.00 (-1.00,1.00)	.11 (.06,.16)
Achievement motivation	.27 (.06,.50)	- .99 (-1.00,1.00)	.03 (-.01,.08)	.27 (.10,.76)	-.99 (-1.00,1.00)	.05 (-.03,.13)	.29 (.16,.61)	-1.00 (-1.00,1.00)	.03 (-.03,.08)	.29 (.27,.69)	.60 (-1.00,1.00)	.03 (-.02,.08)

Purpose in life	.64 (.48,.80)	.53 (-1.00, 1.00)	.32 (.28,.37)	.53 (.53,.77)	1.00 (-1.00, 1.00)	.37 (.30,.44)	.72 (.51,.97)	1.00 (.59,1.00)	.30 (.24,.35)	.75 (.75, 1.00)	.33 (-1.00, 1.00)	.32 (.28,.37)
Importance of democracy and equality	- .03 (-.19,.14)	- .16 (-1.00, 1.00)	.00 (-.05,.05)	-.06 (-.42,.01)	1.00 (-1.00, 1.00)	-.01 (-.08,.07)	-.02 (-.25,.22)	-1.00 (-1.00,1.00)	.00 (-.06,.06)	.08 (.01,.38)	-1.00 (-1.00, 1.00)	.01 (-.04,.06)
Environmental concerns	- .01 (-.19,.16)	-1.00 (-1.00, 1.00)	.02 (-.02,.07)	-.06 (-.37,-.02)	1.00 (-1.00, 1.00)	.03 (-.05,.11)	.11 (-.13,.37)	-.99 (-1.00,1.00)	.01 (-.05,.07)	.05 (-.24,.35)	-.47 (-1.00, 1.00)	.02 (-.03,.07)
Religiosity	.01 (-.21,.22)	.04 (-1.00, 1.00)	.01 (-.04,.06)	.33 (.05,.78)	-1.00 (-1.00, 1.00)	-.03 (-.12,.05)	-.19 (-.50,-.17)	.19 (-.09,.62)	.04 (-.02,.10)	-.06 (-.41,.03)	.06 (-.16,.31)	.01 (-.04,.06)
Importance of leisure	.46 (.30,.62)	1.00 (-1.00, 1.00)	.18 (.13,.23)	.37 (-.08,.37)	1.00 (-1.00, 1.00)	.22 (.14,.30)	.55 (.55,.83)	1.00 (-1.00,1.00)	.16 (.09,.21)	.43 (.43,.71)	1.00 (-1.00, 1.00)	.19 (.14,.24)
Alcohol use	- .22 (- .41,-.03)	1.00 (-1.00, 1.00)	- .15 (-.20,- .09)	-.31 (-.90,-.31)	1.00 (.07, 1.00)	-.20 (-.29,-.11)	-.10 (-.36,.18)	-1.00 (-1.00,1.00)	-.12 (-.19,-.05)	-.30 (-.66,-.15)	.53 (.53, 1.00)	-.14 (-.20,-.09)
Ever smoked	- .18 (- .37,-.01)	1.00 (- .97, 1.00)	- .07 (-.12,- .02)	-.07 (-.34,-.07)	1.00 (-1.00, 1.00)	-.09 (-.17,-.01)	-.28 (-.56,-.03)	1.00 (-1.00,1.00)	-.06 (-.12,.00)	-.20 (-.50,.07)	1.00 (-1.00, 1.00)	-.07 (-.12,-.01)
Ever vaped	- .18 (-.45,.09)	.03 (-1.00, 1.00)	- .06 (-.12,- .01)	-.02 (-.02,.44)	-1.00 (-1.00, 1.00)	-.09 (-.17,.00)	-.34 (-.76,.03)	.22 (-1.00,1.00)	-.05 (-.11,.01)	-.37 (-.86,-.10)	.34 (-1.00, 1.00)	-.06 (-.11,-.01)
Cognitive enhancers	- .26 (- .52,-.02)	- .68 (-1.00, 1.00)	- .10 (-.15,- .04)	.04 (-.22,.70)	-1.00 (-1.00, 1.00)	-.17 (-.25,-.08)	-.56 (-1.00,-.22)	.24 (-1.00,1.00)	-.05 (-.11,.01)	-.36 (-.83,.06)	-.06 (-1.00, 1.00)	-.10 (-.15,-.05)
Big 5 Agreeableness	.18 (-.03,.40)	.61 (-1.00, 1.00)	.07 (.03,.12)	.03 (-.32,.30)	.80 (-1.00, 1.00)	.11 (.03,.18)	.29 (-.04,.61)	1.00 (-1.00,1.00)	.06 (.00,.11)	.27 (-.05,.67)	-1.00 (-1.00, 1.00)	.08 (.03,.13)
Big 5 Conscientiousness	.50 (.33,.66)	.04 (-1.00, 1.00)	.17 (.13,.22)	.38 (.17,.70)	-.33 (-1.00, 1.00)	.21 (.14,.28)	.56 (.56,.86)	1.00 (-1.00,1.00)	.15 (.09,.21)	.62 (.48, 1.00)	1.00 (-1.00, 1.00)	.18 (.13,.23)
Big 5 Extraversion	.47 (.34,.61)	1.00 (-1.00, NA)	.15 (.10,.19)	.35 (.07,.54)	1.00 (-1.00, 1.00)	.19 (.11,.26)	.60 (.41,.76)	1.00 (-1.00,1.00)	.12 (.06,.18)	.47 (.46,.73)	1.00 (-1.00, 1.00)	.15 (.10,.20)
Big 5 Neuroticism	- .50 (- .66,-.50)	-1.00 (-1.00, 1.00)	- .26 (-.30,- .21)	-.39 (-.64,-.39)	-1.00 (-1.00, 1.00)	-.32 (-.39,-.25)	-.56 (-.77,-.56)	-1.00 (-1.00,-.87)	-.22 (-.28,-.17)	-.50 (-.79,-.31)	-1.00 (-1.00, 1.00)	-.27 (-.31,-.22)
Big 5 Openness	- .32 (- .50,-.17)	- .94 (-1.00, 1.00)	- .04 (-.09,.00)	-.26 (-.61,-.07)	1.00 (-1.00, 1.00)	-.10 (-.17,-.02)	-.33 (-.60,-.12)	-1.00 (-1.00,1.00)	-.01 (-.07,.04)	-.39 (-.74,-.14)	1.00 (-1.00, 1.00)	-.04 (-.09,.01)
Self-control	.41 (.27,.56)	1.00 (-1.00, 1.00)	.22 (.18,.27)	.38 (.07,.57)	1.00 (-1.00, 1.00)	.25 (.18,.32)	.45 (.34,.68)	1.00 (NA,1.00)	.21 (.15,.26)	.41 (.41,.65)	1.00 (-1.00, 1.00)	.23 (.18,.28)
Not planning for the future	- .27 (- .55,-.27)	-1.00 (-1.00, 1.00)	- .13 (-.18,- .08)	-.28 (-.55,.01)	-.61 (-1.00, 1.00)	-.14 (-.21,-.06)	-.23 (-.88,-.23)	-.45 (-1.00,1.00)	-.13 (-.19,-.07)	-.39 (-.83,-.39)	1.00 (-1.00, 1.00)	-.13 (-.17,-.08)
Ambition	.46 (.25,.74)	-1.00 (-1.00, 1.00)	.03 (-.02,.08)	.55 (.19, 1.00)	-1.00 (-1.00, 1.00)	.06 (-.02,.14)	.39 (.39,.76)	-1.00 (-1.00,1.00)	.02 (-.04,.08)	.61 (.29, 1.00)	-1.00 (-1.00, 1.00)	.03 (-.02,.08)
Physical activity	.27 (.10,.48)	- .98 (-1.00, 1.00)	.05 (.01,.10)	.27 (.04,.64)	-1.00 (-1.00, 1.00)	.10 (.02,.17)	.25 (-.01,.55)	1.00 (-1.00,1.00)	.03 (-.03,.09)	.31 (.01,.66)	.06 (-1.00, 1.00)	.05 (.00,.10)
Health behaviours	.11 (-.06,.28)	.71 (-1.00, 1.00)	.08 (.03,.12)	.23 (.16,.54)	-.82 (-1.00, 1.00)	.01 (-.07,.08)	.02 (-.25,.27)	.57 (-.20,1.00)	.11 (.05,.17)	.07 (-.22,.18)	.50 (-1.00, 1.00)	.07 (.02,.12)
Risk taking behaviour	- .18 (- .36,-.01)	1.00 (-1.00, NA)	- .15 (-.20,- .10)	-.29 (-.61,-.29)	1.00 (.08, 1.00)	-.14 (-.22,-.06)	-.11 (-.34,.14)	-1.00 (-1.00,.90)	-.16 (-.22,-.10)	-.20 (-.48,.08)	1.00 (-1.00, 1.00)	-.15 (-.20,-.10)
Risky sexual behaviour	.11 (-.05,.26)	-1.00 (NA, 1.00)	.05 (.00,.10)	.05 (.05,.31)	-1.00 (-1.00, 1.00)	.12 (.03,.19)	.09 (-.13,.32)	.35 (-1.00,1.00)	.01 (-.05,.08)	-.03 (-.31,.23)	.51 (-1.00, 1.00)	.05 (.00,.10)
Media use	.09 (-.04,.23)	1.00 (-1.00, 1.00)	- .09 (-.14,- .04)	.18 (.18,.41)	1.00 (-1.00, 1.00)	-.11 (-.18,-.03)	.11 (-.09,.34)	-1.00 (-1.00,1.00)	-.09 (-.15,-.03)	.13 (.13,.37)	1.00 (-1.00, NA)	-.08 (-.12,-.03)

Volunteering	.05 (-.13,.23)	1.00 (-1.00, 1.00)	.03 (-.02,.07)	.09 (-.14,.38)	1.00 (-1.00, 1.00)	.02 (-.05,.09)	.00 (-.28,.26)	1.00 (-.71,1.00)	.03 (-.03,.09)	.10 (-.20,.43)	-.30 (-1.00, 1.00)	.03 (-.02,.08)
Internet dating	- .29 (- .53,-.08)	1.00 (-1.00, 1.00)	- .20 (-.25,- .16)	-.20 (-.62,.05)	1.00 (-1.00, 1.00)	-.22 (-.29,-.15)	-.31 (-.64,-.15)	1.00 (-1.00,1.00)	-.20 (-.25,-.14)	-.20 (-.56,.21)	.09 (-1.00, 1.00)	-.19 (-.24,-.15)
Internet dating	- .24 (- .48,-.02)	1.00 (-1.00, 1.00)	- .17 (-.22,- .13)	-.09 (-.48,.29)	1.00 (-1.00, 1.00)	-.20 (-.27,-.12)	-.31 (-.66,-.31)	1.00 (.76,1.00)	-.16 (-.22,-.10)	-.11 (-.51,.31)	-.04 (-1.00, 1.00)	-.15 (-.20,-.11)
Financial literacy: knowledge of products	.32 (.16,.51)	1.00 (-1.00, 1.00)	.17 (.12,.21)	.34 (.09,.63)	1.00 (-1.00, 1.00)	.14 (.06,.21)	.33 (.33,.63)	1.00 (-1.00,1.00)	.18 (.12,.24)	.30 (-.03,.63)	1.00 (-1.00, 1.00)	.17 (.12,.22)
Financial attitudes and behaviour	.49 (.31,.69)	- .62 (-1.00, 1.00)	.28 (.24,.32)	.47 (.47,.83)	-1.00 (-1.00, 1.00)	.29 (.22,.35)	.50 (.50,.80)	1.00 (-1.00,1.00)	.27 (.22,.33)	.55 (.25,.89)	1.00 (-1.00, 1.00)	.27 (.22,.31)

	Model fitting results											
	Functional outcome: adverse mental health											
	Whole sample			Males only			Females only			Same-sex MZ and DZ twins		
Trait	rA	rC	rE	rA	rC	rE	rA	rC	rE	rA	rC	rE
Daily hassles	.71 (.62,.82)	.87 (- .99, 1.00)	.43 (.39,.47)	.65 (.65, 1.00)	1.00 (-1.00, 1.00)	.48 (.40,.54)	.75 (.75,.95)	1.00 (-1.00,1.00)	.41 (.36,.46)	.70 (.58,.86)	.99 (-1.00, 1.00)	.44 (.39,.48)
CHAOS	.44 (.12, 1.00)	1.00 (.99, 1.00)	.23 (.18,.28)	.69 (.69, 1.00)	-.11 (-1.00, 1.00)	.20 (.10,.30)	.54 (-1.00,1.00)	1.00 (.75,1.00)	.24 (.17,.30)	.38 (-.05, 1.00)	1.00 (.97, 1.00)	.24 (.19,.30)
Childhood experiences	.47 (.33,.59)	1.00 (-1.00, 1.00)	.31 (.26,.36)	.27 (-.16,.59)	.45 (-1.00, 1.00)	.42 (.33,.50)	.53 (.37,.68)	1.00 (-1.00,1.00)	.27 (.20,.33)	.55 (.37,.73)	.21 (-1.00, 1.00)	.31 (.26,.36)
Poor sleep quality	.74 (.64,.91)	1.00 (-1.00, 1.00)	.38 (.34,.43)	.59 (-.05, 1.00)	1.00 (-.97, 1.00)	.41 (.32,.48)	.77 (.77,.99)	1.00 (-1.00,1.00)	.37 (.32,.43)	.77 (.58, 1.00)	1.00 (.74, 1.00)	.40 (.35,.44)
Marriage hopes	- .34 (- .71,-.34)	1.00 (-1.00, 1.00)	.02 (-.03,.08)	-1.00 (-1.00, 1.00)	-.10 (-1.00, 1.00)	.03 (-.07,.12)	-.27 (-.57,-.26)	1.00 (-1.00,1.00)	.01 (-.05,.08)	-.28 (-1.00,-.28)	-.99 (-1.00,-.10)	.03 (-.03,.09)
Marriage worries	.31 (.29,.44)	1.00 (.46, NA)	.13 (.08,.18)	.29 (-.14,.61)	1.00 (-1.00, 1.00)	.11 (.02,.20)	.31 (.11,.44)	1.00 (-1.00,1.00)	.15 (.09,.22)	.21 (NA,.40)	1.00 (-1.00, 1.00)	.14 (.08,.19)
Quality of relationship with twin	- .32 (- .50,-.15)	-1.00 (-1.00,-1.00)	- .06 (-.12,.01)	-.67 (-1.00,-.58)	.13 (-1.00, 1.00)	.06 (-.05,.17)	-.25 (-.41,-.05)	-1.00 (-1.00,-.99)	-.12 (-.19,-.04)	-.38 (-.62,-.16)	-1.00 (-1.00,-.36)	-.07 (-.13,.00)
Quality of relationship with mother	- .31 (- .44,-.21)	1.00 (-1.00, 1.00)	- .15 (-.20,- .09)	-.49 (-1.00,-.18)	1.00 (-1.00, 1.00)	-.06 (-.16,.04)	-.30 (-.45,-.16)	-1.00 (-1.00,.99)	-.18 (-.24,-.11)	-.34 (-.52,-.16)	-.15 (-1.00, 1.00)	-.15 (-.20,-.09)
Quality of relationship with father	- .43 (- .60,-.27)	-1.00 (-1.00, 1.00)	- .13 (-.18,- .07)	-.29 (-.73,-.29)	-.45 (-1.00,-.40)	-.17 (-.27,-.07)	-.51 (-.72,-.31)	1.00 (-1.00,1.00)	-.10 (-.17,-.04)	-.48 (-.69,-.26)	.45 (-1.00, 1.00)	-.13 (-.18,-.07)
Number of relationships	.18 (.09,.26)	.73 (-1.00, 1.00)	- .01 (-.06,.04)	.18 (.18,.58)	-1.00 (-1.00, 1.00)	-.04 (-.13,.05)	.21 (.06,.30)	.35 (-1.00,1.00)	.01 (-.06,.07)	.17 (.00,.37)	.96 (-1.00, 1.00)	.00 (-.06,.06)
Longest relationship	- .08 (-.20,.03)	- .93 (-1.00, 1.00)	- .01 (-.07,.04)	-.02 (-.57,.62)	-.81 (-1.00, 1.00)	-.07 (-.16,.03)	-.09 (-.23,.03)	.31 (-1.00,1.00)	.01 (-.06,.08)	-.07 (-.22,.13)	-1.00 (-1.00, 1.00)	.00 (-.06,.06)
Partner violence	.62 (.49,.75)	1.00 (-1.00, 1.00)	.16 (.11,.22)	.36 (-.18,.36)	1.00 (-1.00, 1.00)	.22 (.13,.31)	.70 (.56,.90)	1.00 (-1.00,1.00)	.15 (.09,.21)	.59 (.51,.77)	1.00 (-1.00, 1.00)	.17 (.11,.22)
Contact with mother	.11 (.11,.33)	-1.00 (-1.00, 1.00)	- .07 (-.16,.03)	-.08 (-.89,.42)	1.00 (-1.00, 1.00)	-.13 (-.30,.04)	.14 (.14,.42)	-1.00 (-1.00,1.00)	-.03 (-.14,.08)	.24 (.24,.64)	-1.00 (-1.00,-.89)	-.07 (-.17,.03)
Communication with mother	- .13 (- .39,.13)	-1.00 (-1.00, 1.00)	- .06 (-.16,.03)	-.36 (-1.00,.36)	.67 (-1.00, 1.00)	-.11 (-.30,.07)	-.08 (-.35,.08)	-1.00 (-1.00,1.00)	-.03 (-.15,.08)	-.03 (-.03,.22)	-1.00 (-1.00,-.63)	-.06 (-.16,.04)

Contact with father	.04 (-1.00, 1.00)	-1.00 (-1.00, 1.00)	- .06 (-.14,.03)	-1.00 (-1.00, 1.00)	.76 (-1.00, 1.00)	-.01 (-.16,.13)	.18 (-.24,1.00)	-1.00 (-1.00,1.00)	-.06 (-.17,.04)	.17 (-1.00, 1.00)	-1.00 (-1.00, 1.00)	-.07 (-.16,.02)
Communication with father	- .04 (NA, NA)	-1.00 (-1.00,-1.00)	- .08 (-.17,.00)	-.43 (-1.00,.50)	-.04 (-1.00, 1.00)	-.05 (-.22,.11)	.12 (-1.00,1.00)	-1.00 (-1.00,-.99)	-.09 (-.19,.02)	-.17 (-1.00, 1.00)	-1.00 (-1.00, 1.00)	-.09 (-.18,.00)
Peer pressure	.34 (.25,.49)	.94 (-1.00, 1.00)	.22 (.17,.27)	.46 (.46, 1.00)	-1.00 (-1.00, 1.00)	.18 (.09,.27)	.37 (.25,.51)	-.99 (-1.00,1.00)	.23 (.17,.29)	.37 (.30,.52)	1.00 (-1.00, 1.00)	.21 (.16,.26)
Physical peer victimisation	.34 (.34,.47)	- .34 (-1.00, 1.00)	.11 (.06,.17)	.03 (-1.00,.27)	1.00 (-1.00, 1.00)	.18 (.10,.26)	.74 (.52,1.00)	-.85 (-1.00,1.00)	.04 (-.03,.11)	.32 (.12,.60)	-.85 (-1.00, 1.00)	.13 (.07,.18)
Social peer victimisation	.60 (.49,.83)	1.00 (-1.00, 1.00)	.30 (.25,.35)	.45 (-1.00, 1.00)	1.00 (.89, 1.00)	.35 (.26,.42)	.63 (.54,.82)	-.83 (-1.00,1.00)	.30 (.24,.36)	.67 (.43, 1.00)	1.00 (-1.00, 1.00)	.30 (.25,.36)
Verbal peer victimisation	.56 (.46,.69)	.87 (-1.00, 1.00)	.29 (.24,.33)	.03 (-1.00, 1.00)	1.00 (.87, 1.00)	.32 (.24,.40)	.62 (.54,.86)	-1.00 (-1.00,1.00)	.28 (.22,.34)	.58 (.53,.82)	-.10 (-1.00, 1.00)	.29 (.24,.34)
Cyber peer victimisation	.54 (.46,.65)	.03 (-1.00, 1.00)	.24 (.19,.29)	-.10 (-1.00, 1.00)	1.00 (.48, 1.00)	.28 (.19,.36)	.56 (.48,.70)	-.97 (-1.00,1.00)	.25 (.19,.32)	.53 (.43,.71)	1.00 (-1.00, 1.00)	.24 (.19,.30)
Physical peer perpetration	.28 (.28,.48)	- .61 (-1.00, 1.00)	.08 (.03,.13)	.11 (-1.00, 1.00)	1.00 (-1.00, 1.00)	.11 (.03,.19)	.75 (.35,1.00)	-.36 (-1.00,1.00)	.02 (-.05,.10)	.56 (.07, 1.00)	-1.00 (-1.00, 1.00)	.09 (.03,.14)
Social peer perpetration	.59 (.46,.95)	.63 (NA, 1.00)	.23 (.18,.28)	.58 (-1.00, 1.00)	1.00 (-1.00, 1.00)	.24 (.15,.33)	.63 (.43,1.00)	-1.00 (-1.00,1.00)	.23 (.17,.29)	.71 (.43, 1.00)	-1.00 (-1.00, 1.00)	.23 (.17,.28)
Verbal peer perpetration	.26 (.16,.36)	- .37 (-1.00, 1.00)	.21 (.16,.26)	.13 (-.39,.47)	1.00 (-1.00, 1.00)	.21 (.13,.30)	.33 (.22,.49)	-.84 (-1.00,1.00)	.21 (.15,.27)	.26 (.09,.42)	-1.00 (-1.00, 1.00)	.21 (.16,.26)
Cyber peer perpetration	.31 (.21,.40)	.87 (-1.00, 1.00)	.20 (.15,.25)	.21 (-1.00, 1.00)	1.00 (-1.00, 1.00)	.18 (.09,.27)	.30 (.16,.49)	.42 (-1.00,1.00)	.22 (.16,.28)	.28 (-.01,.32)	1.00 (-1.00, 1.00)	.20 (.15,.25)
Parental role aspirations	- .29 (- .43,-.19)	- .85 (-1.00, 1.00)	- .05 (-.10,.00)	-.37 (-.90,-.37)	-1.00 (-1.00, 1.00)	-.04 (-.13,.04)	-.29 (-.47,-.26)	1.00 (-1.00,1.00)	-.05 (-.12,.01)	-.28 (-.44,-.20)	-1.00 (-1.00, 1.00)	-.04 (-.09,.02)
Occupational role aspirations	- .18 (- .46,-.10)	1.00 (-1.00, 1.00)	.00 (-.05,.05)	-.61 (-1.00,-.13)	1.00 (-1.00, 1.00)	.03 (-.06,.12)	-.10 (-.35,-.10)	1.00 (-1.00,1.00)	-.01 (-.07,.06)	-.32 (-.73,-.07)	1.00 (-1.00, 1.00)	.01 (-.05,.06)
Homecare role aspirations	- .16 (- .26,-.06)	- .50 (-1.00, 1.00)	.01 (-.04,.06)	-.19 (-.56,-.19)	-1.00 (-1.00, 1.00)	.02 (-.07,.11)	-.15 (-.30,-.01)	-.69 (-1.00,1.00)	.00 (-.06,.06)	-.10 (-.32,-.10)	-1.00 (-1.00, 1.00)	.00 (-.05,.06)
Importance of relationships	- .22 (- .30,-.11)	- .96 (-1.00,.98)	- .06 (-.11,- .01)	-.13 (-.13,.19)	-1.00 (-1.00, 1.00)	.00 (-.09,.09)	-.24 (-.33,-.14)	.92 (-1.00,1.00)	-.12 (-.18,-.05)	-.18 (-.29,-.01)	-1.00 (-1.00, 1.00)	-.07 (-.13,-.02)
Achievement motivation	- .13 (- .29,-.03)	.02 (-1.00, 1.00)	- .01 (-.06,.04)	.08 (-.35,.74)	-1.00 (-1.00, 1.00)	-.01 (-.10,.08)	-.13 (-.35,-.04)	.99 (-1.00,1.00)	-.02 (-.08,.04)	-.16 (-.39,-.11)	.88 (-1.00, 1.00)	.00 (-.06,.05)
Purpose in life	- .75 (- .88,-.65)	1.00 (-1.00, 1.00)	- .47 (-.51,- .43)	-1.00 (-1.00,-.72)	.96 (-1.00, 1.00)	-.41 (-.48,-.34)	-.72 (-.84,-.64)	-1.00 (-1.00,1.00)	-.50 (-.54,-.45)	-.75 (-.91,-.61)	-.40 (-1.00, 1.00)	-.47 (-.51,-.42)
Importance of democracy and equality	.06 (-.04,.14)	.09 (NA, 1.00)	.01 (-.04,.06)	.29 (.01,.87)	-1.00 (-1.00, 1.00)	.01 (-.09,.10)	.04 (-.09,.13)	.85 (-1.00,1.00)	.00 (-.07,.06)	.04 (-.12,.16)	1.00 (-1.00, 1.00)	.01 (-.05,.07)
Environmental concerns	.00 (-.11,.11)	1.00 (-1.00, 1.00)	- .06 (-.11,- .01)	-.04 (-.30,.14)	-1.00 (-1.00, 1.00)	.00 (-.09,.10)	.00 (-.15,.15)	1.00 (-1.00,1.00)	-.09 (-.16,-.03)	.02 (-.16,.21)	-1.00 (-1.00, 1.00)	-.06 (-.11,.00)
Religiosity	.08 (-.10,.26)	-1.00 (-1.00, 1.00)	.12 (.06,.17)	-.41 (-1.00,.02)	.13 (-1.00, 1.00)	.25 (.15,.34)	.20 (.00,.41)	-1.00 (-1.00,-1.00)	.06 (-.01,.13)	.03 (-.21,.18)	-1.00 (NA, 1.00)	.12 (.06,.17)
Importance of leisure	- .58 (- .66,-.52)	-1.00 (-1.00, 1.00)	- .27 (-.32,- .22)	-.54 (-.96,-.54)	-1.00 (-1.00, 1.00)	-.30 (-.38,-.21)	-.58 (-.68,-.47)	.44 (-1.00,1.00)	-.26 (-.32,-.20)	-.53 (-.67,-.49)	-1.00 (-1.00, 1.00)	-.28 (-.33,-.22)
Alcohol use	.18 (.03,.34)	-1.00 (-1.00, 1.00)	.16 (.10,.22)	.49 (.49, 1.00)	-1.00 (-1.00,.05)	.16 (.06,.26)	.16 (.04,.27)	-.09 (-1.00,1.00)	.14 (.07,.21)	.15 (-.06,.36)	-1.00 (-1.00, 1.00)	.15 (.08,.21)

Ever smoked	.13 (.02,.22)	.51 (- .95,1.00)	.10 (.05,.16)	.01 (-.47,.01)	1.00 (-1.00,1.00)	.14 (.05,.23)	.15 (.03,.29)	-1.00 (-1.00,1.00)	.09 (.02,.15)	.11 (-.03,.28)	-1.00 (-1.00,1.00)	.11 (.05,.16)
Ever vaped	.35 (.20,.56)	-1.00 (-1.00,1.00)	.06 (.01,.12)	.16 (-.36,.59)	1.00 (-1.00,1.00)	.13 (.03,.22)	.44 (.44,.73)	-1.00 (-1.00,1.00)	.03 (-.03,.10)	.44 (.18,.76)	-1.00 (-1.00,1.00)	.05 (-.01,.11)
Cognitive enhancers	.39 (.26,.56)	1.00 (-1.00,1.00)	.12 (.06,.17)	.23 (-.29,.71)	.05 (-1.00,1.00)	.14 (.05,.23)	.50 (.50,.74)	1.00 (-1.00,1.00)	.11 (.04,.17)	.54 (.42,.88)	-1.00 (-1.00,1.00)	.12 (.07,.18)
Big 5 Agreeableness	- .17 (- .29,-.06)	.99 (-1.00,1.00)	- .08 (-.14,- .03)	-.31 (-1.00,-.03)	1.00 (-1.00,1.00)	-.04 (-.13,.05)	-.26 (-.40,-.11)	-.57 (-1.00,1.00)	-.10 (-.16,-.04)	-.20 (-.39,-.16)	1.00 (-1.00,1.00)	-.10 (-.15,-.04)
Big 5 Conscientiousness	- .46 (- .56,-.38)	- .83 (-1.00,1.00)	- .17 (-.22,- .12)	-.39 (-.75,-.39)	-1.00 (-1.00,1.00)	-.21 (-.29,-.12)	-.52 (-.65,-.42)	-.75 (-1.00,1.00)	-.15 (-.21,-.09)	-.46 (-.60,-.37)	-.49 (-1.00,1.00)	-.17 (-.22,-.12)
Big 5 Extraversion	- .56 (- .63,-.49)	- .50 (-1.00,1.00)	- .28 (-.33,- .24)	-.45 (-.77,-.26)	-1.00 (-1.00,1.00)	-.27 (-.35,-.19)	-.62 (-.70,-.55)	-.89 (-1.00,1.00)	-.30 (-.36,-.24)	-.57 (-.65,-.47)	-1.00 (-1.00,1.00)	-.29 (-.34,-.24)
Big 5 Neuroticism	.83 (.77,.94)	1.00 (-1.00,1.00)	.56 (.52,.59)	.92 (.66,1.00)	.12 (-1.00,1.00)	.54 (.48,.61)	.83 (.79,.93)	.05 (-1.00,1.00)	.56 (.52,.60)	.83 (.78,.95)	1.00 (-1.00,1.00)	.55 (.51,.59)
Big 5 Openness	.19 (.11,.28)	- .80 (-1.00,1.00)	.05 (.00,.11)	.18 (-.11,.49)	1.00 (-1.00,1.00)	.17 (.08,.25)	.19 (.09,.29)	.15 (-1.00,1.00)	-.01 (-.07,.06)	.16 (.04,.30)	1.00 (-1.00,1.00)	.05 (.00,.10)
Self-control	- .61 (- .68,-.55)	- .85 (-1.00,1.00)	- .34 (-.38,- .29)	-.63 (-1.00,-.63)	-1.00 (-1.00,1.00)	-.37 (-.44,-.29)	-.62 (-.70,-.55)	-.67 (-1.00,1.00)	-.32 (-.37,-.26)	-.62 (-.69,-.53)	.20 (NA,1.00)	-.33 (-.38,-.28)
Not planning for the future	.30 (.26,.48)	1.00 (-1.00,1.00)	.12 (.07,.17)	.47 (.09,1.00)	-.91 (-1.00,1.00)	.09 (.00,.18)	.26 (.26,.79)	1.00 (-1.00,1.00)	.12 (.06,.19)	.27 (.22,.51)	.68 (-1.00,1.00)	.11 (.06,.16)
Ambition	- .29 (- .49,-.19)	1.00 (-1.00,1.00)	- .07 (-.12,- .02)	-.44 (-1.00,-.09)	1.00 (-1.00,1.00)	-.09 (-.19,.00)	-.25 (-.49,-.15)	.31 (-1.00,1.00)	-.06 (-.12,.01)	-.35 (-.59,-.19)	1.00 (-1.00,1.00)	-.06 (-.12,-.01)
Physical activity	- .28 (- .37,-.16)	- .95 (-1.00,1.00)	- .01 (-.07,.04)	-.33 (-.75,.01)	-1.00 (-1.00,1.00)	-.06 (-.15,.03)	-.25 (-.36,-.11)	-.93 (-1.00,1.00)	.01 (-.05,.07)	-.18 (-.35,-.13)	-1.00 (-1.00,1.00)	-.01 (-.07,.05)
Health behaviours	- .31 (- .43,-.19)	-1.00 (-1.00,1.00)	- .07 (-.13,- .02)	-.53 (-1.00,-.50)	1.00 (-1.00,1.00)	-.02 (-.12,.08)	-.27 (-.41,-.12)	-1.00 (-1.00,1.00)	-.10 (-.17,-.03)	-.23 (-.39,-.07)	-1.00 (-1.00,1.00)	-.07 (-.12,-.01)
Risk taking behaviour	.10 (.00,.19)	.53 (-1.00,1.00)	.12 (.07,.17)	.07 (-.38,.29)	-.36 (-1.00,1.00)	.13 (.03,.23)	.15 (.04,.24)	-.95 (-1.00,1.00)	.11 (.05,.18)	.09 (-.08,.22)	1.00 (-1.00,1.00)	.12 (.06,.18)
Risky sexual behaviour	.08 (.00,.18)	- .01 (-1.00,1.00)	- .02 (-.07,.03)	.29 (.29,.78)	-1.00 (-1.00,1.00)	-.09 (-.18,.01)	.05 (-.08,.15)	1.00 (-1.00,1.00)	.01 (-.06,.08)	.12 (-.05,.21)	-1.00 (-1.00,1.00)	-.02 (-.08,.04)
Media use	.01 (-.05,.09)	- .58 (-1.00,1.00)	.08 (.02,.13)	-.07 (-.34,-.07)	-1.00 (-1.00,1.00)	.09 (.00,.18)	.05 (-.04,.14)	-.79 (-1.00,NA)	.07 (.00,.13)	-.01 (-.10,.03)	-1.00 (-1.00,1.00)	.06 (.01,.12)
Volunteering	.05 (-.10,.13)	1.00 (-1.00,1.00)	.02 (-.03,.07)	-.25 (-.96,-.25)	1.00 (-1.00,1.00)	.10 (.01,.19)	.09 (-.03,.21)	.82 (-1.00,1.00)	.00 (-.07,.06)	.00 (-.19,.17)	1.00 (-1.00,1.00)	.03 (-.02,.09)
Internet dating	.10 (.07,.20)	-1.00 (-1.00,1.00)	.10 (.04,.15)	.09 (-.44,.55)	-1.00 (-1.00,1.00)	.13 (.03,.22)	.13 (-.03,.31)	.99 (-1.00,1.00)	.08 (.02,.14)	.01 (-.26,.24)	1.00 (-1.00,1.00)	.08 (.03,.14)
Internet dating	.11 (-.01,.25)	- .72 (-1.00,1.00)	.08 (.03,.13)	.07 (-.46,.55)	-1.00 (-1.00,1.00)	.11 (.01,.20)	.14 (.00,.29)	.00 (-1.00,1.00)	.07 (.01,.13)	-.01 (-.30,-.01)	1.00 (-1.00,1.00)	.07 (.02,.13)
Financial literacy: knowledge of products	- .30 (- .42,-.21)	.91 (-1.00,1.00)	- .03 (-.08,.02)	-.23 (-.63,.12)	-1.00 (-1.00,1.00)	-.05 (-.15,.04)	-.34 (-.50,-.23)	.94 (-1.00,1.00)	-.02 (-.09,.04)	-.32 (-.56,-.14)	1.00 (-1.00,1.00)	-.03 (-.09,.03)
Financial attitudes and behaviour	- .25 (- .38,-.16)	.05 (-1.00,1.00)	- .14 (-.19,- .09)	-.27 (-.76,.17)	.80 (-1.00,NA)	-.16 (-.25,-.07)	-.26 (-.40,-.15)	.38 (-1.00,1.00)	-.12 (-.18,-.06)	-.24 (-.44,-.19)	.99 (-1.00,1.00)	-.12 (-.18,-.07)
Trait	Model fitting results											

	Functional outcome: adverse physical health											
	Whole sample			Males only			Females only			Same-sex MZ and DZ twins		
	rA	rC	rE	rA	rC	rE	rA	rC	rE	rA	rC	rE
Daily hassles	.46 (.38,.69)	1.00 (-1.00, 1.00)	.18 (.14,.23)	.50 (.31,.99)	-.89 (-1.00, 1.00)	.17 (.10,.25)	.37 (.37,.72)	.96 (- 1.00,1.00)	.19 (.13,.25)	.44 (.44,.73)	1.00 (-1.00, 1.00)	.18 (.13,.23)
CHAOS	.27 (.27,.81)	1.00 (.83, 1.00)	.15 (.10,.20)	.38 (- .01,.79)	-1.00 (-1.00, 1.00)	.09 (.00,.18)	.22 (- 1.00,1.00)	1.00 (.31,1.00)	.17 (.11,.23)	.09 (-1.00, 1.00)	.80 (.72, 1.00)	.16 (.11,.21)
Childhood experiences	.32 (.24,.49)	1.00 (-1.00, 1.00)	.15 (.10,.21)	.05 (- .29,.05)	1.00 (-1.00, NA)	.29 (.20,.38)	.42 (.18,.67)	.26 (- 1.00,1.00)	.11 (.05,.17)	.39 (.39,.65)	.09 (-1.00, 1.00)	.16 (.10,.21)
Poor sleep quality	.74 (.52, 1.00)	-1.00 (-1.00, 1.00)	.24 (.19,.28)	.67 (.67, 1.00)	1.00 (-1.00, 1.00)	.24 (.16,.31)	.90 (.71,1.00)	-.47 (- 1.00,1.00)	.23 (.17,.29)	.88 (.88, NA)	.37 (-1.00, 1.00)	.23 (.19,.28)
Marriage hopes	.06 (.06,.36)	-1.00 (NA,- 1.00)	- .01 (- .05,.04)	.96 (-1.00, 1.00)	-1.00 (-1.00, 1.00)	.02 (- .06,.11)	.06 (.06,.44)	-1.00 (- 1.00,-.44)	-.02 (- .08,.04)	.19 (- .31,.96)	-.78 (- 1.00,.09)	-.01 (- .06,.04)
Marriage worries	.11 (- .11,.28)	1.00 (-1.00, 1.00)	.09 (.04,.14)	.29 (.06,.52)	.76 (-1.00, 1.00)	-.01 (- .09,.07)	-.02 (- .38,.26)	1.00 (- 1.00,1.00)	.13 (.08,.19)	.02 (- .30,.34)	.89 (-1.00, 1.00)	.10 (.05,.15)
Quality of relationship with twin	- .22 (- .44,- .01)	- .70 (-1.00, 1.00)	.02 (- .04,.07)	-.19 (- .60,.12)	-1.00 (-1.00, 1.00)	.04 (- .05,.14)	-.28 (-.57,- .01)	-.23 (- 1.00,1.00)	.01 (- .06,.08)	-.27 (- .62,.04)	-.30 (- 1.00,.01)	.01 (- .04,.07)
Quality of relationship with mother	- .14 (- .31,.01)	1.00 (-1.00, 1.00)	- .06 (- .11,- .01)	-.03 (- .30,.13)	-.44 (-1.00, 1.00)	-.07 (- .15,.01)	-.18 (- .43,.06)	.51 (- 1.00,1.00)	-.06 (- .13,.00)	-.14 (- .40,.11)	.16 (-1.00, 1.00)	-.06 (- .11,.00)
Quality of relationship with father	- .30 (- .52,- .09)	1.00 (-1.00, 1.00)	- .05 (- .10,.00)	-.20 (- .56,.10)	1.00 (-1.00, 1.00)	-.10 (-.19,- .01)	-.34 (-.65,- .06)	.16 (- 1.00,1.00)	-.03 (- .10,.03)	-.25 (- .56,.04)	-.06 (-1.00, 1.00)	-.05 (- .11,.00)
Number of relationships	.31 (.19,.42)	1.00 (-1.00, 1.00)	.03 (- .02,.08)	.22 (.09,.45)	-.50 (-1.00, 1.00)	.03 (- .05,.11)	.30 (.08,.49)	1.00 (- 1.00,1.00)	.04 (- .02,.10)	.23 (.00,.50)	.79 (-1.00, 1.00)	.04 (- .01,.09)
Longest relationship	.13 (- .04,.28)	1.00 (-1.00, 1.00)	- .06 (- .11,- .01)	.15 (- .22,.15)	.58 (-1.00, 1.00)	-.10 (-.18,- .01)	.13 (- .10,.39)	1.00 (- 1.00,1.00)	-.04 (- .10,.02)	.05 (- .27,.35)	1.00 (-1.00, 1.00)	-.04 (- .10,.01)
Partner violence	.54 (.54,.85)	1.00 (-1.00, NA)	.08 (.03,.13)	.42 (.42, 1.00)	-1.00 (-1.00, 1.00)	.08 (- .01,.16)	.57 (.57,.87)	1.00 (- 1.00,1.00)	.09 (.03,.14)	.54 (.54,.91)	1.00 (-1.00, 1.00)	.08 (.03,.13)
Contact with mother	.08 (- .13,.39)	1.00 (-1.00, 1.00)	- .05 (- .13,.04)	.00 (- .63,.00)	1.00 (-1.00, 1.00)	-.03 (- .18,.11)	.20 (- .18,.56)	-.09 (- 1.00,1.00)	-.06 (- .17,.05)	.07 (.07,.54)	.29 (-1.00, 1.00)	-.05 (- .14,.04)
Communication with mother	- .13 (- .46,.10)	1.00 (-1.00, 1.00)	- .05 (- .13,.04)	-.07 (- .62,.51)	1.00 (-1.00, 1.00)	-.11 (- .25,.05)	-.07 (- .55,.40)	.09 (- 1.00,1.00)	-.03 (- .14,.07)	-.13 (- .57,.31)	.31 (-1.00, 1.00)	-.04 (- .13,.05)
Contact with father	- .06 (-1.00, 1.00)	1.00 (-1.00, 1.00)	- .01 (- .09,.06)	.00 (-1.00, 1.00)	1.00 (-1.00, 1.00)	-.02 (- .15,.12)	-.04 (- 1.00,1.00)	.19 (- 1.00,1.00)	-.02 (- .12,.08)	-.08 (-1.00, 1.00)	.11 (-1.00, 1.00)	-.02 (- .10,.07)
Communication with father	- .02 (- .02,.48)	-1.00 (NA, 1.00)	- .10 (- .17,- .02)	-.10 (-1.00, 1.00)	-1.00 (-1.00, 1.00)	-.02 (- .16,.12)	.04 (- 1.00,1.00)	-.38 (- 1.00,1.00)	-.14 (- .23,-.04)	-.20 (-1.00, 1.00)	-.10 (-1.00, 1.00)	-.10 (-.18,- .03)
Peer pressure	.30 (.15,.54)	-1.00 (-1.00, 1.00)	.08 (.03,.12)	.21 (.04,.53)	-.87 (-1.00, 1.00)	.11 (.03,.19)	.36 (.34,.71)	-1.00 (- 1.00,1.00)	.06 (.00,.12)	.42 (.18,.72)	-1.00 (-1.00, 1.00)	.08 (.03,.13)
Physical peer victimisation	.32 (.01, NA)	1.00 (-1.00, 1.00)	.06 (.01,.11)	.05 (- 1.00,.05)	1.00 (-1.00, 1.00)	.10 (.02,.18)	.86 (.39,1.00)	.93 (- 1.00,1.00)	.01 (- .05,.08)	.28 (.28,.68)	1.00 (-1.00, 1.00)	.09 (.04,.14)
Social peer victimisation	.52 (.33,.85)	1.00 (-1.00, 1.00)	.08 (.03,.13)	1.00 (-1.00, 1.00)	1.00 (-1.00, 1.00)	.11 (.02,.20)	.58 (.52,.88)	1.00 (- 1.00,1.00)	.07 (.01,.13)	.55 (.12, 1.00)	.48 (-1.00, 1.00)	.08 (.03,.14)

Verbal peer victimisation	.50 (.50,.74)	-1.00 (-1.00, 1.00)	.10 (.05,.15)	.44 (-1.00, 1.00)	1.00 (-1.00, 1.00)	.07 (-.02,.15)	.61 (.40,.97)	-1.00 (-1.00,1.00)	.12 (.06,.18)	.46 (.46,.86)	.85 (-.39, 1.00)	.10 (.05,.15)
Cyber peer victimisation	.55 (.42,.79)	- .88 (-1.00, 1.00)	.09 (.04,.14)	.25 (-1.00, 1.00)	1.00 (-.30, 1.00)	.14 (.05,.23)	.71 (.52,1.00)	-1.00 (-1.00,1.00)	.07 (.01,.13)	.62 (.62,.95)	.89 (-1.00, 1.00)	.09 (.04,.14)
Physical peer perpetration	.36 (.08,.69)	1.00 (- .83, 1.00)	- .01 (-.05,.04)	.25 (-1.00, 1.00)	.94 (-1.00, 1.00)	.03 (-.05,.10)	1.00 (.55,1.00)	-1.00 (-1.00,1.00)	-.07 (-.13,.00)	.51 (-1.00, 1.00)	-.63 (-1.00, 1.00)	-.01 (-.06,.05)
Social peer perpetration	.40 (.25,.86)	-1.00 (-1.00, 1.00)	.10 (.05,.14)	.35 (.35,.99)	-.74 (-1.00, 1.00)	.08 (.00,.17)	.50 (.08,1.00)	-1.00 (-1.00,1.00)	.10 (.04,.16)	.58 (.05, 1.00)	-.53 (-1.00, 1.00)	.09 (.03,.14)
Verbal peer perpetration	.33 (.21,.53)	- .99 (-1.00, 1.00)	.04 (-.01,.08)	.29 (.07,.57)	-.12 (-1.00, 1.00)	.00 (-.08,.08)	.43 (.43,.80)	-1.00 (-1.00,1.00)	.05 (-.01,.11)	.33 (.23,.66)	1.00 (-1.00, 1.00)	.03 (-.02,.08)
Cyber peer perpetration	.37 (.31,.64)	-1.00 (-1.00, 1.00)	.07 (.02,.12)	.31 (-.20,.79)	.88 (-1.00, 1.00)	.05 (-.04,.13)	.54 (.54,1.00)	-1.00 (-1.00,1.00)	.07 (.01,.13)	.43 (.43,.89)	-.01 (-1.00, 1.00)	.07 (.02,.12)
Parental role aspirations	- .04 (-.21,.13)	.63 (-1.00, 1.00)	- .05 (-.09,.00)	.16 (-.04,.59)	-1.00 (-1.00, 1.00)	-.13 (-.21,-.05)	-.18 (-.52,.12)	.59 (-1.00,1.00)	.00 (-.06,.06)	-.02 (-.28,.26)	-1.00 (-1.00, 1.00)	-.05 (-.10,.00)
Occupational role aspirations	- .16 (-.46,.02)	1.00 (-1.00, 1.00)	- .01 (-.05,.04)	-.07 (-.96,.08)	1.00 (-1.00, 1.00)	-.05 (-.12,.04)	-.16 (-.53,-.16)	1.00 (-1.00,1.00)	.01 (-.05,.07)	-.27 (-.71,.07)	1.00 (-1.00, 1.00)	.00 (-.05,.05)
Homecare role aspirations	- .04 (-.17,.14)	-1.00 (-1.00, 1.00)	.01 (-.04,.06)	-.09 (-.26,.19)	.38 (-1.00, 1.00)	-.01 (-.09,.07)	.00 (-.25,.27)	-1.00 (-1.00,1.00)	.03 (-.03,.09)	.03 (-.08,.34)	-.60 (-1.00, 1.00)	-.01 (-.06,.05)
Importance of relationships	.01 (-.09,.16)	-1.00 (-1.00, 1.00)	- .03 (-.07,.02)	.14 (.00,.53)	-1.00 (-1.00, 1.00)	-.06 (-.13,.02)	-.08 (-.27,.02)	-1.00 (-1.00,1.00)	-.01 (-.07,.05)	.07 (-.03,.32)	-1.00 (-1.00, 1.00)	-.04 (-.09,.01)
Achievement motivation	- .09 (-.25,.08)	-1.00 (-1.00, 1.00)	.01 (-.04,.05)	.06 (-.13,.60)	-1.00 (-1.00, 1.00)	-.03 (-.11,.06)	-.14 (-.42,.05)	1.00 (-1.00,1.00)	.02 (-.04,.08)	-.04 (-.37,.25)	-.63 (-1.00, 1.00)	.01 (-.05,.06)
Purpose in life	- .40 (- .60,-.22)	1.00 (-1.00, 1.00)	- .18 (-.23,- .13)	-.35 (-.76,-.08)	1.00 (-1.00, 1.00)	-.19 (-.27,-.10)	-.41 (-.66,-.36)	1.00 (-1.00,1.00)	-.18 (-.24,-.12)	-.53 (-.83,-.25)	.59 (-1.00, 1.00)	-.18 (-.23,-.13)
Importance of democracy and equality	.03 (-.09,.15)	.26 (-1.00, NA)	- .04 (-.08,.01)	.17 (.11,.50)	1.00 (-1.00, 1.00)	-.10 (-.18,-.01)	-.05 (-.22,-.03)	-.40 (-1.00,1.00)	-.01 (-.07,.05)	-.01 (-.09,.22)	.00 (-1.00, 1.00)	-.04 (-.09,.01)
Environmental concerns	- .11 (-.26,.03)	-1.00 (-1.00, 1.00)	- .05 (-.10,.00)	-.11 (-.26,.16)	-1.00 (-1.00, 1.00)	-.07 (-.15,.02)	-.18 (-.43,-.11)	.39 (-1.00,1.00)	-.03 (-.10,.03)	-.10 (-.35,-.10)	-.30 (-1.00, 1.00)	-.05 (-.11,.00)
Religiosity	.11 (-.11,.33)	-1.00 (-1.00, 1.00)	.01 (-.05,.06)	.10 (-.28,.50)	-1.00 (-1.00, 1.00)	-.02 (-.11,.07)	.13 (-.14,.42)	-.25 (-1.00,1.00)	.02 (-.05,.08)	.20 (-.10,.52)	-.15 (-1.00, 1.00)	.01 (-.04,.06)
Importance of leisure	- .28 (- .42,-.10)	-1.00 (-1.00, 1.00)	- .16 (-.21,- .12)	-.08 (-.31,.42)	-1.00 (NA, 1.00)	-.21 (-.29,-.13)	-.36 (-.58,-.15)	-1.00 (-1.00,1.00)	-.15 (-.21,-.09)	-.21 (-.47,-.02)	-1.00 (-1.00, 1.00)	-.18 (-.23,-.13)
Alcohol use	.32 (.13,.55)	-1.00 (NA, 1.00)	.03 (-.02,.09)	.51 (.51, 1.00)	-1.00 (-1.00, 1.00)	.01 (-.09,.11)	.23 (.02,.49)	-1.00 (-1.00,1.00)	.05 (-.02,.12)	.47 (.16,.83)	-.99 (-1.00,.17)	.02 (-.04,.08)
Ever smoked	.15 (.02,.30)	- .44 (-1.00, 1.00)	.07 (.03,.12)	.15 (-.02,.40)	-.95 (-1.00, 1.00)	.07 (-.01,.16)	.12 (-.10,.36)	1.00 (-1.00,1.00)	.08 (.02,.14)	.17 (-.06,.40)	-.11 (-1.00, 1.00)	.07 (.02,.12)
Ever vaped	.34 (.25,.59)	-1.00 (NA, 1.00)	.06 (.00,.11)	.25 (.16,.62)	-1.00 (-1.00, 1.00)	.08 (-.01,.16)	.38 (.24,.76)	.08 (-1.00,1.00)	.05 (-.01,.11)	.32 (-.04,.71)	.15 (-1.00, 1.00)	.05 (-.01,.10)
Cognitive enhancers	.44 (.23,.69)	-1.00 (-1.00, 1.00)	.06 (.01,.11)	.23 (-.04,.67)	-1.00 (-1.00, 1.00)	.13 (.04,.21)	.54 (.45,.97)	-.41 (-1.00,1.00)	.03 (-.03,.10)	.58 (.20, 1.00)	-.25 (-1.00, 1.00)	.06 (.00,.11)
Big 5 Agreeableness	- .12 (-.28,.03)	- .72 (-1.00, 1.00)	- .03 (-.07,.02)	-.01 (-.29,.03)	-.76 (-1.00, 1.00)	-.06 (-.14,.03)	-.20 (-.42,.06)	-1.00 (-1.00,.96)	-.01 (-.07,.04)	-.15 (-.40,.11)	-.30 (-1.00, 1.00)	-.04 (-.09,.02)
Big 5 Conscientiousness	- .28 (- .41,-.15)	-1.00 (-1.00, NA)	- .09 (-.14,- .04)	-.13 (-.31,.09)	1.00 (-1.00, 1.00)	-.12 (-.20,-.04)	-.38 (-.61,-.19)	-1.00 (-1.00,1.00)	-.08 (-.14,-.02)	-.29 (-.50,-.09)	-1.00 (-1.00, 1.00)	-.11 (-.16,-.05)

Big 5 Extraversion	- .28 (- .37,-.15)	-1.00 (-1.00, 1.00)	- .08 (-.13,- .03)	-.12 (-.27,.10)	-.92 (-1.00, 1.00)	-.14 (-.22,-.06)	-.35 (-.51,-.17)	-1.00 (-1.00,1.00)	-.06 (-.12,.00)	-.25 (-.41,-.07)	-1.00 (-1.00, 1.00)	-.09 (-.15,-.04)
Big 5 Neuroticism	.50 (.34,.71)	-1.00 (-1.00, 1.00)	.20 (.15,.25)	.52 (.25, 1.00)	-1.00 (-1.00, 1.00)	.23 (.15,.31)	.51 (.32,.76)	1.00 (.93,1.00)	.18 (.12,.24)	.61 (.32,.88)	-.28 (-1.00, 1.00)	.20 (.15,.25)
Big 5 Openness	.09 (-.01,.26)	-1.00 (-1.00, 1.00)	- .02 (-.06,.03)	.11 (-.06,.39)	.74 (-1.00, 1.00)	-.01 (-.10,.07)	.10 (.04,.32)	-1.00 (-1.00,1.00)	-.02 (-.08,.04)	.10 (.09,.34)	-.49 (-1.00, 1.00)	-.03 (-.08,.02)
Self-control	- .39 (- .55,-.28)	.87 (-1.00, 1.00)	- .17 (-.22,- .12)	-.34 (-.64,-.16)	-.47 (-1.00, 1.00)	-.16 (-.24,-.08)	-.43 (-.65,-.26)	-1.00 (-1.00,1.00)	-.18 (-.24,-.12)	-.46 (-.73,-.46)	1.00 (-1.00, 1.00)	-.17 (-.23,-.12)
Not planning for the future	.04 (-.25,.26)	1.00 (-1.00, 1.00)	.09 (.04,.14)	.09 (.09,.42)	-.95 (-1.00, 1.00)	.11 (.03,.19)	-.21 (-1.00,.18)	1.00 (1.00,1.00)	.10 (.04,.16)	-.02 (-.45,.03)	1.00 (-1.00, 1.00)	.10 (.05,.15)
Ambition	- .17 (- .38,-.03)	1.00 (-1.00, 1.00)	- .04 (-.09,.01)	-.01 (-.40,.04)	1.00 (-1.00, 1.00)	-.13 (-.21,-.05)	-.20 (-.48,-.18)	-.98 (-1.00,1.00)	.00 (-.06,.06)	-.20 (-.47,.05)	-.99 (-1.00, 1.00)	-.04 (-.09,.02)
Physical activity	- .20 (- .32,-.04)	-1.00 (-1.00, 1.00)	- .07 (-.12,- .03)	-.13 (-.31,.17)	-.87 (-1.00, 1.00)	-.10 (-.18,-.02)	-.23 (-.45,-.02)	-1.00 (-1.00,1.00)	-.06 (-.12,.00)	-.11 (-.36,.15)	-1.00 (-1.00, 1.00)	-.08 (-.13,-.03)
Health behaviours	- .21 (- .36,-.07)	-1.00 (-1.00, 1.00)	- .10 (-.15,- .05)	-.19 (-.37,.01)	-.34 (-1.00, 1.00)	-.15 (-.23,-.06)	-.18 (-.39,.03)	-1.00 (-1.00,1.00)	-.08 (-.14,-.02)	-.15 (-.38,.06)	-.92 (-1.00, 1.00)	-.10 (-.16,-.05)
Risk taking behaviour	.20 (.06,.37)	-1.00 (-1.00, 1.00)	.12 (.07,.17)	.25 (.25,.43)	-1.00 (-1.00, 1.00)	.11 (.02,.20)	.18 (.08,.40)	-1.00 (-1.00,1.00)	.12 (.06,.18)	.26 (.03,.51)	-1.00 (-1.00, 1.00)	.12 (.06,.17)
Risky sexual behaviour	.17 (.04,.29)	1.00 (-1.00, 1.00)	.05 (.00,.10)	.20 (.18,.51)	-1.00 (-1.00,.68)	.03 (-.06,.12)	.08 (-.12,.12)	1.00 (-1.00,1.00)	.07 (.01,.13)	.16 (.04,.39)	.21 (-1.00, 1.00)	.06 (.00,.11)
Media use	.05 (-.05,.13)	.21 (-1.00, 1.00)	.10 (.05,.15)	.02 (-.12,.18)	-.70 (-1.00, 1.00)	.08 (.00,.16)	.05 (-.14,.12)	1.00 (-1.00,1.00)	.11 (.05,.17)	.01 (-.18,.15)	1.00 (-1.00, 1.00)	.09 (.04,.14)
Volunteering	.18 (.05,.37)	-1.00 (-1.00,.84)	.03 (-.02,.08)	.14 (-.08,.34)	.70 (-1.00, 1.00)	.03 (-.06,.11)	.26 (.04,.55)	-1.00 (-1.00,1.00)	.03 (-.03,.09)	.18 (-.09,.46)	-.03 (-1.00, 1.00)	.02 (-.04,.07)
Internet dating	- .02 (-.28,.17)	1.00 (-1.00, 1.00)	.08 (.03,.13)	-.02 (-.42,.23)	1.00 (-1.00, NA)	.06 (-.02,.15)	-.03 (-.41,.24)	1.00 (-1.00,1.00)	.09 (.03,.15)	-.20 (-.62,.10)	1.00 (.26, 1.00)	.09 (.04,.14)
Internet dating	.10 (-.12,.25)	1.00 (-1.00, 1.00)	.05 (.01,.10)	.00 (-.33,.30)	.47 (-1.00, 1.00)	.06 (-.02,.14)	.11 (-.22,.33)	1.00 (-1.00,1.00)	.06 (.00,.12)	-.16 (-.58,.19)	1.00 (.94, 1.00)	.06 (.01,.11)
Financial literacy: knowledge of products	- .09 (-.21,.10)	-1.00 (-1.00, 1.00)	- .03 (-.08,.01)	-.10 (-.33,.13)	.20 (-1.00, 1.00)	-.06 (-.14,.02)	-.04 (-.26,.27)	-1.00 (-1.00,1.00)	-.02 (-.08,.04)	.01 (-.28,.31)	-1.00 (-1.00, 1.00)	-.05 (-.10,.01)
Financial attitudes and behaviour	- .16 (-.30,.01)	-1.00 (-1.00, 1.00)	- .15 (-.20,- .11)	-.12 (-.31,-.12)	.08 (-1.00, 1.00)	-.21 (-.29,-.13)	-.15 (-.36,.14)	-1.00 (-1.00,1.00)	-.13 (-.19,-.07)	-.17 (-.44,.00)	.90 (-1.00, 1.00)	-.16 (-.21,-.11)
	Model fitting results											
	Functional outcome: achieved educational level											
	Whole sample			Males only			Females only			Same-sex MZ and DZ twins		
Trait	rA	rC	rE	rA	rC	rE	rA	rC	rE	rA	rC	rE
Daily hassles	.07 (.07,.39)	- .98 (-1.00, 1.00)	.00 (-.05,.05)	.00 (-.55,.57)	1.00 (-1.00, 1.00)	-.01 (-.09,.07)	.12 (-.28,.56)	-.25 (-1.00,1.00)	.00 (-.06,.06)	.09 (.09,.44)	-1.00 (-1.00, 1.00)	.00 (-.05,.06)
CHAOS	- .09 (- .62,-.09)	- .33 (- .65,-.07)	.01 (-.04,.06)	-.39 (-1.00,-.39)	-.18 (-1.00,.57)	.09 (.00,.17)	.24 (-1.00,1.00)	-.36 (-.73,-.08)	-.03 (-.09,.03)	-.09 (-1.00, 1.00)	-.32 (-.83,.02)	.01 (-.04,.06)
Childhood experiences	- .05 (-.27,.16)	- .18 (- .56,-.13)	.01 (-.04,.07)	-.25 (-.77,-.25)	-.08 (-1.00, 1.00)	.07 (-.02,.16)	.01 (-.11,.28)	-.16 (-.55,-.16)	-.01 (-.08,.06)	-.03 (-.30,.24)	-.19 (-.69,.16)	.01 (-.04,.07)

Poor sleep quality	.25 (-.05,.64)	-1.00 (-1.00,- .41)	- .03 (-.08,.02)	.37 (.37, 1.00)	-1.00 (-1.00,-.93)	-.02 (-.10,.06)	.21 (-.15,.70)	-1.00 (-1.00,-1.00)	-.03 (-.09,.03)	.50 (.02, 1.00)	-1.00 (-1.00,-.38)	-.03 (-.08,.02)
Marriage hopes	.61 (.26, 1.00)	- .25 (-1.00,-.25)	- .04 (-.09,.01)	1.00 (.21, 1.00)	-.34 (-1.00,-.34)	-.04 (-.11,.04)	.45 (.08,.95)	-.08 (-1.00,1.00)	-.03 (-.09,.03)	.67 (.40, 1.00)	-.26 (-1.00,.24)	-.04 (-.09,.00)
Marriage worries	.03 (-.08,.29)	- .97 (-1.00, 1.00)	- .05 (-.09,.00)	-.09 (-.57,.33)	1.00 (-1.00, 1.00)	-.03 (-.11,.05)	.14 (-.11,.54)	-.45 (-1.00,1.00)	-.06 (-.12,.00)	.05 (-.24,.39)	-.21 (-1.00, 1.00)	-.05 (-.09,.00)
Quality of relationship with twin	.00 (-.22,.23)	.18 (.02,.38)	- .04 (-.09,.01)	-.04 (-.48,.41)	.24 (-.03, 1.00)	-.04 (-.13,.05)	.02 (-.16,.31)	.15 (-.06,.41)	-.04 (-.11,.02)	.04 (-.27,.36)	.20 (-.02,.54)	-.04 (-.09,.01)
Quality of relationship with mother	.00 (-.20,.20)	1.00 (-1.00, NA)	.00 (-.05,.05)	.07 (-.07,.43)	1.00 (-1.00, 1.00)	-.02 (-.10,.06)	.00 (-.26,.26)	.13 (-1.00,1.00)	.00 (-.06,.07)	.11 (-.13,.37)	-.24 (-1.00, 1.00)	.00 (-.06,.05)
Quality of relationship with father	.00 (-.23,.23)	.22 (.17,.54)	.01 (-.04,.06)	-.04 (-.49,.39)	.40 (-.14, 1.00)	-.01 (-.10,.08)	.05 (-.08,.35)	.13 (-.20,.47)	.02 (-.04,.09)	.14 (-.14,.42)	.02 (-.30,.14)	.01 (-.04,.06)
Number of relationships	- .22 (- .42,-.04)	-1.00 (-1.00, 1.00)	- .01 (-.06,.04)	-.32 (-.72,-.03)	1.00 (-1.00, 1.00)	-.07 (-.14,.01)	-.09 (-.34,.14)	-1.00 (-1.00,1.00)	.02 (-.04,.08)	-.13 (-.39,.11)	-.85 (-1.00, 1.00)	-.02 (-.07,.03)
Longest relationship	- .01 (-.26,.30)	-1.00 (-1.00, 1.00)	- .05 (-.10,.00)	.13 (-.39, 1.00)	-1.00 (-1.00,.35)	-.05 (-.14,.03)	-.07 (-.37,.27)	-1.00 (-1.00,1.00)	-.05 (-.11,.01)	-.01 (-.27,.35)	-1.00 (-1.00, 1.00)	-.06 (-.11,-.01)
Partner violence	- .23 (-.55,.08)	-1.00 (-1.00, 1.00)	- .01 (-.06,.04)	-.59 (-1.00,-.59)	1.00 (-1.00, 1.00)	.05 (-.03,.13)	-.08 (-.46,.39)	-1.00 (-1.00,1.00)	-.03 (-.09,.02)	-.27 (-.65,-.24)	-1.00 (-1.00, 1.00)	-.01 (-.06,.04)
Contact with mother	- .40 (- .76,-.40)	- .33 (-1.00, 1.00)	- .12 (-.21,- .04)	-.31 (-1.00,-.31)	-.23 (-1.00, 1.00)	-.12 (-.27,.03)	-.43 (-.81,-.43)	-.40 (-1.00,1.00)	-.13 (-.23,-.03)	-.21 (-.67,-.21)	-.63 (-1.00,.16)	-.12 (-.20,-.03)
Communication with mother	- .02 (-.41,.37)	- .01 (-.53,.49)	- .03 (-.12,.05)	.18 (-.58, 1.00)	-.38 (-1.00,.60)	.00 (-.16,.15)	-.16 (-.66,.31)	.21 (-.37,1.00)	-.05 (-.15,.06)	-.08 (-.52,.38)	.11 (.11, 1.00)	-.03 (-.12,.06)
Contact with father	-1.00 (-1.00,- .50)	.25 (-.07,.73)	.01 (-.06,.08)	-1.00 (-1.00,-.50)	.49 (.49, 1.00)	-.03 (-.14,.08)	-.81 (-1.00,-.23)	.10 (-.35,.68)	.02 (-.08,.11)	-.81 (-1.00, NA)	-.06 (-.28,.48)	.02 (-.06,.10)
Communication with father	- .13 (- .69,-.13)	.20 (-.04,.48)	- .01 (-.09,.07)	-.33 (-1.00,.50)	.38 (-.12, 1.00)	-.02 (-.15,.12)	-.01 (-1.00,1.00)	.14 (-.12,.44)	.00 (-.10,.10)	.38 (-1.00, 1.00)	-.02 (-.38,.32)	.00 (-.08,.08)
Peer pressure	.07 (-.18,.32)	1.00 (-1.00, 1.00)	- .03 (-.08,.02)	-.04 (-.48,-.04)	1.00 (-1.00, 1.00)	-.01 (-.09,.07)	.14 (-.18,.50)	1.00 (-1.00,1.00)	-.05 (-.11,.01)	.03 (-.20,.32)	1.00 (-1.00, 1.00)	-.03 (-.08,.02)
Physical peer victimisation	- .21 (-.57,.16)	-1.00 (-1.00, 1.00)	.01 (-.04,.06)	-.16 (-.78,.53)	-1.00 (-1.00, 1.00)	.03 (-.05,.11)	-.36 (-1.00,.94)	-1.00 (-1.00,1.00)	.00 (-.07,.07)	-.03 (-.41,.52)	-1.00 (-1.00, 1.00)	.02 (-.04,.07)
Social peer victimisation	- .29 (- .64,-.29)	.99 (-1.00, 1.00)	.01 (-.04,.07)	-1.00 (-1.00, 1.00)	.31 (.31, 1.00)	.10 (.01,.18)	-.14 (-.52,.21)	1.00 (-1.00,1.00)	-.04 (-.10,.02)	-.39 (-1.00,-.39)	.49 (-1.00, 1.00)	.02 (-.04,.07)
Verbal peer victimisation	- .03 (-.32,.23)	-1.00 (-1.00,.95)	.03 (-.03,.08)	.11 (-1.00, 1.00)	-.63 (-1.00, 1.00)	.07 (-.02,.15)	-.06 (-.39,.27)	-1.00 (-1.00,1.00)	-.01 (-.08,.05)	.09 (.02,.47)	-1.00 (-1.00,.97)	.02 (-.03,.08)
Cyber peer victimisation	- .24 (-.52,.02)	- .92 (-1.00, 1.00)	.02 (-.04,.07)	.00 (-1.00, 1.00)	-.38 (-1.00,.32)	.04 (-.05,.14)	-.28 (-.62,-.14)	1.00 (-1.00,1.00)	-.01 (-.08,.05)	-.23 (-.54,-.22)	-1.00 (-1.00, 1.00)	.01 (-.04,.07)
Physical peer perpetration	- .27 (-.65,.19)	-1.00 (-1.00, 1.00)	- .02 (-.07,.04)	-.26 (-1.00, 1.00)	-1.00 (-1.00, 1.00)	.00 (-.08,.07)	-.31 (-1.00,1.00)	-1.00 (-1.00,1.00)	-.04 (-.11,.04)	.15 (-1.00, 1.00)	-1.00 (-1.00,-.33)	-.02 (-.07,.04)
Social peer perpetration	- .07 (-.52,.41)	-1.00 (-1.00,.82)	- .03 (-.08,.03)	-.20 (-1.00,.68)	-1.00 (-1.00, 1.00)	.01 (-.08,.10)	.01 (-.54,.82)	-1.00 (-1.00,1.00)	-.05 (-.11,.02)	-.04 (-1.00, 1.00)	-.41 (-1.00, 1.00)	-.03 (-.08,.03)
Verbal peer perpetration	- .03 (-.29,.23)	-1.00 (-1.00, 1.00)	- .01 (-.06,.04)	.03 (-.42,.57)	-1.00 (-1.00, 1.00)	.06 (-.03,.14)	-.08 (-.45,.28)	1.00 (-1.00,1.00)	-.06 (-.13,.00)	.08 (.06,.43)	-1.00 (-1.00, 1.00)	-.01 (-.06,.04)
Cyber peer perpetration	- .12 (-.46,.25)	-1.00 (-1.00, 1.00)	- .03 (-.08,.03)	-.13 (-.83, 1.00)	-1.00 (-1.00, 1.00)	.03 (-.06,.11)	-.17 (-.64,.26)	1.00 (-1.00,1.00)	-.06 (-.13,.01)	-.03 (-.35,.38)	-1.00 (-1.00, 1.00)	-.03 (-.09,.02)

Parental role aspirations	.09 (-.16,.38)	-1.00 (-1.00, 1.00)	- .06 (-.11,- .01)	.09 (-.40,.72)	-1.00 (-1.00, 1.00)	-.02 (-.10,.06)	.10 (.10,.49)	-.52 (-1.00,1.00)	-.09 (-.16,-.03)	.11 (-.16,.45)	-1.00 (NA, 1.00)	-.07 (-.12,-.02)
Occupational role aspirations	.27 (-.03,.58)	1.00 (-1.00, 1.00)	.07 (.02,.12)	.38 (.38, 1.00)	-1.00 (-1.00, 1.00)	.05 (-.03,.13)	.19 (-.20,.55)	1.00 (-1.00,1.00)	.09 (.03,.15)	.25 (.25,.63)	1.00 (-1.00, 1.00)	.07 (.02,.12)
Homecare role aspirations	- .21 (-.47,.01)	1.00 (-1.00, 1.00)	.01 (-.04,.06)	.02 (.02,.17)	-.24 (-1.00, 1.00)	-.02 (-.10,.06)	-.40 (-.81,-.36)	1.00 (-1.00,1.00)	.03 (-.03,.09)	-.29 (-.64,.01)	.44 (-1.00, 1.00)	.01 (-.04,.06)
Importance of relationships	.04 (-.15,.23)	1.00 (-1.00, NA)	.01 (-.03,.06)	.01 (-.23,.16)	1.00 (-1.00, 1.00)	.03 (-.05,.10)	.08 (.08,.33)	.29 (-1.00,1.00)	.00 (-.05,.06)	.02 (-.19,.23)	1.00 (-1.00, 1.00)	.01 (-.04,.06)
Achievement motivation	.08 (-.11,.32)	-1.00 (NA,.14)	.07 (.02,.12)	-.08 (-.71,-.08)	.46 (-1.00, 1.00)	.10 (.02,.18)	.15 (-.13,.45)	-1.00 (-1.00,1.00)	.05 (-.01,.11)	.23 (-.07,.54)	-1.00 (-1.00, 1.00)	.06 (.01,.11)
Purpose in life	.17 (-.06,.42)	- .26 (-1.00, 1.00)	.03 (-.02,.08)	.07 (-.39,.55)	.30 (-1.00, 1.00)	.02 (-.06,.11)	.24 (-.04,.55)	-1.00 (-1.00,1.00)	.04 (-.02,.10)	.12 (-.17,.42)	.01 (-1.00, 1.00)	.03 (-.02,.08)
Importance of democracy and equality	.33 (.15,.54)	1.00 (-1.00, 1.00)	.03 (-.02,.08)	.31 (.31,.80)	.28 (-1.00, 1.00)	.01 (-.07,.10)	.36 (.15,.63)	1.00 (-1.00,1.00)	.04 (-.02,.10)	.33 (.12,.56)	1.00 (-1.00, 1.00)	.03 (-.02,.08)
Environmental concerns	.30 (.13,.50)	1.00 (-1.00, 1.00)	.06 (.01,.11)	.23 (.16,.57)	1.00 (-1.00, 1.00)	.09 (.01,.17)	.38 (.33,.66)	.30 (-1.00,1.00)	.04 (-.02,.10)	.81 (NA, NA)	.01 (-1.00, NA)	-.14 (-1.00,1.00)
Religiosity	.01 (-.23,.24)	.26 (.07,.49)	- .01 (-.06,.04)	-.05 (-.51,.38)	.22 (-.08, 1.00)	.00 (-.08,.09)	.04 (-.14,.34)	.28 (.04,.60)	-.01 (-.07,.05)	-.09 (-.39,.21)	.34 (.10,.83)	-.01 (-.06,.04)
Importance of leisure	.17 (-.06,.40)	1.00 (.94, 1.00)	.00 (-.05,.05)	.19 (-.27,.68)	1.00 (-1.00, 1.00)	-.02 (-.10,.07)	.16 (-.13,.45)	1.00 (.46,1.00)	.02 (-.05,.08)	.15 (-.11,.43)	1.00 (-1.00, 1.00)	.01 (-.04,.06)
Alcohol use	.09 (-.16,.36)	- .38 (-1.00, 1.00)	.02 (-.04,.08)	-.16 (-.93,.38)	.03 (-1.00, 1.00)	.12 (.03,.22)	.19 (-.09,.50)	-1.00 (-1.00,1.00)	-.05 (-.12,.03)	.00 (-.33,.29)	.00 (-1.00, 1.00)	.02 (-.04,.08)
Ever smoked	.03 (-.16,.25)	-1.00 (-1.00,- .44)	- .01 (-.06,.04)	-.13 (-.48,-.13)	-1.00 (-1.00, 1.00)	.00 (-.09,.08)	.16 (-.08,.50)	-1.00 (-1.00,-.78)	-.02 (-.08,.05)	-.01 (-.23,.23)	-1.00 (-1.00, 1.00)	-.02 (-.07,.03)
Ever vaped	- .33 (- .64,-.33)	- .43 (-1.00, 1.00)	- .01 (-.07,.04)	-.33 (-.89,-.33)	-1.00 (-1.00,.97)	.01 (-.08,.10)	-.37 (-.80,-.01)	-.18 (-1.00,1.00)	-.03 (-.09,.04)	-.25 (-.64,-.25)	-.54 (-1.00, 1.00)	-.01 (-.07,.04)
Cognitive enhancers	.23 (-.04,.55)	-1.00 (-1.00, 1.00)	- .02 (-.07,.03)	.17 (-.33,.83)	-1.00 (-1.00, 1.00)	.00 (-.09,.09)	.28 (.28,.72)	-.61 (-1.00,1.00)	-.03 (-.10,.04)	.27 (-.10,.66)	-.48 (-1.00,-.48)	-.02 (-.07,.03)
Big 5 Agreeableness	.03 (-.20,.30)	1.00 (-1.00, 1.00)	.00 (-.05,.05)	.04 (-.47,.49)	1.00 (-1.00, 1.00)	.01 (-.07,.09)	.03 (-.37,.42)	1.00 (.09,1.00)	-.01 (-.07,.05)	.02 (-.27,.30)	1.00 (-1.00, 1.00)	.00 (-.05,.05)
Big 5 Conscientiousness	.13 (-.10,.35)	1.00 (-1.00, 1.00)	.07 (.03,.12)	-.01 (-.48,.11)	1.00 (-1.00, 1.00)	.08 (.00,.16)	.23 (-.06,.54)	1.00 (-1.00,1.00)	.07 (.01,.14)	.11 (-.13,.34)	1.00 (-1.00, 1.00)	.07 (.02,.12)
Big 5 Extraversion	- .01 (-.23,.17)	1.00 (-1.00, 1.00)	.07 (.02,.11)	-.13 (-.67,-.12)	1.00 (-1.00, 1.00)	.05 (-.03,.13)	.06 (-.21,.13)	1.00 (-.28,1.00)	.08 (.02,.14)	-.04 (-.28,.16)	1.00 (-1.00, 1.00)	.07 (.02,.12)
Big 5 Neuroticism	.10 (-.15,.38)	-1.00 (-1.00, 1.00)	- .03 (-.08,.02)	-.04 (-.73,-.04)	-.39 (-1.00, 1.00)	.01 (-.08,.09)	.15 (-.14,.48)	-1.00 (-1.00,1.00)	-.05 (-.11,.01)	.10 (.07,.35)	-.41 (-1.00, 1.00)	-.02 (-.07,.03)
Big 5 Openness	.07 (-.15,.27)	1.00 (-1.00, 1.00)	.05 (.00,.10)	-.10 (-.63,-.10)	1.00 (-1.00, 1.00)	.09 (.01,.17)	.16 (.08,.43)	1.00 (-1.00,1.00)	.03 (-.03,.09)	.07 (-.18,.29)	1.00 (-1.00, 1.00)	.06 (.01,.11)
Self-control	.23 (.03,.46)	-1.00 (-1.00, 1.00)	.00 (-.05,.05)	.25 (-.14,.77)	-.99 (-1.00, 1.00)	-.01 (-.09,.07)	.22 (-.02,.52)	-1.00 (-1.00,1.00)	.00 (-.06,.06)	.22 (.00,.48)	-1.00 (-1.00, 1.00)	.00 (-.06,.05)
Not planning for the future	- .25 (- .62,-.25)	- .65 (-1.00, 1.00)	- .02 (-.07,.03)	-.19 (-.75,.31)	-1.00 (-1.00, 1.00)	-.02 (-.10,.06)	-.36 (-1.00,.17)	-.20 (-1.00,1.00)	-.02 (-.09,.04)	-.35 (-.77,-.22)	.94 (-1.00, 1.00)	-.03 (-.08,.02)
Ambition	.18 (-.06,.42)	1.00 (-1.00, 1.00)	.07 (.02,.12)	.17 (-.30,.62)	1.00 (-1.00, 1.00)	.06 (-.02,.14)	.19 (-.13,.50)	1.00 (-1.00,1.00)	.07 (.01,.14)	.19 (.07,.48)	1.00 (-1.00, 1.00)	.07 (.02,.12)

Physical activity	.10 (-.12,.31)	1.00 (-1.00, 1.00)	.06 (.01,.10)	.36 (.36,.92)	-1.00 (-1.00, 1.00)	-.02 (-.10,.06)	-.03 (-.38,.23)	1.00 (.30,1.00)	.10 (.04,.16)	.00 (-.29,.25)	1.00 (-1.00, 1.00)	.05 (.00,.10)
Health behaviours	.26 (.07,.47)	.67 (.67, 1.00)	.03 (-.02,.08)	.35 (.35,.76)	1.00 (-1.00, 1.00)	-.01 (-.10,.07)	.25 (.00,.52)	.44 (NA,1.00)	.06 (-.01,.12)	.21 (-.03,.45)	.66 (-.04, 1.00)	.03 (-.02,.08)
Risk taking behaviour	- .06 (-.27,.14)	1.00 (-1.00, NA)	.03 (-.02,.08)	-.96 (NA, NA)	.32 (NA, NA)	.43 (-.99, 1.00)	.06 (-.19,.32)	1.00 (-1.00,1.00)	.00 (-.07,.06)	-.15 (-.41,.08)	1.00 (-1.00, 1.00)	.03 (-.02,.08)
Risky sexual behaviour	- .13 (-.30,.03)	-1.00 (-1.00, 1.00)	.00 (-.05,.05)	-.28 (-.44,-.28)	.27 (-1.00, 1.00)	.00 (-.08,.08)	-.01 (-.22,.20)	-1.00 (-1.00,-.21)	-.01 (-.07,.05)	-.05 (-.27,.15)	-.97 (-1.00,-.97)	-.01 (-.06,.04)
Media use	.05 (-.10,.23)	-1.00 (-1.00, 1.00)	- .02 (-.07,.03)	-.55 (NA, NA)	-.08 (-1.00, 1.00)	-.02 (-1.00,.98)	.06 (-.14,.31)	-1.00 (-1.00,1.00)	-.03 (-.09,.03)	.02 (-.14,.21)	-1.00 (-1.00, 1.00)	-.03 (-.08,.02)
Volunteering	.19 (-.02,.41)	1.00 (-1.00, 1.00)	.03 (-.02,.08)	.23 (-.10,.66)	1.00 (-1.00, 1.00)	-.03 (-.11,.06)	.18 (-.09,.46)	1.00 (-1.00,1.00)	.06 (.00,.12)	.11 (-.14,.38)	1.00 (-1.00, 1.00)	.02 (-.03,.07)
Internet dating	.14 (-.13,.42)	.76 (- .96, 1.00)	.04 (-.01,.09)	.32 (.32,.88)	-1.00 (-1.00, 1.00)	.02 (-.06,.10)	.04 (-.33,.38)	1.00 (-1.00,1.00)	.05 (-.01,.11)	.01 (-.35,.07)	.51 (-1.00, 1.00)	.03 (-.02,.08)
Internet dating	.07 (.07,.33)	1.00 (-1.00, 1.00)	.05 (.00,.09)	.29 (.29,.88)	-1.00 (-1.00, 1.00)	.02 (-.06,.10)	-.05 (-.43,.27)	1.00 (-1.00,1.00)	.06 (.00,.12)	-.06 (-.43,.30)	.42 (-1.00, 1.00)	.04 (-.01,.09)
Financial literacy: knowledge of products	.08 (-.15,.31)	-1.00 (-1.00, 1.00)	.03 (-.02,.07)	.06 (-.33,.44)	1.00 (-1.00, 1.00)	.06 (-.02,.14)	.06 (-.26,.40)	-1.00 (-1.00,1.00)	.01 (-.05,.07)	.05 (.05,.35)	-.70 (-1.00, 1.00)	.02 (-.03,.07)
Financial attitudes and behaviour	.15 (-.10,.41)	.85 (-1.00, 1.00)	.03 (-.02,.07)	.03 (-.52,.49)	1.00 (-1.00, 1.00)	.02 (-.06,.09)	.22 (-.01,.60)	-1.00 (-1.00,1.00)	.03 (-.03,.09)	.16 (-.09,.47)	-1.00 (-1.00, 1.00)	.02 (-.03,.07)
	Model fitting results											
	Functional outcome: planned educational level											
	Whole sample			Males only			Females only			Same-sex MZ and DZ twins		
Trait	rA	rC	rE	rA	rC	rE	rA	rC	rE	rA	rC	rE
Daily hassles	- .06 (-.31,.12)	.63 (-1.00, 1.00)	.01 (-.05,.07)	-.22 (-.56,-.22)	-.28 (-1.00, 1.00)	.01 (-.08,.11)	-.04 (-.46,-.04)	1.00 (-1.00,1.00)	.01 (-.07,.09)	-.04 (-.38,.18)	.65 (-1.00, 1.00)	.02 (-.05,.08)
CHAOS	- .26 (- .90,-.26)	-1.00 (-1.00, 1.00)	.03 (-.03,.09)	-.22 (-.71,.23)	-1.00 (-1.00, 1.00)	.06 (-.04,.16)	-.45 (-1.00,-.45)	-1.00 (-1.00,1.00)	.01 (-.06,.09)	-.15 (-1.00, 1.00)	-1.00 (-1.00, 1.00)	.03 (-.03,.10)
Childhood experiences	- .09 (-.30,.11)	-1.00 (-1.00, 1.00)	.01 (-.06,.08)	-.01 (-.31,.38)	-1.00 (-1.00, 1.00)	-.03 (-.14,.08)	-.20 (-.52,.08)	1.00 (-1.00,1.00)	.04 (-.05,.13)	-.12 (-.42,.15)	1.00 (-1.00, 1.00)	.02 (-.06,.09)
Poor sleep quality	- .20 (- .47,-.20)	-1.00 (NA, 1.00)	.02 (-.04,.08)	-.27 (-1.00,.12)	-1.00 (-1.00, 1.00)	.04 (-.06,.14)	-.18 (-.63,-.14)	1.00 (-1.00,1.00)	.01 (-.07,.09)	-.27 (-1.00,.20)	-1.00 (-1.00, 1.00)	.01 (-.06,.08)
Marriage hopes	.28 (.28,.69)	1.00 (-1.00, 1.00)	- .04 (-.10,.02)	1.00 (-1.00, 1.00)	-1.00 (-1.00, 1.00)	-.03 (-.12,.07)	.21 (.21,.59)	1.00 (-1.00,1.00)	-.04 (-.12,.03)	.14 (.14,.76)	1.00 (-1.00, 1.00)	-.04 (-.10,.02)
Marriage worries	- .01 (-.19,.23)	-1.00 (-1.00, 1.00)	.00 (-.06,.06)	-.04 (-.28,.17)	-.96 (-1.00, 1.00)	-.01 (-.10,.08)	.04 (-.17,.42)	-1.00 (-1.00,1.00)	.01 (-.07,.08)	.00 (-.30,.32)	-1.00 (NA, 1.00)	-.01 (-.07,.05)
Quality of relationship with twin	- .22 (-.47,.01)	1.00 (.33, 1.00)	- .05 (-.11,.02)	-.44 (-.97,-.05)	1.00 (1.00, 1.00)	-.04 (-.14,.06)	-.09 (-.36,.20)	1.00 (-1.00,1.00)	-.05 (-.14,.03)	-.19 (-.49,.12)	1.00 (-1.00, 1.00)	-.05 (-.12,.01)
Quality of relationship with mother	- .05 (-.24,.07)	1.00 (-1.00, NA)	.07 (.01,.13)	.00 (-.27,.18)	-.72 (-1.00, 1.00)	.02 (-.08,.12)	-.10 (-.38,-.10)	1.00 (-1.00,1.00)	.09 (.01,.16)	-.10 (-.37,-.10)	1.00 (-1.00, 1.00)	.07 (.01,.14)
Quality of relationship with father	- .08 (-.28,.15)	1.00 (-1.00, 1.00)	.03 (-.04,.09)	-.23 (-.79,-.19)	1.00 (.22, 1.00)	.04 (-.07,.15)	.03 (-.28,.34)	1.00 (-1.00,1.00)	.01 (-.07,.10)	-.05 (-.34,.24)	1.00 (-1.00, 1.00)	.03 (-.03,.10)

Number of relationships	- .18 (- .28,-.06)	.56 (-1.00, 1.00)	.02 (-.04,.07)	-.15 (-.22,.09)	-.81 (-1.00, 1.00)	-.02 (-.11,.08)	-.20 (-.28,-.03)	-.84 (-1.00,1.00)	.04 (-.03,.11)	-.13 (-.34,-.06)	-1.00 (-1.00, 1.00)	.00 (-.06,.06)
Longest relationship	- .15 (-.28,.01)	- .92 (-1.00, 1.00)	- .01 (-.07,.05)	-.14 (-.50,-.14)	.94 (-1.00, 1.00)	.05 (-.05,.14)	-.17 (-.34,.07)	.69 (-1.00,1.00)	-.05 (-.12,.03)	-.13 (-.37,.09)	-.27 (-1.00, 1.00)	-.03 (-.10,.03)
Partner violence	- .16 (-.16,.19)	-1.00 (-1.00, 1.00)	- .03 (-.09,.03)	-.42 (-1.00,-.42)	-1.00 (-1.00, 1.00)	.07 (-.03,.16)	-.01 (-.01,.65)	-1.00 (-1.00,1.00)	-.07 (-.15,.00)	-.11 (-.22,.35)	-1.00 (-1.00, 1.00)	-.04 (-.10,.02)
Contact with mother	- .35 (- .61,-.35)	-1.00 (-1.00, 1.00)	- .11 (-.22,- .01)	-.34 (-1.00,-.02)	1.00 (-1.00, NA)	-.15 (-.33,.03)	-.36 (-.66,.01)	-1.00 (-1.00,-.21)	-.10 (-.23,.04)	-.29 (-.64,-.14)	-1.00 (-1.00,-1.00)	-.15 (-.26,-.03)
Communication with mother	- .14 (-.54,.08)	1.00 (-1.00, 1.00)	- .02 (-.13,.09)	.00 (-.72,.65)	1.00 (-1.00, 1.00)	-.06 (-.24,.12)	-.21 (-.77,.23)	1.00 (-.18,1.00)	.00 (-.13,.13)	-.21 (-.67,.20)	1.00 (-.98, 1.00)	-.04 (-.15,.08)
Contact with father	- .32 (-1.00, 1.00)	-1.00 (-1.00,- .26)	- .04 (-.15,.06)	-.61 (-1.00, 1.00)	1.00 (-1.00, 1.00)	-.12 (-.27,.03)	-.31 (-1.00,1.00)	-1.00 (-1.00,1.00)	.02 (-.12,.15)	-.30 (-1.00, 1.00)	-1.00 (-1.00, 1.00)	-.06 (-.17,.05)
Communication with father	.53 (.01, 1.00)	-1.00 (-1.00, 1.00)	- .06 (-.16,.04)	-.09 (-1.00, 1.00)	1.00 (-1.00, 1.00)	-.06 (-.23,.11)	1.00 (.57,1.00)	-1.00 (-1.00,-.91)	-.05 (-.16,.06)	1.00 (.36, 1.00)	-1.00 (-1.00,-.94)	-.07 (-.16,.03)
Peer pressure	.02 (-.13,.16)	- .98 (-1.00, 1.00)	- .03 (-.08,.02)	-.10 (-.39,.15)	-.50 (-1.00, 1.00)	.02 (-.08,.11)	.12 (-.13,.35)	1.00 (-.95,1.00)	-.06 (-.12,.01)	.02 (-.28,.23)	1.00 (-1.00, 1.00)	-.02 (-.08,.04)
Physical peer victimisation	- .04 (-.12,.29)	.02 (-1.00, 1.00)	- .04 (-.09,.02)	-.12 (-.44,.72)	-1.00 (-1.00, 1.00)	.00 (-.10,.09)	.08 (-1.00,1.00)	-.90 (-1.00,1.00)	-.07 (-.15,.01)	.06 (.06,.78)	-1.00 (-1.00, 1.00)	-.06 (-.12,.01)
Social peer victimisation	.03 (-.21,.44)	-1.00 (-1.00, 1.00)	- .01 (-.07,.05)	1.00 (-1.00, 1.00)	-1.00 (-1.00, 1.00)	-.02 (-.12,.09)	-.08 (-.25,.32)	-1.00 (-1.00,1.00)	-.01 (-.08,.06)	.27 (-.11, 1.00)	-1.00 (-1.00,-.15)	-.02 (-.09,.04)
Verbal peer victimisation	.05 (-.15,.41)	-1.00 (-1.00, 1.00)	.00 (-.06,.06)	.24 (-1.00, 1.00)	-1.00 (-1.00, 1.00)	-.01 (-.11,.09)	-.01 (-.17,.36)	-.99 (-1.00,1.00)	.00 (-.07,.08)	.21 (-.09,.68)	-1.00 (-1.00, 1.00)	-.02 (-.09,.05)
Cyber peer victimisation	- .17 (-.31,.10)	-1.00 (-1.00, 1.00)	.01 (-.05,.07)	.88 (-1.00, 1.00)	-1.00 (-1.00,-1.00)	-.01 (-.11,.09)	-.18 (-.34,.00)	-.99 (-1.00,1.00)	.00 (-.08,.08)	-.09 (-.09,.27)	-1.00 (-1.00, 1.00)	.01 (-.06,.07)
Physical peer perpetration	- .16 (- .28,-.16)	.95 (-1.00, 1.00)	- .07 (-.13,.00)	-.14 (-1.00, 1.00)	-1.00 (-1.00, 1.00)	-.12 (-.22,-.02)	-.33 (-1.00,1.00)	1.00 (-1.00,1.00)	-.01 (-.09,.08)	.31 (-1.00, 1.00)	-1.00 (-1.00, 1.00)	-.06 (-.13,.01)
Social peer perpetration	- .12 (-.12,.82)	-1.00 (-1.00, 1.00)	.00 (-.06,.06)	.01 (-1.00, 1.00)	-1.00 (-1.00, 1.00)	-.04 (-.15,.07)	.89 (-1.00,1.00)	-1.00 (-1.00,1.00)	.00 (-.08,.10)	.24 (-1.00, 1.00)	-1.00 (-1.00, 1.00)	-.02 (-.08,.04)
Verbal peer perpetration	- .12 (-.24,.05)	- .98 (-1.00, 1.00)	.04 (-.02,.10)	-.08 (-.31,.41)	-.58 (-1.00, 1.00)	.02 (-.08,.11)	-.16 (-.39,-.10)	.98 (-1.00,1.00)	.06 (-.02,.13)	-.10 (-.32,.18)	-.59 (-1.00, 1.00)	.02 (-.04,.08)
Cyber peer perpetration	- .26 (- .40,-.06)	- .78 (-1.00, 1.00)	.00 (-.06,.06)	-.11 (-1.00, 1.00)	-1.00 (NA, 1.00)	-.02 (-.13,.08)	-.32 (-.48,-.10)	.00 (NA,1.00)	.01 (-.07,.08)	-.18 (-.45,.21)	-.65 (-1.00, 1.00)	-.01 (-.07,.06)
Parental role aspirations	.07 (-.10,.21)	- .58 (-1.00, 1.00)	- .07 (-.13,- .02)	.16 (-.16,.47)	.09 (-1.00, 1.00)	-.14 (-.23,-.04)	.01 (-.29,.30)	1.00 (-1.00,1.00)	-.03 (-.11,.04)	.04 (-.20,.24)	1.00 (-1.00, 1.00)	-.08 (-.14,-.02)
Occupational role aspirations	.29 (.14,.56)	- .49 (-1.00, 1.00)	.03 (-.03,.09)	.26 (-.03, 1.00)	-.98 (-1.00, 1.00)	.06 (-.04,.15)	.30 (.05,.64)	-.06 (-1.00,1.00)	.02 (-.05,.09)	.27 (.27,.58)	1.00 (-1.00, 1.00)	.01 (-.05,.08)
Homecare role aspirations	- .15 (- .37,-.05)	1.00 (- .95, 1.00)	.03 (-.03,.09)	-.03 (-.32,.17)	-.57 (-1.00, 1.00)	.01 (-.09,.10)	-.25 (-.66,-.08)	1.00 (-1.00,1.00)	.04 (-.03,.12)	-.27 (-.63,.00)	1.00 (-1.00, 1.00)	.04 (-.03,.10)
Importance of relationships	.08 (-.04,.18)	- .85 (-1.00, 1.00)	- .04 (-.10,.02)	.11 (-.05,.36)	-.99 (-1.00, 1.00)	-.03 (-.12,.06)	.05 (-.23,.05)	1.00 (-1.00,1.00)	-.04 (-.12,.03)	.03 (-.21,.03)	1.00 (-1.00, 1.00)	-.05 (-.11,.01)
Achievement motivation	.04 (-.14,.17)	- .51 (-1.00, 1.00)	.06 (.00,.11)	.22 (-.12,.72)	-1.00 (-1.00, NA)	-.03 (-.12,.07)	-.05 (-.35,.09)	-.26 (-1.00,1.00)	.11 (.04,.18)	-.08 (-.45,.16)	1.00 (-1.00, 1.00)	.05 (-.01,.11)
Purpose in life	.04 (-.20,.20)	1.00 (-1.00, 1.00)	.05 (-.01,.11)	.07 (-.40,.37)	1.00 (-1.00, 1.00)	.07 (-.03,.16)	.02 (-.31,.21)	1.00 (-1.00,1.00)	.04 (-.04,.11)	-.08 (-.42,.06)	1.00 (-.05, 1.00)	.05 (-.01,.11)

Importance of democracy and equality	.31 (.22,.49)	.95 (-1.00, 1.00)	.02 (-.04,.08)	.18 (-.12,.53)	-1.00 (-1.00, 1.00)	.06 (-.04,.15)	.41 (.29,.73)	-.01 (-1.00,1.00)	-.01 (-.09,.06)	.32 (.21,.55)	-.44 (-1.00, 1.00)	.01 (-.06,.07)
Environmental concerns	.41 (.31,.59)	-1.00 (-1.00, 1.00)	.00 (-.06,.05)	.37 (.21,.62)	.23 (-1.00, 1.00)	.01 (-.08,.11)	.46 (.29,.76)	-1.00 (-1.00,1.00)	-.02 (-.09,.06)	.38 (.38,.60)	1.00 (-1.00, 1.00)	-.01 (-.07,.05)
Religiosity	.11 (-.14,.35)	1.00 (-1.00, 1.00)	- .03 (-.09,.04)	.20 (-.20,.65)	1.00 (-1.00, 1.00)	-.09 (-.19,.01)	.06 (-.25,.36)	1.00 (-1.00,1.00)	.01 (-.07,.09)	.03 (-.29,.34)	1.00 (-1.00, 1.00)	-.02 (-.09,.04)
Importance of leisure	.27 (.11,.43)	1.00 (-1.00, 1.00)	- .01 (-.07,.05)	.23 (-.28,.57)	1.00 (-1.00, 1.00)	-.03 (-.13,.08)	.29 (.29,.48)	1.00 (-1.00,1.00)	.00 (-.08,.08)	.16 (-.16,.37)	1.00 (-1.00, 1.00)	.00 (-.07,.07)
Alcohol use	.19 (.13,.47)	-1.00 (-1.00, 1.00)	- .05 (-.13,.02)	.23 (-.31,.98)	-1.00 (-1.00, 1.00)	-.03 (-.16,.11)	.14 (.14,.49)	-1.00 (-1.00,1.00)	-.06 (-.15,.03)	.27 (-.04,.67)	-1.00 (-1.00, 1.00)	-.07 (-.15,.01)
Ever smoked	- .11 (-.23,.04)	- .94 (-1.00, 1.00)	.00 (-.07,.06)	-.06 (-.26,.25)	-.85 (NA, 1.00)	-.04 (-.14,.07)	-.13 (-.35,.02)	-1.00 (-1.00,1.00)	.01 (-.07,.09)	-.11 (-.27,-.03)	-1.00 (-1.00, 1.00)	-.01 (-.07,.06)
Ever vaped	- .29 (- .53,-.08)	-1.00 (-1.00, 1.00)	.00 (-.06,.06)	-.22 (-.59,-.20)	-1.00 (NA, 1.00)	-.02 (-.12,.09)	-.36 (-.78,-.26)	1.00 (-1.00,1.00)	.01 (-.07,.09)	-.25 (-.60,.07)	-1.00 (-1.00, 1.00)	.00 (-.07,.07)
Cognitive enhancers	.06 (-.16,.27)	1.00 (-1.00, 1.00)	- .02 (-.08,.04)	.10 (-.23,.59)	.32 (-1.00, 1.00)	-.02 (-.12,.09)	-.02 (-.41,-.02)	1.00 (-1.00,1.00)	-.01 (-.09,.06)	-.08 (-.52,.28)	1.00 (-1.00, 1.00)	-.02 (-.08,.05)
Big 5 Agreeableness	.07 (-.06,.25)	.20 (-1.00, 1.00)	- .03 (-.09,.03)	.07 (-.17,.43)	.63 (-1.00, 1.00)	-.04 (-.14,.06)	.07 (-.11,.38)	.32 (-1.00,1.00)	-.03 (-.10,.04)	.08 (-.08,.32)	-.82 (-1.00, 1.00)	-.06 (-.12,.01)
Big 5 Conscientiousness	.07 (-.06,.19)	.43 (-1.00, 1.00)	.10 (.04,.16)	.02 (-.21,.28)	-.95 (-1.00, 1.00)	.09 (-.01,.19)	.10 (-.09,.28)	.09 (-1.00,1.00)	.11 (.03,.18)	.08 (-.11,.22)	.81 (-1.00, 1.00)	.09 (.03,.16)
Big 5 Extraversion	.16 (.02,.26)	.57 (-1.00, 1.00)	- .04 (-.10,.02)	.13 (-.13,.31)	1.00 (-1.00, 1.00)	-.05 (-.14,.05)	.19 (.03,.31)	.99 (-1.00,1.00)	-.04 (-.11,.04)	.11 (-.11,.20)	1.00 (-1.00, 1.00)	-.04 (-.10,.03)
Big 5 Neuroticism	- .09 (-.30,.08)	- .93 (-1.00, 1.00)	- .01 (-.07,.05)	-.28 (-1.00,-.08)	1.00 (NA, 1.00)	.05 (-.06,.16)	-.03 (-.25,.18)	1.00 (-1.00,1.00)	-.04 (-.12,.03)	-.14 (-.50,.12)	1.00 (-1.00, 1.00)	-.01 (-.08,.06)
Big 5 Openness	.11 (-.03,.21)	- .22 (-1.00, 1.00)	.00 (-.06,.06)	.09 (-.27,.28)	.31 (-1.00, 1.00)	.01 (-.09,.10)	.12 (-.06,.27)	.94 (-1.00,1.00)	.00 (-.08,.07)	.07 (-.21,.07)	1.00 (.87, 1.00)	.00 (-.07,.06)
Self-control	.11 (.00,.25)	- .97 (-1.00, 1.00)	.03 (-.03,.09)	.12 (-.13,.41)	.69 (-1.00, 1.00)	.02 (-.08,.12)	.10 (-.03,.34)	.56 (-1.00,1.00)	.03 (-.04,.11)	.11 (-.03,.41)	-1.00 (NA, 1.00)	.03 (-.04,.09)
Not planning for the future	- .29 (- .64,-.29)	1.00 (-1.00, 1.00)	- .04 (-.10,.02)	-.27 (-.70,-.07)	-.99 (-1.00, 1.00)	-.02 (-.11,.08)	-.37 (-1.00,.10)	1.00 (-1.00,1.00)	-.05 (-.13,.03)	-.30 (-.71,-.04)	-.91 (-1.00, 1.00)	-.05 (-.11,.02)
Ambition	.25 (.12,.41)	- .69 (-1.00, 1.00)	.05 (-.01,.11)	.34 (.15,.90)	-1.00 (-1.00, 1.00)	-.02 (-.12,.08)	.20 (-.08,.39)	1.00 (-1.00,1.00)	.09 (.01,.16)	.25 (.02,.45)	.74 (-1.00, 1.00)	.03 (-.03,.10)
Physical activity	.22 (.05,.33)	.34 (-1.00, 1.00)	- .01 (-.07,.04)	.24 (.06,.68)	-.67 (-1.00, 1.00)	-.08 (-.17,.01)	.13 (-.19,.33)	1.00 (-1.00,1.00)	.04 (-.03,.11)	.11 (-.18,.32)	1.00 (-1.00, 1.00)	.00 (-.06,.06)
Health behaviours	.32 (.27,.47)	1.00 (-1.00, 1.00)	- .04 (-.10,.02)	.39 (.20,.63)	.50 (-1.00, 1.00)	-.06 (-.15,.04)	.27 (.12,.49)	1.00 (-1.00,1.00)	-.03 (-.10,.05)	.33 (.15,.55)	1.00 (-1.00, 1.00)	-.03 (-.09,.04)
Risk taking behaviour	.12 (-.01,.25)	- .69 (-1.00, 1.00)	- .10 (-.16,- .04)	.11 (.09,.51)	-1.00 (-1.00, 1.00)	-.11 (-.21,.00)	.17 (-.02,.33)	-.05 (-1.00,1.00)	-.10 (-.18,-.02)	.10 (.10,.28)	1.00 (-1.00, 1.00)	-.10 (-.17,-.04)
Risky sexual behaviour	- .11 (-.21,.02)	- .96 (-1.00, 1.00)	- .03 (-.08,.03)	-.10 (-.27,.15)	-.94 (-1.00, 1.00)	-.06 (-.16,.04)	-.12 (-.29,-.01)	-1.00 (-1.00,1.00)	-.01 (-.08,.07)	-.04 (-.07,.08)	-1.00 (-1.00, 1.00)	-.03 (-.10,.03)
Media use	- .04 (-.13,.05)	- .77 (-1.00, 1.00)	- .02 (-.08,.03)	.03 (-.11,.23)	.39 (-1.00, 1.00)	-.03 (-.12,.07)	-.10 (-.26,.03)	.29 (-1.00,1.00)	-.02 (-.10,.06)	-.06 (-.17,.09)	.95 (-.99, 1.00)	-.03 (-.09,.04)
Volunteering	.25 (.16,.48)	- .99 (-1.00, 1.00)	.00 (-.06,.05)	.25 (.08,.68)	-.32 (-1.00, 1.00)	-.06 (-.15,.03)	.26 (.12,.56)	1.00 (-1.00,1.00)	.03 (-.04,.09)	.32 (.13,.65)	-1.00 (-1.00, 1.00)	-.01 (-.07,.05)

Internet dating	.03 (-.14,.21)	1.00 (-1.00, 1.00)	.02 (-.04,.07)	.00 (-.39,.33)	1.00 (-1.00, 1.00)	-.03 (-.12,.06)	.06 (-.24,.31)	.70 (-1.00,1.00)	.04 (-.03,.11)	.01 (-.31,.32)	-1.00 (-1.00, 1.00)	.01 (-.05,.07)
Internet dating	.01 (-.15,.18)	.33 (NA, 1.00)	.02 (-.04,.07)	.00 (-.30,.42)	-.98 (-1.00, 1.00)	-.03 (-.12,.06)	.03 (-.24,.24)	-.57 (-1.00,1.00)	.04 (-.03,.11)	.03 (.03,.35)	-1.00 (-1.00, 1.00)	.01 (-.06,.07)
Financial literacy: knowledge of products	.00 (-.16,.11)	.42 (-1.00, 1.00)	.03 (-.02,.09)	.06 (-.32,.19)	1.00 (-1.00, 1.00)	.00 (-.09,.10)	-.08 (-.33,.10)	1.00 (-1.00,1.00)	.05 (-.02,.13)	-.02 (-.29,.22)	1.00 (-1.00, 1.00)	.04 (-.02,.11)
Financial attitudes and behaviour	.09 (-.06,.22)	.40 (-1.00, 1.00)	.05 (-.01,.11)	.13 (-.18,.41)	.83 (-1.00, 1.00)	.03 (-.06,.12)	.07 (-.15,.25)	-.64 (-1.00,1.00)	.06 (-.01,.14)	.07 (-.15,.27)	.90 (-1.00, 1.00)	.06 (.00,.12)

Note. A= genetic influences, C= shared environmental influences, E= unique environmental influences. Scores were corrected for mean age and sex differences (see Methods).

Table S14. Univariate GREML estimates

	GREML results							
	Family-based				Unrelated samples			
Trait	SNP-h^2	SE	LogL	N	SNP-h^2	SE	LogL	N
Daily hassles	.05	.07	-2466.48	5115	.17	.10	-1859.61	3770
CHAOS	.12	.07	-271.92	5705	.20	.09	-2026.25	4099
Childhood experiences	.15	.07	-2372.97	5116	.17	.10	-1867.48	3777
Poor sleep quality	.13	.07	-2388.89	5026	.07	.10	-1812.23	3713
Marriage hopes	.03	.06	-2784.46	5622	.13	.09	-2042.02	4069
Marriage worries	.10	.07	-2798.85	5621	.06	.09	-2044.96	4068
Quality of relationship with twin	.11	.08	-2522.54	5533	.26	.09	-2023.64	4026
Quality of relationship with mother	.11	.07	-2646.08	5484	.09	.09	-1991.28	3998
Quality of relationship with father	.10	.07	-2473.04	5381	.17	.09	-1967.84	3937
Number of relationships	.14	.07	-2671.69	5620	.23	.09	-1954.13	4069
Longest relationship	.05	.08	-2327.97	4803	.05	.10	-1803.40	3672
Partner violence	.10	.07	-270.17	5453	.19	.09	-203.17	3992
Contact with mother	.10	.15	-113.90	2393	.08	.18	-976.90	2005
Communication with mother	.14	.14	-1187.64	2448	.11	.17	-1035.21	2049
Contact with father	.00	.14	-1166.09	2614	.00	.17	-1006.91	2135
Communication with father	-.08	.12	-1206.95	2835	.00	.15	-1102.89	2313
Peer pressure	.02	.06	-281.79	5568	.00	.09	-2081.99	4045
Physical peer victimisation	.06	.07	-249.71	4966	.07	.10	-1814.18	3691
Social peer victimisation	.17	.07	-2462.50	4966	.16	.10	-1836.95	3691
Verbal peer victimisation	.14	.07	-2556.27	4964	.10	.10	-1927.58	3690

Cyber peer victimisation	.16	.07	-2488.27	4963	.18	.10	-1932.56	3689
Physical peer perpetration	.00	.07	-2533.76	4965	.04	.10	-1957.28	3690
Social peer perpetration	.12	.07	-2313.53	4964	.09	.10	-1661.46	3689
Verbal peer perpetration	.00	.07	-2566.01	4964	.00	.10	-1884.04	3689
Cyber peer perpetration	.16	.08	-253.70	4964	.25	.10	-199.04	3689
Parental role aspirations	.04	.07	-2651.00	5121	.00	.09	-1913.54	3781
Occupational role aspirations	.00	.07	-2545.07	5124	.00	.10	-184.20	3782
Homecare role aspirations	.10	.07	-253.68	5124	.13	.10	-1841.62	3782
Importance of relationships	.06	.06	-2795.22	5670	.08	.09	-2006.33	4112
Achievement motivation	.04	.07	-2838.80	5669	.12	.09	-2037.22	4111
Purpose in life	.09	.07	-2781.45	5667	.14	.09	-207.67	4110
Importance of democracy and equality	.05	.07	-2813.41	5657	.07	.09	-2046.04	4105
Environmental concerns	.19	.07	-2788.12	5663	.24	.09	-2054.45	4109
Religiosity	.00	.06	-215.37	5571	.00	.09	-1688.22	4047
Importance of leisure	.20	.07	-2463.74	5026	.26	.10	-1842.84	3714
Alcohol use	.01	.08	-214.11	4635	.05	.10	-1616.28	3503
Ever smoked	.04	.07	-2466.61	5043	.00	.09	-1842.05	3728
Ever vaped	.09	.07	-2427.37	4985	.07	.10	-1914.77	3730
Cognitive enhancers	.05	.07	-275.32	5508	.08	.10	-184.88	3703
Big 5 Agreeableness	.12	.08	-2521.59	5040	.00	.09	-1998.17	4043
Big 5 Conscientiousness	.00	.06	-2699.13	5500	.01	.09	-197.73	4038

Big 5 Extraversion	.13	.07	-2709.25	5528	.21	.09	-1934.41	4059
Big 5 Neuroticism	.06	.07	-2702.47	5534	.05	.09	-2005.99	4063
Big 5 Openness	.16	.07	-2723.37	5519	.25	.09	-1985.46	4053
Self-control	.02	.06	-2716.50	5498	.00	.08	-1944.17	4037
Not planning for the future	.06	.07	-2678.72	5493	.19	.09	-1946.78	4033
Ambition	.03	.07	-2572.27	5117	.11	.10	-1878.06	3771
Physical activity	.04	.07	-2754.09	5548	.00	.09	-2033.70	4031
Health behaviours	.12	.07	-2734.04	5571	.11	.09	-2047.28	4039
Risk taking behaviour	.05	.07	-2624.36	5493	.08	.09	-1981.79	4034
Risky sexual behaviour	.17	.07	-2606.46	5397	.22	.09	-1963.86	3955
Media use	.13	.07	-2625.49	5526	.04	.09	-1957.01	4022
Volunteering	.06	.07	-2737.51	5577	.17	.09	-2003.49	4050
Internet dating (scale)	.03	.07	-2711.68	5506	.00	.09	-1984.05	4013
Internet dating (y/n)	.05	.07	-2755.54	5524	.00	.09	-2009.21	4020
Financial literacy: knowledge of products	.15	.07	-2647.35	5440	.13	.09	-1994.02	3988
Financial attitudes and behaviour	.06	.07	-2695.96	5439	.00	.09	-1961.40	3987
	Family-based				Unrelated samples			
Functional outcome	SNP-h^2	SE	SNP-h^2	SE	SNP-h^2	SE	SNP-h^2	SE
Wellbeing factor (1st PC)	.04	.13	-1359.09	2703	.04	.13	-1358.65	2701
P factor (1st PC)	.20	.17	-1097.11	2142	.20	.17	-1096.09	2139
Health factor (1st PC)	.00	.16	-1232.74	2292	.00	.16	-1232.50	2289
Achieved educational level	.01	.07	-2607.07	5546	.09	.09	-1979.12	4028
Planned educational level	.14	.10	-1642.97	3641	.24	.12	-1409.25	2956

Note. SE= standard error; LogL= log likelihood; N= sample size.

Table S15. Bivariate GREML results.

	Bivariate GREML results																			
	Unrelated samples																			
	Functional outcomes																			
	Wellbeing				Adverse mental health				Adverse physical health				Achieved educational level				Planned educational level			
Trait	rG	SE	LogL	N	rG	SE	LogL	N	rG	SE	LogL	N	rG	SE	LogL	N	rG	SE	LogL	N
Daily hassles	-1.00	0.56	-2739.61	5716	1.00	0.67	-2377.47	5304	1.00	0.97	-2691.59	5422	-0.30	0.67	-3739.67	7627	-0.74	0.52	-3070.06	6363
CHAOS	0.11	0.57	-2883.73	6019	-0.97	1.47	-2687.30	5593	-0.06	0.59	-2865.27	5709	0.35	0.56	-3961.66	8083	-0.50	0.40	-3250.38	6725
Childhood experiences	0.54	0.57	-2767.14	5715	-1.00	7.05	-2554.95	5306	-1.00	4.17	-2716.06	5422	-0.71	0.59	-3744.28	7630	-0.52	0.45	-3080.20	6366
Poor sleep quality	0.37	0.77	-2744.32	5666	1.00	0.99	-2361.02	5254	-0.25	1.97	-2573.29	5374	1.00	1.18	-3693.22	7560	-0.73	0.78	-3024.94	6301
Marriage hopes	0.71	0.54	-2964.66	5998	-0.72	0.89	-2810.50	5568	0.18	0.97	-2931.40	5684	-0.44	0.69	-3997.05	8079	-0.20	0.48	-3275.37	6710
Marriage worries	-1.00	2.66	-2909.27	5997	0.17	0.98	-2776.09	5567	-1.00	2.63	-2928.96	5683	1.00	2.98	-4034.90	8078	1.00	1.26	-3281.90	6709
Quality of relationship with twin	-0.27	0.38	-2955.23	5959	0.28	0.68	-2781.59	5530	-0.88	1.22	-2914.63	5647	-0.26	0.48	-3984.54	8026	-0.17	0.34	-3266.47	6676
Quality of relationship with mother	-0.13	0.66	-2910.16	5928	0.50	1.43	-2726.78	5500	1.00	2.57	-2881.56	5617	-0.56	0.87	-3947.93	7988	0.05	0.60	-3227.53	6641
Quality of relationship with father	0.22	0.61	-2859.19	5865	0.26	1.70	-2677.81	5433	0.09	1.72	-2850.92	5549	0.54	0.57	-3900.40	7912	0.36	0.40	-3199.09	6575
Number of relationships	0.16	0.40	-2898.47	5997	-0.02	0.55	-2716.90	5567	0.51	1.89	-2819.90	5683	-1.00	0.81	-3926.55	8078	-0.46	0.36	-3189.10	6709
Longest relationship	-0.18	0.96	-2748.72	5653	0.61	1.41	-2598.08	5215	-0.43	1.62	-2708.37	5329	-1.00	1.10	-3668.39	7494	-0.71	0.88	-3008.86	6242
Partner violence	0.07	0.49	-2927.00	5927	0.98	0.76	-2713.74	5494	1.00	80.06	-2885.35	5606	-0.35	0.54	-3951.16	7960	-0.54	0.44	-3262.48	6631
Contact with mother	1.00	1.87	-2093.93	4241	1.00	1.96	-1861.14	3744	1.00	12.92	-2013.62	3880	-1.00	1.98	-2779.81	5736	-0.26	0.73	-2203.06	4661
Communication with mother	1.00	0.97	-2119.14	4276	-0.71	1.41	-1888.19	3780	1.00	10.19	-2035.48	3915	-0.83	0.92	-2849.76	5775	-0.67	0.84	-2276.94	4702
Contact with father	1.00	2.84	-2115.33	4358	1.00	23.22	-1895.94	3874	-1.00	1653 26.26	-2045.25	4005	-1.00	5.45	-2820.35	5882	-1.00	97.77	-2240.86	4783
Communication with father	1.00	2246. 15	-2199.38	4506	1.00	1969 32.82	-1988.65	4029	-1.00	1444 47.49	-2137.22	4158	1.00	3.60	-2949.42	6052	log likelihood not coverged within 100 iterations			

Peer pressure	1.00	2.07	-2983.73	5973	-1.00	7.95	-2787.55	5550	-1.00	1685 29.38	-2993.24	5659	1.00	29.00	-4041.78	8033	-1.00	7.27	-3315.26	6678
Physical peer victimisation	1.00	1.13	-2771.77	5647	-1.00	2.29	-2590.71	5230	0.18	9.80	-2733.65	5351	-0.02	0.84	-3699.18	7532	0.26	0.68	-3042.70	6276
Social peer victimisation	0.28	0.60	-2777.15	5647	0.99	0.44	-2487.26	5230	0.58	0.87	-2713.35	5351	-0.23	0.57	-3740.31	7532	-0.19	0.44	-3073.28	6276
Verbal peer victimisation	0.59	0.82	-2864.08	5645	1.00	0.91	-2589.89	5228	0.96	1.20	-2783.10	5349	-0.22	0.78	-3811.11	7532	-0.48	0.76	-3154.33	6276
Cyber peer victimisation	0.16	0.48	-2869.82	5645	0.82	0.69	-2607.49	5228	0.92	1.86	-2795.31	5349	-0.22	0.56	-3820.62	7530	-0.18	0.41	-3158.93	6274
Physical peer perpetration	0.66	1.23	-2914.01	5645	-1.00	2.29	-2742.51	5228	-1.00	2.12	-2872.98	5349	-0.22	1.64	-3822.19	7532	0.57	2.35	-3181.46	6276
Social peer perpetration	-0.02	0.58	-2621.86	5644	0.73	0.95	-2380.97	5227	-0.14	1.26	-2552.32	5348	-0.26	0.77	-3534.56	7531	-0.44	0.57	-2901.04	6275
Verbal peer perpetration	-1.00	77.84	-2842.33	5644	1.00	4.36	-2647.52	5227	1.00	5.13	-2790.79	5348	-1.00	29.27	-3756.96	7531	-1.00	3.46	-3101.43	6275
Cyber peer perpetration	-0.34	0.35	-2945.86	5644	-0.08	0.50	-2755.48	5227	-0.51	1.20	-2881.45	5348	log likelihood not coverged within 100 iterations				0.27	0.39	-3202.89	6275
Parental role aspirations	1.00	1.35	-2863.25	5720	1.00	31.18	-2691.70	5311	1.00	4.09	-2824.22	5427	0.02	0.45	-3850.10	7531	-1.00	12.41	-3141.58	6370
Occupational role aspirations	1.00	39.19	-2793.30	5721	1.00	3.29	-2620.99	5312	1.00	4.27	-2744.90	5428	-1.00	1689. 99	-3679.48	7635	-1.00	12.59	-3031.71	6372
Homecare role aspirations	1.00	0.62	-2767.82	5721	0.44	1.02	-2618.23	5312	-0.05	1.08	-2745.04	5428	-1.00	1.23	-3749.80	7635	-0.74	0.57	-3053.08	6372
Importance of relationships	0.47	0.72	-2927.17	6039	0.85	1.52	-2771.22	5611	1.00	507.7 3	-2898.97	5726	-0.25	0.79	-3951.54	8100	-0.51	0.59	-3236.35	6741
Achievement motivation	0.90	0.67	-2977.52	6038	0.08	0.77	-2809.41	5610	-1.00	4.72	-2941.90	5725	-0.06	0.64	-3980.46	8099	0.80	0.62	-3265.01	6740
Purpose in life	0.66	0.36	-2820.97	6038	-0.31	0.71	-2546.39	5610	1.00	28.93	-2901.23	5725	-0.93	0.83	-4009.97	8099	-0.35	0.44	-3289.64	6739
Importance of democracy and equality	-1.00	1.02	-2993.78	6034	0.77	1.35	-2816.62	5605	1.00	10.70	-2942.61	5720	1.00	1.05	-3949.76	8096	-0.10	0.73	-3259.52	6735
Environmental concerns	-0.22	0.40	-3004.03	6038	0.58	0.72	-2827.32	5609	1.00	12.04	-2945.20	5724	0.81	0.49	-3932.61	8100	0.42	0.33	-3243.83	6739
Religiosity	1.00	21.11	-2641.02	5975	1.00	1.91	-2466.99	5552	1.00	11.70	-2601.55	5661	-1.00	5.28	-3646.42	8035	-1.00	77.02	-2921.66	6679
Importance of leisure	0.56	0.29	-2712.01	5665	-0.27	0.43	-2472.71	5255	-0.13	1.45	-2714.58	5371	0.61	0.50	-3686.71	7564	0.24	0.33	-3037.22	6299
Alcohol use	0.93	3.45	-2577.93	5473	1.00	9.21	-2404.25	5049	-1.00	104.9 6	-2611.44	5168	1.00	4.52	-3499.60	7274	1.00	0.96	-2844.07	6082
Ever smoked	1.00	2.09	-2804.21	5680	1.00	3.72	-2616.19	5264	1.00	14.62	-2748.28	5383	-1.00	15.28	-3725.30	7578	1.00	1.65	-3068.14	6322

Ever vaped	-1.00	1.63	-2871.76	5690	1.00	2.29	-2710.45	5274	1.00	4.84	-2804.16	5393	-0.06	1.28	-3758.01	7572	0.07	0.79	-3119.27	6324
Cognitive enhancers	-0.11	0.61	-2759.04	5666	-0.14	1.88	-2592.03	5248	-1.00	9.05	-2775.75	5372	1.00	1.59	-3691.31	7542	1.00	0.76	-3041.72	6295
Big 5 Agreeableness	-1.00	2.52	-2935.80	5970	1.00	6.11	-2775.80	5563	-1.00	2.97	-2856.83	5652	-1.00	68.46	-3903.77	7955	-1.00	2.72	-3214.46	6639
Big 5 Conscientiousness	1.00	9.89	-2867.58	5965	-1.00	1.50	-2685.41	5558	-1.00	3.67	-2810.44	5647	-1.00	3.32	-3885.34	7951	-1.00	3.46	-3180.01	6635
Big 5 Extraversion	0.56	0.36	-2820.79	5985	-0.11	0.37	-2554.72	5578	-0.38	0.50	-2771.96	5667	-0.12	0.53	-3827.90	7968	0.24	0.41	-3143.91	6653
Big 5 Neuroticism	-1.00	0.78	-2825.00	5989	0.45	0.75	-2385.10	5582	0.12	0.76	-2787.52	5671	1.00	1.17	-3921.39	7972	-0.35	0.77	-3218.88	6657
Big 5 Openness	-0.76	0.56	-2901.21	5980	log likelihood not converged within 100 iterations				0.78	0.94	-2847.25	5662	-0.17	0.46	-3878.40	7965	-0.18	0.36	-3194.44	6649
Self-control	1.00	1.89	-2827.77	5965	1.00	2.94	-2863.70	5573	-1.00	4.68	-2751.14	5647	log likelihood not converged within 100 iterations				1.00	1.58	-3163.83	6635
Not planning for the future	-0.46	0.47	-2883.81	5961	-0.15	0.59	-2717.18	5554	0.15	0.79	-2808.27	5643	0.06	0.53	-3847.91	7947	-0.38	0.42	-3144.80	6631
Ambition	0.26	0.52	-2826.34	5716	0.38	0.96	-2646.17	5305	1.00	2.04	-2785.65	5423	-0.39	0.82	-3743.28	7627	0.12	0.52	-3079.21	6363
Physical activity	-1.00	21704.16	-2965.28	5969	-1.00	40.67	-2796.81	5536	1.00	4.28	-2919.23	5654	1.00	3.23	-3979.40	8046	1.00	1.91	-3263.78	6676
Health behaviours	0.58	0.63	-2986.72	5975	0.54	0.92	-2796.89	5544	1.00	2.06	-2927.93	5661	1.00	0.80	-3956.02	8061	1.00	0.77	-3268.17	6688
Risk taking behaviour	-0.79	0.85	-2927.31	5962	1.00	1.58	-2763.49	5556	1.00	3.99	-2832.55	5645	1.00	1.10	-3889.60	7949	1.00	0.95	-3195.08	6633
Risky sexual behaviour	0.84	0.55	-2905.21	5877	0.28	0.57	-2732.49	5452	0.16	0.71	-2839.28	5566	-0.88	0.69	-3894.04	7915	-0.43	0.39	-3181.54	6578
Media use	0.59	1.24	-2911.32	5963	1.00	1.58	-2729.35	5527	1.00	10.49	-2865.00	5643	-1.00	1.69	-3920.59	8033	-1.00	1.16	-3181.49	6667
Volunteering	-0.75	0.55	-2951.22	5977	1.00	0.81	-2779.68	5554	1.00	3.42	-2925.94	5664	0.56	0.53	-3937.45	8038	0.35	0.41	-3230.69	6682
Internet dating (scale)	log likelihood not coverged within 100 iterations				1.00	2.11	-2759.40	5515	1.00	3.46	-2875.94	5632	-1.00	4.51	-3939.61	8020	1.00	12.65	-3220.52	6660
Internet dating (y/n)	-1.00	6.57	-2909.25	5951	1.00	2.08	-2782.50	5523	1.00	3.59	-2901.16	5640	-1.00	9.03	-3971.54	8031	1.00	39.67	-3246.58	6667
Financial literacy: knowledge of products	-0.59	0.96	-2903.79	5948	-0.22	0.64	-2754.70	5492	-0.36	1.12	-2890.28	5605	-0.14	0.58	-3951.91	7971	0.14	0.47	-3228.68	6623
Financial attitudes and behaviour	-1.00	78.76	-2808.21	5948	1.00	11.21	-2705.62	5492	-1.00	7.74	-2844.70	5605	log likelihood not converged within 100 iterations				1.00	8.13	-3187.73	6621

Note. SE= standard error; LogL= log likelihood; N= sample size.

Supplementary Figures

Figure S1. The Bivariate Cholesky Decomposition.

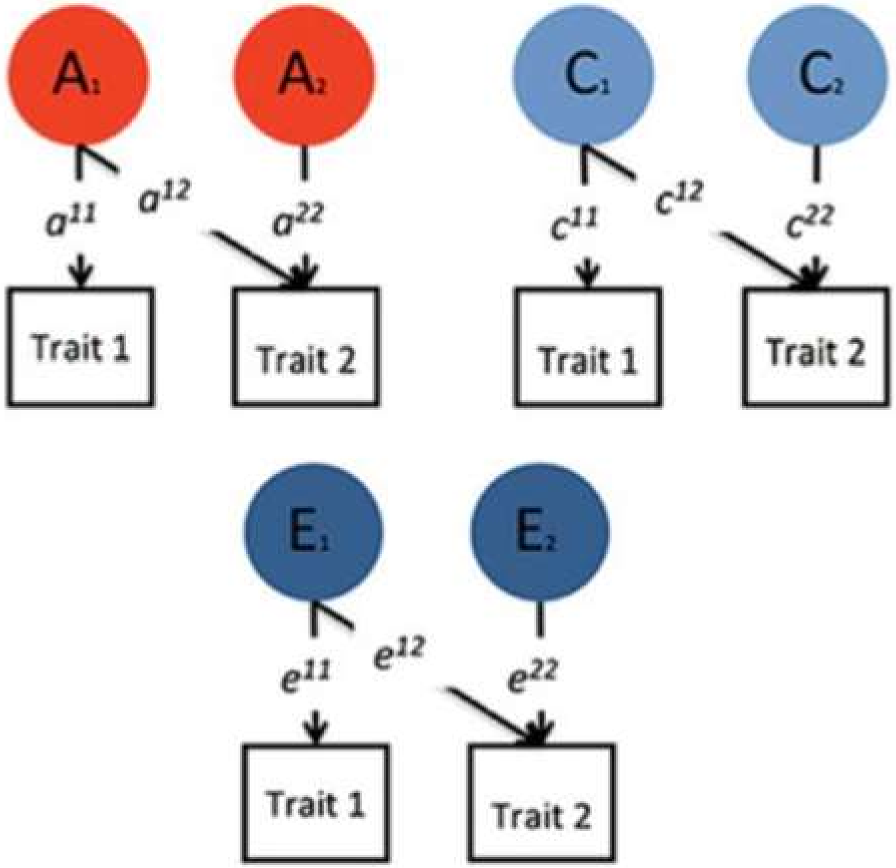

Figure S2. Phenotypic correlations between key outcomes and emerging adulthood variables (indicated by the total length of the bar); bivariate genetic estimates for additive genetic (A), shared environmental (C) and non-shared environmental (E) contributions to these correlations for males (a), females (b) and when excluding DZ opposite sex twin pairs (c).

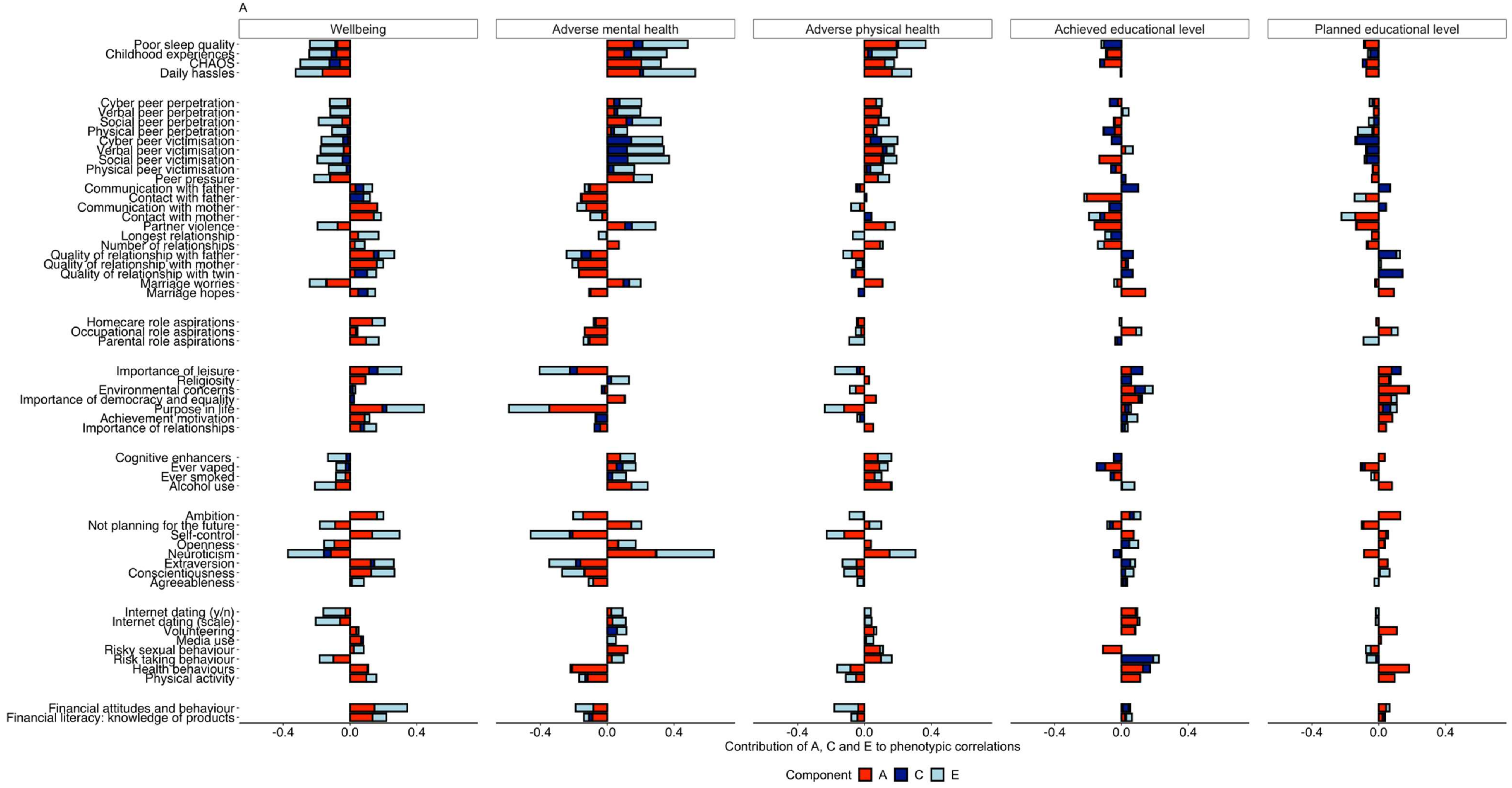

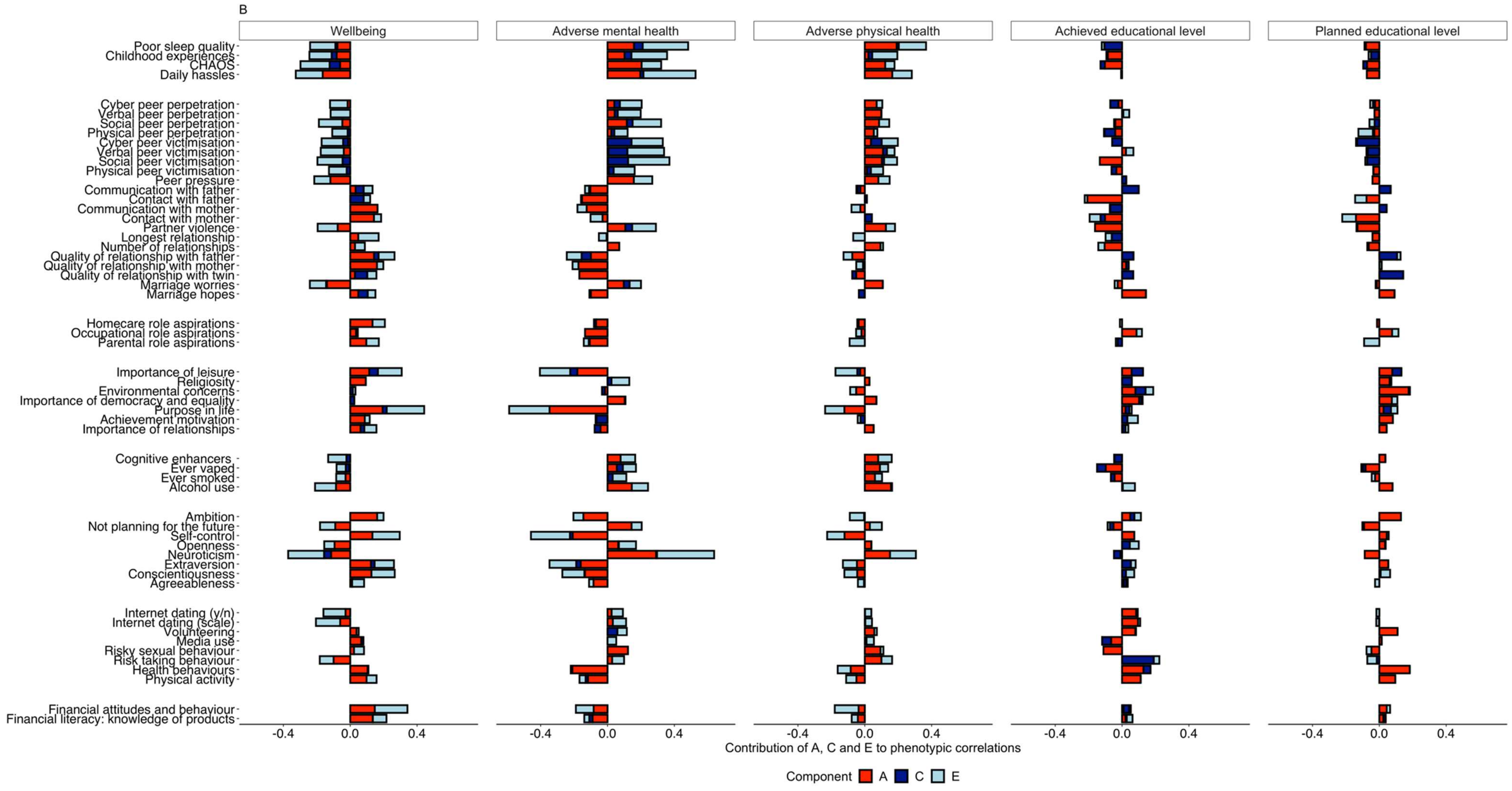
B
Wellbeing
Adverse mental health
Adverse physical health
Achieved educational level
Planned educational level
Poor sleep quality
Childhood experiences
CHAOS
Daily hassles
Cyber peer perpetration
Verbal peer perpetration
Social peer perpetration
Physical peer perpetration
Cyber peer victimisation
Verbal peer victimisation
Social peer victimisation
Physical peer victimisation
Peer pressure
Communication with father
Contact with father
Communication with mother
Contact with mother
Partner violence
Longest relationship
Number of relationships
Quality of relationship with father
Quality of relationship with mother
Quality of relationship with twin
Marriage worries
Marriage hopes
Homecare role aspirations
Occupational role aspirations
Parental role aspirations
Importance of leisure
Religiosity
Environmental concerns
Importance of democracy and equality
Purpose in life
Achievement motivation
Importance of relationships
Cognitive enhancers
Ever vaped
Ever smoked
Alcohol use
Ambition
Not planning for the future
Self-control
Openness
Neuroticism
Extraversion
Conscientiousness
Agreeableness
Internet dating (y/n)
Internet dating (scale)
Volunteering
Media use
Risky sexual behaviour
Risk taking behaviour
Health behaviours
Physical activity
Financial attitudes and behaviour
Financial literacy: knowledge of products
-0.4
0.0
0.4
Contribution of A, C and E to phenotypic correlations
Component
A
C
E

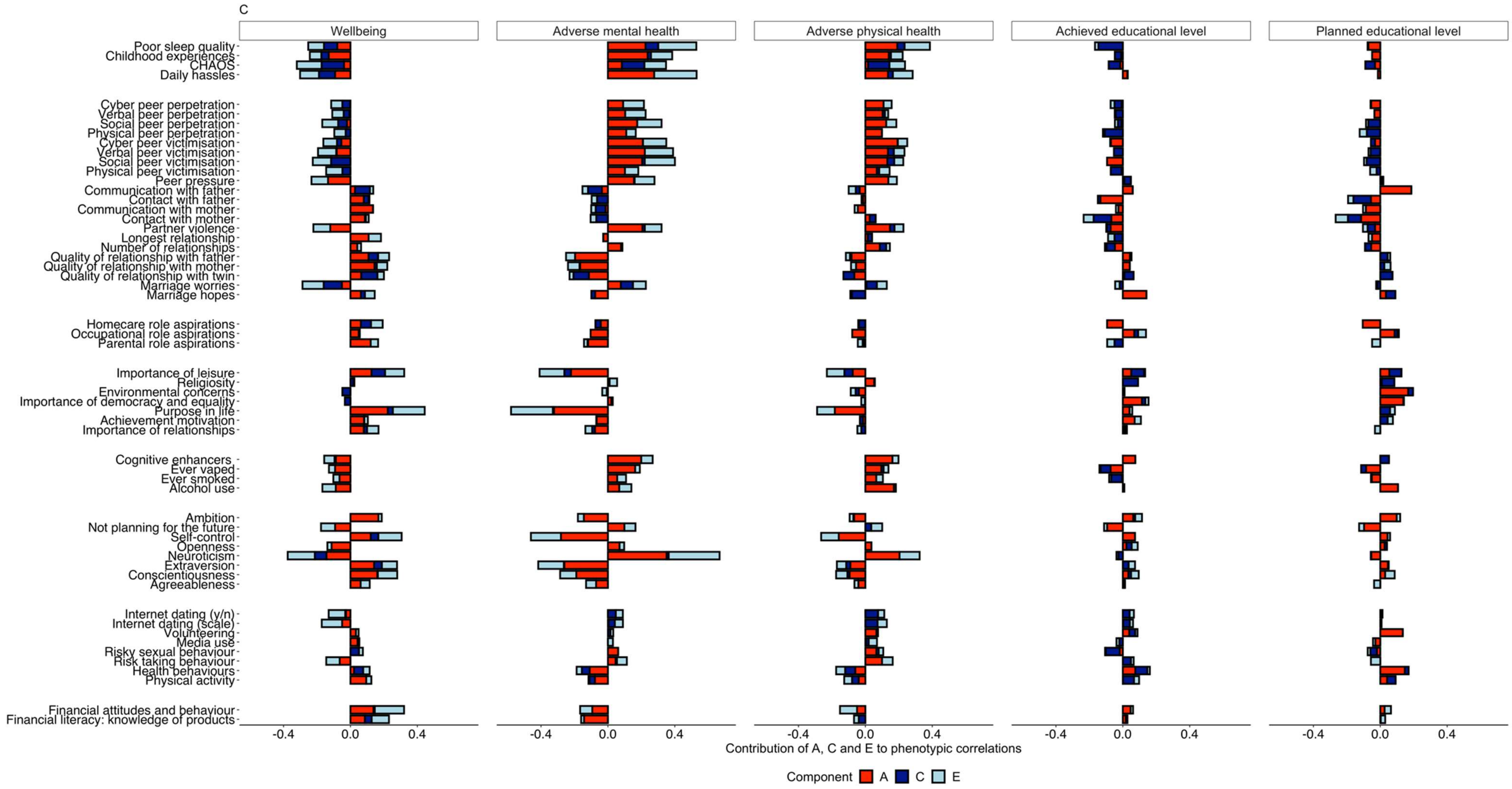
C
Wellbeing
Adverse mental health
Adverse physical health
Achieved educational level
Planned educational level
Poor sleep quality
Childhood experiences
CHAOS
Daily hassles
Cyber peer perpetration
Verbal peer perpetration
Social peer perpetration
Physical peer perpetration
Cyber peer victimisation
Verbal peer victimisation
Social peer victimisation
Physical peer victimisation
Peer pressure
Communication with father
Contact with father
Communication with mother
Contact with mother
Partner violence
Longest relationship
Number of relationships
Quality of relationship with father
Quality of relationship with mother
Quality of relationship with twin
Marriage worries
Marriage hopes
Homecare role aspirations
Occupational role aspirations
Parental role aspirations
Importance of leisure
Religiosity
Environmental concerns
Importance of democracy and equality
Purpose in life
Achievement motivation
Importance of relationships
Cognitive enhancers
Ever vaped
Ever smoked
Alcohol use
Ambition
Not planning for the future
Self-control
Openness
Neuroticism
Extraversion
Conscientiousness
Agreeableness
Internet dating (y/n)
Internet dating (scale)
Volunteering
Media use
Risky sexual behaviour
Risk taking behaviour
Health behaviours
Physical activity
Financial attitudes and behaviour
Financial literacy: knowledge of products
-0.4
0.0
0.4
Contribution of A, C and E to phenotypic correlations
Component A C E

Figure S3. Twin correlations for psychological and behavioural traits and key functional outcomes for the whole sample.

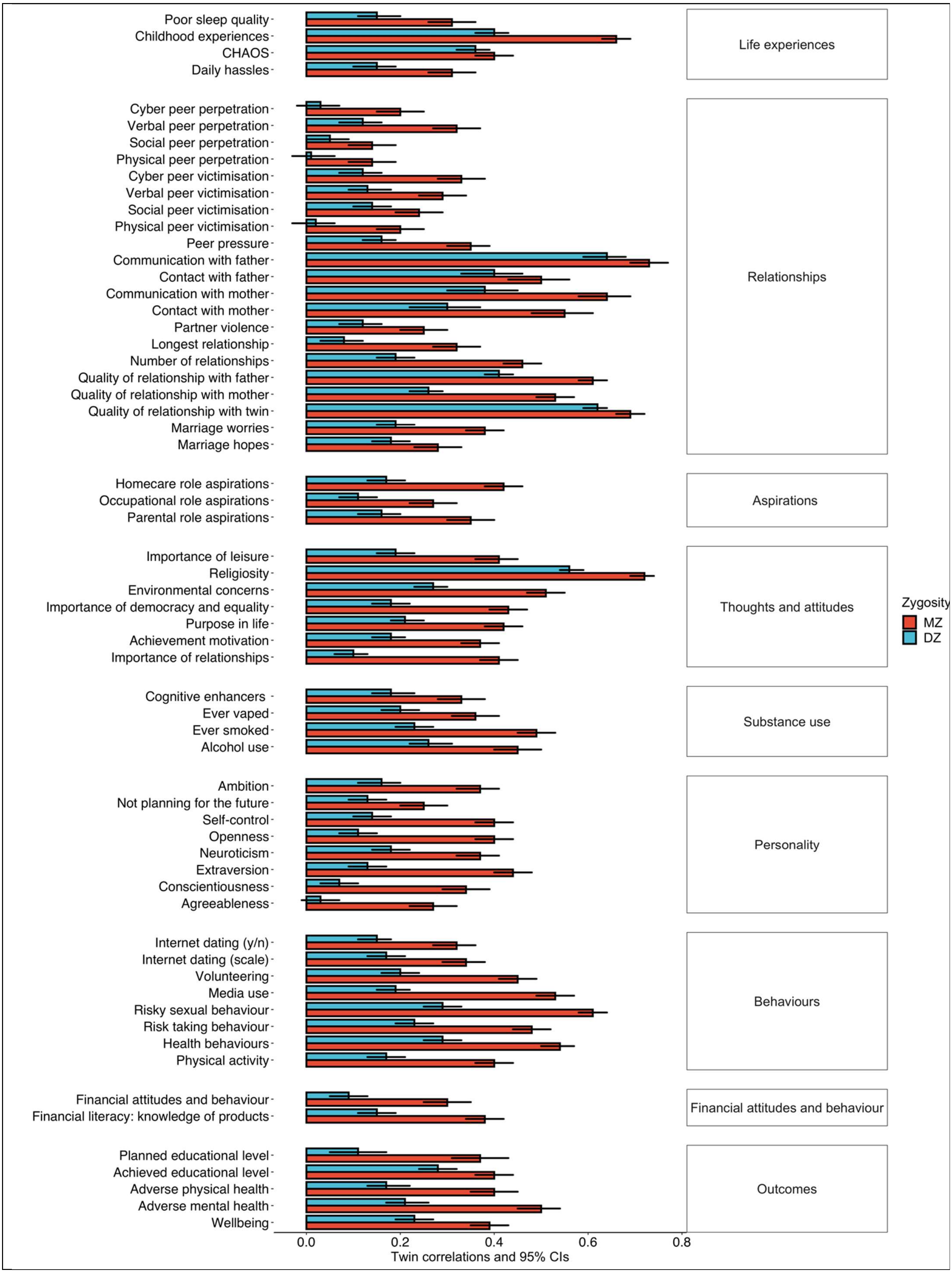

Figure S4. Univariate analyses of additive genetic (A), shared environmental (C), and non-shared environmental (E) components of variance for variables for psychological traits and functional outcomes for when calculated for males and females separately (a) and for the whole sample when opposite sex DZ twin pairs were excluded (b).

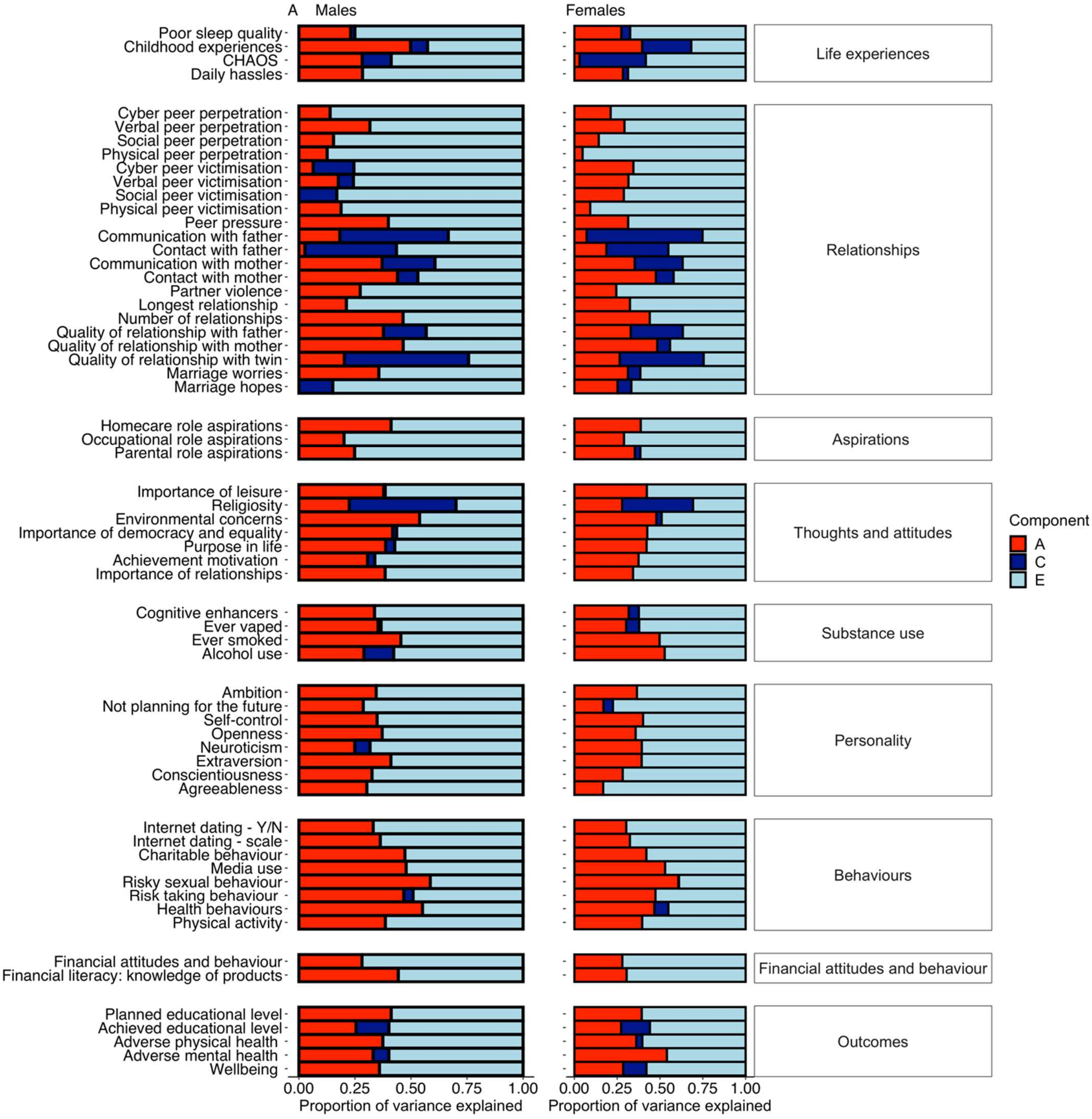

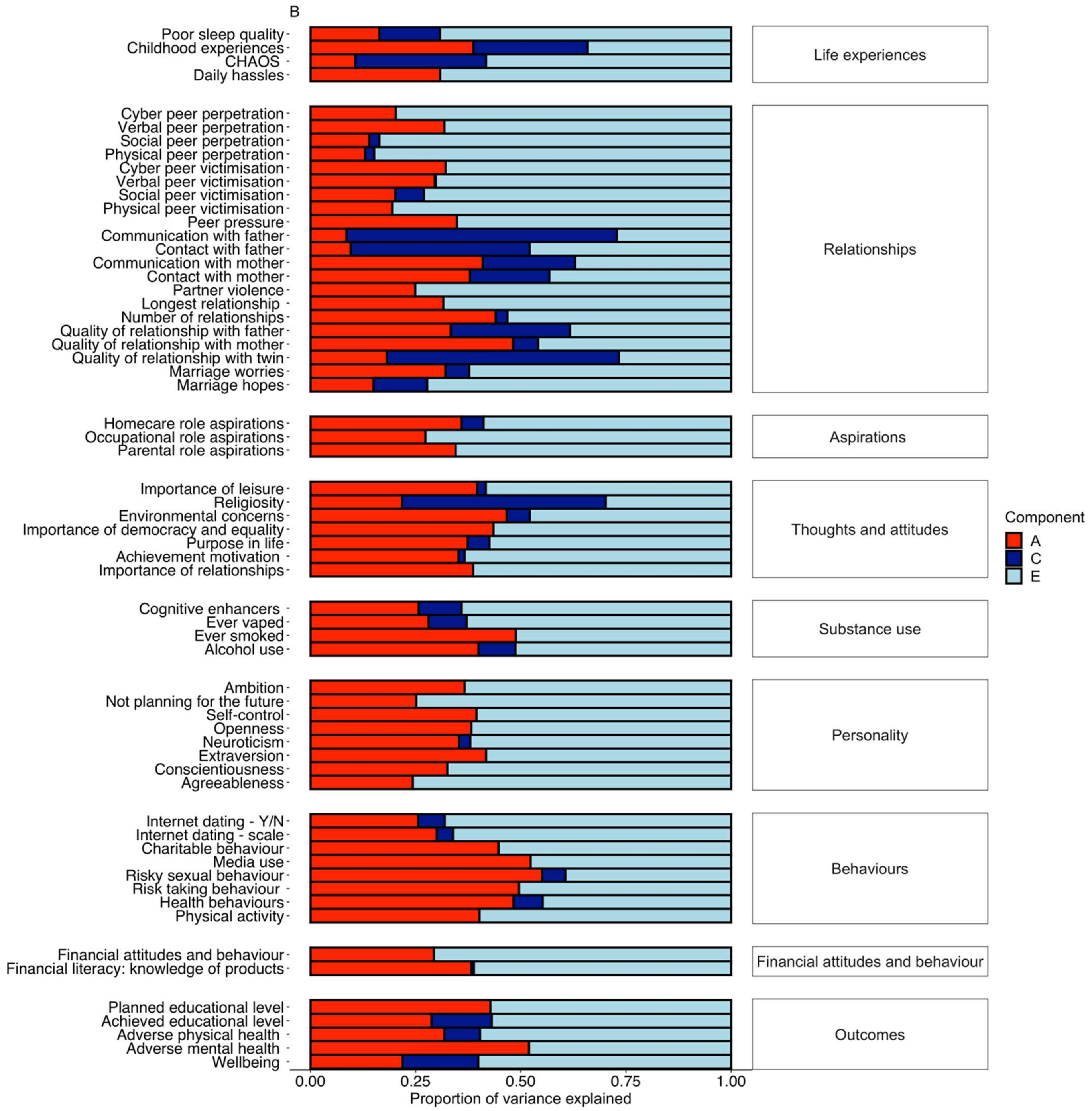
B
Poor sleep quality
Childhood experiences
CHAOS
Daily hassles
Life experiences
Cyber peer perpetration
Verbal peer perpetration
Social peer perpetration
Physical peer perpetration
Cyber peer victimisation
Verbal peer victimisation
Social peer victimisation
Physical peer victimisation
Peer pressure
Communication with father
Contact with father
Communication with mother
Contact with mother
Partner violence
Longest relationship
Number of relationships
Quality of relationship with father
Quality of relationship with mother
Quality of relationship with twin
Marriage worries
Marriage hopes
Relationships
Homecare role aspirations
Occupational role aspirations
Parental role aspirations
Aspirations
Importance of leisure
Religiosity
Environmental concerns
Importance of democracy and equality
Purpose in life
Achievement motivation
Importance of relationships
Thoughts and attitudes
Cognitive enhancers
Ever vaped
Ever smoked
Alcohol use
Substance use
Ambition
Not planning for the future
Self-control
Openness
Neuroticism
Extraversion
Conscientiousness
Agreeableness
Personality
Internet dating - Y/N
Internet dating - scale
Charitable behaviour
Media use
Risky sexual behaviour
Risk taking behaviour
Health behaviours
Physical activity
Behaviours
Financial attitudes and behaviour
Financial literacy: knowledge of products
Financial attitudes and behaviour
Planned educational level
Achieved educational level
Adverse physical health
Adverse mental health
Wellbeing
Outcomes
0.00
0.25
0.50
0.75
1.00
Proportion of variance explained
Component
A
C
E

Figure S5. Genetic correlations between psychological and behavioural traits and key functional outcomes for the males (a), females (b) and for the whole sample when opposite sex DZ twin were excluded (c)

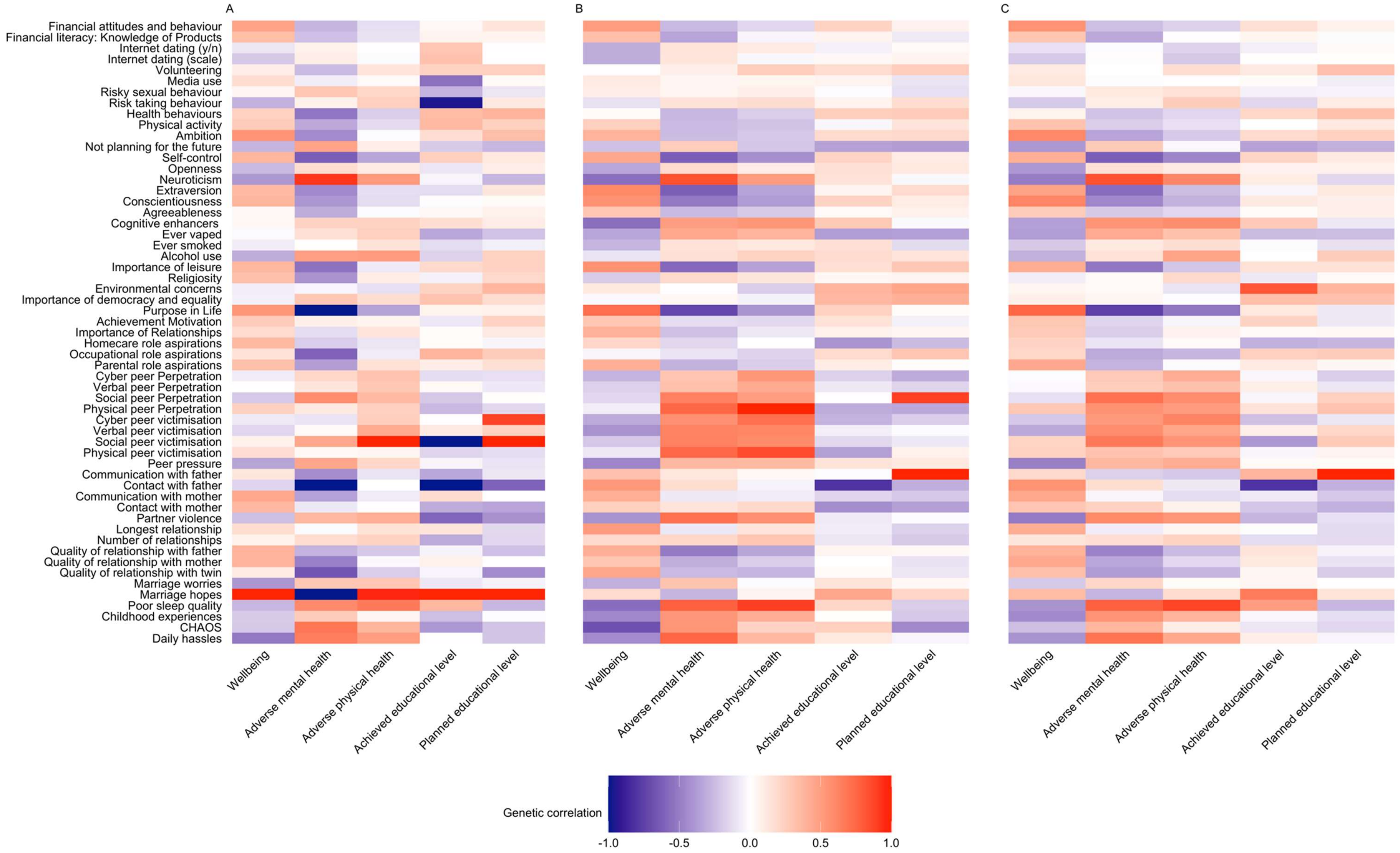

Figure S6. Shared environmental correlations between psychological and behavioural traits and key functional outcomes (a) for the whole sample; (b) MZ and DZ same sex; (c) males only; (d) females only.

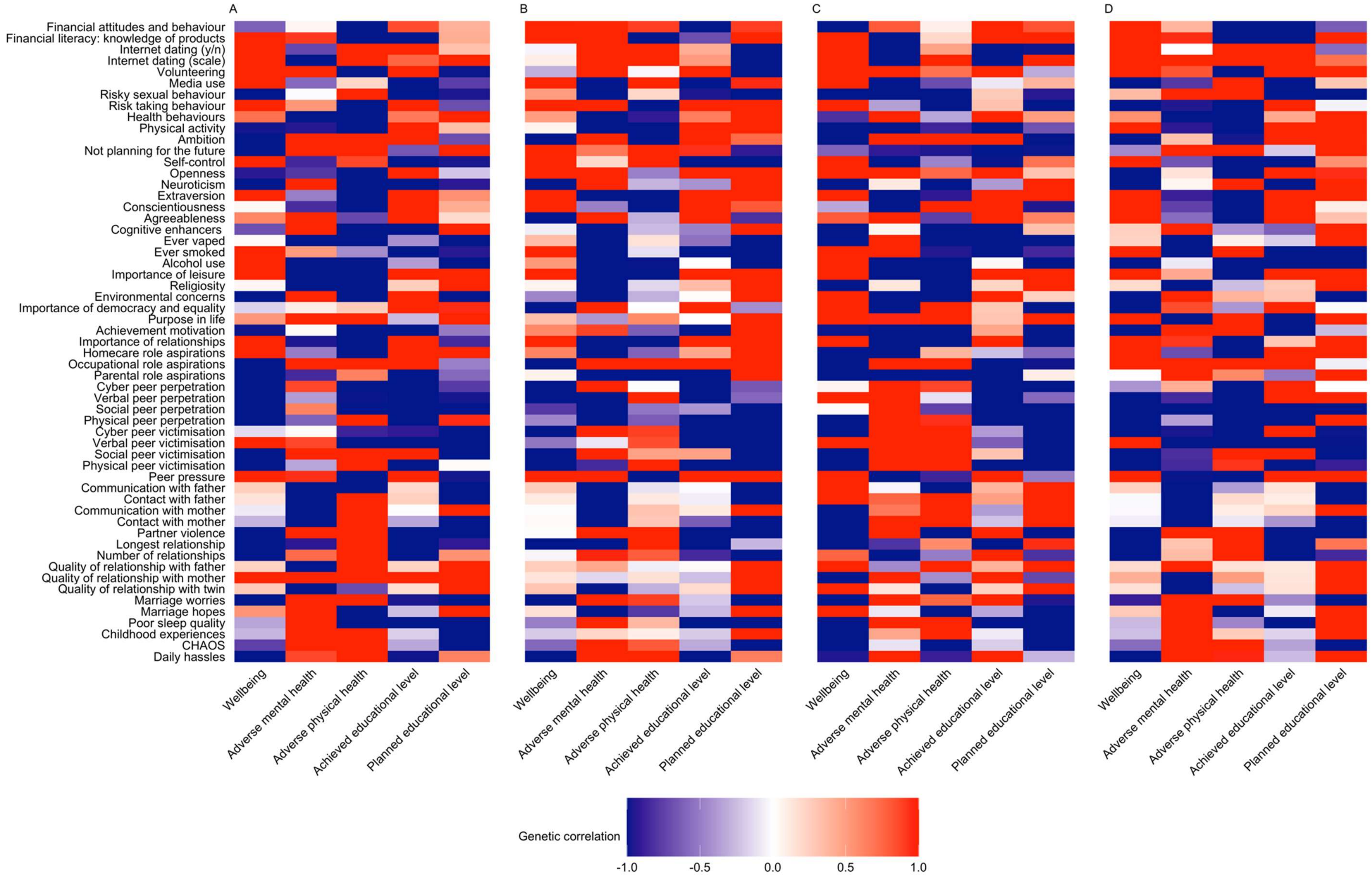

Figure S7. Non-shared environmental correlations between psychological and behavioural traits and key functional outcomes (a) for the whole sample; (b) MZ and DZ same sex twin pairs only; (c) males only; (d) females only

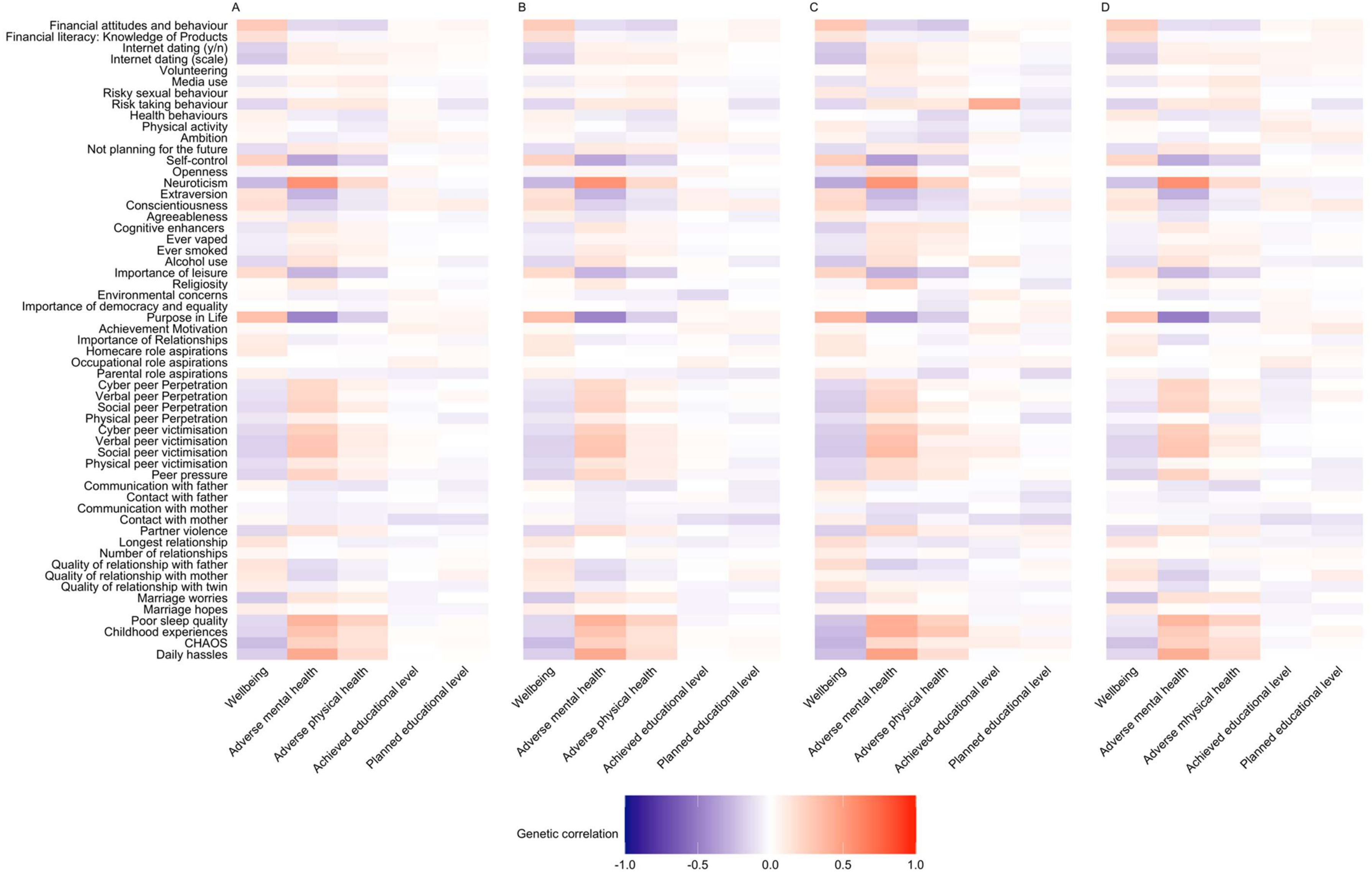

Figure S8. Univariate GREML analyses; SNP heritabilities for psychological traits and functional outcomes for the whole sample (standard errors represented in error bars; reml-no-constrain option indicated by the point estimates below 0).

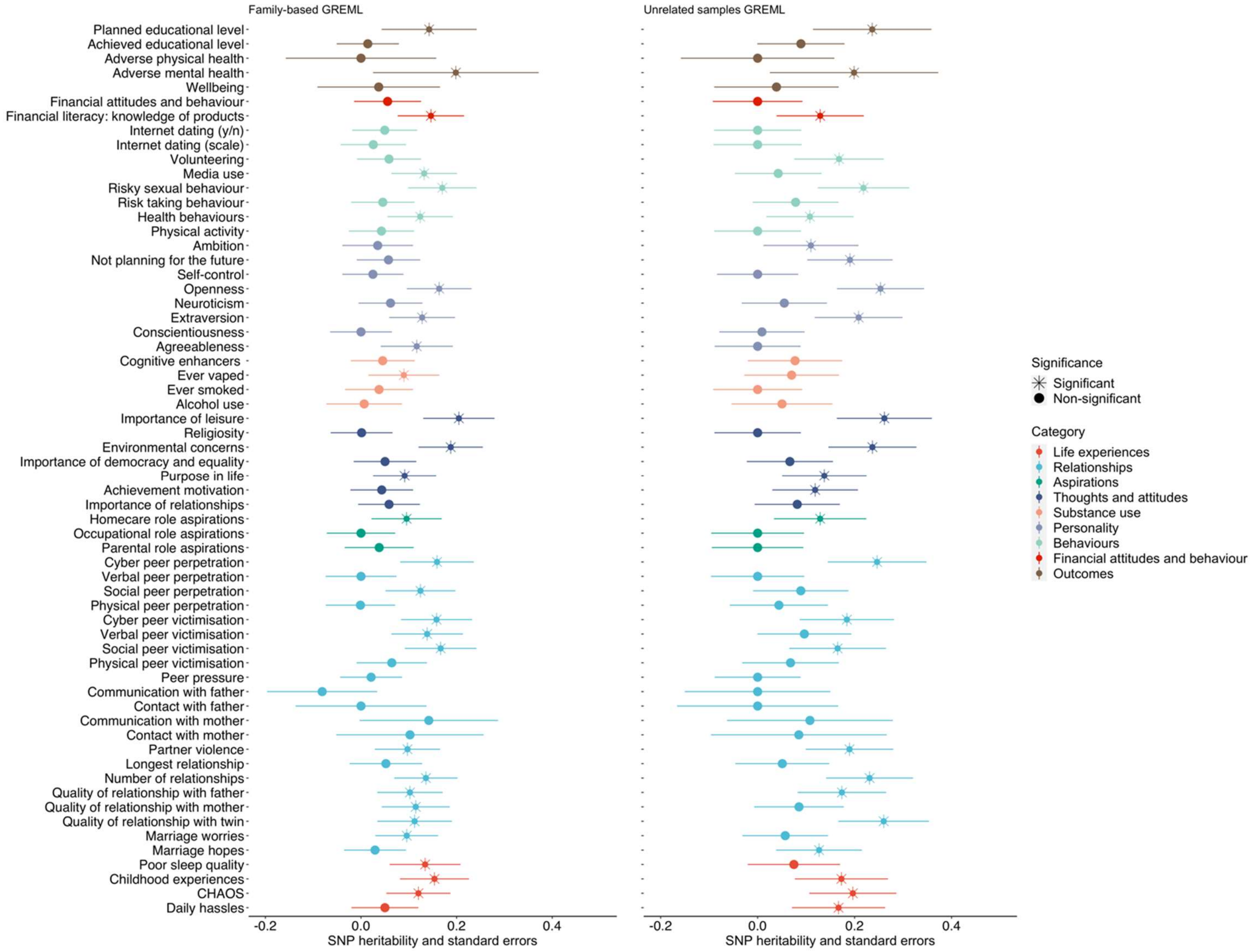

Figure S9. Univariate twin analyses of additive genetic (A), shared environmental (C), and non-shared environmental (E) components of variance for variables for psychological traits and functional outcomes for the whole sample for untransformed data and when using Van der Waerden transformation

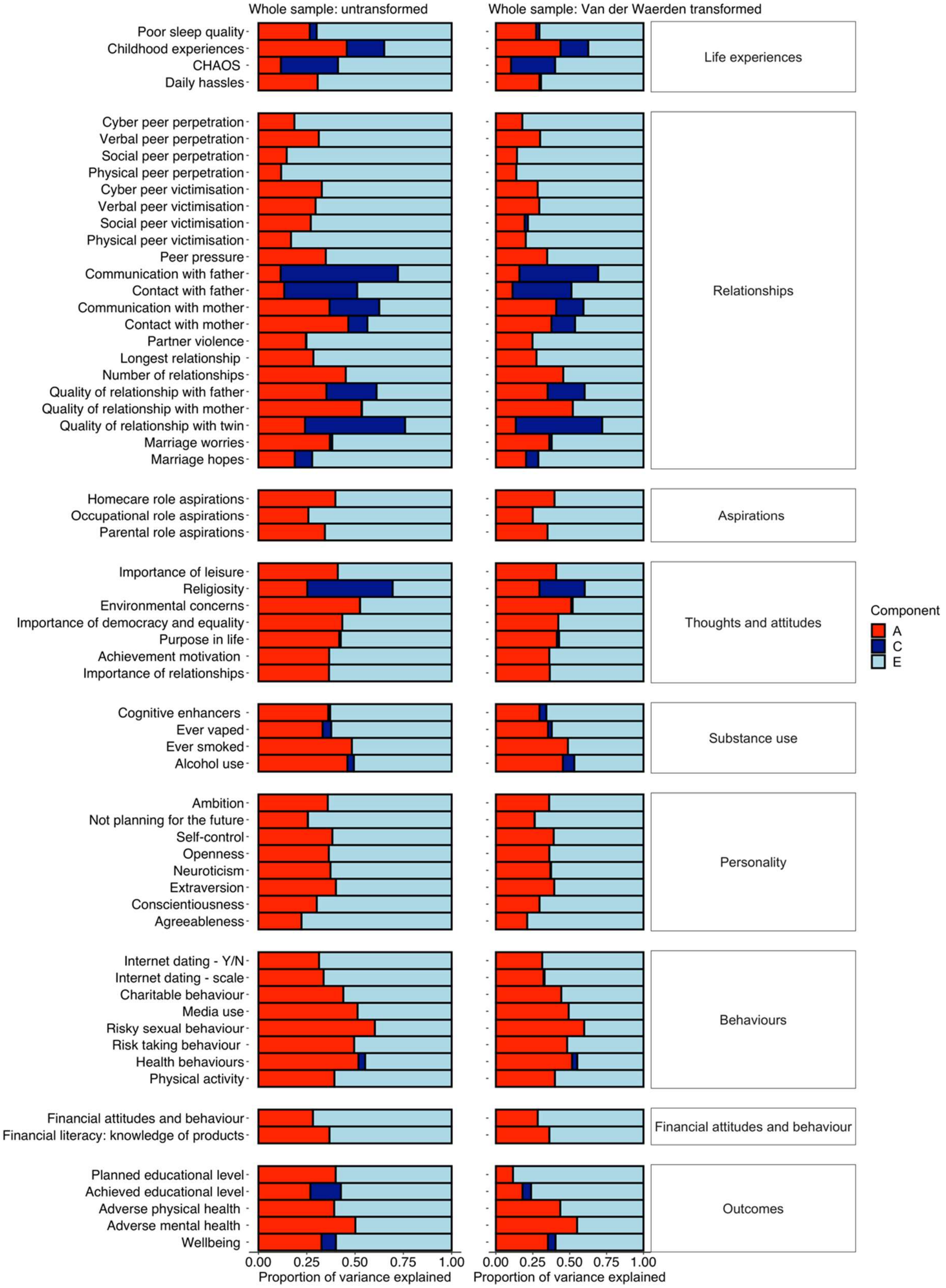

Figure S10. Univariate GREML analyses; SNP heritabilities for psychological traits and functional outcomes for the whole sample for untransformed data and when using Van der Waerden transformation (standard errors represented in error bars).

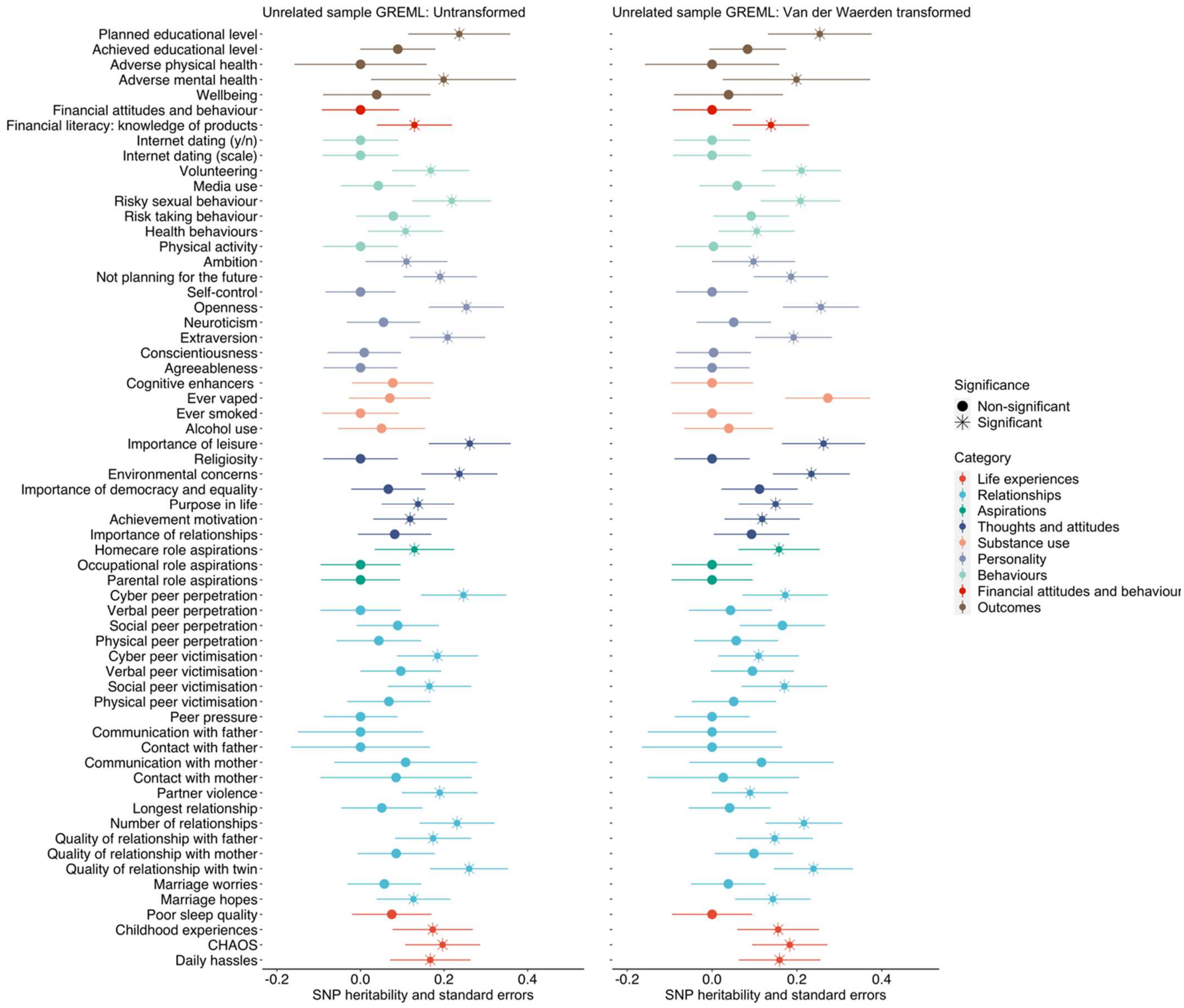
Unrelated sample GREML: Untransformed
Unrelated sample GREML: Van der Waerden transformed
Planned educational level
Achieved educational level
Adverse physical health
Adverse mental health
Wellbeing
Financial attitudes and behaviour
Financial literacy: knowledge of products
Internet dating (y/n)
Internet dating (scale)
Volunteering
Media use
Risky sexual behaviour
Risk taking behaviour
Health behaviours
Physical activity
Ambition
Not planning for the future
Self-control
Openness
Neuroticism
Extraversion
Conscientiousness
Agreeableness
Cognitive enhancers
Ever vaped
Ever smoked
Alcohol use
Importance of leisure
Religiosity
Environmental concerns
Importance of democracy and equality
Purpose in life
Achievement motivation
Importance of relationships
Homecare role aspirations
Occupational role aspirations
Parental role aspirations
Cyber peer perpetration
Verbal peer perpetration
Social peer perpetration
Physical peer perpetration
Cyber peer victimisation
Verbal peer victimisation
Social peer victimisation
Physical peer victimisation
Peer pressure
Communication with father
Contact with father
Communication with mother
Contact with mother
Partner violence
Longest relationship
Number of relationships
Quality of relationship with father
Quality of relationship with mother
Quality of relationship with twin
Marriage worries
Marriage hopes
Poor sleep quality
Childhood experiences
CHAOS
Daily hassles
-0.2
0.0
0.2
0.4
SNP heritability and standard errors
Significance
Non-significant
Significant
Category
Life experiences
Relationships
Aspirations
Thoughts and attitudes
Substance use
Personality
Behaviours
Financial attitudes and behaviour
Outcomes
